# Synthesis and Facile Dearomatization of Highly Electrophilic Nitroisoxazolo[4,3-*b*]pyridines

**DOI:** 10.3390/molecules25092194

**Published:** 2020-05-08

**Authors:** Maxim A. Bastrakov, Alexey K. Fedorenko, Alexey M. Starosotnikov, Ivan V. Fedyanin, Vladimir A. Kokorekin

**Affiliations:** 1 N.D. Zelinsky Institute of Organic Chemistry RAS; Leninsky prosp. 47, 119991 Moscow, Russia; alexeyfedorenko21@mail.ru (A.K.F.); alexey41@list.ru (A.M.S.); kokorekin@yandex.ru (V.A.K.); 2 Chemistry Department, Lomonosov Moscow State University, Leninskie gory, 1/3, 119991 Moscow, Russia; 3 A.N. Nesmeyanov Institute of Organoelement Compounds, Vavilova str. 28, 119991 Moscow, Russia; octy@xrlab.ineos.ac.ru

**Keywords:** nitro group, nitropyridines, isoxazolo[4,3-*b*]pyridines, 1,4-dihydropyridines, nucleophilic addition, Diels-Alder reaction, dearomatization

## Abstract

A number of novel 6-R-isoxazolo[4,3-*b*]pyridines were synthesized and their reactions with neutral C-nucleophiles (1,3-dicarbonyl compounds, π-excessive (het)arenes, dienes) were studied. The reaction rate was found to be dependent on the nature of the substituent 6-R. The most reactive 6-nitroisoxazolo[4,3-*b*]pyridines are able to add C-nucleophiles in the absence of a base under mild conditions. In addition, these compounds readily undergo [4+2]-cycloaddition reactions on aromatic bonds C=C(NO_2_) of the pyridine ring, thus indicating the superelectrophilic nature of 6-NO_2_-isoxazolo[4,3-*b*]pyridines.

## 1. Introduction

The nitro group is considered to be a versatile and unique functional group in organic chemistry. Synthetic and natural compounds containing nitro groups display great structural diversity [1,2], and they exhibit a wide range of biological activities [3] including antibiotic [1], antitumor [1,4], and anti-HIV activities [5,6,7]. In addition, nitroarenes are used as agrochemical preparations [8,9], energetic compounds [10] and in the production of innovative materials [11].

It is well known that the introduction of one or more nitro groups in aromatic or heteroaromatic nucleus increases the electron-deficient character of the molecule. Such compounds have been extensively studied in recent decades due to their interesting, sometimes exceptional, properties. Their high susceptibility to undergoing nucleophilic addition or substitution processes with very weak nucleophiles has raised considerable interest, leading to numerous synthetic, biological, and analytical applications [12,13,14,15,16,17,18,19,20,21,22,23,24,25,26,27,28,29,30,31].

**Figure 1:**
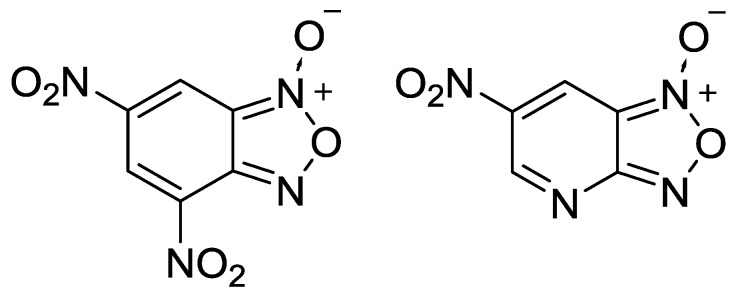
Selected examples of superelectrophiles.

Such compounds possess extremely high reactivity towards carbon and heteroatomic nucleophiles, therefore a special term, “superelectrophile”, was coined in order to distinguish them from other electrophilic aromatics [26,32]. Typical examples of such compounds are given below (Figure 1).

In addition, these compounds are capable to undergo [4+2]-cycloadditions to the C=C(NO_2_) aromatic bond, behaving as electron-poor dienophiles with dienes, or as heterodienes with electron-rich dienophiles within normal or inverse electronic demands, respectively [16,33,34,35]. The above-mentioned interactions with nucleophiles or dienes resulted in dearomatization of the initial aromatic nitro compound. At the same time, dearomatization as a method of converting accessible, cheap, and simple aromatic compounds into more saturated, inaccessible and promising intermediates of greater molecular complexity is a very important approach in modern organic chemistry [36,37].

**Scheme 1:**
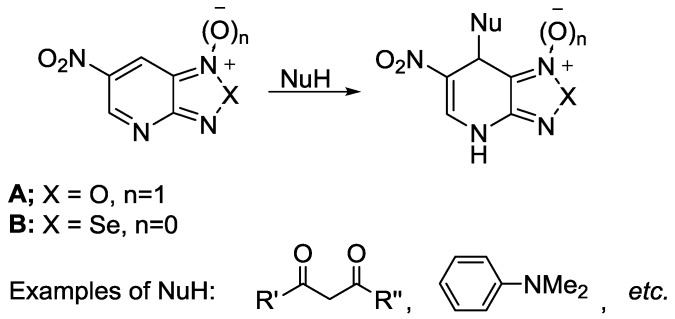
Reactions of condensed nitropyridines with nucleophiles.

This work is part of our ongoing research on highly electrophilic systems and the application of the dearomatization strategy in the synthesis of new polyfunctional azaheterocycles [38,39,40,41,42,43,44,45,46,47,48]. We have previously shown that nitropyridines fused with π-deficient heterocycles (furoxan **A**, selenadiazole **B**), Scheme 1, react with neutral nucleophiles with the formation of 1,4-addition products—dihydropyridine derivatives [45,46,48].

**Figure 2:**
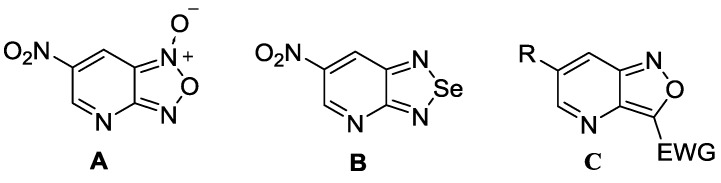
Pyridines fused with high-electrophilic heterocycles.

Another possible condensed pyridines structurally close to heterocyclic systems **A** and **B** and presumably having a similar electron-deficient character are isoxazolo[4,3-*b*]pyridines **C**, Figure 2. The present work is devoted to the synthesis of pyridine derivatives condensed with an isoxazole ring and study of their interaction with various neutral C-nucleophiles as well as their behavior in [4+2]-cycloaddition reactions.

## 2. Results and Discussion

### 2.1. Synthesis of 6-R-Isoxazolo[4,3-b]pyridines **3a**–**j**

**Scheme 2:**
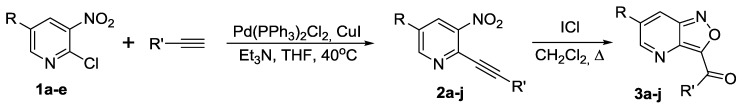
Synthesis of 6-R-isoxazolo[4,3-b]pyridines **3a**–**j**.

6-R-Isoxazolo[4,3-*b*]pyridines **3a**–**j** were synthesized according to a two-steps procedure, previously described in the literature for **3j** [49]. Commercially available 2-chloro-3-nitropyridines **1a**–**e** used as starting compounds were involved in Sonogashira cross-coupling with terminal alkynes to give 2-alkynylpyridines **2a**–**j**. In turn, the cycloisomerization of compounds **2a**–**j** in the presence of catalytic amounts of iodine(I) chloride gave the desired 6-R-3-acylisoxazolo[4,3-*b*]pyridines **3a**–**j** in good yields, Scheme 2, Table 1.

In the case of compounds **2e** and **2f**, ^1^H NMR spectroscopy showed that, along with the expected isoxazolo[4,3-b]pyridines, the formation of minor unidentified products (5–10%) occurred. All attempts to isolate target compounds in their pure forms failed, therefore compounds **3e,f** were used without further purification**.** The structure of compounds **2** and **3** was established on the basis of NMR and HRMS data, and for compounds **2a**, **2c**, **3b** it was additionally confirmed by X-Ray analysis.

### 2.2. X-ray of ***2a***, ***2c***, ***3b***

The crystals of **2a** and **2c** are isostructural with minor differences in the unit cell parameters. All bonds, bond angles and torsion angles are typical as confirmed by a Mogul geometry check [50]. The bond angles at the triple bond C2-C7-C8 (176.11(14) and 176.37(15)°) and C7-C8-C9 (174.20(14) and 171.90(16)° in **2a** and **2c**) deviate from the idealized value of 180° for linear conformation. The angles between the average planes of nitro groups and pyridine group are within the range 7.09(17)–12.44(14)°, despite the presence of the short intramolecular contact O1···C7 (2.6763(17) and 2.6580(18) Å in **2a** and **2c**). Pyridine and phenyl rings are nearly co-planar with interplane angles equal to 5.97(5) and 5.61(5)°. In crystal packing, a head-to-tail arrangement of the molecules is observed with π-stacking interaction between formally acceptor dinitro substituted pyridine ring and phenyl moieties (C···C from ca. 3.3 Å). All other intermolecular contacts are weak and non-directional.

**Figure 3:**
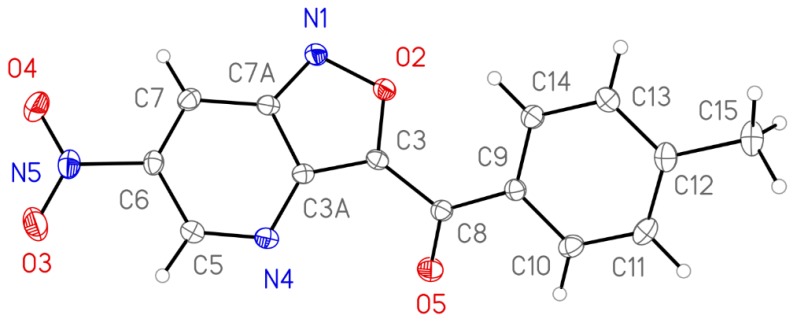
General view of **3b** in crystal. Anisotropic displacement parameters for non-hydrogen atoms are drawn at 50% probability.

The crystal of **3b** is a first example of determined crystal structure containing isoxazolo[4,3-b]pyridine ring, Figure 3. The distribution of bond distances in the heterocycle (N1-O2 1.4109(18), O2-C3 1.3487(19), C3-C3A 1.378(2), N1-C7A 1.330(2), C3A-C7A 1.426(2) Å) is quite similar to the one found in a number of benzo[c]isoxazoles found in Cambridge Structural Database and confirms the canonical structure shown in Figure 3. Due to steric reasons, the heterocycle and tolyl substituents are non-coplanar with O2-C3-C8-C9 torsion angle equal to 48.4(2)°. In crystal molecules, infinite π-stacks (C···C from ca. 3.4 Å) of alternating molecules with head-to-tail arrangement of heterocycles and tolyl fragments are formed.

### 2.3. Nucleophilic Addition to 6-R-Isoxaxolo[4,3-b]pyridines

**Scheme 3:**
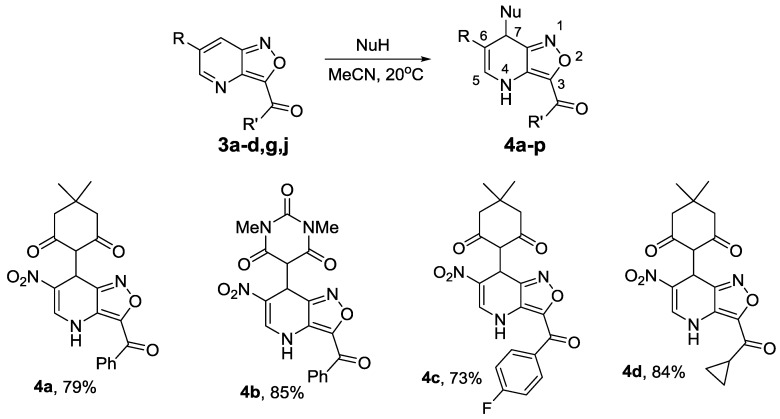

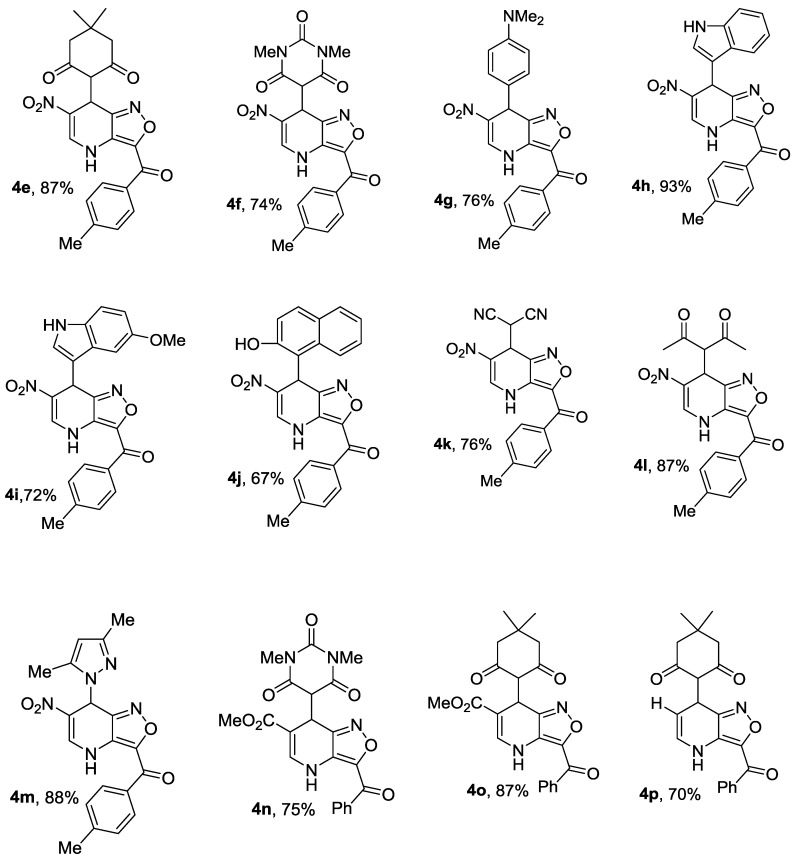
Reactions of 6-R-isoxazolo[4,3-b]pyridines **3** with nucleophiles.

We studied the interaction of isoxazolo[4,3-*b*]pyridines **3** with neutral C-nucleophiles: CH acids and π-excessive (het)arenes. It was found that nitro derivatives **3a**–**d** react with all ranges of used nucleophiles under mild conditions (MeCN, room temperature, base-free), forming 1,4-addition products, **4a**–**m**, Scheme 3. As in the case of **A** [45], β-dicarbonyl compounds react with **3** in enolic form. The reaction rate was similar to that of superelectrophiles **A** and **B** [45,46] (Scheme 1), thus indicating the high electrophilicity of isoxazolo[4,3-*b*]pyridine system. Some of the reactions proceeded almost immediately after mixing the reagents, the others were completed within an hour.

The methoxycarbonyl derivative **3g** forms adducts **4n,o** with 1,3-dicarbonyl compounds somewhat slower: full conversion of starting material required 2–3 h without the addition of a base. 6-Unsubstituted isoxazolopyridine **3j** gave the adduct **4p** with most acidic dimedone after 4 h stirring, Scheme 3. Surprisingly, we were unable to isolate any adducts of isoxazolo[4,3-*b*]pyridines **3h** and **3i** containing electron-withdrawing Cl and CF_3_ groups in position 6. The application of more drastic conditions (MeCN, 80 °C) was not effective; the starting compounds were recovered. The reason for the observed reactivity is not clear, however, we can conclude that the ability of 6-R-isoxazolo[4,3-*b*]pyridines to add neutral C-nucleophiles depends on the substituent 6-R and decreases in the following order

$${\mathrm{NO}}_{2}>{\mathrm{CO}}_{2}\mathrm{Me}>H\gg \mathrm{Cl},{\mathrm{CF}}_{3}$$

The structures of compounds **4** were established on the basis of NMR spectroscopy and HRMS data. In ^1^H NMR spectra of adducts **4,** the signals corresponding to H(7) protons in the range of 5.0–5.5 ppm, as well as downfield signals of NH protons (9.8–10.4 ppm) and H(5) at 8.1 ppm, were observed as doublets with close coupling constants. This confirms the nucleophilic addition at position 7 and is consistent with the results obtained previously for other highly electrophilic azolopyridines [45,46,48].

### 2.4. 6-NO_2_-Isoxazolo[4,3-b]pyridines in Diels-Alder Reactions

The ability to add weak (neutral) nucleophiles is one of the features inherent to superelectrophilic aromatic systems. However, increasing the electrophilicity leads to a decrease in aromaticity. Therefore, (hetero)aromatic superelectrophiles are prone to undergo [4+2]-cycloaddition with dienes or nucleophilic dienophiles [16,26,32,33,34,35].

We found that only 6-NO_2_-isoxazolo[4,3-*b*]pyridines **3a**–**f** are able to give cycloaddition products in Diels-Alder reactions with 2,3-dimethyl-1,3-butadiene while compounds **3g**–**j** with other substituents at position 6 were unreactive. This once again highlights the originality of the nitro group among other electron-withdrawing functional groups and its impact on the electrophilicity of the aromatic systems.

**Scheme 4:**
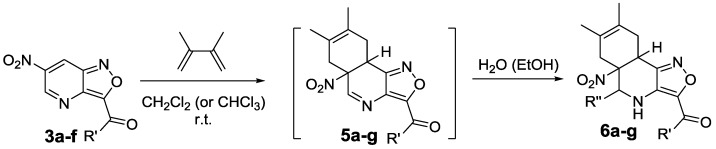
[4+2]-Cycloaddition reactions of 6-NO_2_-isoxazolo[4,3-b]pyridines **3a**–**f.**

Reactions of compounds **3a**–**f** with 2,3-dimethyl-1,3-butadiene were carried out in CH_2_Cl_2_ or CHCl_3_ at room temperature (Scheme 4, Table 2). The C=C–NO_2_ fragment of a pyridine ring acts as a dienophile and the process proceeds in accordance with normal electron demands. However, in all cases, instead of the expected adducts **5**, we isolated compounds **6a**–**g** —products of the further addition of H_2_O to a C=N-double bond.

The intermediate adducts **5a**–**g** are likely to be unstable and exhibit an extremely high tendency to react with nucleophiles (e.g., water) to form compounds **6a**–**g** in good yields. Carrying out the reaction in an inert atmosphere, the additional purification of all solvents did not allow us to isolate compounds **5**. Apparently the formation of products **6** occurs on contact with air moisture at the isolation step. Reaction of **3b** with dimethylbutadiene in chloroform (stabilized with 1.5% EtOH) gave compound **6g**—the product of EtOH addition. In our opinion, this fact indirectly confirms the hypothesis of the high electrophilicity of compounds **5**.

**Figure 4:**
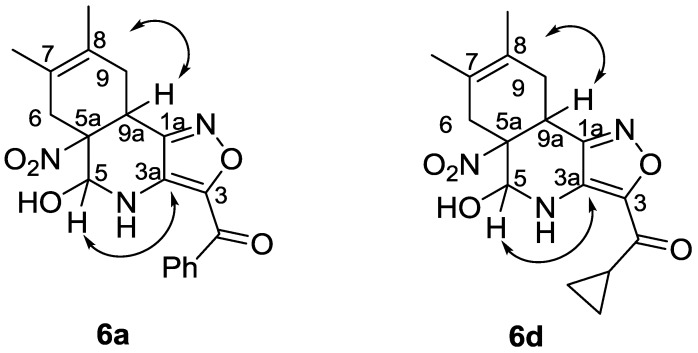
Selected interactions in 2D HMBC spectra of compounds **6a,d**.

The structure of cycloadducts **6** was proved by 2D NMR spectroscopy experiments (COSY, ^1^H-^13^C HMBC, ^1^H-^13^C HSQC). For compounds **6a** and **6d,** the full assignment of hydrogen and carbon atoms in the NMR spectra was made. NMR experiments confirmed the proposed addition of a diene at the C=C(NO_2_) bond, Figure 4.

Cross peaks corresponding to H(5)-C(3a), as well as H(9a)-C(1a) and H(9a)-C(8) interactions were observed in the ^1^H-^13^C HMBC spectra of these compounds. In addition, we observed the coupling of two nonequivalent protons H(9) with the H(9a) proton. Such interactions were described earlier for similar cycloadducts of pyridofuroxan **A** (Figure 2) [34]. These data allowed us to make an unambiguous conclusion about the direction of cycloaddition and hydration.

## 3. Conclusions

A number of new 6-R-isoxazolo[4,3-*b*]pyridines were synthesized, starting from 2-chloro-3-nitropyridines. It was found that, in reactions with neutral C-nucleophiles, the reactivity of 6-R-isoxazolo[4,3-*b*]pyridines depends on the nature of the substituent 6-R. 6-Nitro derivatives were found to add 1,3-dicarbonyl compounds and π-excessive arenes and hetarenes to the pyridine ring under mild conditions to form the corresponding 1,4-adducts. In addition, reactions with 2,3-dimethyl-1,3-butadiene led to [2+4]-adducts on the aromatic bonds C=C(NO_2_) of the pyridine ring. The condensed 3,4-dihydropyridines thus formed easily, adding the molecule of water to C=N double bond to give polyfunctionalized tetrahydropyridine derivatives. All the above properties of 6-nitroisoxazolo[4,3-*b*]pyridines make it possible to classify compounds of this class as superelectrophiles.

## 4. Materials and Methods

### 4.1. General Information

All chemicals were of commercial grade and used directly without purification. Melting points were measured on a Stuart SMP 20 apparatus. ^1^H and ^13^C NMR spectra were recorded on Bruker AC-200 (at 200 and 50 MHz, respectively), Bruker AM-300 (at 300.13 and 75.13 MHz, respectively), Bruker Avance DRX 500 (at 500 and 125 MHz, respectively) or Bruker Avance II 600 spectrometer (at 600 and 150 MHz, respectively) in DMSO-d_6_ or CDCl_3_. IR spectra were recorded on BrukerAlpha spectrometer, and the samples were prepared as KBr pellets. HRMS spectra were recorded on a Bruker micrOTOF II mass spectrometer using ESI. All reactions were monitored by TLC analysis using ALUGRAM SIL G/UV254 plates, which were visualized by UV light. Compounds **1a**–**e** were purchased from commercial suppliers. Compounds **2j** and **3j** were synthesized according to the method [48]. X-ray data collection was performed on a Bruker APEX II diffractometer equipped with Apex II CCD detector and operating with MoKα radiation (λ = 0.71073 Å). Frames were integrated using the Bruker SAINT software package [51] by a narrow-frame algorithm. A semi-empirical absorption correction was applied with the SADABS program [52] using the intensity data of equivalent reflections. The structures were solved with a dual-space approach with SHELXT program [53] and refined by the full-matrix least-squares technique against F^2^_hkl_ in anisotropic approximation with SHELXL [54] software package. All hydrogen atoms were placed in calculated positions and refined in the riding model, with U_iso_(H) constrained to be 1.5U_eq_ and 1.2U_eq_ of the parent methyl and all other carbon atoms. Detailed crystallographic information is given in Table S3 in Supplementary Materials. Crystallographic data have been deposited to the Cambridge Crystallographic Data Centre, CCDC 1983530-1983532 can be retrieved free of charge via https://www.ccdc.cam.ac.uk/structures.

### 4.2. Synthesis of Compounds ***2a***–***i***

A mixture of the appropriate 2-chloropyridine **1** (5 mmol), PdCl_2_(PPh_3_)_2_ (0.17 g; 5 mol-%), and Et_3_N (1.01 g; 10 mmol) was suspended in anhydrous THF (20 mL). The appropriate acetylene (5.5 mmol) was then injected under argon, followed by addition of CuI (0.02 g; 2.5 mol-%). The reaction mixture was stirred under argon at 40 °C temperature until full completion (1–3 h, completion observed by TLC). Solvent was evaporated under the reduced pressure; the crude residue was purified by column chromatography (elution by chloroform).

*3,5-Dinitro-2-(phenylethynyl)pyridine* (**2a**). 72%. Orange powder. M.p. 183–185 °C. ^1^H NMR (300 MHz, CDCl_3_): δ 7.45–7.54 (m, 3H, Ph_._), 7.74–7.76 (d, *J* = 7.2 Hz, 2H, Ph), 9.17 (s, 1H, H4), 9.63 (s, 1H, H6). ^13^C NMR (75 MHz, DMSO-d_6_): δ 85.1, 100.9, 119.9, 128.7, 129.2, 131.3, 132.4, 142.3, 146.2, 148.3. HRMS (ESI) calc. for [C_13_H_8_N_3_O_4_]^+^ [M + H]^+^ 270.0509, found 270.0514.

*3,5-Dinitro-2-(p-tolylethynyl)pyridine* (**2b**). 63%. Orange powder. M.p. 203–205 °C. ^1^H NMR (300 MHz, CDCl_3_): δ 2.46 (s, 3H, Me), 7.30 (d, *J* = 8.0 Hz, 2H, *p*-Tolyl), 7.66 (d, *J* = 8.0 Hz, 2H, p-Tolyl), 9.18 (d, *J* = 2.2 Hz, 1H, H4), 9.63 (d, *J* = 2.2 Hz, 1H, H6). ^13^C NMR (75 MHz, DMSO-d_6_): δ 21.3, 84.9, 101.6, 116.8, 128.6, 129.8, 132.4, 139.8, 141.7, 142.1, 146.0, 148.3. HRMS (ESI) calc. for [C_14_H_10_N_3_O_4_]^+^ [M + H]^+^ 284.0666, found 284.0669.

*3,5-Dinitro-2-((4-fluorophenyl)ethynyl)pyridine* (**2c**). 61%. Orange powder. M.p. 170–172 °C. ^1^H NMR (200 MHz, DMSO-d_6_): δ 7.40 (t, *J* = 8.5 Hz, 2H, 4F-Ph), 7.78 (dd, *J* = 7.9, 5.7 Hz, 2H, 4F-Ph), 9.21 (d, *J* = 2.2 Hz, 1H, H4), 9.65 (d, *J* = 2.2 Hz, 1H, H6). ^13^C NMR (75 MHz, DMSO-d_6_): δ 85.0, 99.8, 128.8, 116.7 (d, *^2^J_C–F_* = 22.5 Hz), 116.6, 116.0, 130.6, 135.2 (d, *^3^J_C–F_* = 9.2 Hz), 139.6, 142.3, 146.1, 147.2, 148.4, 163.5 (d, *^1^J_C–F_* = 251.5 Hz). HRMS (ESI) calc. for [C_13_H_7_FN_3_O_4_]^+^ [M + H]^+^ 288.0415, found 288.0417.

*2-(Cyclopropylethynyl)-3,5-dinitropyridine* (**2d**). 84%. Orange powder. M.p. 128–130 °C. ^1^H NMR (300 MHz, CDCl_3_): δ 1.09–1.18 (m, 4H), 1.68 (dt, *J* = 13.1, 6.6 Hz, 1H), 9.08 (d, *J* = 2.1 Hz, 1H, H4), 9.53 (d, *J* = 2.1 Hz, 1H, H6). ^13^C NMR (75 MHz, DMSO-d_6_): δ 0.4, 9.9, 72.1, 109.5, 128.4, 141.7, 148.0. HRMS (ESI) calc. for [C_10_H_8_N_3_O_4_]^+^ [M + H]^+^ 234.0509, found 234.0517.

*2-(Cyclopentylethynyl)-3,5-dinitropyridine* (**2e**). 32%. Orange oil. ^1^H NMR (300 MHz, CDCl_3_): δ 1.75–1.64 (m, 2H), 1.87 (m, 4H), 2.10 (m, 2H), 3.04 (p, *J* = 7.2 Hz, 1H), 9.08 (d, *J* = 2.1 Hz, 1H, H4), 9.55 (d, *J* = 2.1 Hz, 1H, H6). ^13^C NMR (75 MHz, CDCl_3_): δ 25.3, 31.2, 33.2, 76.5, 112.5, 127.9, 141.3, 142.0, 146.1, 147.7. HRMS (ESI) calc. for [C_12_H_12_N_3_O_4_]^+^ [M + H]^+^ 262.0822, found 262.0816.

*2-(Hept-1-yn-1-yl)-3,5-dinitropyridine* (**2f**). 35%. Orange oil. ^1^H NMR (300 MHz, CDCl_3_): δ 0.78–1.06 (m, 3H), 1.25–1.55 (m, 4H), 1.74 (p, *J* = 7.0 Hz, 2H), 2.63 (t, *J* = 7.1 Hz, 2H), 9.08 (d, *J* = 2.1 Hz, 1H, H4), 9.56 (d, *J* = 2.1 Hz, 1H, H6). ^13^C NMR (75 MHz, CDCl_3_): δ 14.0, 20.4, 22.3, 27.5, 31.2, 108.8, 112.6, 128.0, 128.4, 141.5, 142.2, 147.8 HRMS (ESI) calc. for [C_12_H_14_N_3_O_4_]^+^ [M + H]^+^ 264.0979, found 264.0970.

*Methyl-5-nitro-6-(phenylethynyl)nicotinate* (**2g**) 76%. Orange powder. M.p. 117–119 °C. ^1^H NMR (300 MHz, CDCl_3_): δ 4.05 (s, 3H, Me), 7.42–7.52 (m, 3H, Ph.), 7.71–7.74 (d, *J* = 7.2 Hz, 2H, Ph), 8.95 (d, *J* = 1,3 Hz, 1H, H4), 9.39 (d, *J* = 1.3 Hz, 1H, H6). ^13^C NMR (75 MHz, CDCl_3_): δ 53.2, 85.4, 101.4, 121.0, 125.0, 128.7, 130.7, 133.0, 133.5, 140.5, 146.5, 153.9, 163.5. HRMS (ESI) calc. for [C_15_H_11_N_2_O_4_]^+^ [M + H]^+^ 283.0713, found 283.0721.

*3-Nitro-2-(phenylethynyl)-5-(trifluoromethyl)pyridine* (**2h**) 42% Yellow powder. M.p. 124–126 °C. ^1^H NMR (300 MHz, CDCl_3_): δ 7.47 (m, 3H, Ph), 7.73 (d, *J* = 6.5 Hz, 2H, Ph), 8.65 (s, 1H, H4), 9.09 (s, 1H, H6). ^13^C NMR (126 MHz, CDCl_3_): δ 84.8, 101.5, 120.9, 124.4 (q, *^1^J_C–F_* = 273.0 Hz), 125.8, 128.8, 130.4 (q, *^3^J_C–F_* = 3.7 Hz), 131.0, 133.1, 140.6, 146.1, 149.9 (q, *^3^J_C–F_* = 3.5 Hz). HRMS (ESI) calc. for [C_14_H_8_F_3_N_2_O_2_]^+^ [M + H]^+^ 293.0532, found 293.0542.

*5-Chloro-3-nitro-2-(phenylethynyl)pyridine* (**2i**) 40%. M.p. 103–105 °C. ^1^H NMR (300 MHz, CDCl_3_): δ 7.41–7.48 (m, 3H, Ph_._), 7.69–7.71 (d, *J* = 6.8 Hz, 2H, Ph), 8.41 (d, *J* = 1.6 Hz, 1H, H4), 8.81 (d, *J* = 1.6 Hz, 1H, H6). ^13^C NMR (75 MHz, CDCl_3_): δ 84.5, 99.3, 121.3, 128.7, 130.4, 130.9, 132.3, 132.8, 135.5, 152.7. HRMS (ESI) calc. for [C_13_H_8_ClN_2_O_2_]^+^ [M + H]^+^ 259.0269, found 259.0259.

### 4.3. Synthesis of Compounds ***3a***–***i***

Iodine monochloride (19.5 mg, 0.12 mmol) was added to a solution of the appropriate compound **2** (4 mmol) in dichloromethane (20 mL), and the resulting solution was heated under reflux until full completion (4–8 h.). Solvent was evaporated under the reduced pressure; the crude residue was purified by column chromatography (elution by dichloromethane).

*(6-Nitroisoxazolo[4,3-*b*]pyridin-3-yl)(phenyl)methanone* (**3a**) 85% Yellowish powder. M.p. 135–137 °C. ^1^H NMR (300 MHz, CDCl_3_): δ 7.63 (t, *J* = 7.7 Hz, 2H, Ph_._), 7.77 (t, *J* = 7.4 Hz, 1H, Ph), 8.23 (d, *J* = 7.4 Hz, 2H, Ph) 9.08 (d, *J* = 2.2 Hz, 1H, H5), 9.55 (d, *J* = 2.2 Hz, 1H, H7). ^13^C NMR (75 MHz, CDCl_3_): δ 122.7, 129.7, 131.2, 134.9, 135.7, 135.9, 144.7, 149.6, 150.4, 163.0, 181.1. HRMS (ESI) calc. for [C_13_H_8_N_3_O_4_]^+^ [M + H]^+^ 270.0509, found 270.0508.

*(6-Nitroisoxazolo[4,3-*b*]pyridin-3-yl)(p-tolyl)methanone* (**3b**) 87% Yellowish powder. M.p. 158–160 °C. ^1^H NMR (300 MHz, CDCl_3_): δ 2.52 (s, 3H, Me), 7.43 (d, *J* = 8.1 Hz, 2H, *p*-Tolyl), 8.14 (d, *J* = 8.2 Hz, 2H, *p*-Tolyl), 9.07 (d, *J* = 2.2 Hz, 1H, H5), 9.53 (d, *J* = 2.2 Hz, 1H, H7). ^13^C NMR (151 MHz, CDCl_3_): δ 22.1, 122.1, 129.9, 130.8, 132.8, 134.1, 144.0, 146.5, 148.8, 149.7, 162.8, 180.0. HRMS (ESI) calc. for [C_14_H_10_N_3_O_4_]^+^ [M + H]^+^ 284.0666, found 284.0669.

*(4-Fluorophenyl)(6-nitroisoxazolo[4,3-*b*]pyridin-3-yl)methanone* (**3c**) 72%. Yellowish powder. M.p. 115–117 °C. ^1^H NMR (300 MHz, CDCl_3_): δ 7.33 (d, *J* = 8.5 Hz, 2H, 4F-Ph), 8.32 (dd, *J* = 8.8, 5.3 Hz, 2H, 4F-Ph), 9.09 (d, *J* = 2.2 Hz, 1H, H5), 9.55 (d, *J* = 2.2 Hz, 1H, H7). ^13^C NMR (75 MHz, DMSO-d_6_): δ 116.5 (d, *^2^J_C–F_* = 22.2 Hz), 122.1, 131.8, 133.5 (d, *^3^J_C–F_* = 9.8 Hz), 134.3, 144.2, 149.1, 149.8, 163.8 (d, *^1^J_C–F_* = 225.4 Hz), 168.7, 178.8. HRMS (ESI) calc. for [C_13_H_7_FN_3_O_4_]^+^ [M + H]^+^ 288.0415, found 288.0411.

*Cyclopropyl(6-nitroisoxazolo[4,3-*b*]pyridin-3-yl)methanone* (**3d**) 74%. Yellowish powder. M.p. 118–120 °C. ^1^H NMR (300 MHz, CDCl_3_): δ 1.32-1.37 (m, 2H), 1.53–1.56 (m, 2H), 3.43–3.51 (m, 1H), 9.06 (d, *J* = 2.2 Hz, 1H, H5), 9.54 (d, *J* = 2.2 Hz, 1H, H7). ^13^C NMR (75 MHz, CDCl_3_): δ 14.3, 20.3, 122.3, 133.4, 148.9, 150.0, 161.4, 188.0. HRMS (ESI) calc. for [C_10_H_8_N_3_O_4_]^+^ [M + H]^+^ 234.0509, found 234.0521.

*Methyl-3-benzoylisoxazolo[4,3-*b*]pyridine-6-carboxylate* (**3g**) 65%. Yellowish powder. M.p. 114–116 °C. ^1^H NMR (300 MHz, CDCl_3_): δ 4.07 (s, 3H, Me), 7.61 (t, *J* = 7.6 Hz, 2H, Ph_._), 7.74 (t, *J* = 7.4 Hz, 1H, Ph), 8.25 (d, *J* = 7.5 Hz, 2H, Ph), 8.85 (d, *J* = 1.7 Hz, 1H, H5), 9.36 (d, *J* = 1.7 Hz, 1H, H7). ^13^C NMR (75 MHz, CDCl_3_): δ 53.3, 127.6, 127.8, 128.9, 130.6, 134.6, 135.7, 150.8, 155.0, 161.4, 164.2, 165.8, 180.8. HRMS (ESI) calc. for [C_15_H_11_N_2_O_4_]^+^ [M + H]^+^ 283.0713, found 283.0709.

*Phenyl(6-(trifluoromethyl)isoxazolo[4,3-*b*]pyridin-3-yl)methanone* (**3h**) 73%. Yellowish powder. M.p. 117–119 °C. ^1^H NMR (300 MHz, CDCl_3_): δ 7.62 (t, *J* = 7.6 Hz, 2H, Ph), 7.75 (t, *J* = 7.3 Hz, 1H, Ph), 8.24 (d, *J* = 7.8 Hz, 2H, Ph), 8.52 (s, 1H, H5), 8.98 (s, 1H, H7). ^13^C NMR (75 MHz, CDCl_3_): δ 122.5 (q, *^1^J_C-F_* = 273.6 Hz) 123.5 (q, *^2^J_C-F_* = 3.1 Hz), 126.1, 128.4, 128.7, 129.1, 130.0, 130.6, 134.1, 134.8, 135.6, 149.7, 151.1, 151.1, 161.9, 180.8. HRMS (ESI) calc. for [C_14_H_8_F_3_N_2_O_2_]^+^ [M + H]^+^ 293.0532, found 293.0533.

*(6-Chloroisoxazolo[4,3-*b*]pyridin-3-yl)(phenyl)methanone* (**3i**) 60%. Beige powder. M.p. 120–122 °C. ^1^H NMR (300 MHz, CDCl_3_): δ 7.61 (t, *J* = 7.6 Hz, 2H, Ph_._), 7.74 (t, *J* = 7.4 Hz, 1H, Ph), 8.14 (d, *J* = 1.7 Hz, 1H, H5), 8.23 (d, *J* = 7.5 Hz, 2H, Ph), 8,71 (s, 1H, H7). ^13^C NMR (75 MHz, CDCl_3_): δ 121.8, 129.0, 130.6, 131.9, 133.8, 134.7, 135.7, 151.5, 155.5, 161.1, 180.9. HRMS (ESI) calc. for [C_13_H_8_ClN_2_O_2_]^+^ [M + H]^+^ 259.0269, found 259.0276.

### 4.4. Synthesis of Compounds ***4a***–***p***

A mixture of the appropriate isoxazole **3** (0.5 mmol) and nucleophile (0.5 mmol) was dissolved in anhydrous CH_3_CN (5 mL). The reaction mixture was stirred at r.t. until full completion (1–3 h, by TLC). The solution was diluted with water (25 mL), and the obtained precipitate was filtered off.

*2-(3-Benzoyl-6-nitro-4,7-dihydroisoxazolo[4,3-*b*]pyridin-7-yl)-5,5-dimethylcyclohexane-1,3-dione* (**4a**) 79%. Yellow powder. M.p. 244–246 °C. ^1^H NMR (DMSO-d_6_): δ 0.93 (s, 6H, 2Me), 2.25 (br.s, 4H, 2CH_2_ ), 5.75 (br.s, 1H, H7), 7.64 (t, *J* = 7.5 Hz, 2H, Ph), 7.75 (t, *J* = 7.3 Hz, 1H, Ph), 8.02 (d, *J* = 5.7 Hz, 1H, H5), 8.14 (d, *J* = 7.7 Hz, 2H, Ph), 10.56 (d, *J* = 6.1 Hz, NH). ^13^C NMR (75 MHz, DMSO-d_6_): δ 27.3, 28.0, 31.8, 42.6, 49.8, 50.0, 126.8, 129.1, 129.3, 134.2, 135.2, 137.5, 137.6, 146.3, 157.5, 172.6, 173.1, 180.8. HRMS (ESI) calc. for [C_21_H_20_N_3_O_6_]^+^ [M + H]^+^ 410.1347, found 410.1340.

*5-(3-Benzoyl-6-nitro-4,7-dihydroisoxazolo[4,3-b]pyridin-7-yl)-1,3-dimethylpyrimidine-2,4,6-(1H,3H,5H)-trione* (**4b**) 85%. Yellow powder. M.p. 234–235 °C. ^1^H NMR (300 MHz, DMSO-d_6_): δ 3.03 (s, 3H, Me), 3.17 (s, 3H, Me), 4.65 (s, 1H, CH), 5.52 (s, 1H, H7), 7.64 (t, *J* = 7.2 Hz, 2H, Ph), 7.76 (t, *J* = 7.2 Hz, 1H, Ph), 8.13 (d, *J* = 7.1 Hz, 3H, Ph and H5), 10.93 (d, *J* = 3.7 Hz, 1H, NH). ^13^C NMR (75 MHz, DMSO-d_6_): δ 28.1, 28.3, 33.5, 33.8, 33.9, 52.4, 129.1, 129.4, 134.4, 139.7, 151.1, 166.2, 166.6, 180.6. HRMS (ESI) calc. for [C_19_H_16_N_5_O_7_]^+^ [M + H]^+^ 426.1044, found 426.1037.

*2-(3-(4-Fluorobenzoyl)-6-nitro-4,7-dihydroisoxazolo[4,3-b]pyridin-7-yl)-5,5-dimethylcyclohexane-1,3-dione* (**4c**) 73%. Yellow powder. M.p. 251–253 °C. ^1^H NMR (300 MHz, DMSO-d_6_): δ 0.93 (s, 6H, 2Me), 2.24 (br.s, 4H, 2CH_2_), 5.75 (s, 1H, H7), 7.48 (t, *J* = 8.8 Hz, 2H, 4F-Ph), 8.01 (s, 1H, H5), 8.23 (dd, *J* = 8.7, 5.6 Hz, 2H, 4F-Ph), 10.54 (d, *J* = 1.5 Hz, 1H, NH). ^13^C NMR (75 MHz, DMSO-d_6_): δ 27.4, 27.5, 31.7, 116.2 (d, *^2^J_C–F_* = 22.1 Hz), 126.6, 126.8, 131.8, 132.4 (d, *^3^J_C–F_* = 9.8 Hz), 137.3, 146.1, 157.4, 165.4 (d, *^1^J_C–F_* = 253.5 Hz) 179.1. HRMS (ESI) calc. for [C_21_H_19_FN_3_O_6_]^+^ [M + H]^+^ 428.1252, found 428.1256.

*2-(3-(Cyclopropanecarbonyl)-6-nitro-4,7-dihydroisoxazolo[4,3-b]pyridin-7-yl)-5,5-dimethylcyclohexane-1,3-dione* (**4d**) 84%. Yellow powder. M.p. 258–260 °C. ^1^H NMR (300 MHz, DMSO-d_6_): δ 0.92 (s, 6H, 2Me), 1.16 (s, 4H, 2CH_2_ (c-Pr)), 2.26 (br.s, 4H, 2CH_2_), 2.65 (s, 1H, CH), 5.71 (br.s, 1H, H7), 7.95 (s, 1H, H5), 10.39 (br.s, 1H, NH). ^13^C NMR (75 MHz, DMSO-d_6_): δ 12.0, 12.3, 18.2, 27.6, 31.7, 122.8, 126.1, 137.5, 146.2, 157.65, 189.6. HRMS (ESI) calc. for [C_18_H_20_N_3_O_6_]^+^ [M + H]^+^ 374.1346, found 374.1341.

*5,5-Dimethyl-2-(3-(4-methylbenzoyl)-6-nitro-4,7-dihydroisoxazolo[4,3-b]pyridin-7-yl)-cyclohexane-1,3-dione* (**4e**) 87%. Yellow powder. M.p. 225–227 °C. ^1^H NMR (300 MHz, DMSO-d_6_): δ 0.93 (s, 6H, 2Me), 2.43 (s, 3H, Me, *p*-Tolyl), 5.75 (s, 1H, H7), 7.45 (d, *J* = 7.2 Hz, 2H, *p*-Tolyl), 7.98–8.13 (m, 3H, *p*-Tolyl and H5), 10.49 (d, *J* = 6.7 Hz, 1H, NH). ^13^C NMR (75 MHz, DMSO-d_6_): δ 21.3, 27.2, 31.7, 129.4, 129.6, 132.6, 144.9, 157.3, 180.2. HRMS (ESI) calc. for [C_22_H_22_N_3_O_6_]^+^ [M + H]^+^ 424.1503, found 424.1505.

*1,3-Dimethyl-5-(3-(4-methylbenzoyl)-6-nitro-4,7-dihydroisoxazolo[4,3-b]pyridin-7-yl)-pyrimidine-2,4,6(1H,3H,5H)-trione* (**4f**) 74%. Yellow powder. M.p. 241–243 °C. ^1^H NMR (300 MHz, DMSO-d_6_): δ 2.43 (s, 3H, Me, *p*-Tolyl), 3.03 (s, 3H, Me), 3.17 (s, 3H, Me), 4.64 (s, 1H, CH), 5.51 (s, 1H, H7), 7.45 (d, *J* = 8.1 Hz, 2H, *p*-Tolyl), 8.06 (d, *J* = 8.1 Hz, 2H, *p*-Tolyl), 8.14 (d, *J* = 6.4 Hz, 1H, H5), 10.90 (d, *J* = 6.5 Hz, 1H, NH). ^13^C NMR (75 MHz, DMSO-d_6_): δ 21.3, 28.1, 28.3, 33.8, 52.4, 124.1, 125.4, 129.6, 129.7, 132.3, 139.7, 145.3, 148.1, 151.1, 155.1, 166.2, 166.6, 180.1. HRMS (ESI) calc. for [C_20_H_18_N_5_O_7_]^+^ [M + H]^+^ 440.1201, found 440.1197.

*(7-(4-(Dimethylamino)phenyl)-6-nitro-4,7-dihydroisoxazolo[4,3-b]pyridin-3-yl)(p-tolyl)-methanone* (**4g**) 76%. Orange powder. M.p. 237–239 °C. ^1^H NMR (200 MHz, DMSO-d_6_): δ 2.40 (s, 3H, Me, p-Tolyl), 2.83 (s, 6H, 2Me), 5.62 (s, 1H, H7), 6.64 (d, *J* = 8.7 Hz, 2H, Ar), 7.08 (d, *J* = 8.4 Hz, 2H, *p*-Tolyl), 7.42 (d, *J* = 8.1 Hz, 2H, Ar ), 8.04 (d, *J* = 8.1 Hz, 2H, *p*-Tolyl), 8.21 (s, 1H. H5), 10.68 (s, 1H, NH). ^13^C NMR (75 MHz, DMSO-d_6_): δ 21.3, 37.6, 40.1, 112.5, 124.4, 127.2, 127.7, 129.1, 129.5, 129.6, 132.4, 137.2, 145.0, 148.1, 149.7, 156.8, 180.1. HRMS (ESI) calc. for [C_22_H_21_N_4_O_4_]^+^ [M + H]^+^ 405.1557, found 405.1564.

*(7-(1H-indol-3-yl)-6-nitro-4,7-dihydroisoxazolo[4,3-b]pyridin-3-yl)(p-tolyl)methanone* (**4h**) 93%. Yellow powder. M.p. 221–223 °C. ^1^H NMR (300 MHz, DMSO-d_6_): δ 2.40 (s, 3H, Me), 6.03 (s, 1H, H7), 6.99–7.38 (m, 7H, indole and *p*-Tolyl), 8.05 (s, 2H, *p*-Tolyl), 8.23 (s, 1H, H5), 10.75 (s, 1H, NH), 11.07 (s, 1H, NH). ^13^C NMR (126 MHz, DMSO-d_6_): δ 21.3, 30.4, 66.3, 111.8, 114.2, 118.1, 119.0, 121.3, 123.4, 123.6, 124.7, 125.0, 126.7, 129.5, 129.6, 132.4, 136.4, 136.9, 145.0, 147.9, 156.4, 180.1. HRMS (ESI) calc. for [C_22_H_17_N_4_O_4_]^+^ [M + H]^+^ 401.1243, found 401.1240.

*(7-(5-Methoxy-1H-indol-3-yl)-6-nitro-4,7-dihydroisoxazolo[4,3-b]pyridin-3-yl)(p-tolyl)-methanone* (**4i**) 72%. Yellow powder. M.p. 181–183 °C. ^1^H NMR (300 MHz, DMSO-d_6_): δ 2.41 (s, 3H, Me), 3.71 (s, 3H, OMe), 6.00 (s, 1H, H7), 6.74 (d, *J* = 9.3 Hz, 1H, indole H6), 6.92 (s, 1H, indole H4), 7.24 (m, 2H, indole H2 and H7), 7.43 (d, *J* = 7.5 Hz, 2H, *p*-Tolyl), 8.06 (d, *J* = 7.8 Hz, 2H, *p*-Tolyl), 8.23 (s, 1H, H5), 10.78 (s, 1H, NH), 10.91 (s, 1H, NH). ^13^C NMR (75 MHz, DMSO-d_6_): δ 21.3, 30.4, 55.2, 66.3, 100.2, 111.1, 112.5, 114.1, 124.1, 124.8, 125.4, 126.7, 129.6, 129.7, 131.6, 132.5, 136.9, 145.1, 153.3, 156.5, 180.2. HRMS (ESI) calc. for [C_23_H_19_N_4_O_5_]^+^ [M + H]^+^ 431.1349, found 431.1340.

*(7-(2-Hydroxynaphthalen-1-yl)-6-nitro-4,7-dihydroisoxazolo[4,3-b]pyridin-3-yl)(p-tolyl)-methanone* (**4j**) 67%. Yellow powder. M.p. 204–206 °C. ^1^H NMR (300 MHz, DMSO-d_6_): δ 2.41 (s, 3H, Me), 6.62 (s, 1H, H7), 7.00 (d, *J* = 9.2 Hz, 1H, Ar H3), 7.35 (t, *J* = 7.5 Hz, 1H, Ar H6), 7.44 (d, *J* = 8.0 Hz, 2H, *p*-Tolyl), 7.58 (t, *J* = 7.8 Hz, 1H, Ar H5), 7.72 (d, *J* = 8.8 Hz, 1H, Ar H8), 7.81 (d, *J* = 8.2 Hz, 1H, Ar ), 8.06 (d, *J* = 8.2 Hz, 2H, *p*-Tolyl), 8.17 (s, 1H, H7), 8.53 (d, *J* = 8.9 Hz, 1H, Ar H4), 9.89 (s, NH). ^13^C NMR (75 MHz, DMSO-d_6_): δ 20.2, 29.4, 117.2, 117.7, 121.4, 121.7, 125.6, 125.8, 126.7, 127.0, 128.2, 128.3, 128.4, 128.5, 131.4, 131.8, 136.3, 136.5, 143.9, 145.2, 151.9, 152.1, 156.3, 179.1. HRMS (ESI) calc. for [C_24_H_18_N_3_O_5_]^+^ [M + H]^+^ 428.1240, found 428.1237.

*2-(3-(4-Methylbenzoyl)-6-nitro-4,7-dihydroisoxazolo[4,3-b]pyridin-7-yl)malononitrile* (**4k**) 76%. Yellow powder. M.p. 214–216 °C. ^1^H NMR (300 MHz, DMSO-d_6_): δ 2.45 (s, 3H, Me), 5.59 (s, 1H, CH), 5.67 (s, 1H, H7), 7.49 (d, *J* = 6.1 Hz, 2H, *p*-Tolyl), 8.12 (d, *J* = 6.7 Hz, 2H, *p*-Tolyl), 8.32 (s, 1H. H5), 11.23 (s, 1H, NH). ^13^C NMR (75 MHz, DMSO-d_6_): δ 21.4, 27.9, 35.1, 112.1, 112.2, 120.5, 124.7, 129.7, 132.1, 140.8, 145.5, 149.5, 151.6, 180.0. Found, %: C, 58.39; H, 3.22; N, 20.07; C_17_H_11_N_5_O_4_ Calc., %: C, 58.46; H, 3.17; N, 20.05.

*3-(3-(4-Methylbenzoyl)-6-nitro-4,7-dihydroisoxazolo[4,3-b]pyridin-7-yl)pentane-2,4-dione* (**4l**) 87%. Yellow powder. M.p. 188–190 °C. ^1^H NMR (300 MHz, DMSO-d_6_): δ 2.05 (s, 3H, Me, *p*-Tolyl), 2.42 (d, *J* = 6.4 Hz, 6H, 2Me), 4.90 (s, 1H, CH), 5.30 (s, 1H, H7), 7.45 (d, *J* = 8.1 Hz, 2H, *p*-Tolyl), 8.07 (d, *J* = 7.1 Hz, 3H, *p*-Tolyl and H5), 10.80 (s, 1H, NH). ^13^C NMR (75 MHz, DMSO-d_6_): δ 21.3, 29.6, 31.1, 32.0, 66.9, 124.1, 126.3, 129.6, 132.3, 139.6, 145.2, 154.9, 180.1, 203.2, 204.8. HRMS (ESI) calc. for [C_19_H_17_N_3_O_6_+NH_4_]^+^ [M + NH_4_]^+^ 401.1453, found 401.1456.

*(7-(3,5-Dimethyl-1H-pyrazol-1-yl)-6-nitro-4,7-dihydroisoxazolo[4,3-b]pyridin-3-yl)(p-tolyl)-methanone* (**4m**) 88%. Yellow powder. M.p. 202–204 °C. ^1^H NMR (300 MHz, DMSO-d_6_): δ 1.95 (s, 3H, Me), 2.05 (s, 3H, Me, *p*-Tolyl), 2.42 (s, 3H, Me), 5.82 (s, 1H, H7), 7.14 (s, 1H), 7.44 (d, *J* = 8.1 Hz, 2H, *p*-Tolyl), 8.07 (d, *J* = 8.1 Hz, 2H, *p*-Tolyl), 8.34 (s, 1H) 11.19 (s, 1H, NH). Found, %: C, 58.25; H, 4.22; N, 19.07; C_18_H_15_N_5_O_4_ Calc., %: C, 59.18; H, 4.14; N, 19.17.

*Methyl-3-benzoyl-7-(1,3-dimethyl-2,4,6-trioxohexahydropyrimidin-5-yl)-4,7-dihydroisoxazo-lo[4,3-b]pyridine-6-carboxylate* (**4n**) 75%. Beige powder. M.p. 170–172 °C. ^1^H NMR (300 MHz, DMSO-d_6_): δ 3.03 (s, 3H, Me), 3.13 (s, 3H, Me), 3.63 (s, 3H, CO_2_Me), 4.43 (s, 1H, CH), 5.07 (s, 1H, H7), 7.44 (d, *J* = 5.4 Hz, 1H, H5), 7.63 (t, *J* = 7.3 Hz, 2H, Ph), 7.74 (t, *J* = 7.0 Hz, 1H, Ph), 8.12 (d, *J* = 7.7 Hz, 2H, Ph), 10.2 (d, *J* = 5.3 Hz, 1H, NH). ^13^C NMR (75 MHz, DMSO-d_6_): δ 28.0, 28.1, 33.3, 51.3, 54.0, 99.1, 127.2, 129.0, 129.2, 134.0, 135.2, 138.9, 151.4, 154.9, 166.4, 167.8, 180.5. HRMS (ESI) calc. for [C_21_H_19_N_4_O_7_]^+^ [M + H]^+^ 439.1248, found 439.1242.

*Methyl-3-benzoyl-7-(4,4-dimethyl-2,6-dioxocyclohexyl)-4,7-dihydroisoxazolo[4,3-b]pyridine-6-carboxylate* (**4o**) 87%. Beige powder. M.p. 232–234 °C. ^1^H NMR (300 MHz, DMSO-d_6_): δ 0.93 (s, 6H, 2Me), 2.16 (br.s, 4H, 2CH_2_), 3.53 (s, 3H, CO_2_Me), 5.36 (s, 1H, H7), 7.33 (s, 1H, H5), 7.61 (t, *J* = 7.3 Hz, 2H, Ph), 7.70 (t, *J* = 7.1 Hz, 1H, Ph), 8.11 (d, *J* = 7.6 Hz, 2H, Ph), 9.73 (d, *J* = 5.0 Hz, NH). ^13^C NMR (75 MHz, DMSO-d_6_): δ 26.1, 27.3, 28.3, 31.7, 40.0, 50.7, 102.6, 128.7, 128.8, 129.0, 133.7, 135.6, 136.6, 136.7, 144.5, 157.4, 166.3, 180.4. HRMS (ESI) calc. for [C_23_H_23_N_2_O_6_]^+^ [M + H]^+^ 423.1551, found 423.1545.

*2-(3-Benzoyl-4,7-dihydroisoxazolo[4,3-b]pyridin-7-yl)-5,5-dimethylcyclohexane-1,3-dione* (**4p**) 70%. Beige powder. M.p. 180–182 °C. ^1^H NMR (300 MHz, DMSO-d_6_): δ 0.91 (s, 3H, Me), 0.99 (s, 3H, Me), 2.08–2.40 (m, 4H, 2CH_2_), 4.33 (s, 1H, CH), 5.76 (s, 1H, H7), 7.56-7.67 (m, 4H, Ph and H6), 8.08 (d, *J* = 6.4 Hz, 2H, Ph), 8.44 (s, 1H, H5). ^13^C NMR (75 MHz, DMSO-d_6_): δ 19.4, 24.6, 27.0, 28.5, 31.9, 41.3, 49.7, 77.0, 110.4, 124.2, 126.8, 128.7, 128.8, 128.9, 130.3, 133.2, 134.4, 156.5, 169.5, 180.0, 194.2. HRMS (ESI) calc. for [C_21_H_21_N_2_O_4_]^+^ [M + H]^+^ 365.1496, found 365.1494.

### 4.5. Synthesis of Compounds ***6a***–***g***

2,3-Dimethylbutadiene (0.5 mL, 4.5 mmol) was added to a solution of the appropriate isoxazolopyridine **3** (0.5 mmol) in dichloromethane (or CHCl_3_) (5 mL). The reaction mixture was stirred at r.t. until full completion (normally 4–8 h, TLC control). The solution was diluted with hexane (15 mL), and the obtained precipitate was filtered off.

*(5-Hydroxy-7,8-dimethyl-5a-nitro-4,5,5a,6,9,9a-hexahydroisoxazolo[4,3-c]isoquinolin-3-yl)-(phenyl)methanone* (**6a**) 74%. Beige powder. M.p. 173–175 °C. ^1^H NMR (300 MHz, CDCl_3_): δ 1.56 (s, 3H, Me), 1.67 (s, 3H, Me), 2.45 (d, *J* = 18.1 Hz, 1H), 2.77–3.06 (m, 3H), 3.27 (d, *J* = 3.9 Hz, 1H, OH), 4.35 (d, *J* = 6.8 Hz, 1H), 5.41 (t, *J* = 4.0 Hz, 1H), 6.68 (d, *J* = 3.3 Hz, 1H), 7.55 (t, *J* = 7.6 Hz, 2H, Ph), 7.65 (t, *J* = 7.3 Hz, 1H, Ph), 8.27 (d, *J* = 7.2 Hz, 2H, Ph). ^13^C NMR (75 MHz, CDCl3): δ 18.8, 19.0, 29.3, 30.2, 35.1, 78.6, 89.3, 119.6, 124.2, 128.9, 129.8, 131.5, 133.9, 135.6, 146.7, 154.3, 181.8. HRMS (ESI) calc. for [C_19_H_20_N_3_O_5_] + [M + H]^+^ 370.1397, found 370.1397.

*(5-Hydroxy-7,8-dimethyl-5a-nitro-4,5,5a,6,9,9a-hexahydroisoxazolo[4,3-c]isoquinolin-3-yl)-(p-tolyl)methanone* (**6b**) 80%. Beige powder. M.p. 148–150 °C. ^1^H NMR (300 MHz, CDCl_3_): δ 1.56 (s, 3H, Me), 1.66 (s, 3H, Me), 2.47–3.06 (m, 7H, 2CH_2_+Me(*p*-Tolyl), 3.25 (d, *J* = 3.9 Hz, 1H, OH), 4.34 (d, *J* = 6.9 Hz, 1H), 5.39 (t, *J* = 3.6 Hz, 1H), 6.65 (d, *J* = 4.0 Hz, 1H, NH), 7.35 (d, *J* = 8.2 Hz, 2H, *p*-Tolyl), 8.20 (d, *J* = 8.1 Hz, 2H, *p*-Tolyl). ^13^C NMR (75 MHz, CDCl_3_): δ 18.8, 19.0, 21.9, 29.3, 30.2, 35.1, 78.7, 89.4, 119.6, 124.2, 129.6, 130.0, 131.3, 133.1, 144.9, 146.8, 154.3, 181.4. HRMS (ESI) calc. for [C_20_H_22_N_3_O_5_]^+^ [M + H]^+^ 384.1553, found 384.1548.

*(4-Fluorophenyl)(5-hydroxy-7,8-dimethyl-5a-nitro-4,5,5a,6,9,9a-hexahydroisoxazolo[4,3-c]-isoquinolin-3-yl)methanone* (**6c**) 73%. Beige powder. M.p. 177–179 °C. ^1^H NMR (300 MHz, CDCl_3_): δ 1.56 (s, 3H, Me), 1.67 (s, 3H, Me), 2.47(d, *J* = 17.4Hz, 1H), 2.78–2.91 (m, 3H, 2CH_2_), 3.18 (d, *J* = 3.6 Hz, 1H, OH), 4.34 (d, *J* = 6.9 Hz, 1H), 5.41 (t, *J* = 3.3 Hz, 1H), 6.65 (d, *J* = 3.2 Hz, 1H, NH), 7.23 (t, *J* = 8.6 Hz, 2H, 4F-Ph), 8.34 (dd, *J* = 8.7, 5.4 Hz, 2H, 4F-Ph). ^13^C NMR (75 MHz, CDCl_3_): δ 18.8, 19.0, 29.4, 30.2, 35.1, 78.6, 89.3, 116.2 (d, *^2^J_C_*_–*F*_ = 21.9 Hz), 119.6, 124.2, 131.5, 131.9, 132.0, 132.6 (d, *^3^J_C_*_–*F*_ = 9.5 Hz), 146.5, 154.3, 166.2 (d, *^1^J_C_*_–*F*_ = 256.5 Hz) 180.0. HRMS (ESI) calc. for [C_19_H_19_FN_3_O_5_]^+^ [M + H]^+^ 388.1303, found 388.1316.

*Cyclopropyl-(5-hydroxy-7,8-dimethyl-5a-nitro-4,5,5a,6,9,9a-hexahydroisoxazolo[4,3-c]-isoquinolin-3-yl)methanone* (**6d**) 80%. Beige powder. M.p. 148–150 °C. ^1^H NMR (300 MHz, CDCl_3_): δ 1.12–1.27 (m, 4H, c-Pr), 1.55 (s, 3H, Me), 1.65 (s, 3H, Me), 2.43 (d, *J* = 18.6 Hz, 1H), 2.61-3.00 (m, 4H, 2CH_2_+1H(c-Pr)), 3.67 (s, 1H, OH), 4.30 (d, *J* = 6.9 Hz, 1H), 5.31 (d, *J* = 3.5 Hz, 1H), 6.19 (d, *J* = 3.4 Hz, 1H, NH). ^13^C NMR (75 MHz, CDCl_3_): δ 12.2, 12.4, 18.2, 18.8, 18.9, 29.3, 30.1, 35.1, 78.6, 89.3, 119.5, 124.2, 127.4, 146.8, 154.7, 191.5. HRMS (ESI) calc. for [C_16_H_20_N_3_O_5_+NH_4_]^+^ [M + NH_4_]^+^ 351.1663, found 351.1668.

*Cyclopentyl(5-hydroxy-7,8-dimethyl-5a-nitro-4,5,5a,6,9,9a-hexahydroisoxazolo[4,3-c]-isoquinolin-3-yl)methanone* (**6e**) 51%. Beige powder. M.p. 105–107 °C. 1H NMR (300 MHz, CDCl_3_): δ 1.55 (s, 3H, Me), 1.60–1.73 (m, 7H, Me+2CH_2_), 1.80–2.08 (m, 4H, 2CH_2_), 2.43 (d, *J* = 18.0 Hz, 1H), 2.76-3.04 (m, 3H, 2CH_2_), 3.39 (s, 1H, OH), 3.47-3.59 (m, 1H, CH), 4.29 (d, *J* = 7.1 Hz, 1H), 5.34 (d, *J* = 3.3 Hz, 1H), 6.23 (d, *J* = 3.3 Hz, 1H, NH). ^13^C NMR (75 MHz, CDCl_3_): δ 18.8, 19.0, 26.4, 29.1, 29.2, 29.3, 30.2, 35.1, 47.9, 78.6, 89.3, 119.5, 124.2, 128.3, 146.2, 154.5, 194.5. HRMS (ESI) calc. for [C_18_H_22_N_3_O_5_]^-^ [M − H]^−^ 360.1568, found 360.1565.

*1-(5-Hydroxy-7,8-dimethyl-5a-nitro-4,5,5a,6,9,9a-hexahydroisoxazolo[4,3-c]isoquinolin-3-yl)-hexan-1-one* (**6f**) 35%. Beige powder. M.p. 96–98 °C. ^1^H NMR (300 MHz, CDCl_3_): δ 0.92 (t, *J* = 6.4 Hz, 3H, Me(n-C_5_H_11_)), 1.33–1.42 (m, 4H, 2CH_2,_ n-C_5_H_11_), 1.55 (s, 3H, Me), 1.73 (m, 5H, Me+CH_2_(n-C_5_H_11_)), 2.42 (d, *J* = 18.2 Hz, 1H), 2.55–2.99 (m, 5H, 2CH_2_+CH_2_(n-C_5_H_11_)), 3.52 (br.s, 1H, OH), 4.29 (d, *J* = 6.4 Hz, 1H), 5.34 (d, *J* = 3.6 Hz, 1H), 6.24 (d, *J* = 3.3 Hz, 1H, NH). ^13^C NMR (75 MHz, CDCl_3_): δ 14.0, 18.7, 19.0, 22.5, 23.5, 29.4, 30.2, 31.6, 35.1, 39.4, 78.7, 89.4, 119.6, 124.2, 128.1, 146.4, 154.6, 192.0. HRMS (ESI) calc. for [C_18_H_26_N_3_O_5_]^+^ [M + H]^+^ 364.1866, found 364.1875.

*(5-Ethoxy-7,8-dimethyl-5a-nitro-4,5,5a,6,9,9a-hexahydroisoxazolo[4,3-c]isoquinolin-3-yl)-(p-tolyl)methanone* (**6g**) 42%. Yellowish powder. M.p. 134–136 °C. ^1^H NMR (300 MHz, CDCl_3_): δ 1.13 (t, *J* = 7.0 Hz, 3H, Et), 1.54 (s, 3H, Me), 1.65 (s, 3H, Me), 2.47–3.07 (m, 7H, 2CH_2_+Me(*p*-Tolyl), 3.50–3.32 (m, 1H, CH_2,_ Et), 3.84–3.67 (m, 1H, CH_2,_ Et), 4.30 (d, *J* = 6.8 Hz, 1H), 4.95 (d, *J* = 4.0 Hz, 1H), 6.78 (d, *J* = 3.4 Hz, 1H, NH), 7.35 (d, *J* = 8.1 Hz, 2H, *p*-Tolyl), 8.22 (d, *J* = 8.2 Hz, 2H, *p*-Tolyl). ^13^C NMR (75 MHz, CDCl_3_): δ 14.7, 18.8, 19.0, 22.0, 29.7, 30.2, 35.2, 63.9, 84.5, 88.9, 119.3, 124.2, 129.6, 130.0, 131.4, 133.2, 144.8, 146.9, 154.6, 181.2. HRMS (ESI) calc. for [C_22_H_25_N_3_O_5_]^+^ [M + H]^+^ 412.1866, found 412.1859.

## Figures and Tables

**Table 1:** Isolated yields of compounds **2a**–**j** and **3 a**–**j**.

Compound 1	R	R′	Product 2, Yield (%)	Product 3, Yield (%)
**1a**	NO_2_	Ph	**2a**, 72	**3a**, 85
**1a**	NO_2_	4-Me-C_6_H_4_	**2b**, 63	**3b**, 87
**1a**	NO_2_	4-F-C_6_H_4_	**2c**, 61	**3c**, 72
**1a**	NO_2_	*c*-C_3_H_5_	**2d**, 84	**3d**, 74
**1a**	NO_2_	*c*-C_5_H_9_	**2e**, 82	**3e**, 71 *
**1a**	NO_2_	*n*-C_5_H_11_	**2f**, 35	**3f**, 80 *
**1b**	CO_2_Me	Ph	**2g**, 76	**3g**, 65
**1c**	CF_3_	Ph	**2h**, 42	**3h**, 73
**1d**	Cl	Ph	**2i**, 40	**3i**, 60
**1e**	H	Ph	**2j**, 82	**3j**, 80

* The yield is shown for the crude product.

**Table 2:** Isolated yields of compounds **6a**–**g.**

Compound 3	R′	R″	Product 6, Yield (%)
**3a**	Ph	**OH**	**6a**, 74
**3b**	4-Me-C_6_H_4_	**OH**	**6b**, 80
**3c**	4-F-C_6_H_4_	**OH**	**6c**, 73
**3d**	*c*-C_3_H_5_	**OH**	**6d**, 80
**3e**	*c*-C_5_H_9_	**OH**	**6e**, 51
**3f**	*n*-C_5_H_11_	**OH**	**6f**, 35
**3b**	4-Me-C_6_H_4_	**OEt**	**6g**, 42

## Supplementary Materials

NMR spectra, HRMS and X-ray analysis data.
