# Supplementary material for: Synthesis and Facile Dearomatization of Highly Electrophilic Nitroisoxazolo[4,3-b]pyridines
Source: Molecules. 2020 May 8;25(9):2194. doi: 10.3390/molecules25092194 (PMC7248838; doi:10.3390/molecules25092194)
Supplement: Supplementary file 1 [file molecules-25-02194-s001.pdf]

Table of contents

NMR and HRMS spectra for **2a** S3-S5

NMR and HRMS spectra for **2b** S6-S8

NMR and HRMS spectra for **2c** S9-S11

NMR and HRMS spectra for **2d** S12-S14

NMR and HRMS spectra for **2e** S15-S17

NMR and HRMS spectra for **2f** S18-S20

NMR and HRMS spectra for **2g** S21-S23

NMR and HRMS spectra for **2h** S24-S26

NMR and HRMS spectra for **2i** S27-S29

NMR and HRMS spectra for **3a** S30-S32

NMR and HRMS spectra for **3b** S33-S35

NMR and HRMS spectra for **3c** S36-S38

NMR and HRMS spectra for **3d** S39-S41

NMR and HRMS spectra for **3g** S42-S44

NMR and HRMS spectra for **3h** S45-S47

NMR and HRMS spectra for **3i** S48-S50

NMR and HRMS spectra for **4a** S51-S53

NMR and HRMS spectra for **4b** S54-S56

NMR and HRMS spectra for **4c** S57-S59

NMR and HRMS spectra for **4d** S60-S62

NMR and HRMS spectra for **4e** S63-S65

NMR and HRMS spectra for **4f** S66-S68

NMR and HRMS spectra for **4g** S69-S71

NMR and HRMS spectra for **4h** S72-S74

NMR and HRMS spectra for **4i** S75-S77

NMR and HRMS spectra for **4j** S78-S80

NMR and HRMS spectra for **4k** S81-S83

NMR and HRMS spectra for **4l** S84-S86

NMR spectrum for **4m** S87

NMR and HRMS spectra for **4n** S88-S90

NMR and HRMS spectra for **4o** S91-S93

NMR and HRMS spectra for **4p** S94-S96

NMR and HRMS spectra for **6a** S97-S99

NMR and HRMS spectra for **6b** S100-S102

NMR and HRMS spectra for **6c** S103-S105
NMR and HRMS spectra for **6d** S106-S108
NMR and HRMS spectra for **6e** S109-S111
NMR and HRMS spectra for **6f** S112-S114
NMR and HRMS spectra for **6g** S115-S117
X-Ray data for **2a** S118-S120
X-Ray data for **2c** S121-S123
X-Ray data for **3b** S124-S126
Crystallographic data for **2a, 2c** and **3b** S127

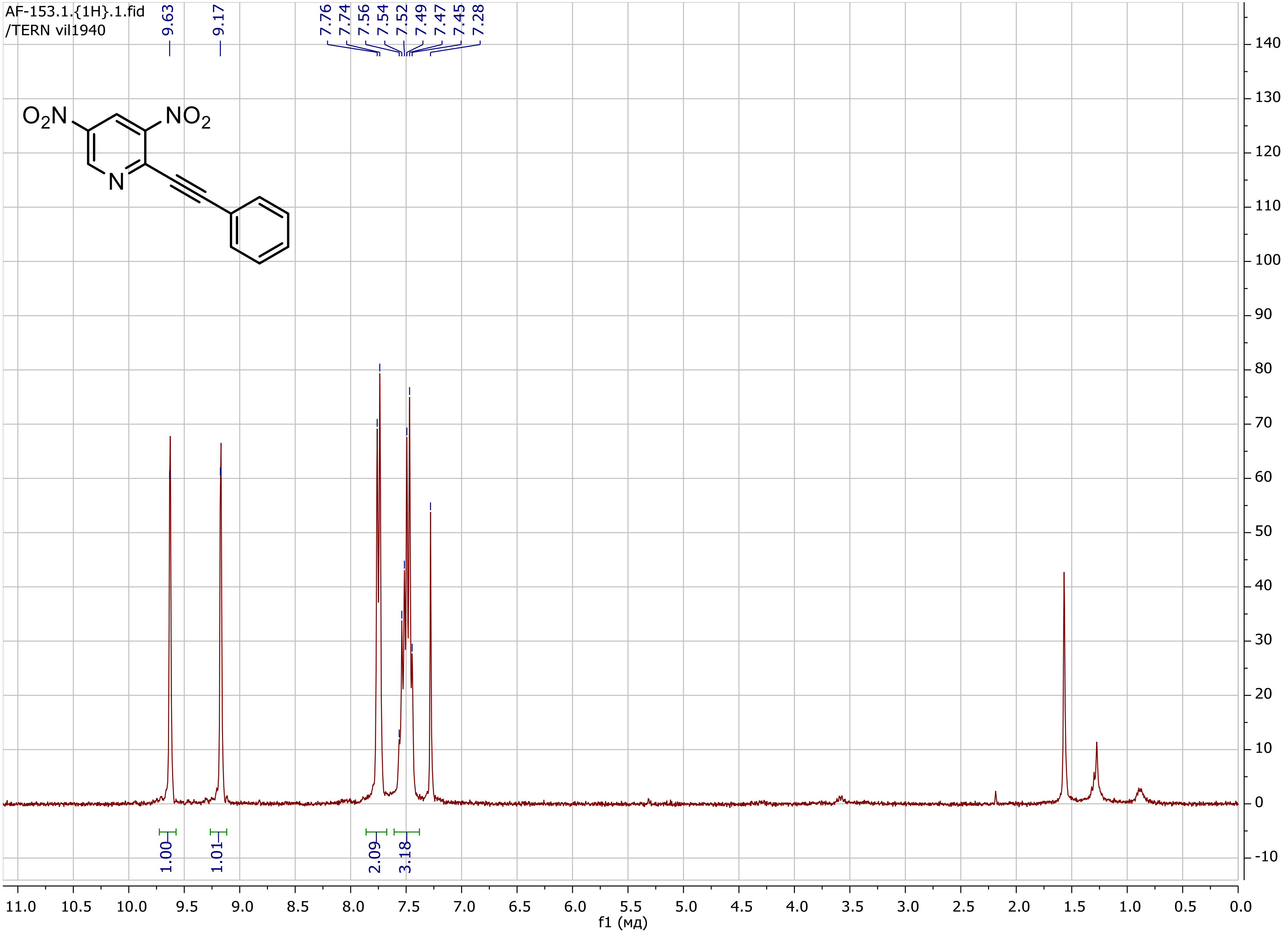
AF-153.1.{1H}.1.fid
/TERN vil1940
9.63
9.17
7.76
7.74
7.56
7.54
7.52
7.49
7.47
7.45
7.28
O2N
NO2
N
1.00
1.01
2.09
3.18
11.0 10.5 10.0 9.5 9.0 8.5 8.0 7.5 7.0 6.5 6.0 5.5 5.0 4.5 4.0 3.5 3.0 2.5 2.0 1.5 1.0 0.5 0.0
f1 (мд)
140 130 120 110 100 90 80 70 60 50 40 30 20 10 0 -10

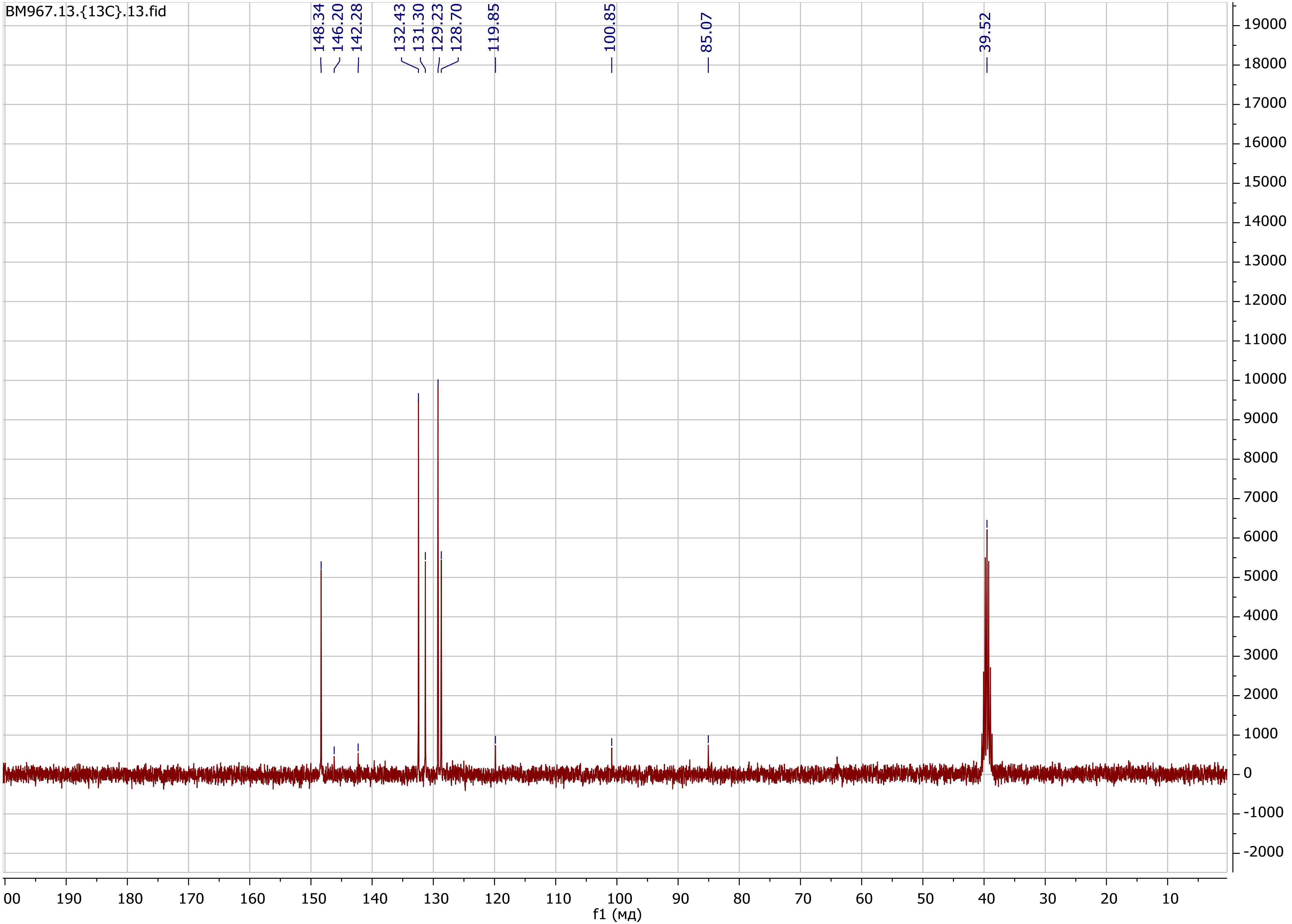
BM967.13.{13C}.13.fid
148.34
146.20
142.28
132.43
131.30
129.23
128.70
119.85
100.85
85.07
39.52
f1 (мд)

Display Report

Analysis Info

Analysis Name	D:\Data\Kolotyrkina\2014\Bastrakov\0305012.d	Acquisition Date	05.03.2014 14:47:17
Method	tune_low.m	Operator	BDAL@DE
Sample Name	/LPIK BM967	Instrument / Ser#	micrOTOF 10248
Comment	C13H7N3O4 mw 269 calibrant added		

Acquisition Parameter

Source Type	ESI	Ion Polarity	Positive	Set Nebulizer	0.4 Bar
Focus	Not active			Set Dry Heater	180 °C
Scan Begin	50 m/z	Set Capillary	4500 V	Set Dry Gas	4.0 l/min
Scan End	3000 m/z	Set End Plate Offset	-500 V	Set Divert Valve	Waste

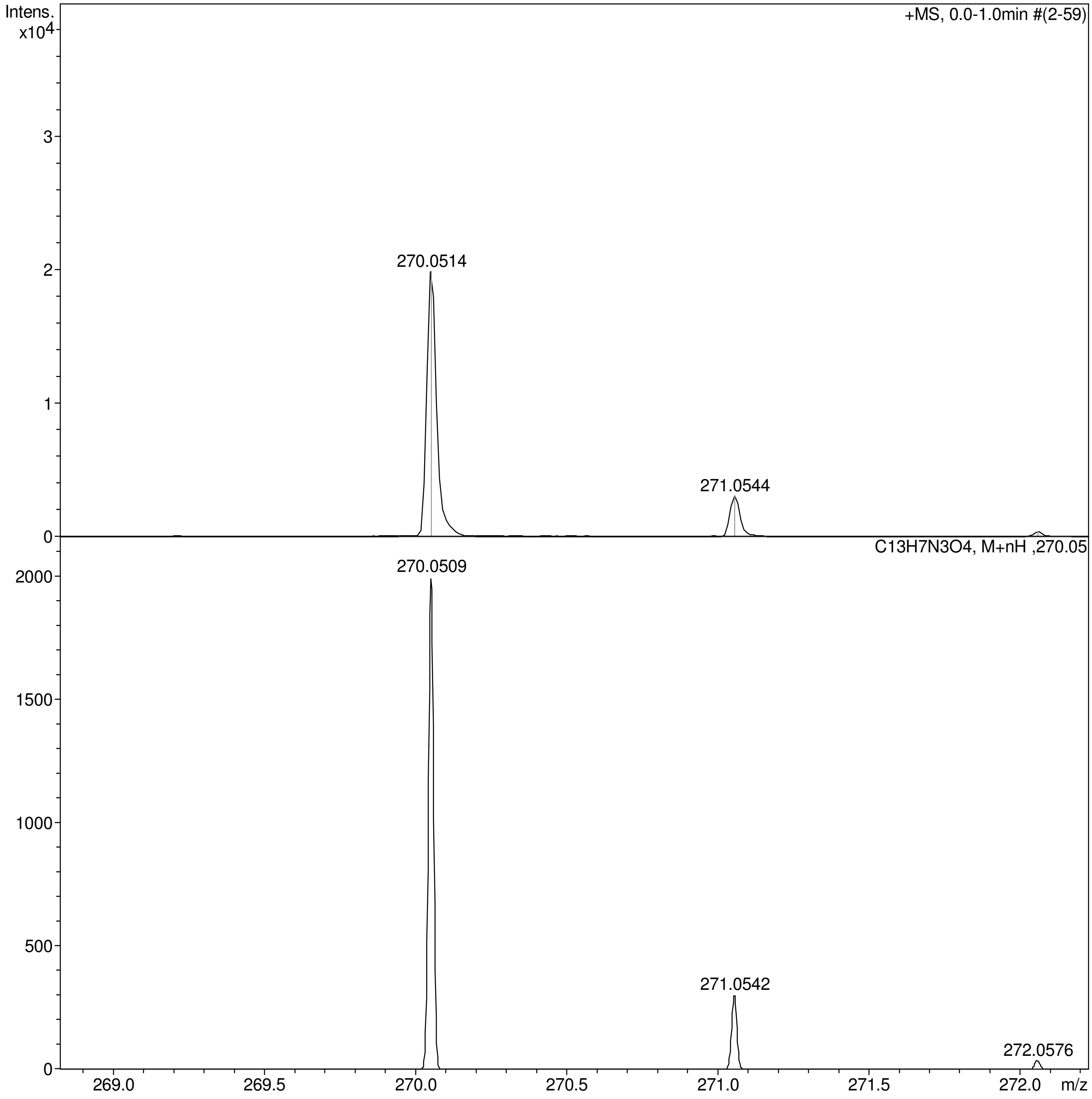

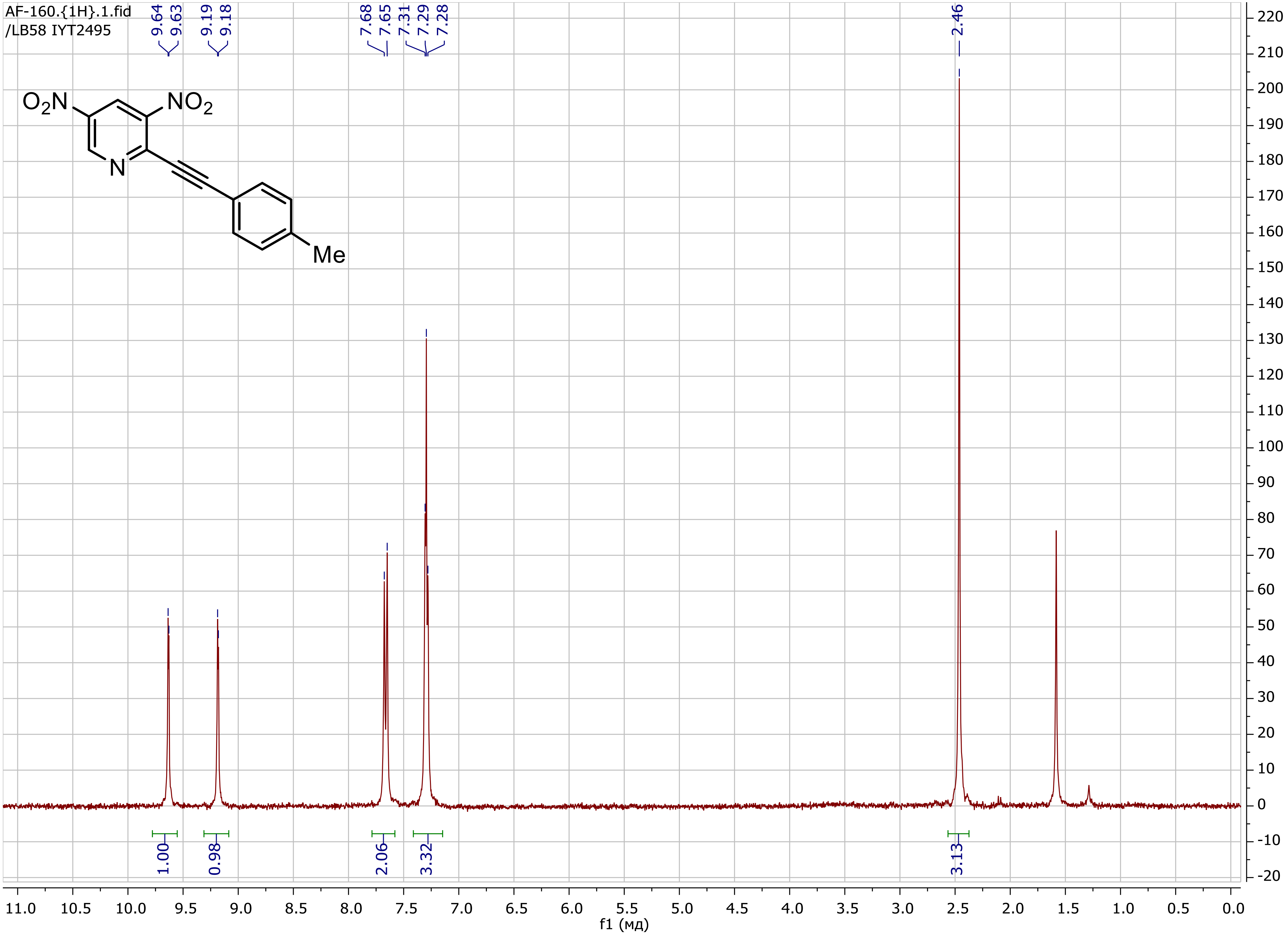
AF-160.{1H}.1.fid
/LB58 IYT2495
9.64
9.63
9.19
9.18
7.68
7.65
7.31
7.29
7.28
2.46
O2N
NO2
N
Me
1.00
0.98
2.06
3.32
3.13
f1 (мд)

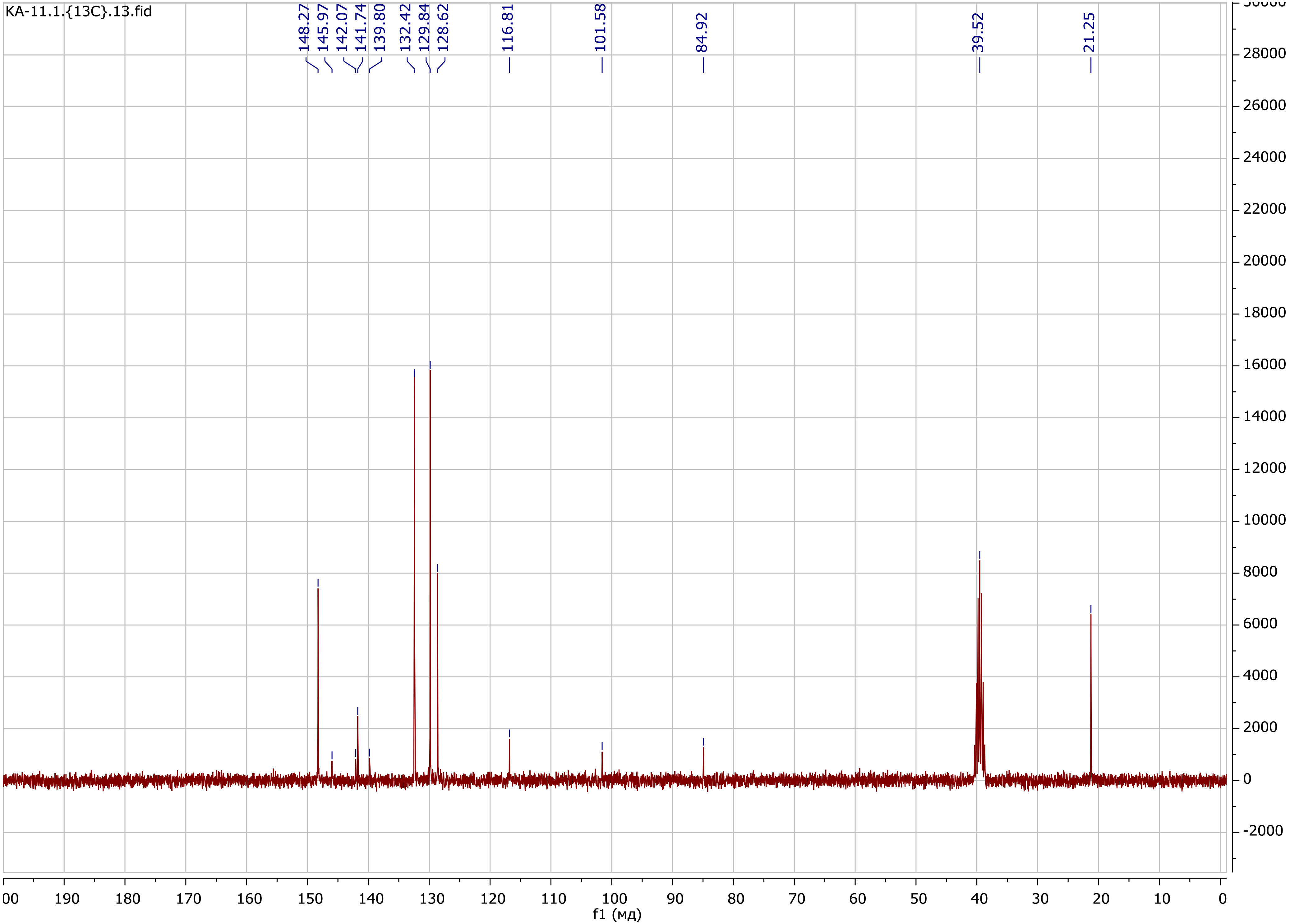
KA-11.1.{13C}.13.fid
148.27
145.97
142.07
141.74
139.80
132.42
129.84
128.62
116.81
101.58
84.92
39.52
21.25
f1 (мд)

Display Report

Analysis Info

Analysis Name	D:\Data\Kolotyrkina\2014\Bastrakov\0416005.d	Acquisition Date	16.04.2014 13:30:51
Method	tune_low.m	Operator	BDAL@DE
Sample Name	/LPIK KA-11	Instrument / Ser#	micrOTOF 10248
Comment	C14H9N3O4 mw 283 calibrant added		

Acquisition Parameter

Source Type	ESI	Ion Polarity	Positive	Set Nebulizer	0.4 Bar
Focus	Not active			Set Dry Heater	180 °C
Scan Begin	50 m/z	Set Capillary	4500 V	Set Dry Gas	4.0 l/min
Scan End	3000 m/z	Set End Plate Offset	-500 V	Set Divert Valve	Waste

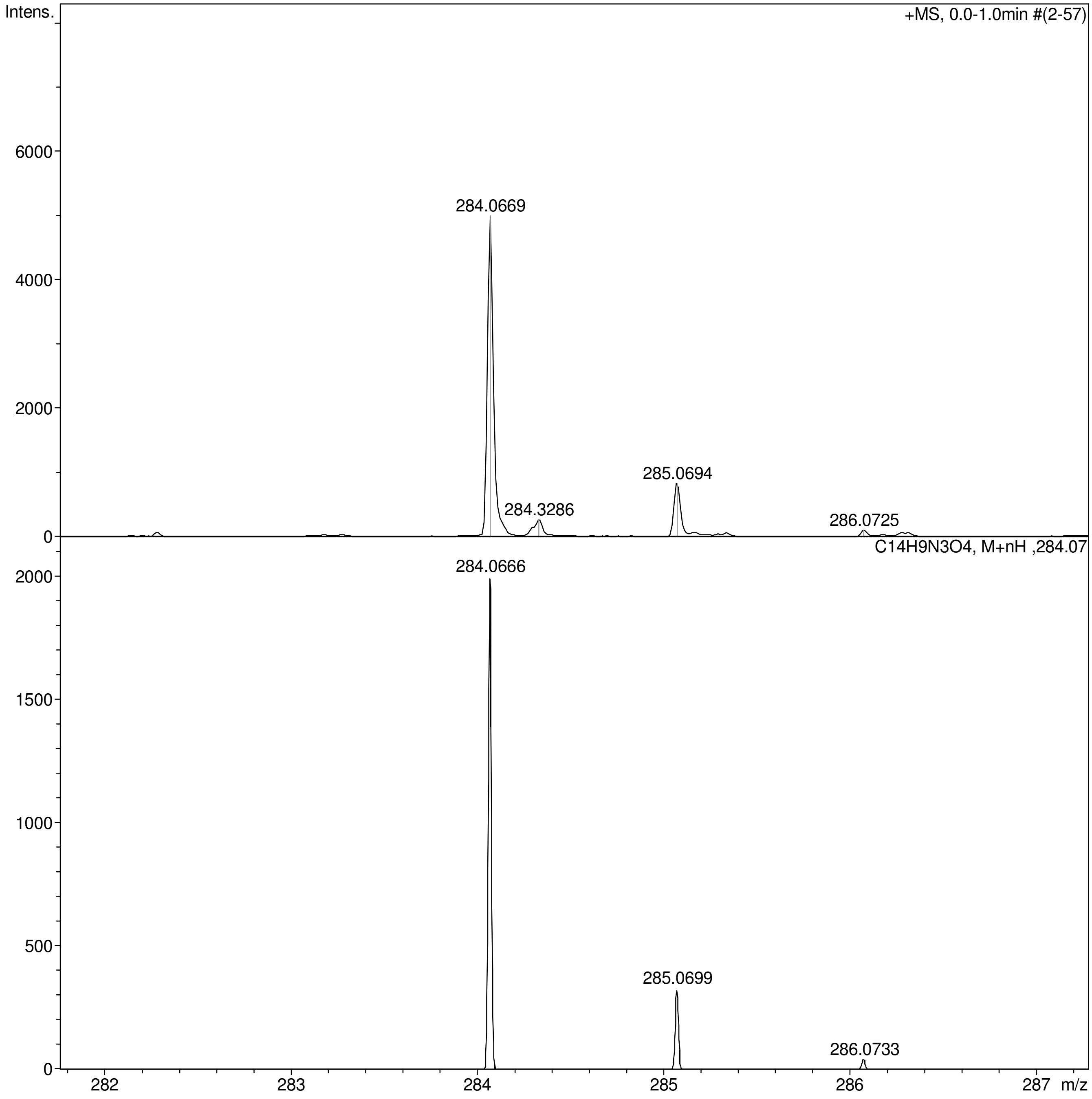

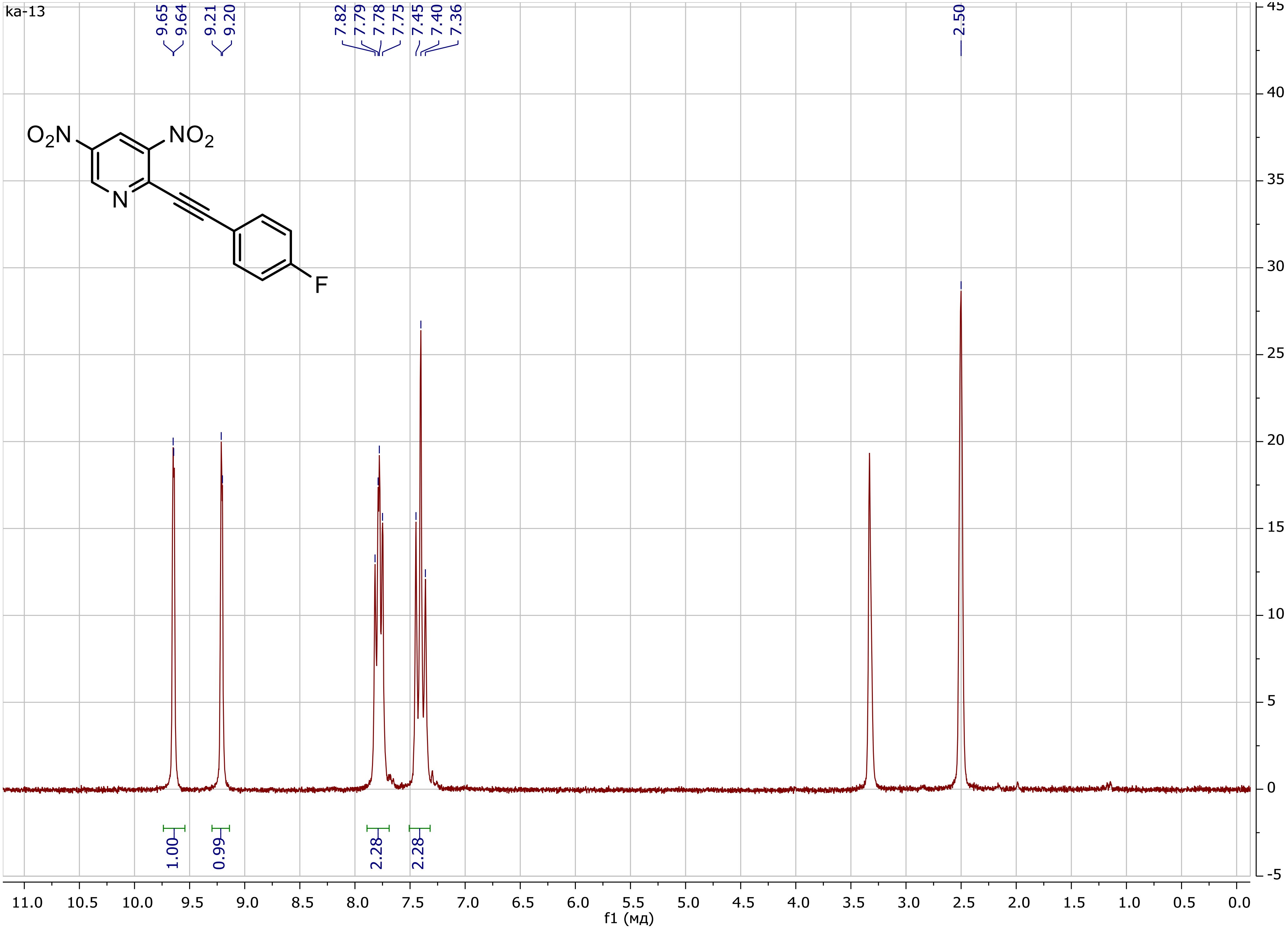
ka-13
9.65
9.64
9.21
9.20
7.82
7.79
7.78
7.75
7.45
7.40
7.36
2.50
O_2N
NO_2
N
F
1.00
0.99
2.28
2.28
f1 (мд)

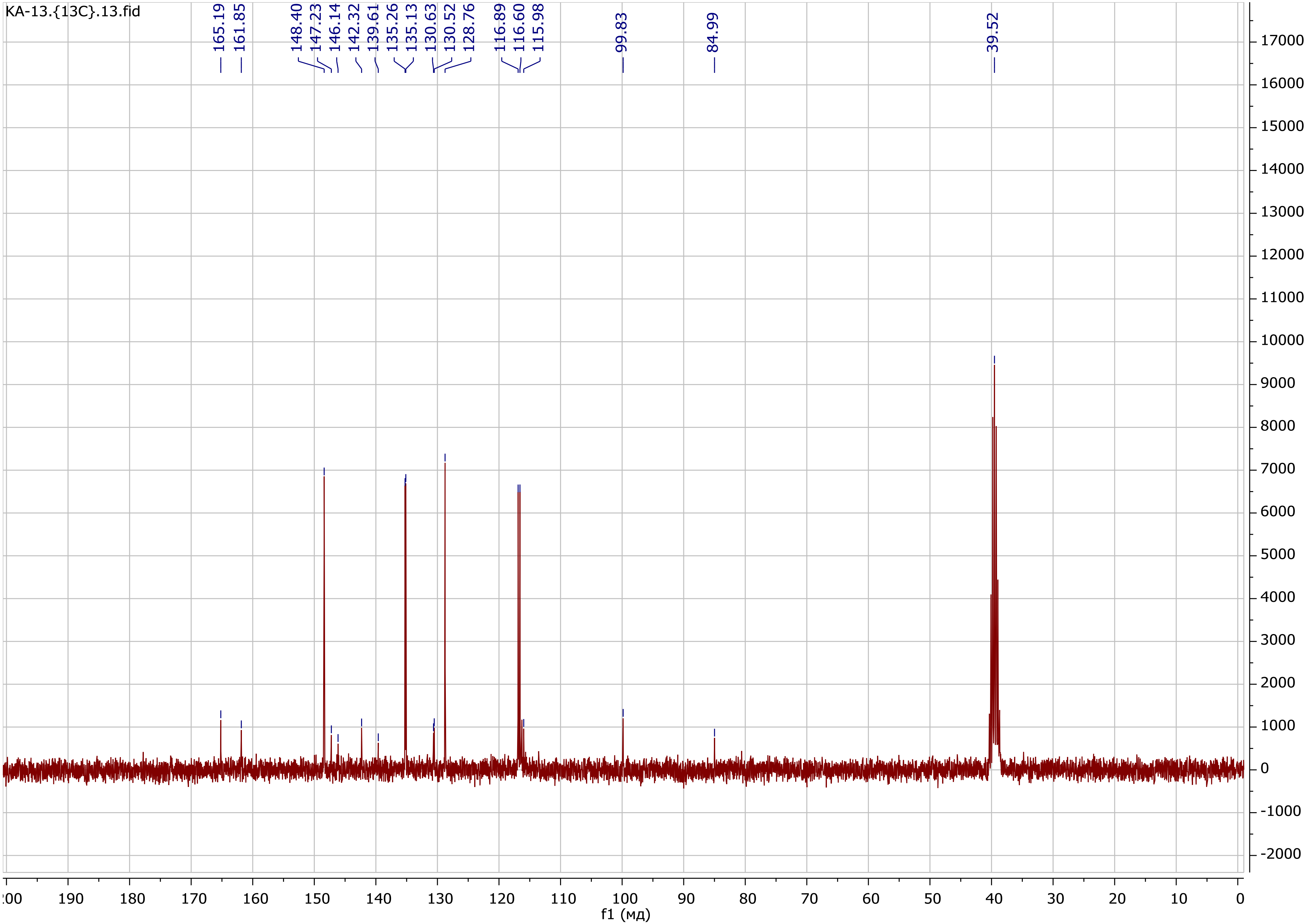

KA-13.{13C}.13.fid
165.19
161.85
148.40
147.23
146.14
142.32
139.61
135.26
135.13
130.63
130.52
128.76
116.89
116.60
115.98
99.83
84.99
39.52
f1 (мд)

Display Report

Analysis Info

Analysis Name	D:\Data\Chizhov\Shevelev\Bastrakov\ka-13_&clblow.d	Acquisition Date	28.04.2014 16:58:30
Method	tune_low.m	Operator	BDAL@DE
Sample Name	/LPIK KA-13	Instrument / Ser#	micrOTOF 10248
Comment	CH3CN 100 %, dil. 2, calibrant added		

Acquisition Parameter

Source Type	ESI	Ion Polarity	Positive	Set Nebulizer	0.4 Bar
Focus	Not active			Set Dry Heater	180 °C
Scan Begin	50 m/z	Set Capillary	4500 V	Set Dry Gas	4.0 l/min
Scan End	3000 m/z	Set End Plate Offset	-500 V	Set Divert Valve	Waste

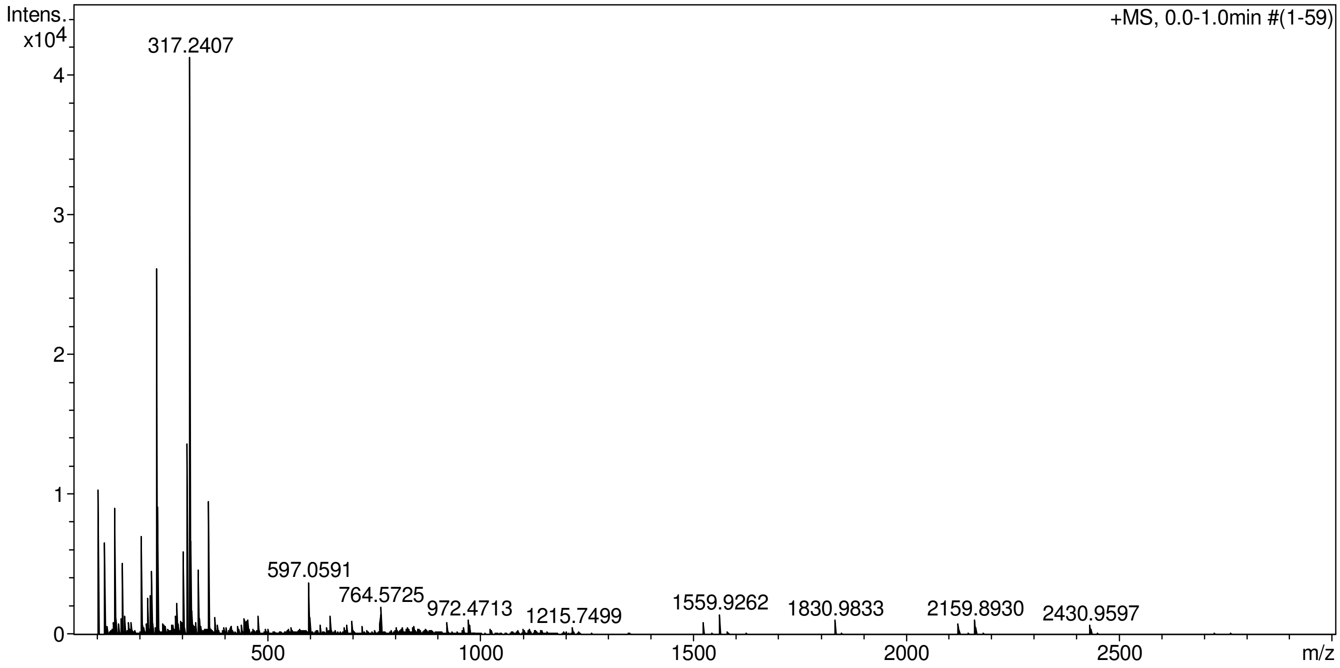

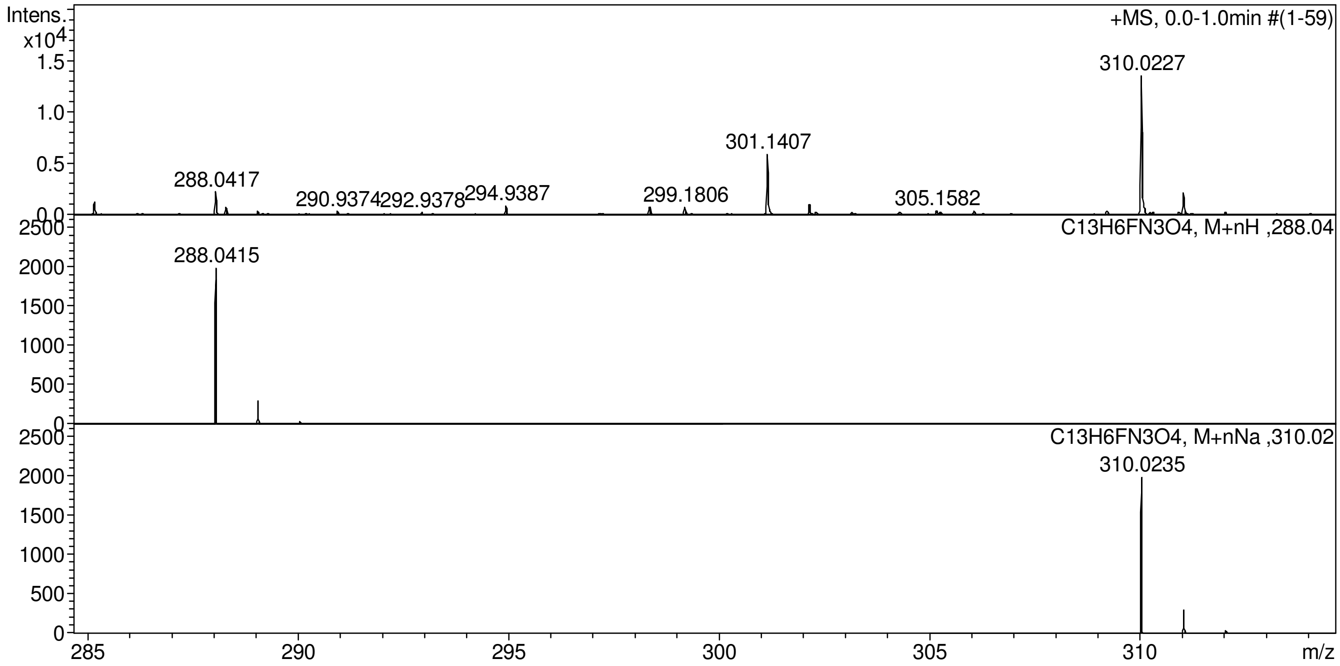

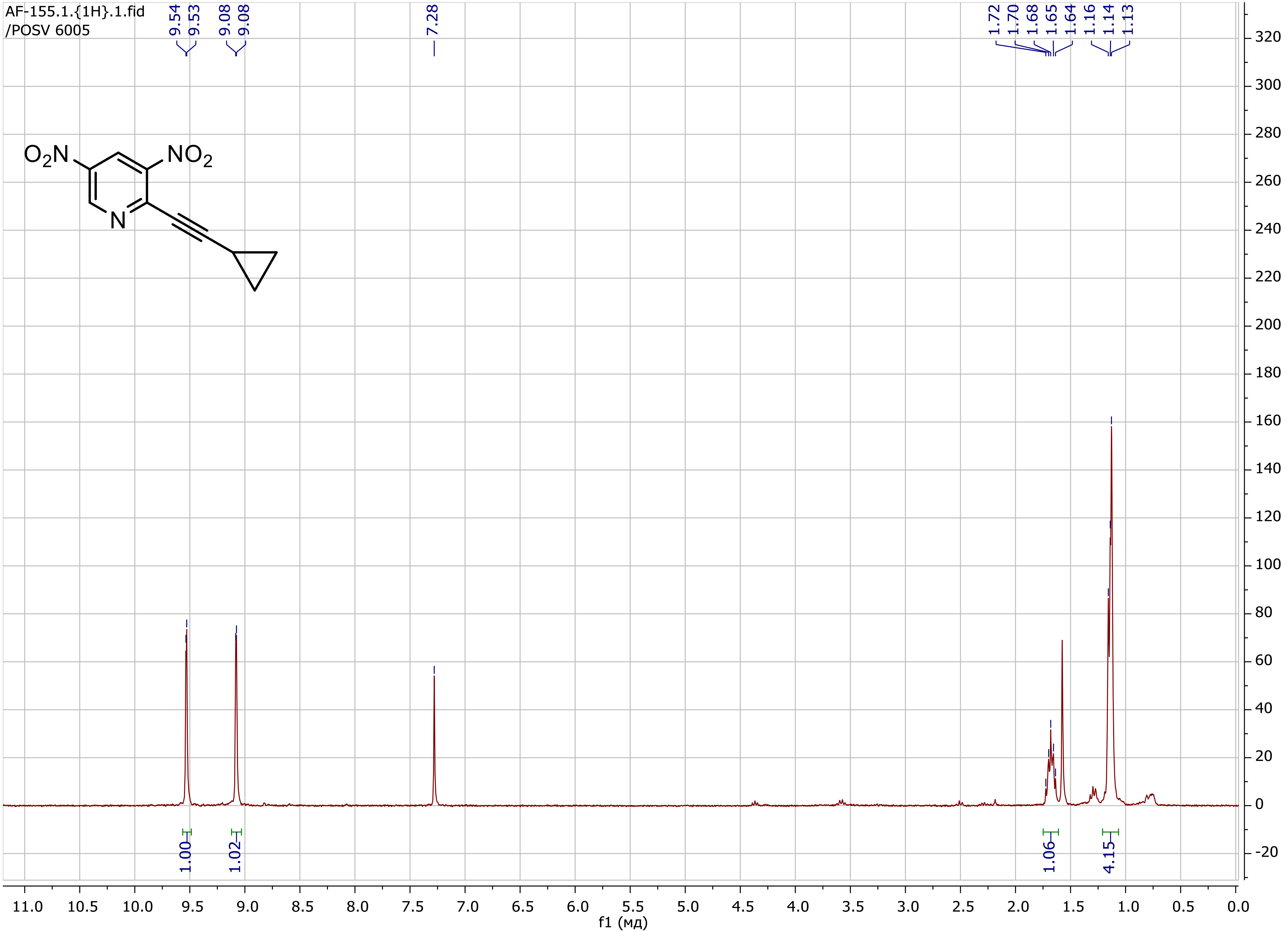
AF-155.1.{1H}.1.fid
/POSV 6005
9.54
9.53
9.08
9.08
7.28
1.72
1.70
1.68
1.65
1.64
1.16
1.14
1.13
O2N
NO2
N
1.00
1.02
1.06
4.15
f1 (мд)

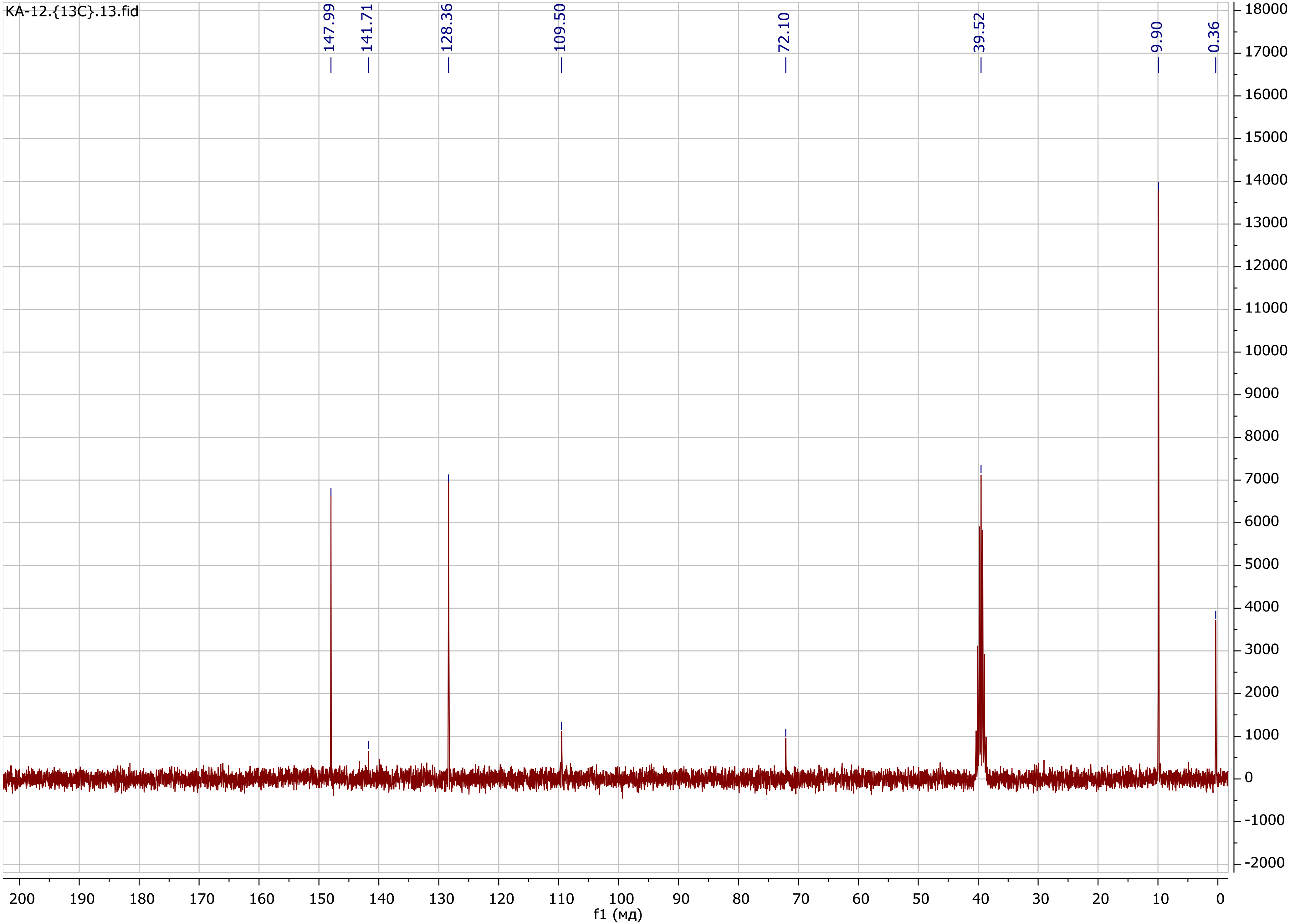
KA-12.{13C}.13.fid
147.99
141.71
128.36
109.50
72.10
39.52
9.90
0.36
f1 (мд)

Display Report

Analysis Info

Analysis Name	D:\Data\Kolotyrkina\2014\Bastrakov\0415017.d	Acquisition Date	15.04.2014 16:15:34
Method	tune_low.m	Operator	BDAL@DE
Sample Name	/LPIK KA-12	Instrument / Ser#	micrOTOF 10248
Comment	C10H7N3O4 mw 233 in CH3CN calibrant added		

Acquisition Parameter

Source Type	ESI	Ion Polarity	Positive	Set Nebulizer	0.4 Bar
Focus	Not active			Set Dry Heater	180 °C
Scan Begin	50 m/z	Set Capillary	4500 V	Set Dry Gas	4.0 l/min
Scan End	3000 m/z	Set End Plate Offset	-500 V	Set Divert Valve	Waste

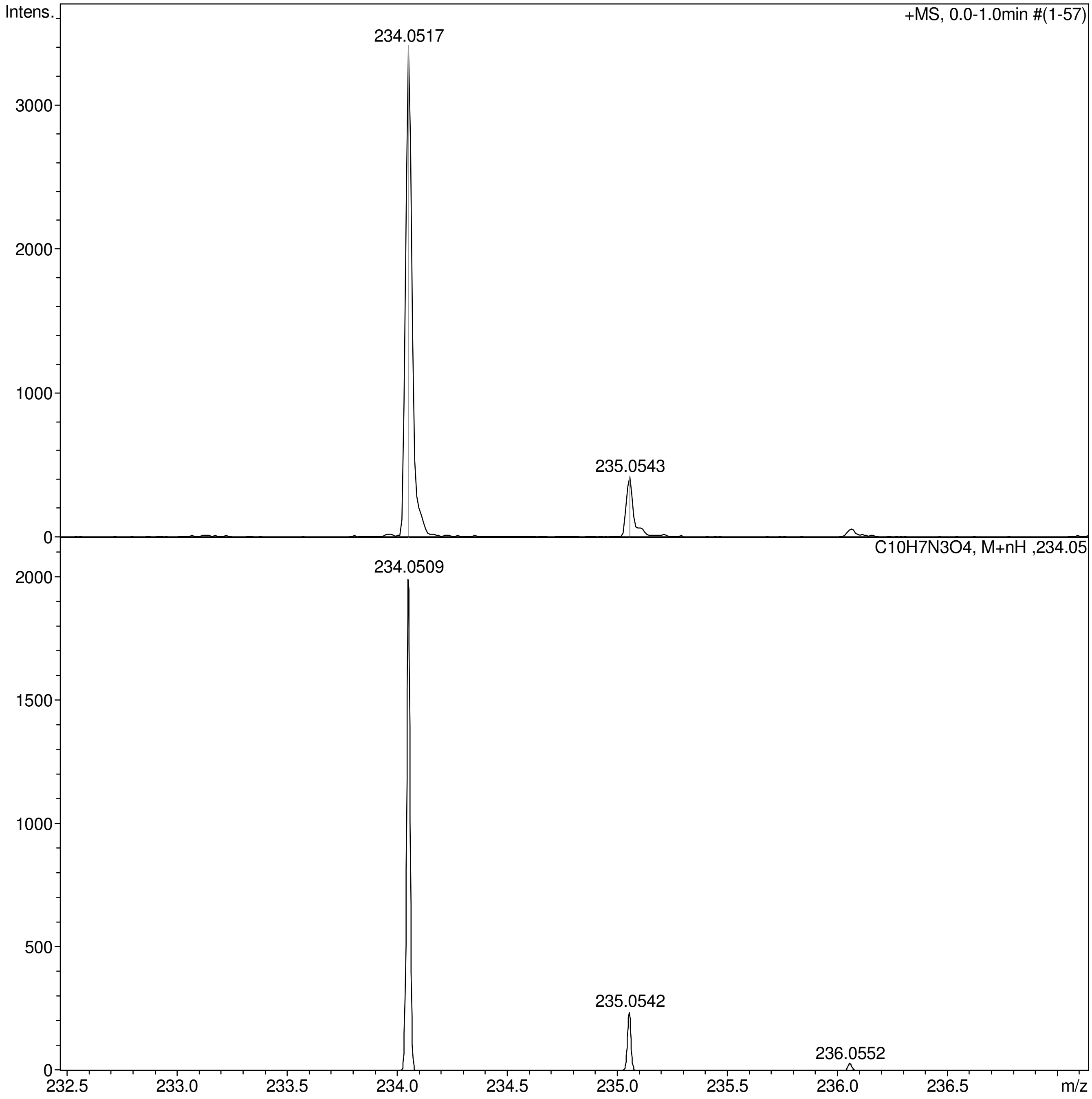

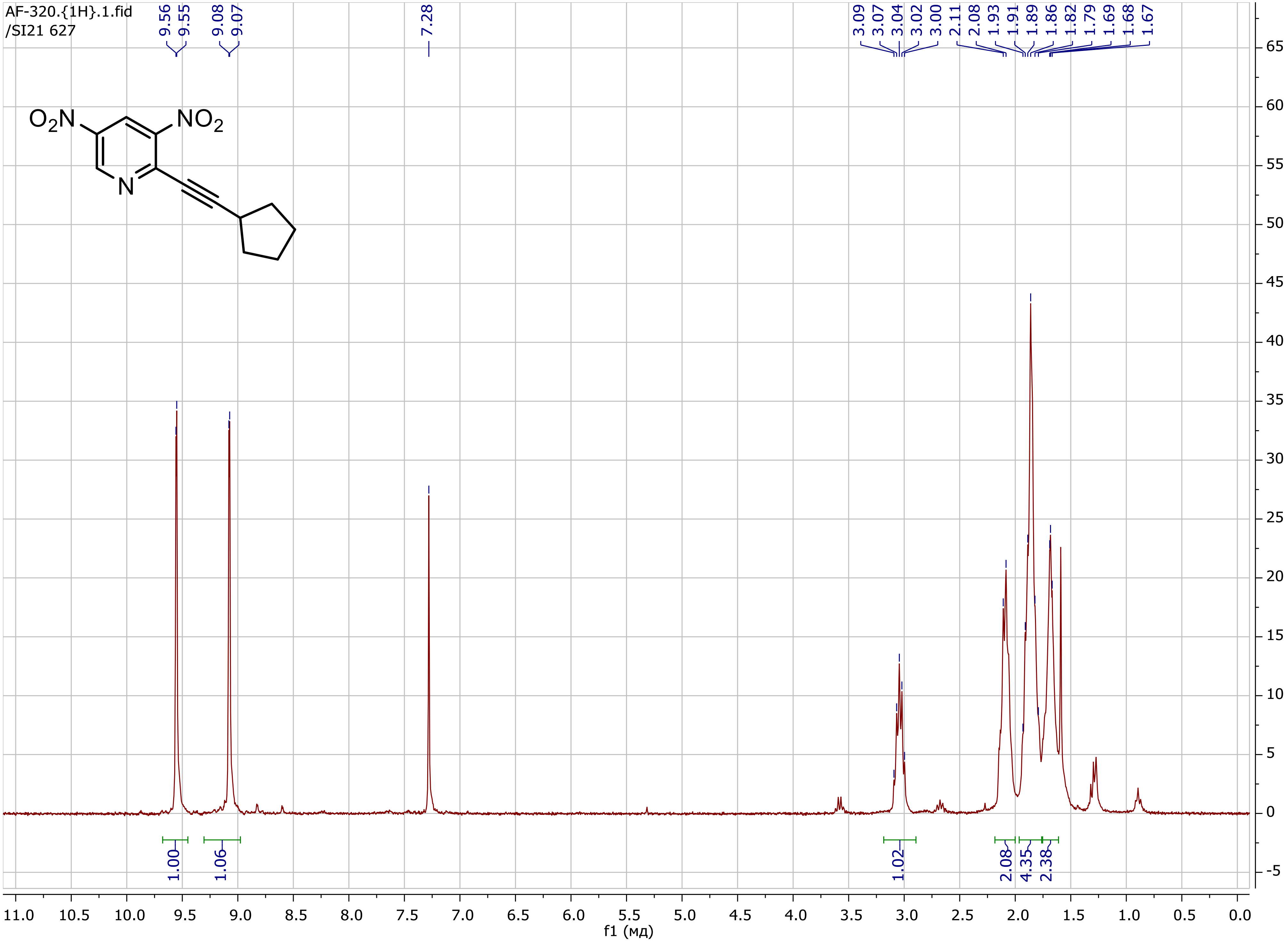
AF-320.{1H}.1.fid
/SI21 627
9.56
9.55
9.08
9.07
7.28
3.09
3.07
3.04
3.02
3.00
2.11
2.08
1.93
1.91
1.89
1.86
1.82
1.79
1.69
1.68
1.67
O2N
NO2
N
1.00
1.06
1.02
2.08
4.35
2.38
f1 (мд)

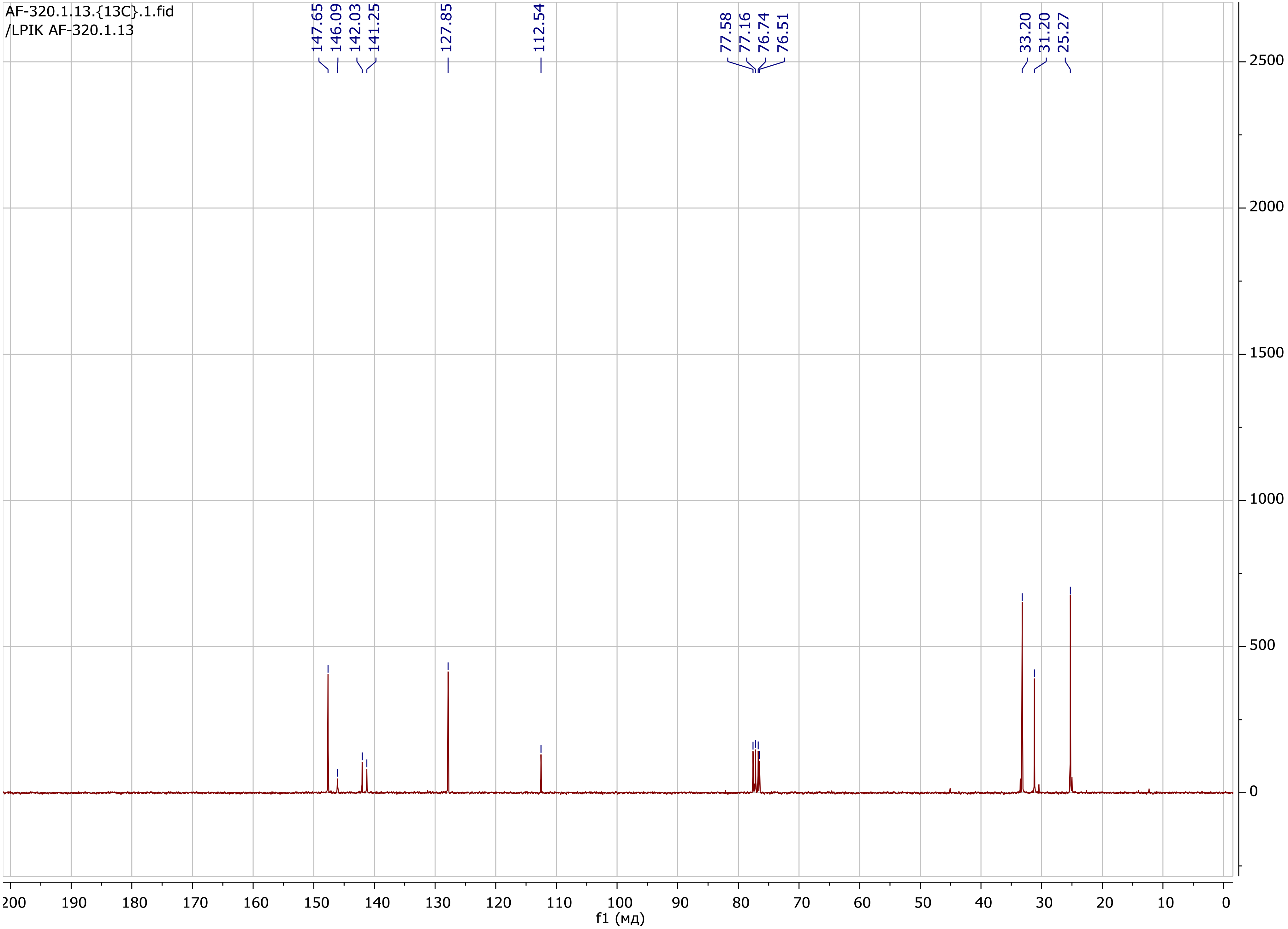
AF-320.1.13.{13C}.1.fid
/LPIK AF-320.1.13
147.65
146.09
142.03
141.25
127.85
112.54
77.58
77.16
76.74
76.51
33.20
31.20
25.27
2500
2000
1500
1000
500
0
200 190 180 170 160 150 140 130 120 110 100 90 80 70 60 50 40 30 20 10 0
f1 (мд)

Display Report

Analysis Info

Analysis Name	D:\Data\Kolotyrkina\2019\Bastrakov\0925011.d	Acquisition Date	25.09.2019 13:14:51
Method	tune_50-1600.m	Operator	BDAL@DE
Sample Name	/LPIK AF-320	Instrument / Ser#	micrOTOF 10248
Comment	C12H11N3O4 mH 262.0822 calibrant added		

Acquisition Parameter

Source Type	ESI	Ion Polarity	Positive	Set Nebulizer	1.0 Bar
Focus	Not active			Set Dry Heater	200 °C
Scan Begin	50 m/z	Set Capillary	4500 V	Set Dry Gas	4.0 l/min
Scan End	1600 m/z	Set End Plate Offset	-500 V	Set Divert Valve	Waste

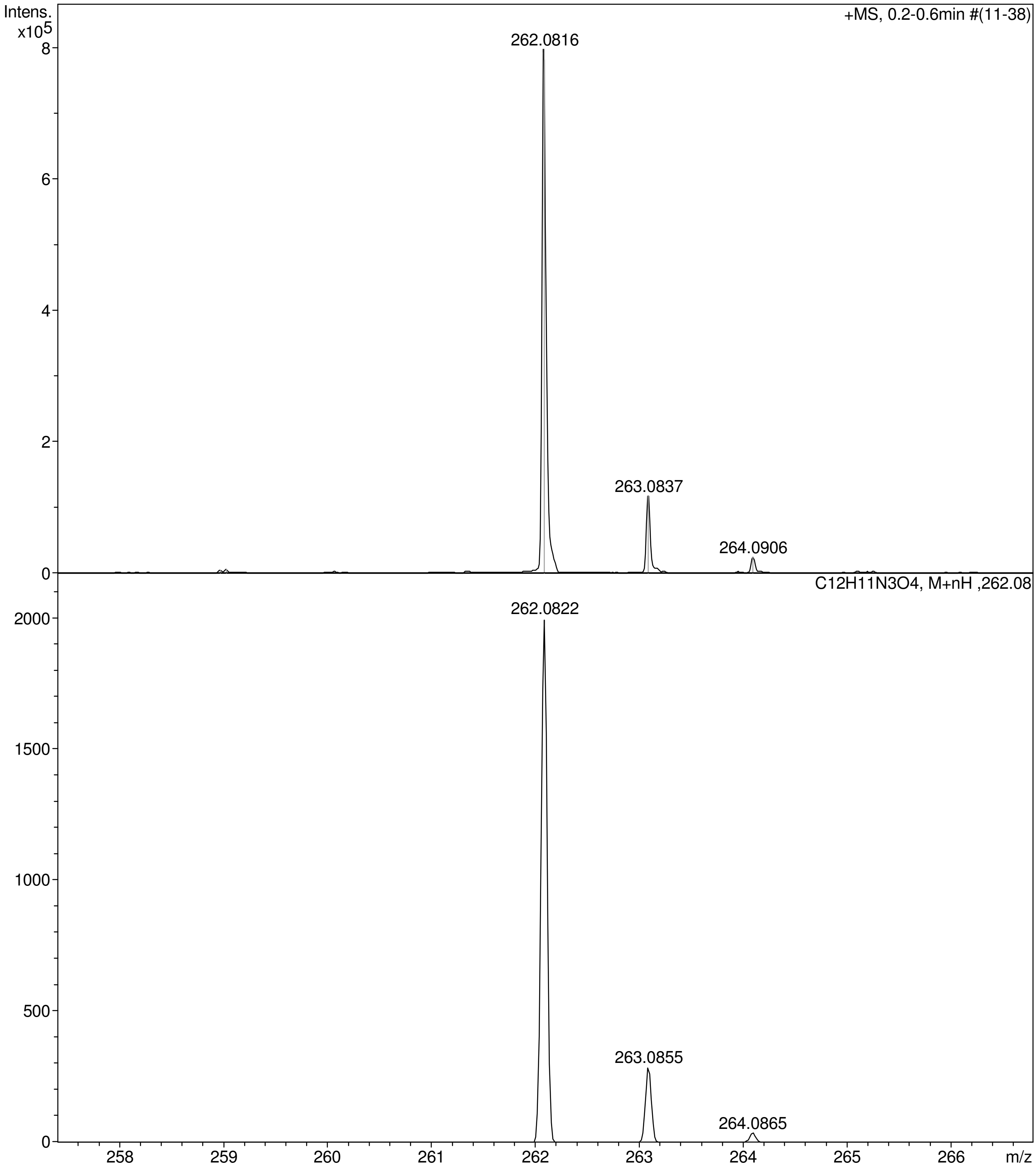

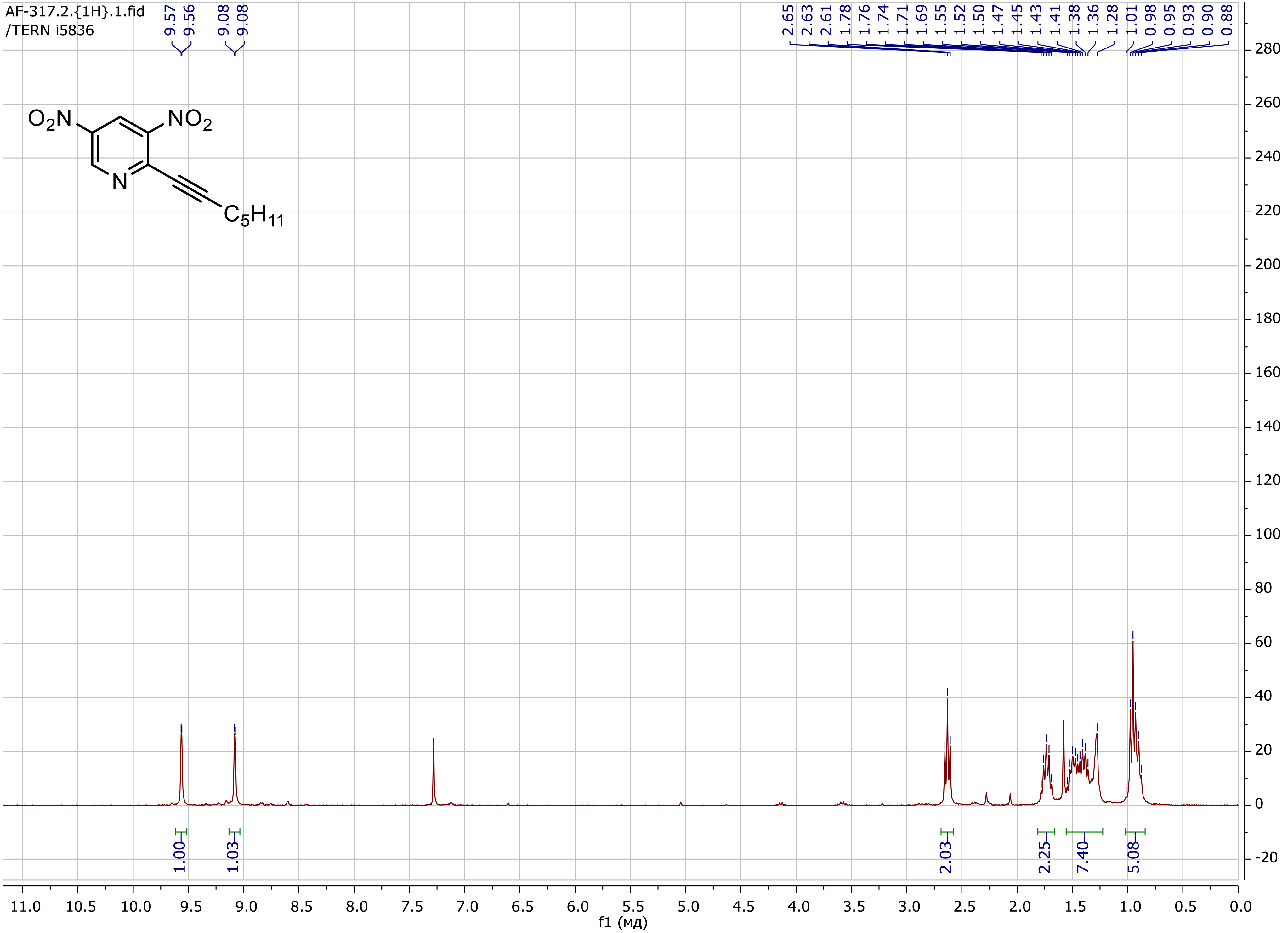
AF-317.2.{1H}.1.fid
/TERN i5836
9.57
9.56
9.08
9.08
2.65
2.63
2.61
1.78
1.76
1.74
1.71
1.69
1.55
1.52
1.50
1.47
1.45
1.43
1.41
1.38
1.36
1.28
1.01
0.98
0.95
0.93
0.90
0.88
O2N
NO2
N
C5H11
1.00
1.03
2.03
2.25
7.40
5.08
f1 (мд)

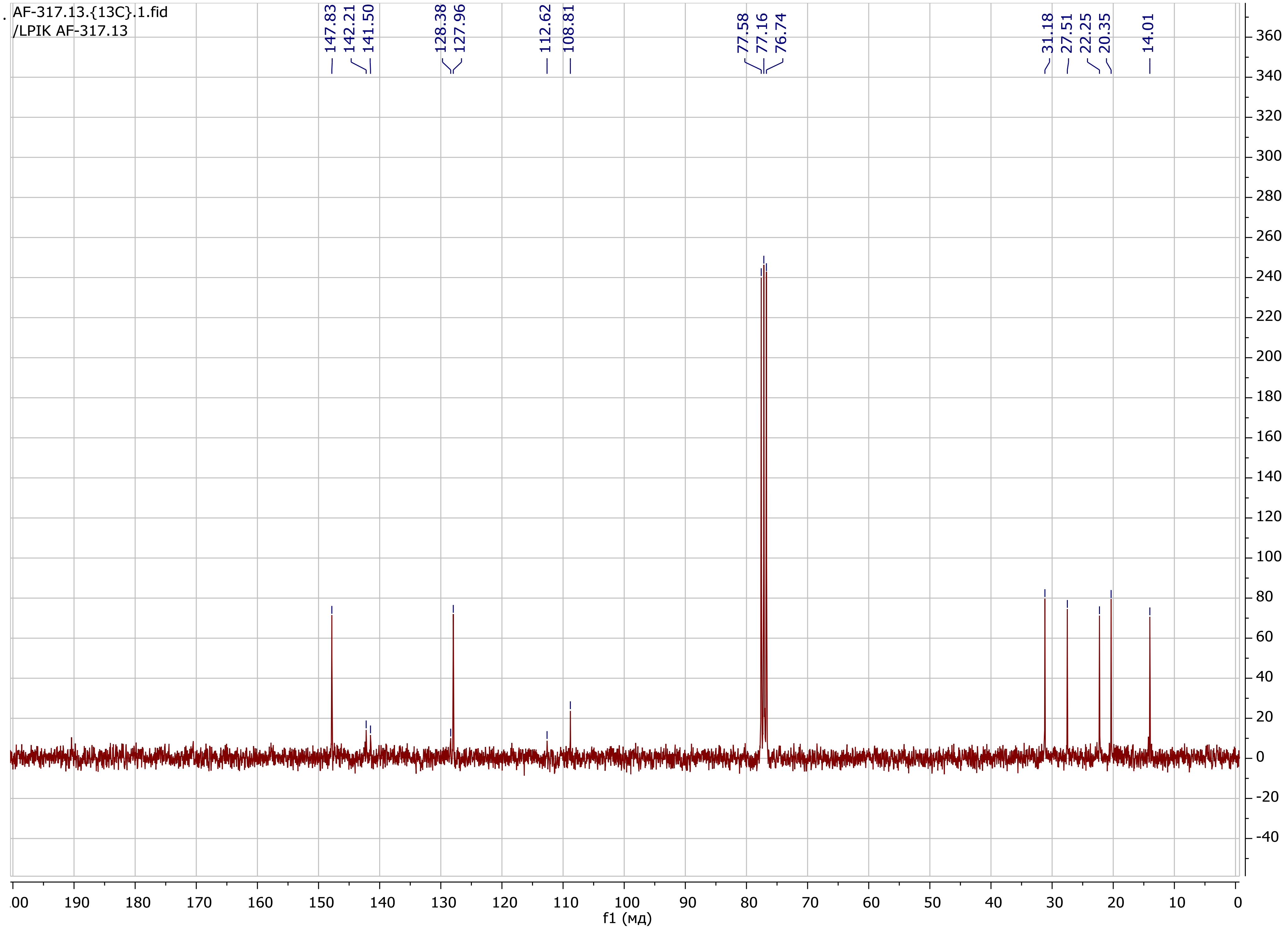

AF-317.13.{13C}.1.fid
/LPIK AF-317.13
147.83
142.21
141.50
128.38
127.96
112.62
108.81
77.58
77.16
76.74
31.18
27.51
22.25
20.35
14.01
f1 (мд)

Display Report

Analysis Info

Analysis Name	D:\Data\Kolotyrkina\2019\Bastrakov\0925002.d	Acquisition Date	25.09.2019 12:13:31
Method	tune_50-1600.m	Operator	BDAL@DE
Sample Name	/LPIK AF-317	Instrument / Ser#	micrOTOF 10248
Comment	C12H13N3O4 mH 264.0979 calibrant added		

Acquisition Parameter

Source Type	ESI	Ion Polarity	Positive	Set Nebulizer	1.0 Bar
Focus	Not active			Set Dry Heater	200 °C
Scan Begin	50 m/z	Set Capillary	4500 V	Set Dry Gas	4.0 l/min
Scan End	1600 m/z	Set End Plate Offset	-500 V	Set Divert Valve	Waste

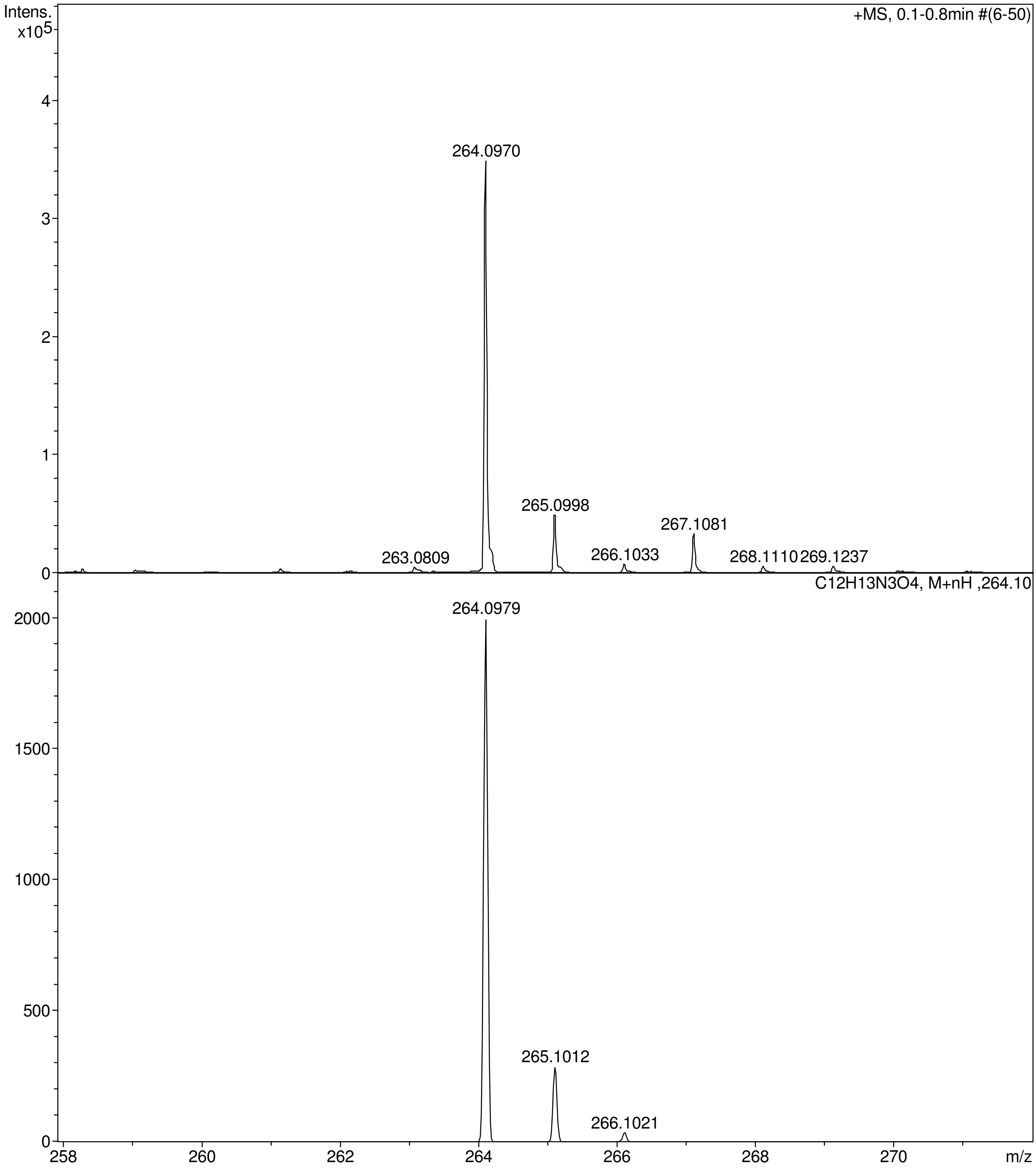

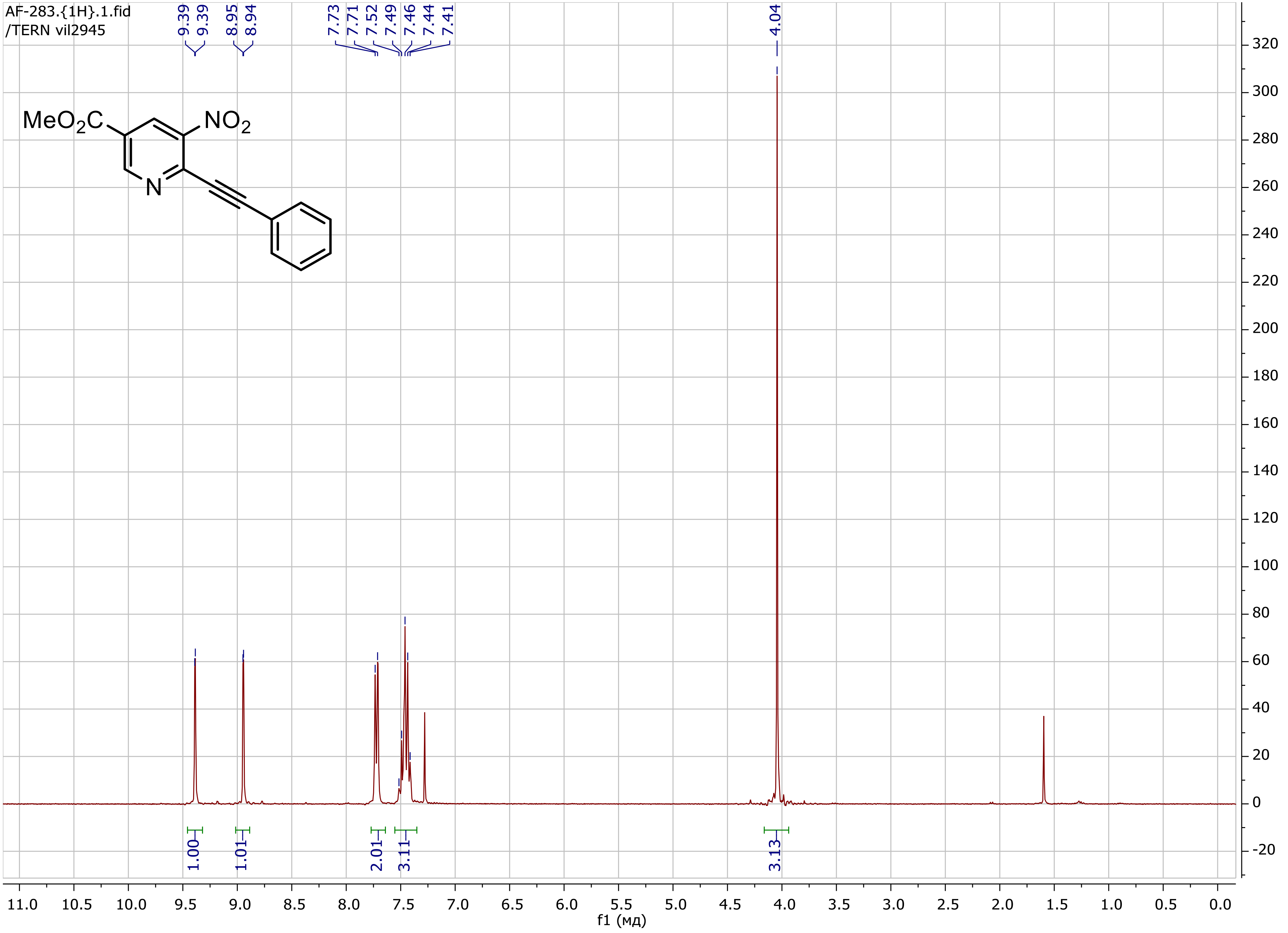
AF-283.{1H}.1.fid
/TERN vil2945
MeO2C
NO2
N
9.39
9.39
8.95
8.94
7.73
7.71
7.52
7.49
7.46
7.44
7.41
4.04
1.00
1.01
2.01
3.11
3.13
f1 (мд)

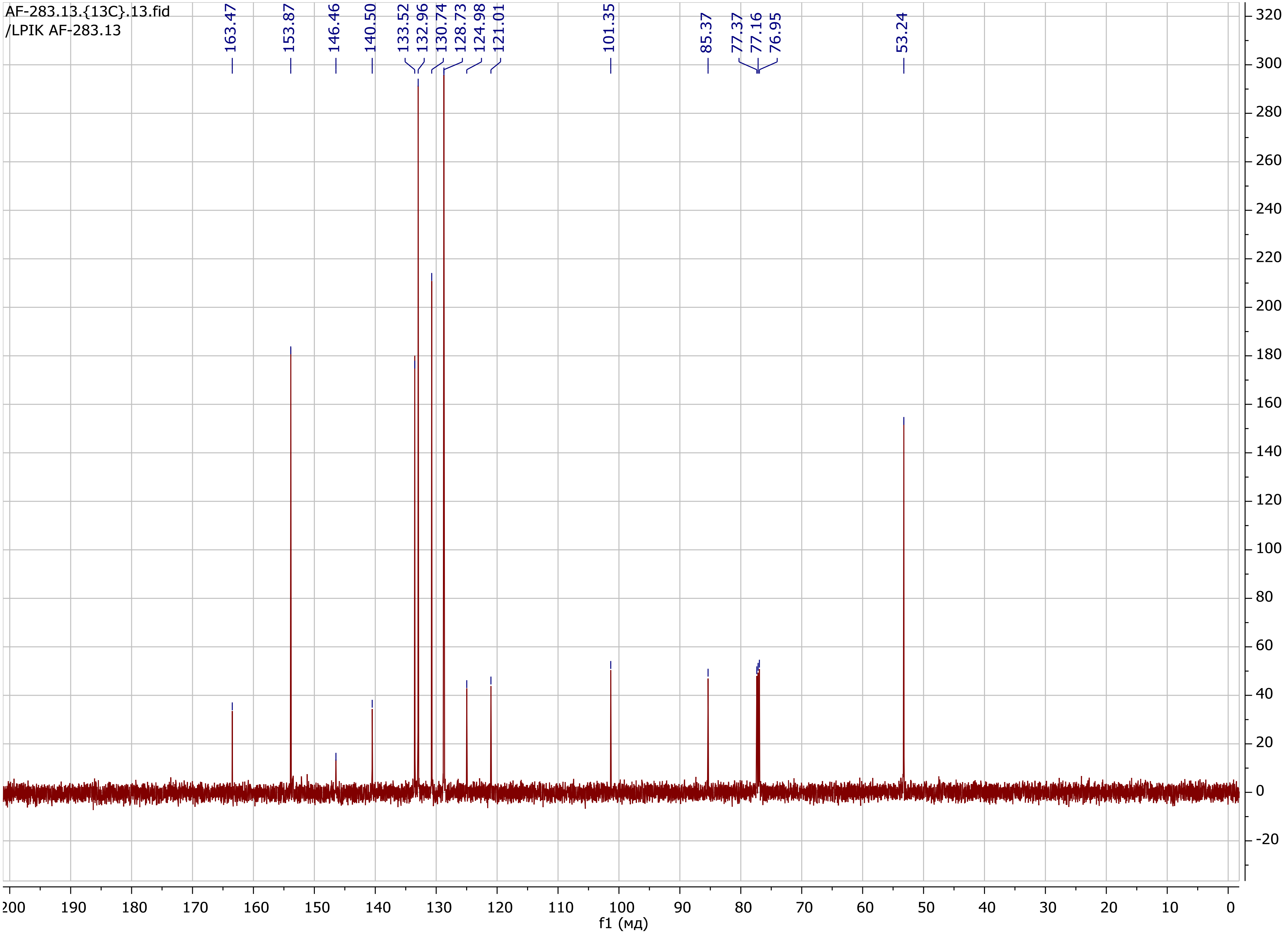
AF-283.13.{13C}.13.fid
/LPIK AF-283.13
163.47
153.87
146.46
140.50
133.52
132.96
130.74
128.73
124.98
121.01
101.35
85.37
77.37
77.16
76.95
53.24
f1 (мд)

Display Report

Analysis Info

Analysis Name	D:\Data\Kolotyrkina\2019\Bastrakov\0423023.d	Acquisition Date	23.04.2019 12:33:25
Method	tune_50-1600.m	Operator	BDAL@DE
Sample Name	/LPIK AF-283	Instrument / Ser#	micrOTOF 10248
Comment	C15H19N2O4 mH 283.0713 calibrant added		

Acquisition Parameter

Source Type	ESI	Ion Polarity	Positive	Set Nebulizer	1.0 Bar
Focus	Not active			Set Dry Heater	200 °C
Scan Begin	50 m/z	Set Capillary	4500 V	Set Dry Gas	4.0 l/min
Scan End	1600 m/z	Set End Plate Offset	-500 V	Set Divert Valve	Waste

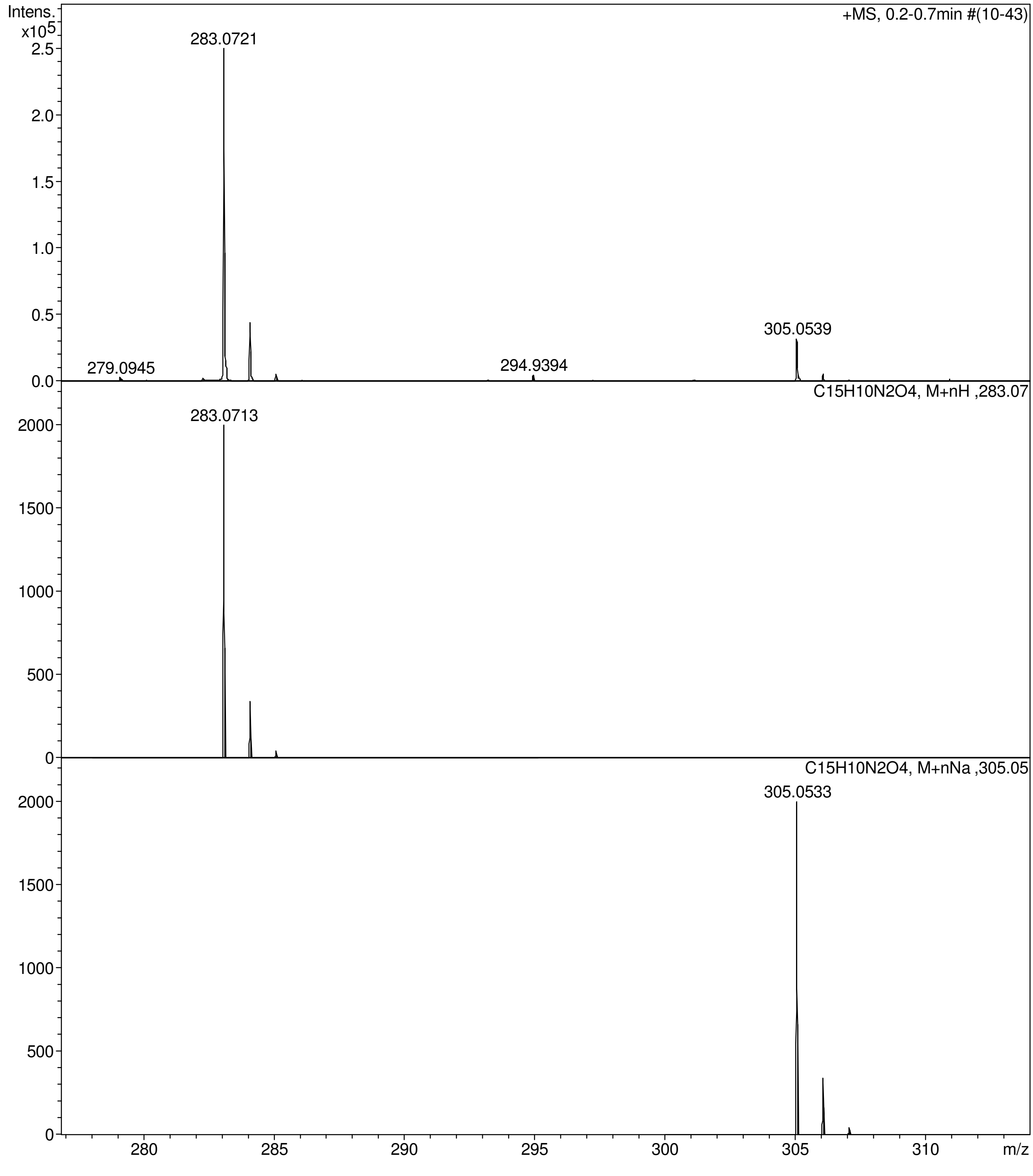

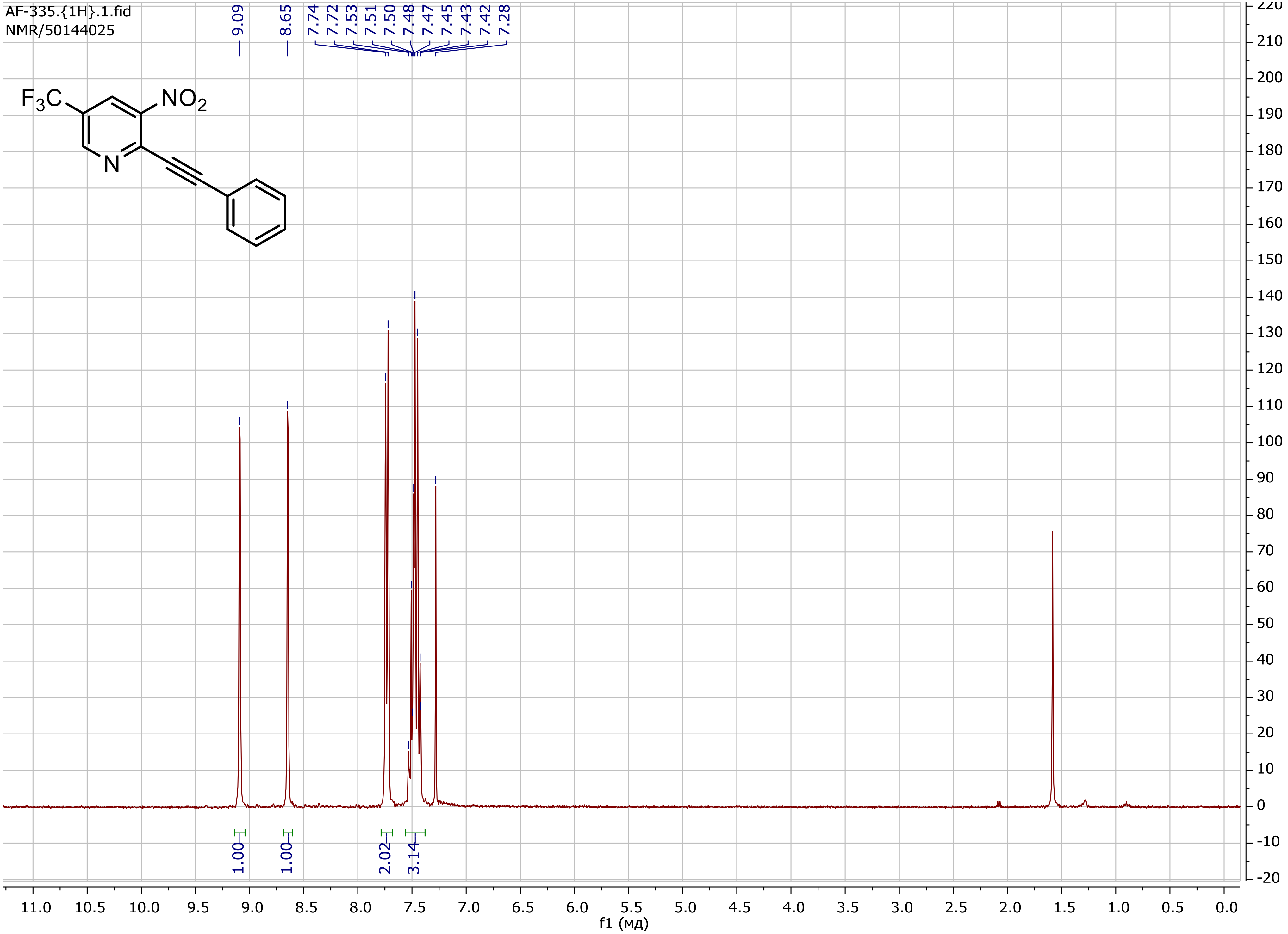
AF-335.{1H}.1.fid
NMR/50144025
F3C
NO2
N
9.09
8.65
7.74
7.72
7.53
7.51
7.50
7.48
7.47
7.45
7.43
7.42
7.28
1.00
1.00
2.02
3.14
f1 (мд)

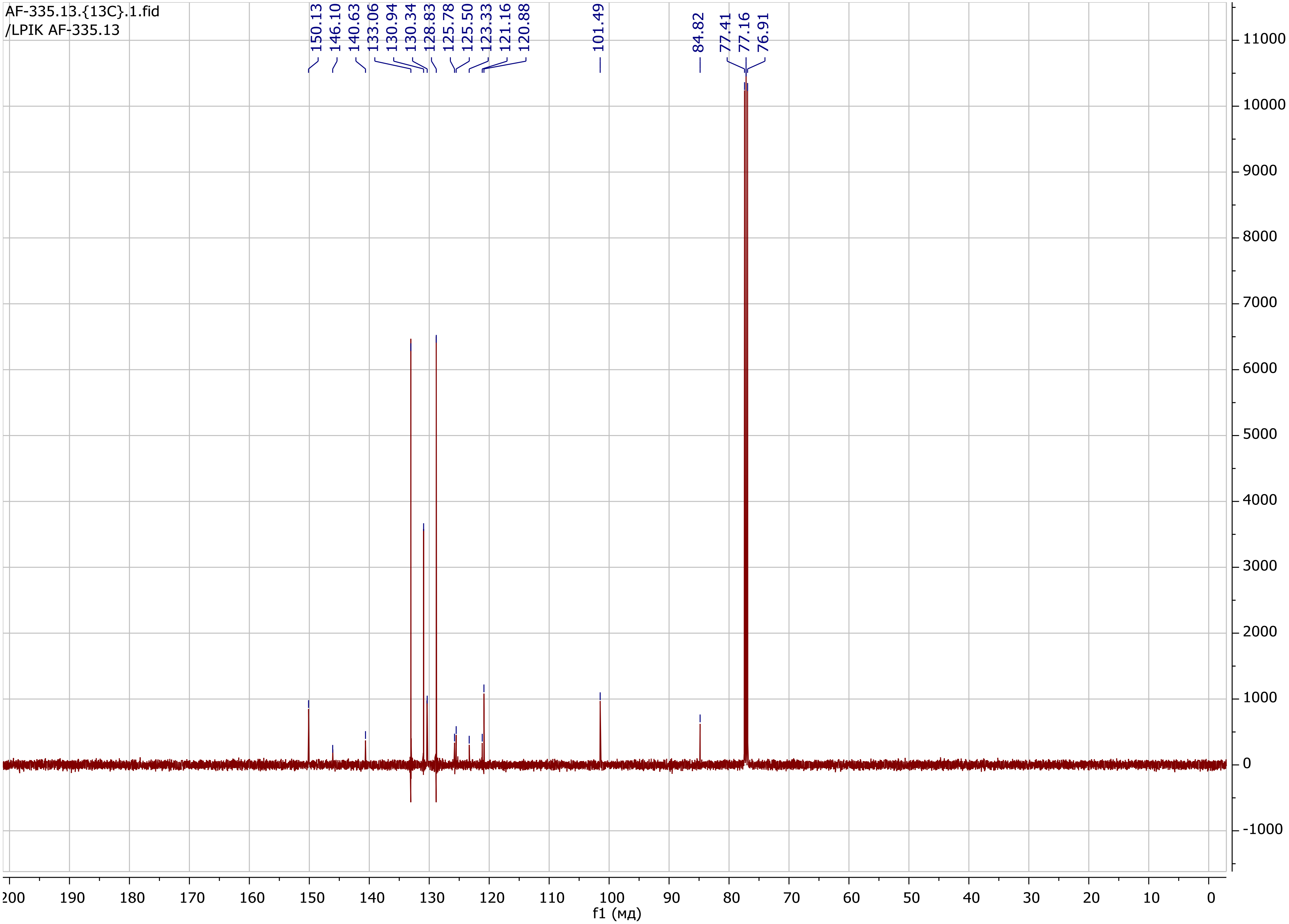

AF-335.13.{13C}.1.fid
/LPIK AF-335.13
150.13
146.10
140.63
133.06
130.94
130.34
128.83
125.78
125.50
123.33
121.16
120.88
101.49
84.82
77.41
77.16
76.91
f1 (мд)

Display Report

Analysis Info

Analysis Name	D:\Data\Kolotyrkina\2020\Bastrakov\0121013.d	Acquisition Date	21.01.2020 15:17:23
Method	tune_50-1600.m	Operator	BDAL@DE
Sample Name	/LPIK AF-335	Instrument / Ser#	micrOTOF 10248
Comment	C14H7F3N2O2 mH 293.0532 calibrant added		

Acquisition Parameter

Source Type	ESI	Ion Polarity	Positive	Set Nebulizer	1.0 Bar
Focus	Not active			Set Dry Heater	200 °C
Scan Begin	50 m/z	Set Capillary	4500 V	Set Dry Gas	4.0 l/min
Scan End	1600 m/z	Set End Plate Offset	-500 V	Set Divert Valve	Waste

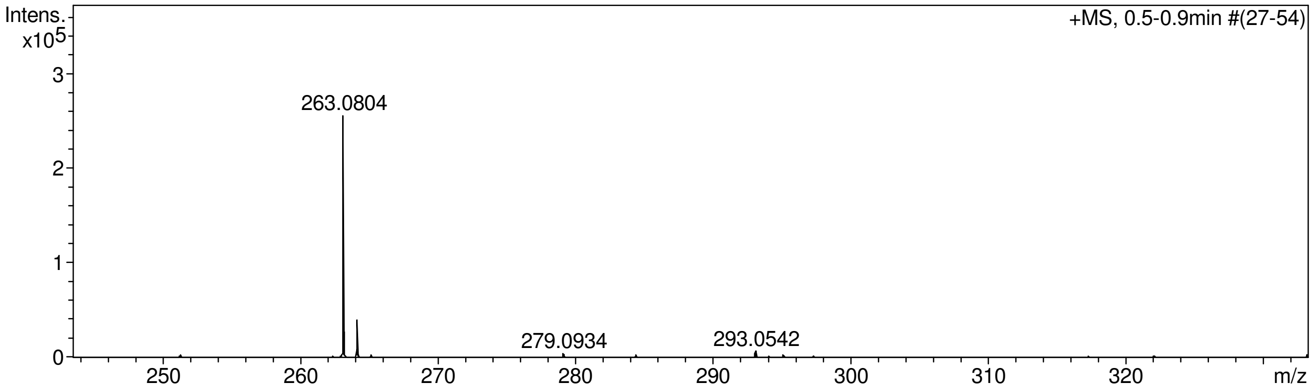

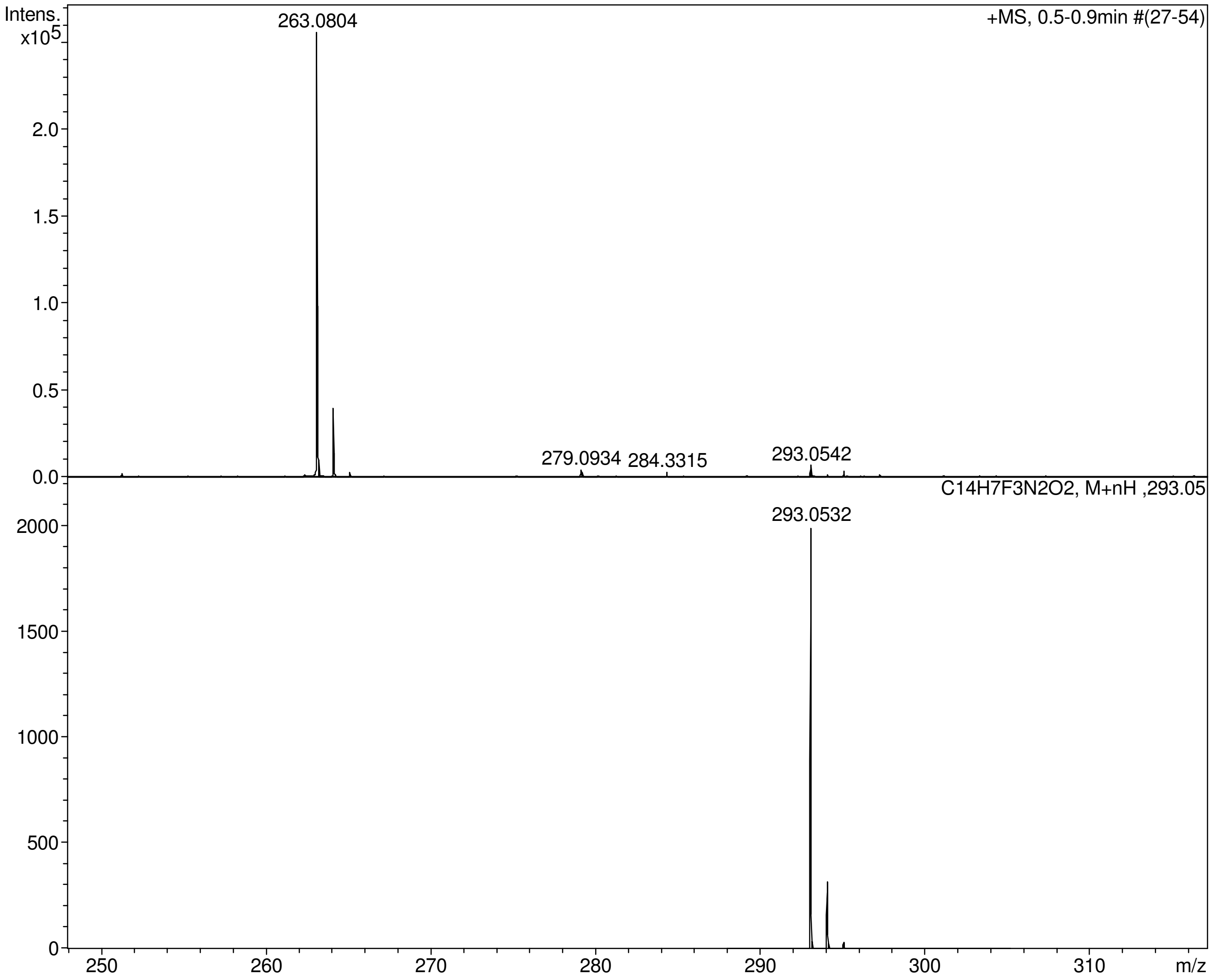

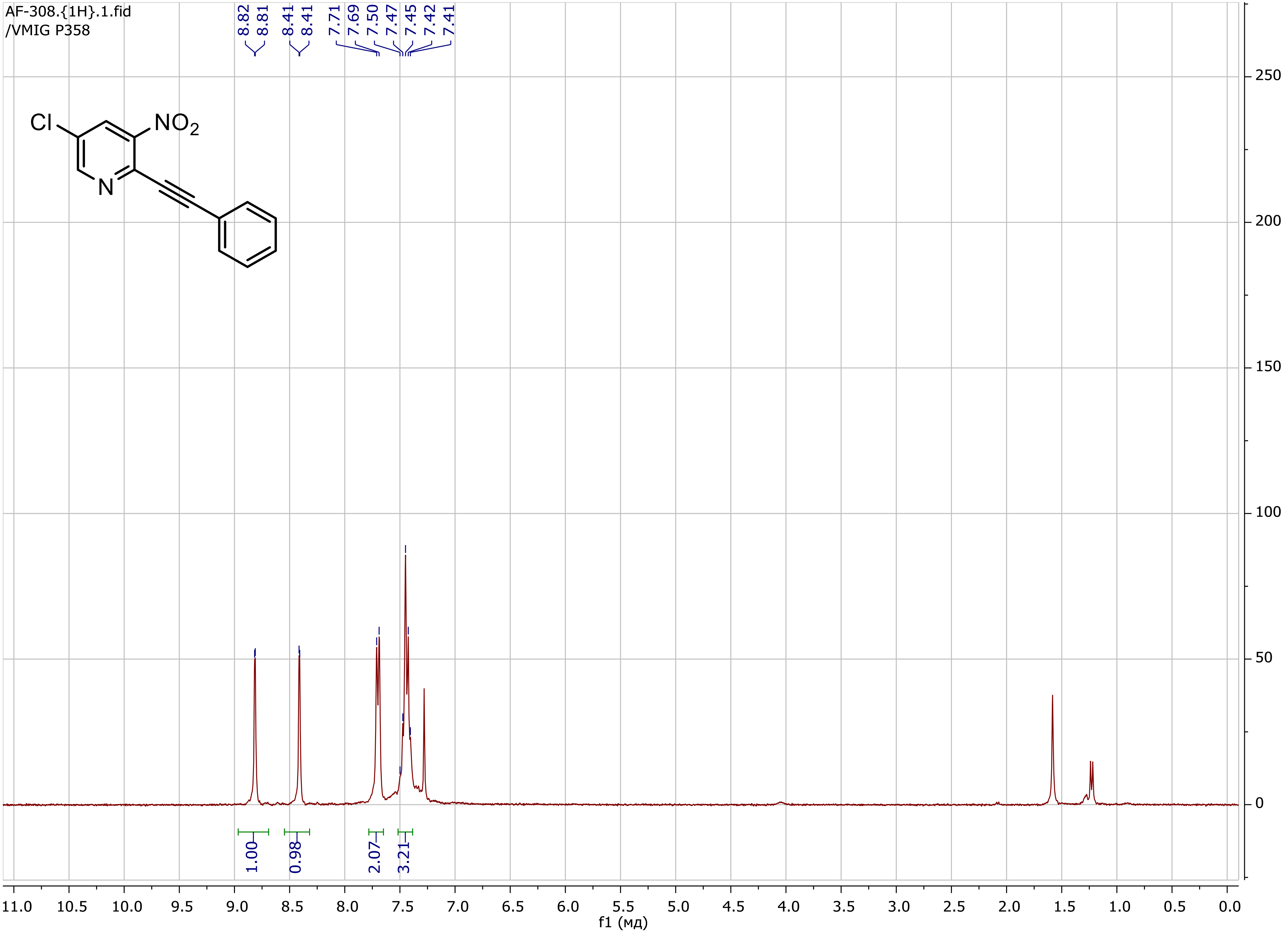

AF-308.{1H}.1.fid
/VMIG P358
8.82
8.81
8.41
8.41
7.71
7.69
7.50
7.47
7.45
7.42
7.41
Cl
NO2
N
1.00
0.98
2.07
3.21
11.0 10.5 10.0 9.5 9.0 8.5 8.0 7.5 7.0 6.5 6.0 5.5 5.0 4.5 4.0 3.5 3.0 2.5 2.0 1.5 1.0 0.5 0.0
f1 (мд)
250
200
150
100
50
0

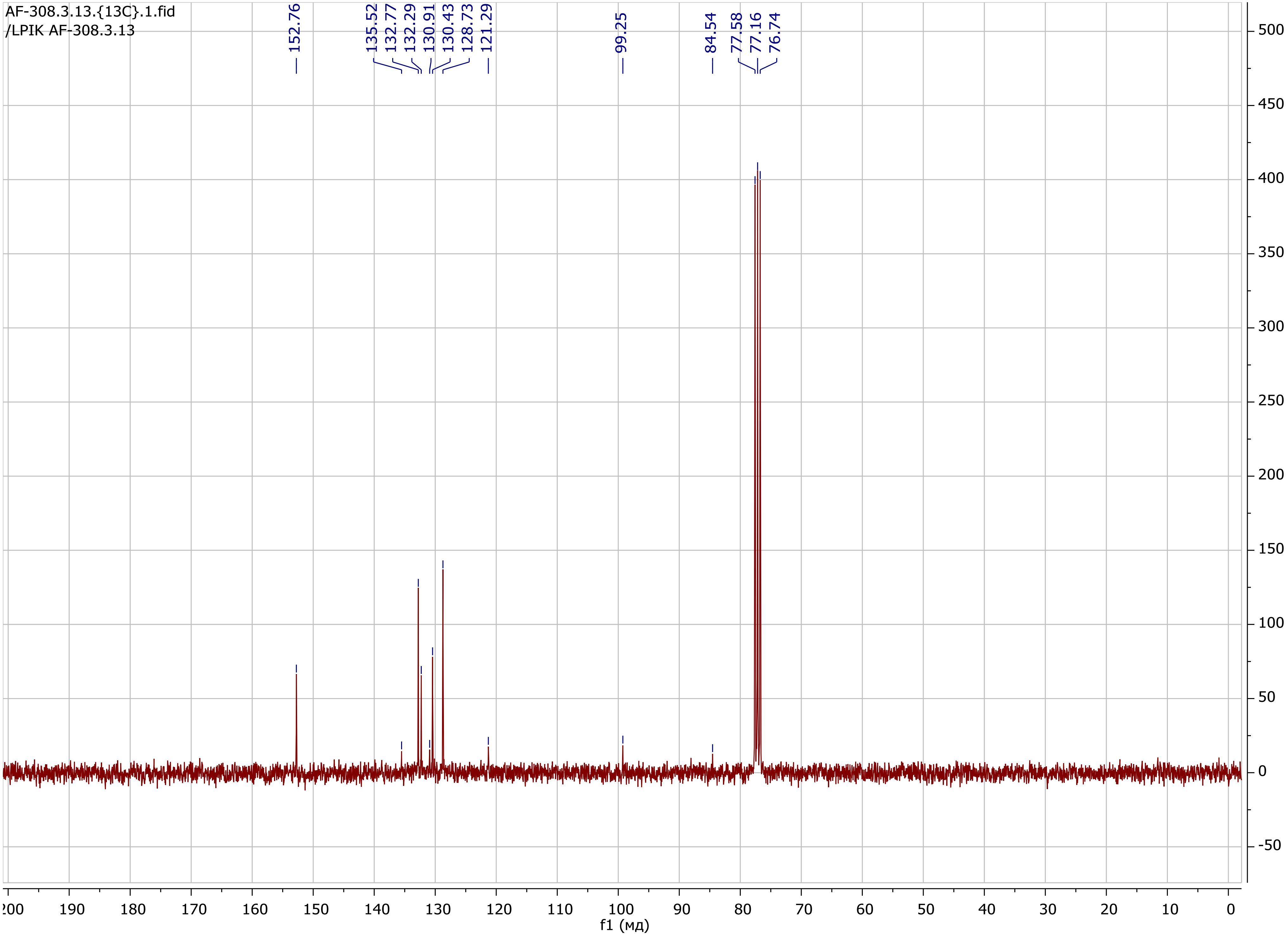
AF-308.3.13.{13C}.1.fid
/LPIK AF-308.3.13
152.76
135.52
132.77
132.29
130.91
130.43
128.73
121.29
99.25
84.54
77.58
77.16
76.74
f1 (мд)

Display Report

Analysis Info

Analysis Name	D:\Data\Kolotyrkina\2019\Bastrakov\0925005.d	Acquisition Date	25.09.2019 12:36:54
Method	tune_50-1600.m	Operator	BDAL@DE
Sample Name	/LPIK AF-308	Instrument / Ser#	micrOTOF 10248
Comment	C13H7ClN2O2 mH 259.0268 calibrant added		

Acquisition Parameter

Source Type	ESI	Ion Polarity	Positive	Set Nebulizer	1.0 Bar
Focus	Not active			Set Dry Heater	200 °C
Scan Begin	50 m/z	Set Capillary	4500 V	Set Dry Gas	4.0 l/min
Scan End	1600 m/z	Set End Plate Offset	-500 V	Set Divert Valve	Waste

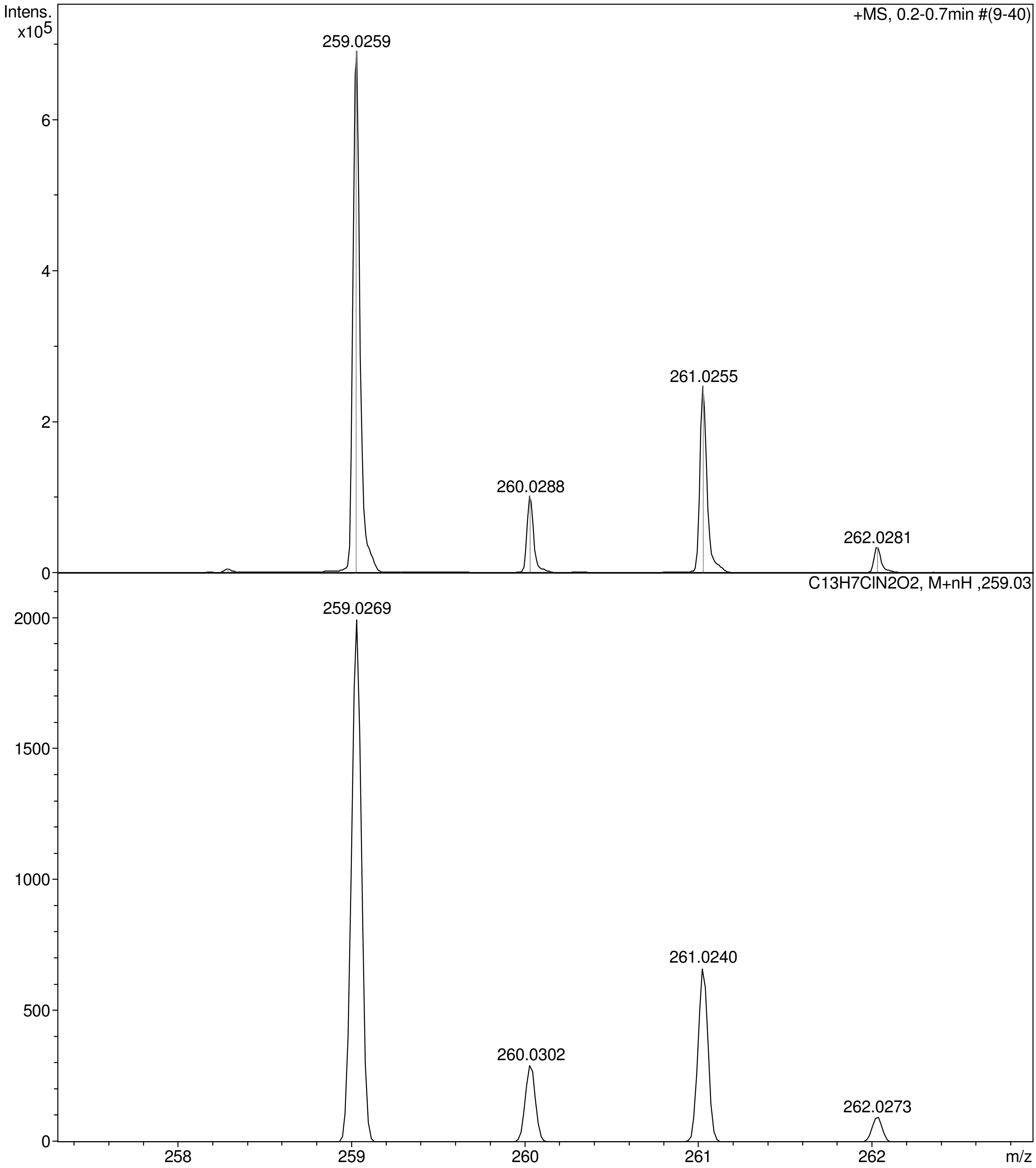

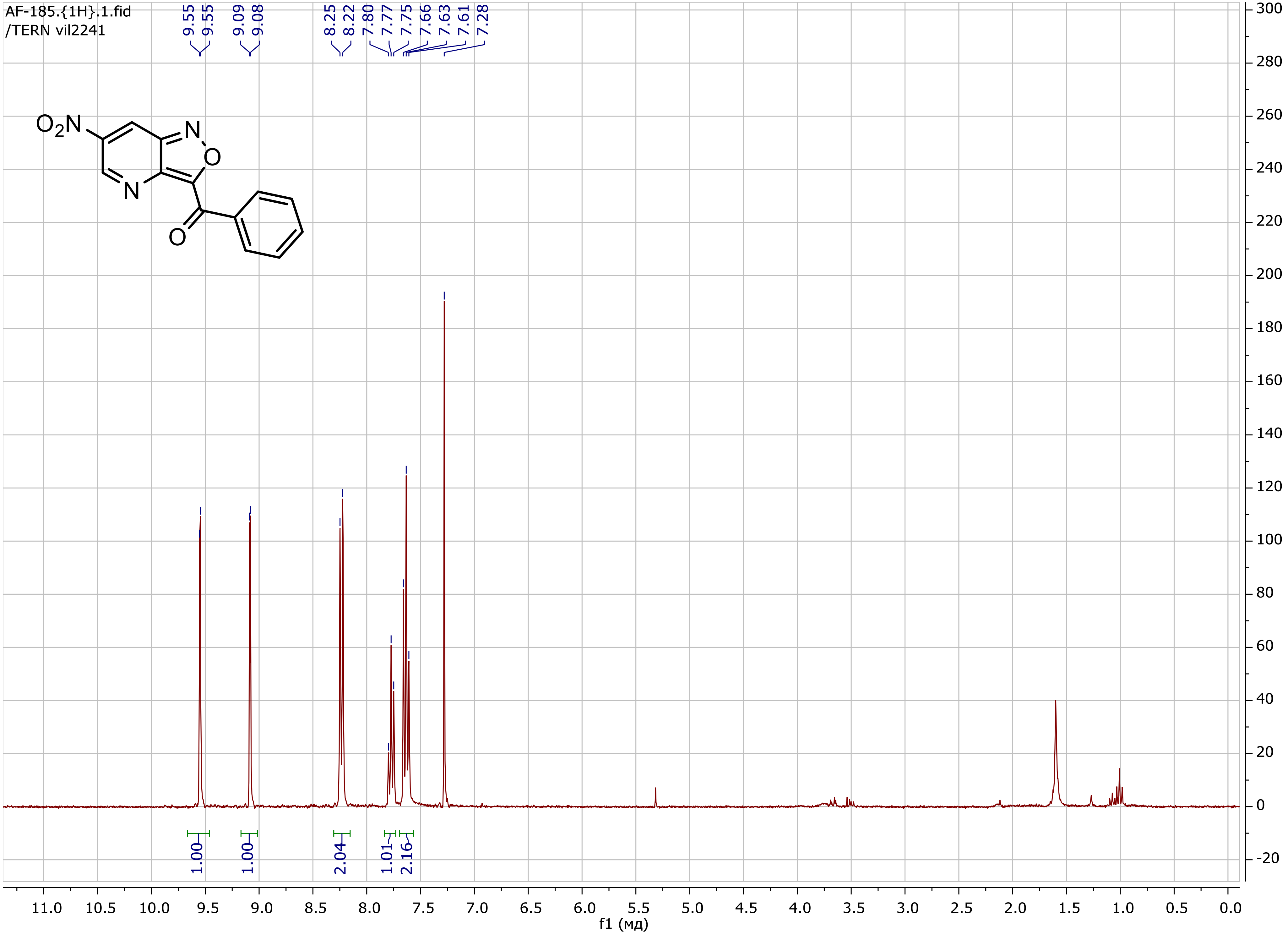

AF-185.{1H}.1.fid
/TERN vil2241
9.55
9.55
9.09
9.08
8.25
8.22
7.80
7.77
7.75
7.66
7.63
7.61
7.28
O2N
N
O
N
O
1.00
1.00
2.04
1.01
2.16
f1 (мд)

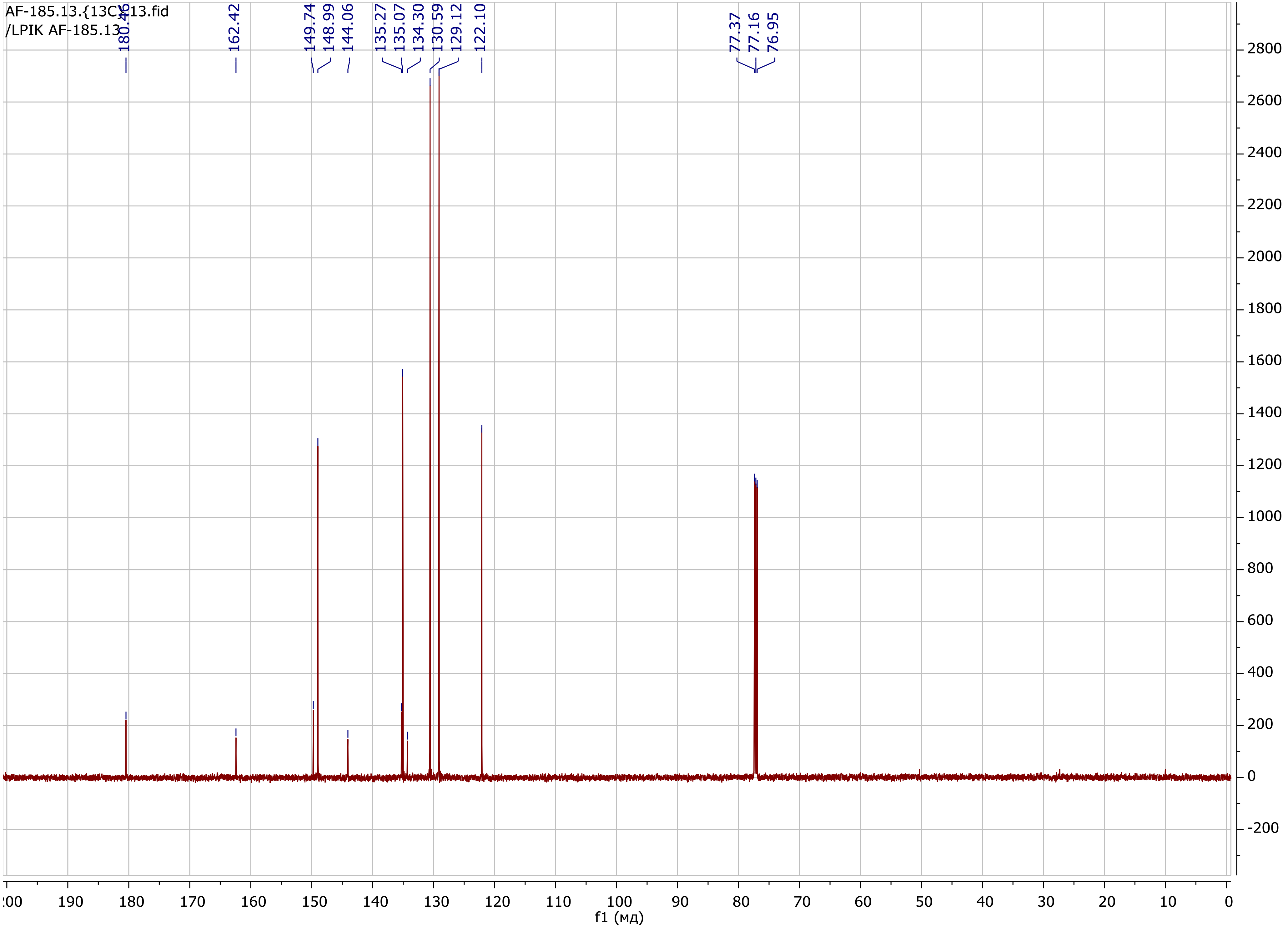
AF-185.13.{13C}13.fid
/LPIK AF-185.13
180.46
162.42
149.74
148.99
144.06
135.27
135.07
134.30
130.59
129.12
122.10
77.37
77.16
76.95
f1 (мд)

Display Report

Analysis Info

Analysis Name	D:\Data\Kolotyrkina\2018\Bastrakov\1113027.d	Acquisition Date	13.11.2018 13:07:39
Method	tune_50-1600.m	Operator	BDAL@DE
Sample Name	/LPIK AF-185	Instrument / Ser#	micrOTOF 10248
Comment	C13H7N3O4 mH 270.0509 calibrant added		

Acquisition Parameter

Source Type	ESI	Ion Polarity	Positive	Set Nebulizer	1.0 Bar
Focus	Not active			Set Dry Heater	200 °C
Scan Begin	50 m/z	Set Capillary	4500 V	Set Dry Gas	4.0 l/min
Scan End	1600 m/z	Set End Plate Offset	-500 V	Set Divert Valve	Waste

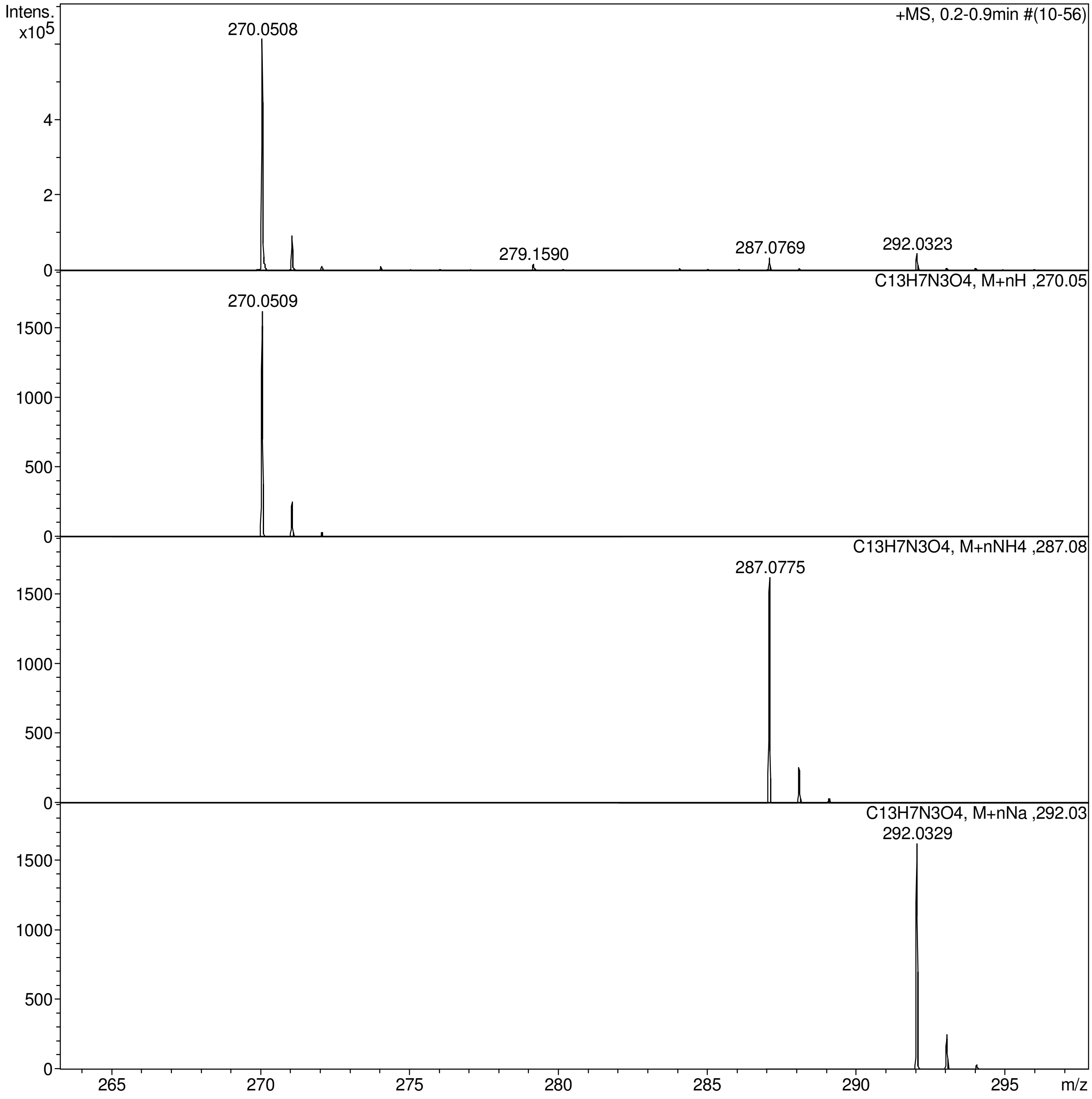

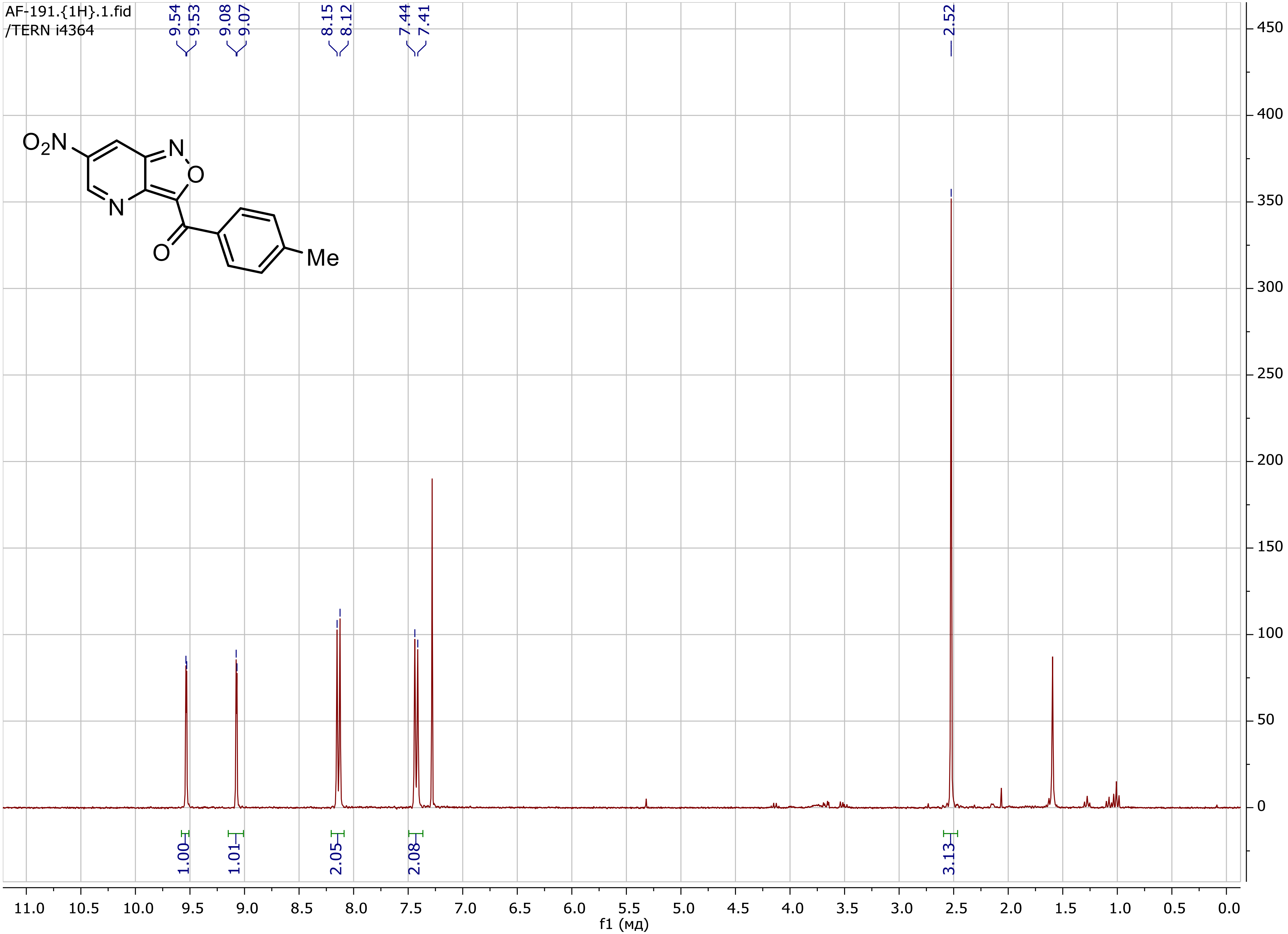
AF-191.{1H}.1.fid
/TERN i4364
9.54
9.53
9.08
9.07
8.15
8.12
7.44
7.41
2.52
O2N
N
O
N
O
Me
1.00
1.01
2.05
2.08
3.13
450
400
350
300
250
200
150
100
50
0
11.0
10.5
10.0
9.5
9.0
8.5
8.0
7.5
7.0
6.5
6.0
5.5
5.0
4.5
4.0
3.5
3.0
2.5
2.0
1.5
1.0
0.5
0.0
f1 (мд)

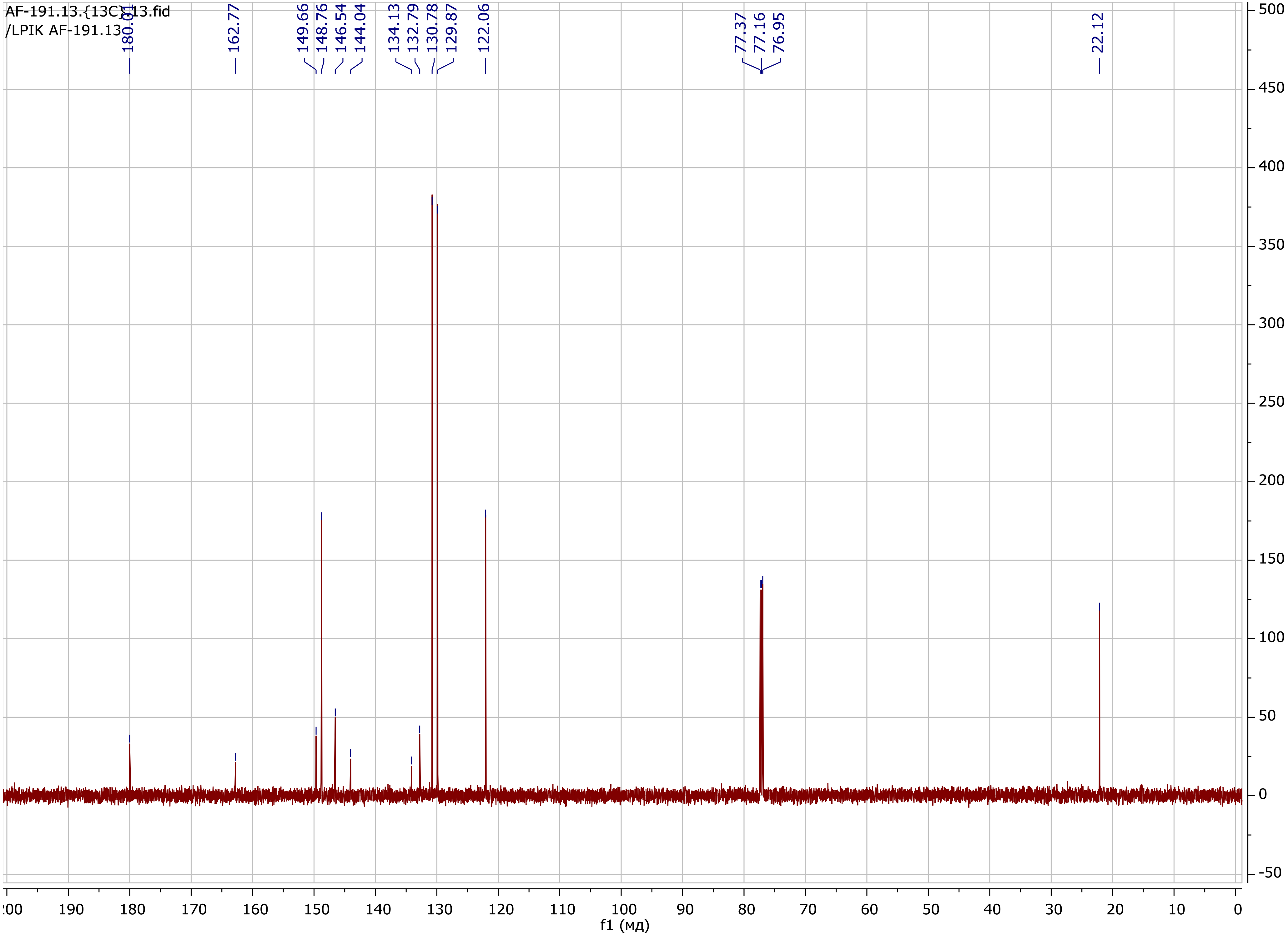
AF-191.13.{13C}13.fid
/LPIK AF-191.13
180.01
162.77
149.66
148.76
146.54
144.04
134.13
132.79
130.78
129.87
122.06
77.37
77.16
76.95
22.12
f1 (мд)

Display Report

Analysis Info

Analysis Name	D:\Data\Kolotyrkina\2018\Bastrakov\1113028.d	Acquisition Date	13.11.2018 13:12:57
Method	tune_50-1600.m	Operator	BDAL@DE
Sample Name	/LPIK AF-191	Instrument / Ser#	micrOTOF 10248
Comment	C14H9N3O4 mH 284.0665 calibrant added		

Acquisition Parameter

Source Type	ESI	Ion Polarity	Positive	Set Nebulizer	1.0 Bar
Focus	Not active			Set Dry Heater	200 ℃
Scan Begin	50 m/z	Set Capillary	4500 V	Set Dry Gas	4.0 l/min
Scan End	1600 m/z	Set End Plate Offset	-500 V	Set Divert Valve	Waste

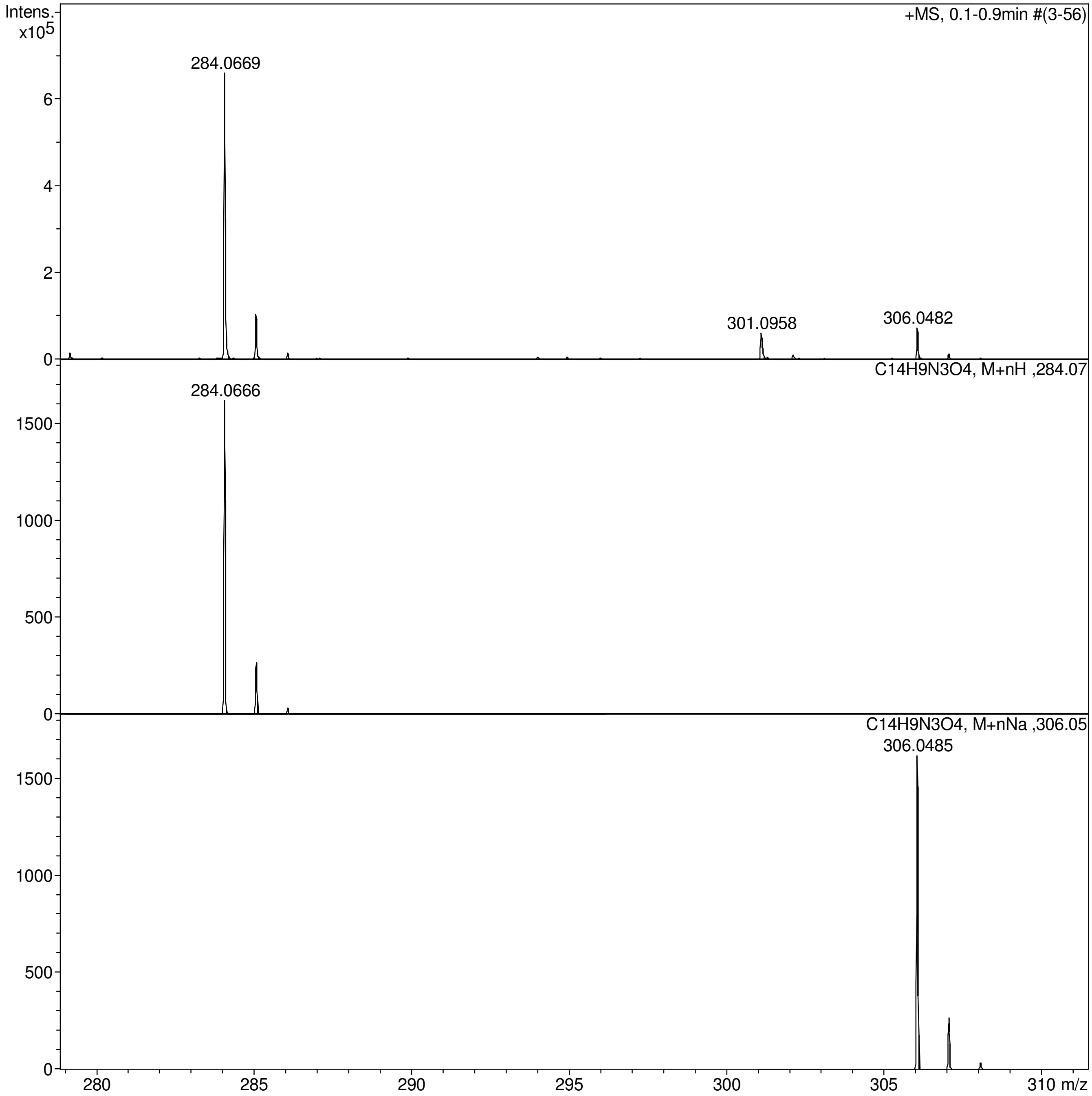

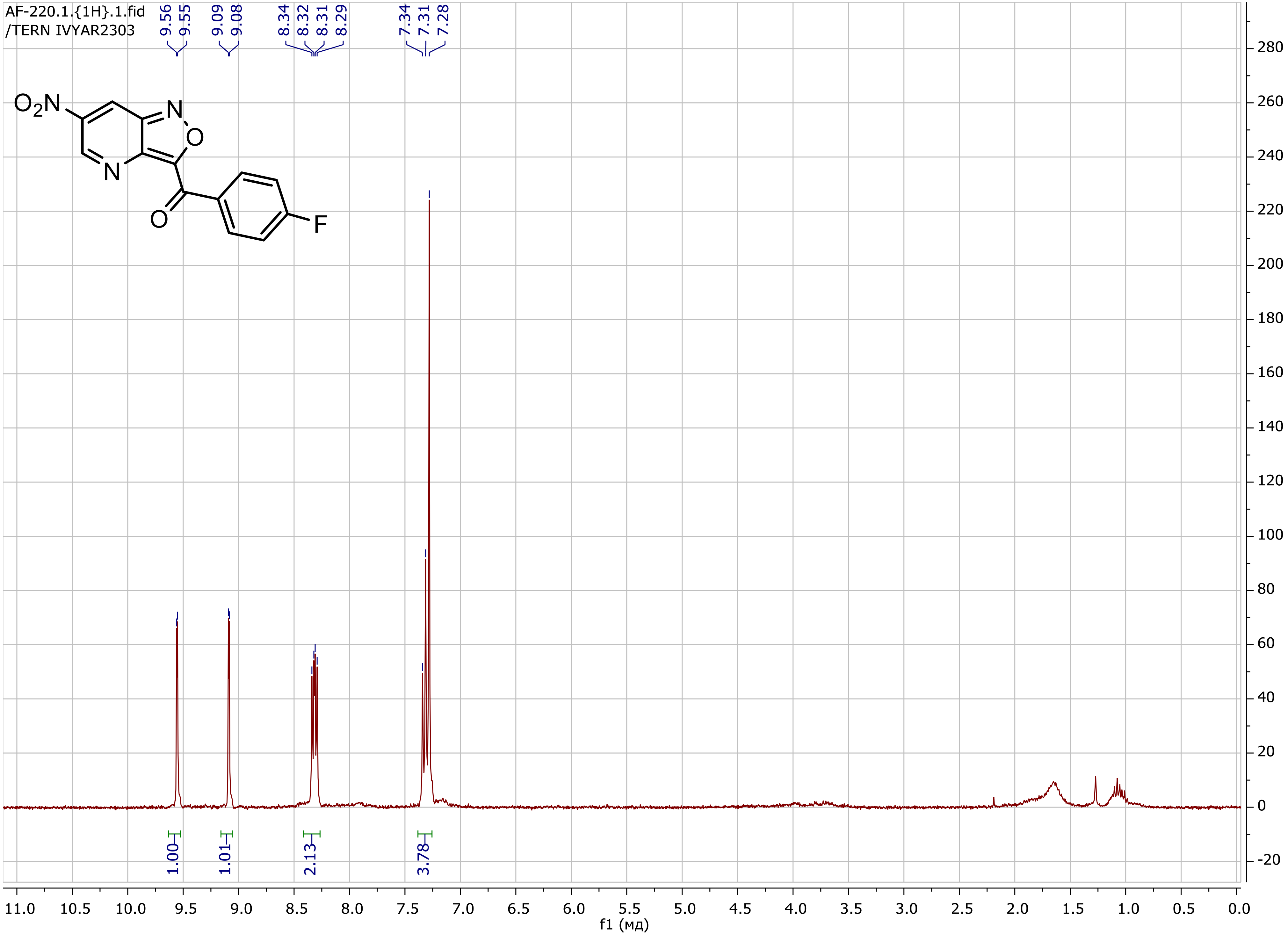

AF-220.1.{1H}.1.fid
/TERN IVYAR2303
9.56
9.55
9.09
9.08
8.34
8.32
8.31
8.29
7.34
7.31
7.28
O_2N
N
O
N
O
F
1.00
1.01
2.13
3.78
f1 (мд)

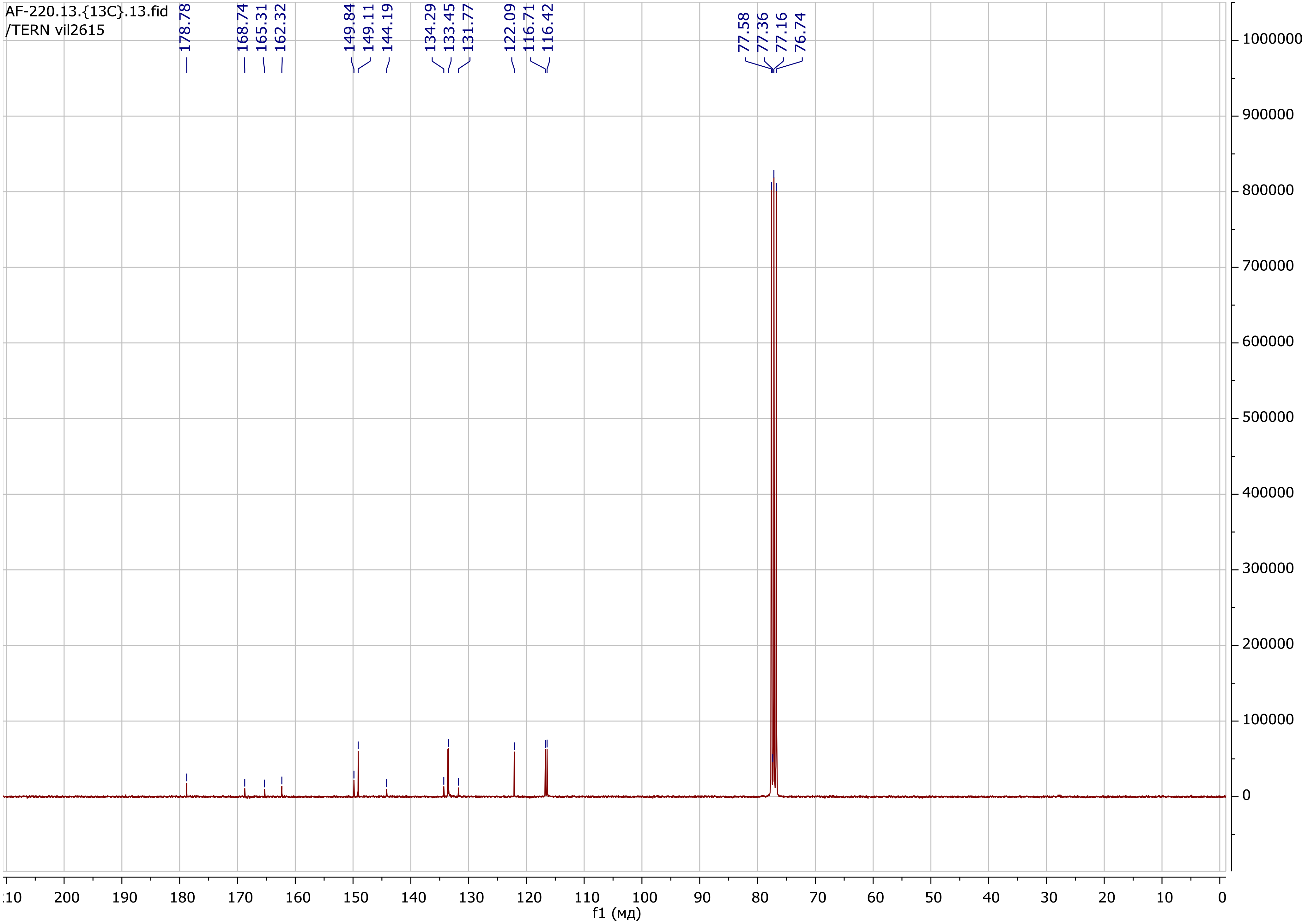
AF-220.13.{13C}.13.fid
/TERN vil2615
178.78
168.74
165.31
162.32
149.84
149.11
144.19
134.29
133.45
131.77
122.09
116.71
116.42
77.58
77.36
77.16
76.74
f1 (мд)

Display Report

Analysis Info

Analysis Name	D:\Data\Kolotyrkina\2019\Bastrakov\0326039.d	Acquisition Date	26.03.2019 18:58:20
Method	tune_50-1600.m	Operator	BDAL@DE
Sample Name	/LPIK AF-220	Instrument / Ser#	micrOTOF 10248
Comment	C13H6FN3O4 mH 288.0415 calibrant added CH3CN		

Acquisition Parameter

Source Type	ESI	Ion Polarity	Positive	Set Nebulizer	1.0 Bar
Focus	Not active			Set Dry Heater	200 °C
Scan Begin	50 m/z	Set Capillary	4500 V	Set Dry Gas	4.0 l/min
Scan End	1600 m/z	Set End Plate Offset	-500 V	Set Divert Valve	Waste

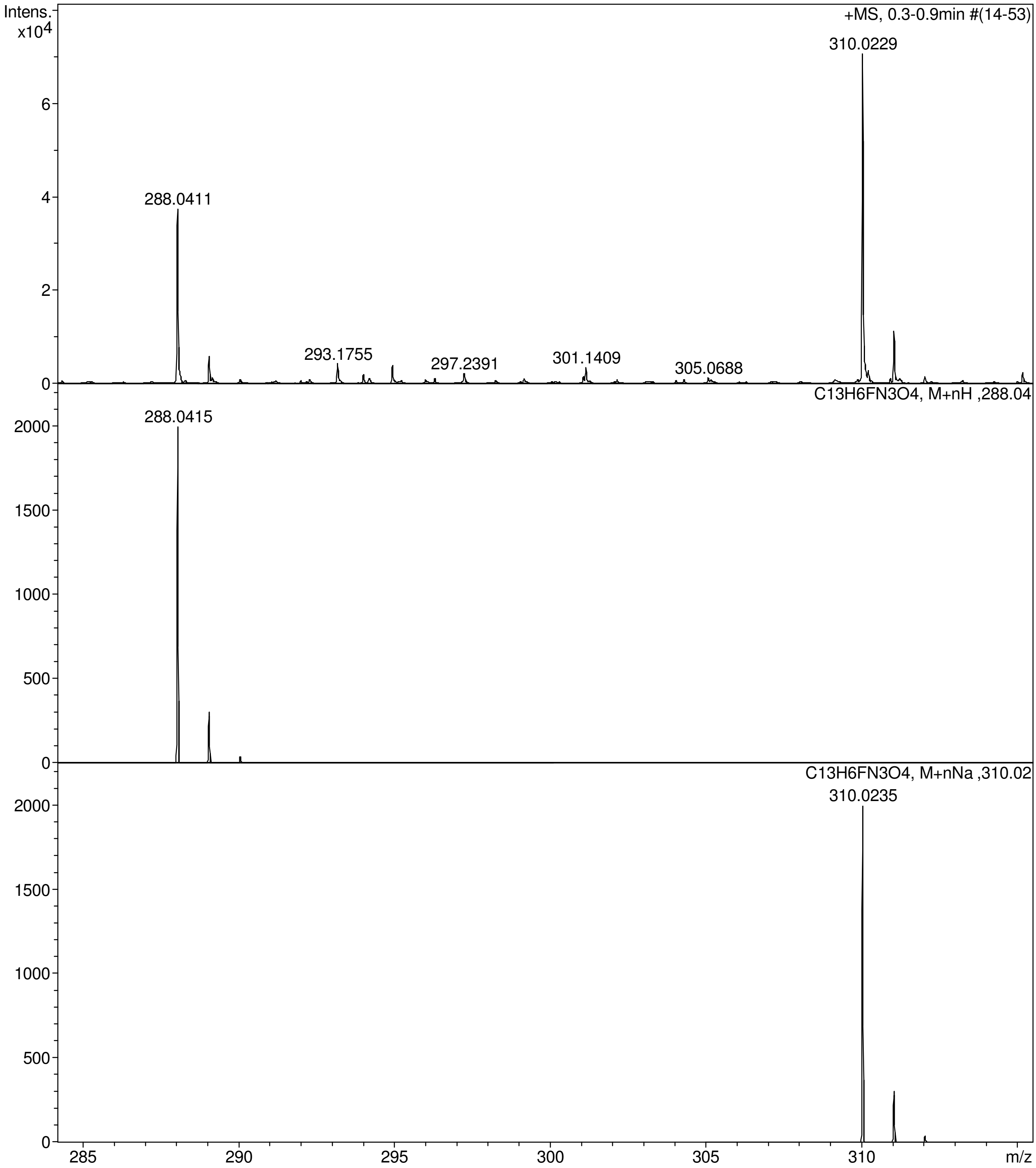

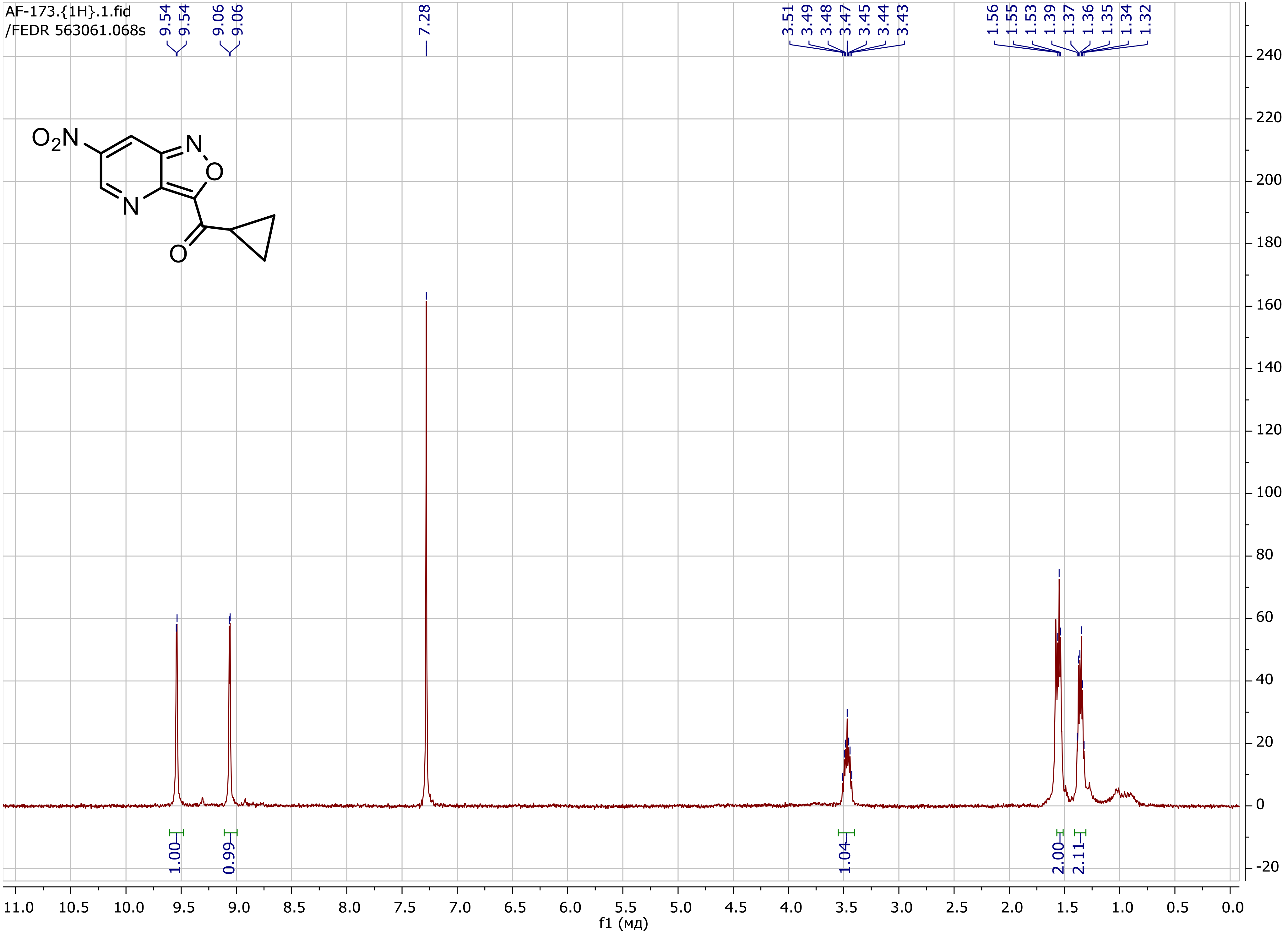
AF-173.{1H}.1.fid
/FEDR 563061.068s
9.54 9.54 9.06 9.06 7.28 3.51 3.49 3.48 3.47 3.45 3.44 3.43 1.56 1.55 1.53 1.39 1.37 1.36 1.35 1.34 1.32
O_2N
N
O
N
O
1.00
0.99
1.04
2.00
2.11
11.0 10.5 10.0 9.5 9.0 8.5 8.0 7.5 7.0 6.5 6.0 5.5 5.0 4.5 4.0 3.5 3.0 2.5 2.0 1.5 1.0 0.5 0.0
f1 (мд)
240 220 200 180 160 140 120 100 80 60 40 20 0 -20

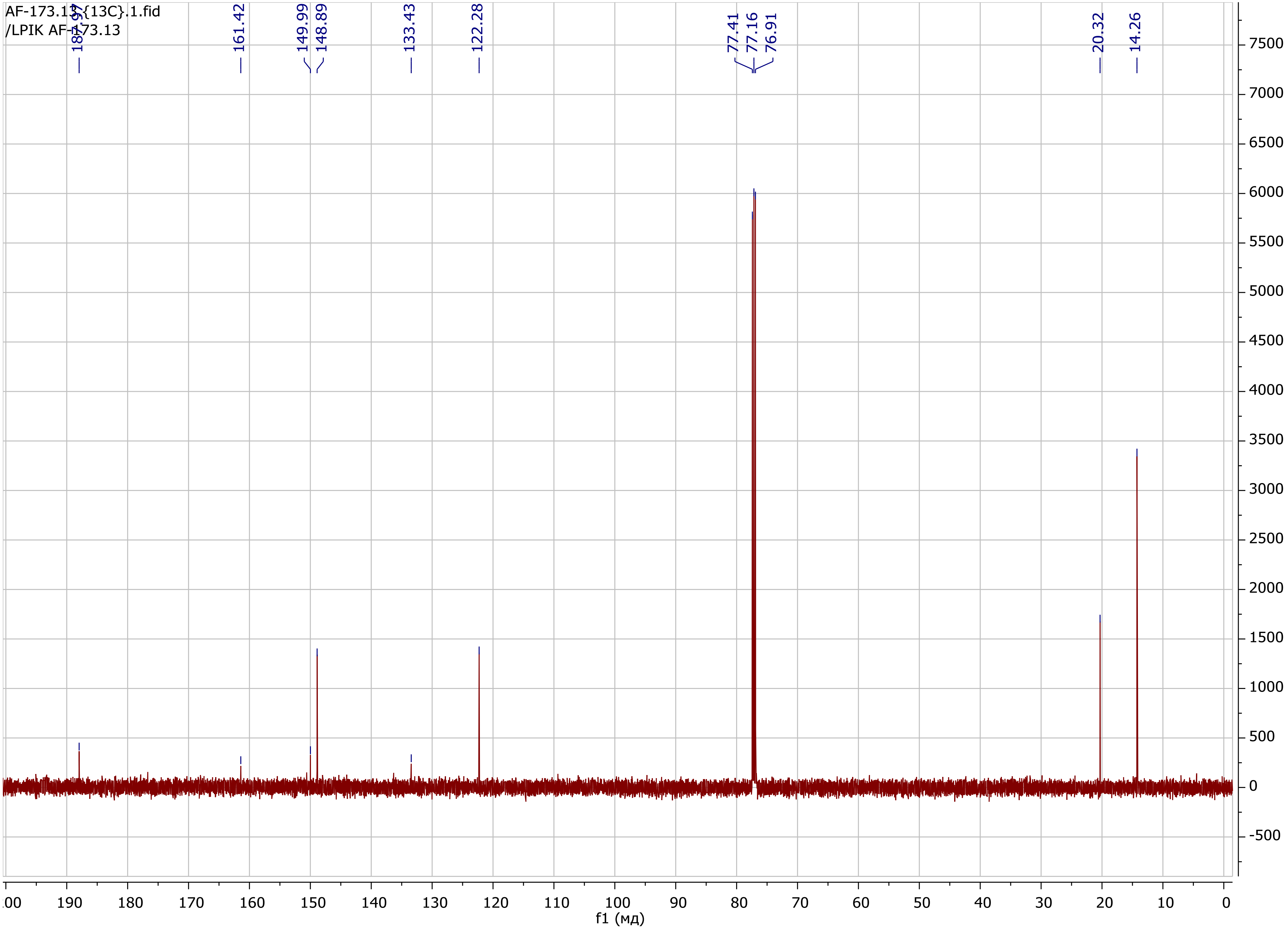
AF-173.13.{13C}.1.fid
/LPIK AF-173.13
187.97
161.42
149.99
148.89
133.43
122.28
77.41
77.16
76.91
20.32
14.26
f1 (мд)

Display Report

Analysis Info

Analysis Name	D:\Data\Kolotyrkina\2014\Bastrakov\0415017.d	Acquisition Date	15.04.2014 16:15:34
Method	tune_low.m	Operator	BDAL@DE
Sample Name	/LPIK KA-12	Instrument / Ser#	micrOTOF 10248
Comment	C10H7N3O4 mw 233 in CH3CN calibrant added		

Acquisition Parameter

Source Type	ESI	Ion Polarity	Positive	Set Nebulizer	0.4 Bar
Focus	Not active			Set Dry Heater	180 °C
Scan Begin	50 m/z	Set Capillary	4500 V	Set Dry Gas	4.0 l/min
Scan End	3000 m/z	Set End Plate Offset	-500 V	Set Divert Valve	Waste

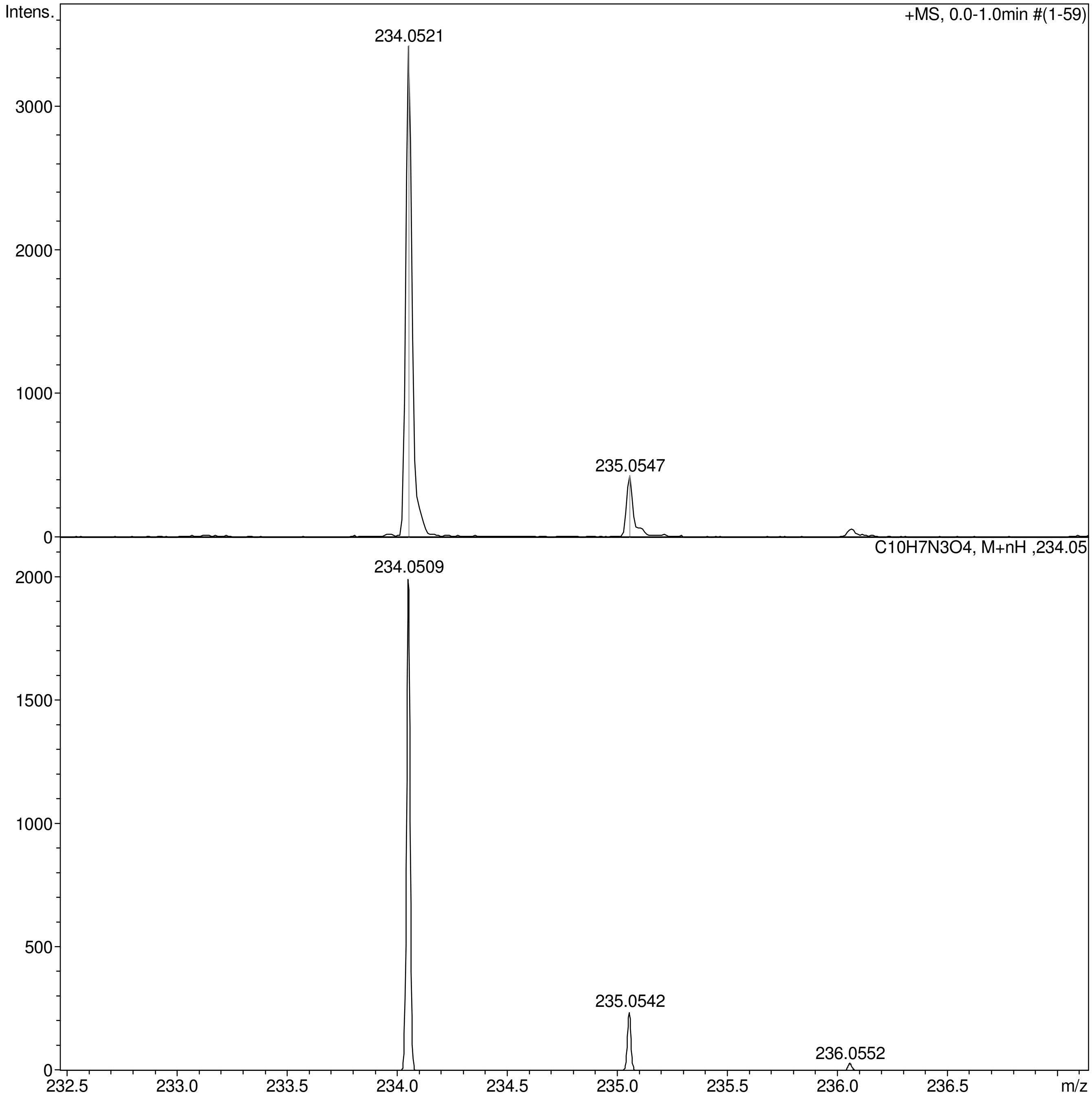

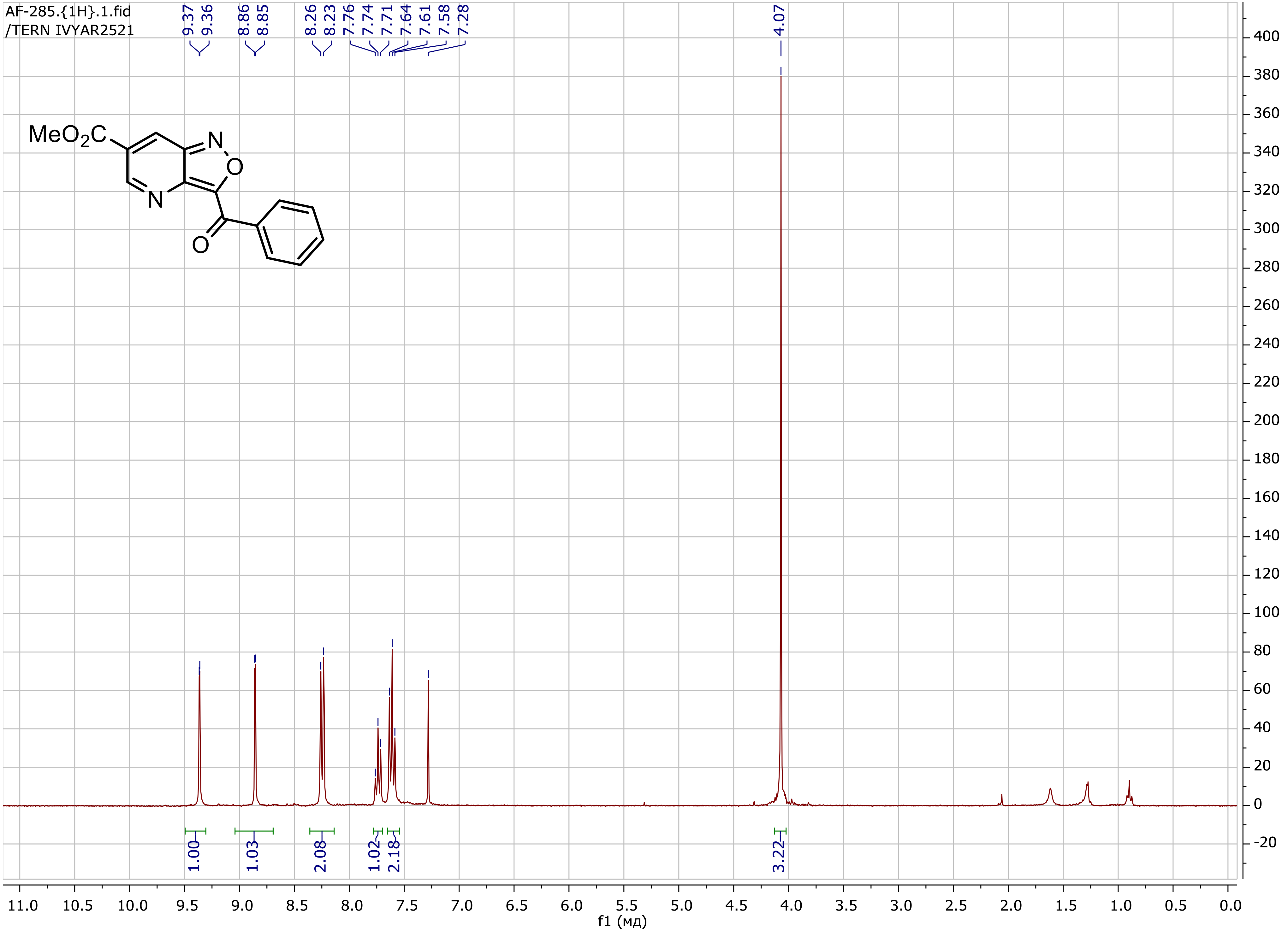
AF-285.{1H}.1.fid
/TERN IVYAR2521
9.37
9.36
8.86
8.85
8.26
8.23
7.76
7.74
7.71
7.64
7.61
7.58
7.28
4.07
MeO2C
N
O
N
O
1.00
1.03
2.08
1.02
2.18
3.22
f1 (мд)

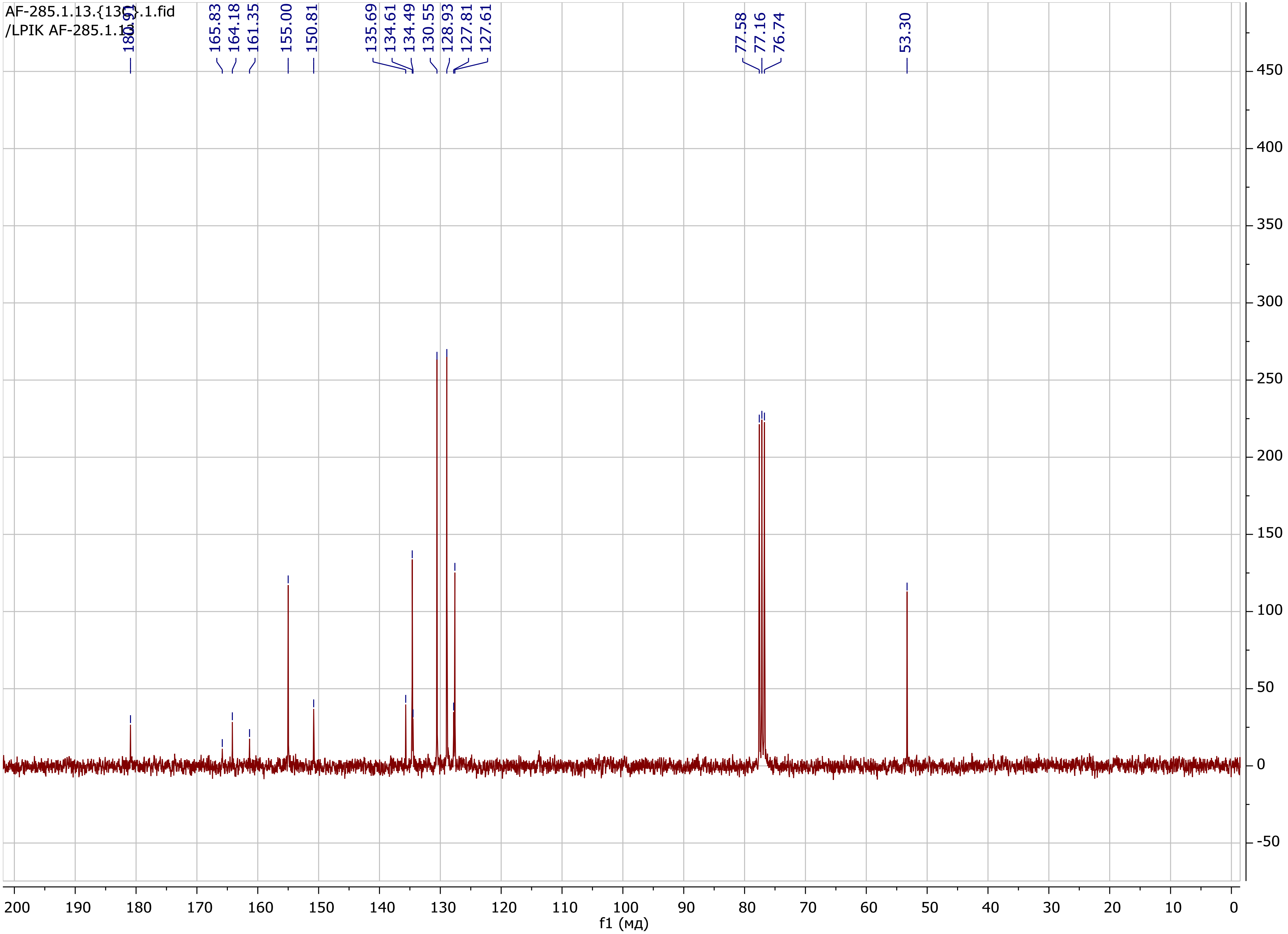
AF-285.1.13.{13C}.1.fid
/LPIK AF-285.1.13
180.91
165.83
164.18
161.35
155.00
150.81
135.69
134.61
134.49
130.55
128.93
127.81
127.61
77.58
77.16
76.74
53.30
f1 (мд)

Display Report

Analysis Info

Analysis Name	D:\Data\Kolotyrkina\2019\Bastrakov\0423025.d	Acquisition Date	23.04.2019 12:50:10
Method	tune_50-1600.m	Operator	BDAL@DE
Sample Name	/LPIK AF-285	Instrument / Ser#	micrOTOF 10248
Comment	C15H10N2O4 mH 283.0713 calibrant added		

Acquisition Parameter

Source Type	ESI	Ion Polarity	Positive	Set Nebulizer	1.0 Bar
Focus	Not active			Set Dry Heater	200 °C
Scan Begin	50 m/z	Set Capillary	4500 V	Set Dry Gas	4.0 l/min
Scan End	1600 m/z	Set End Plate Offset	-500 V	Set Divert Valve	Waste

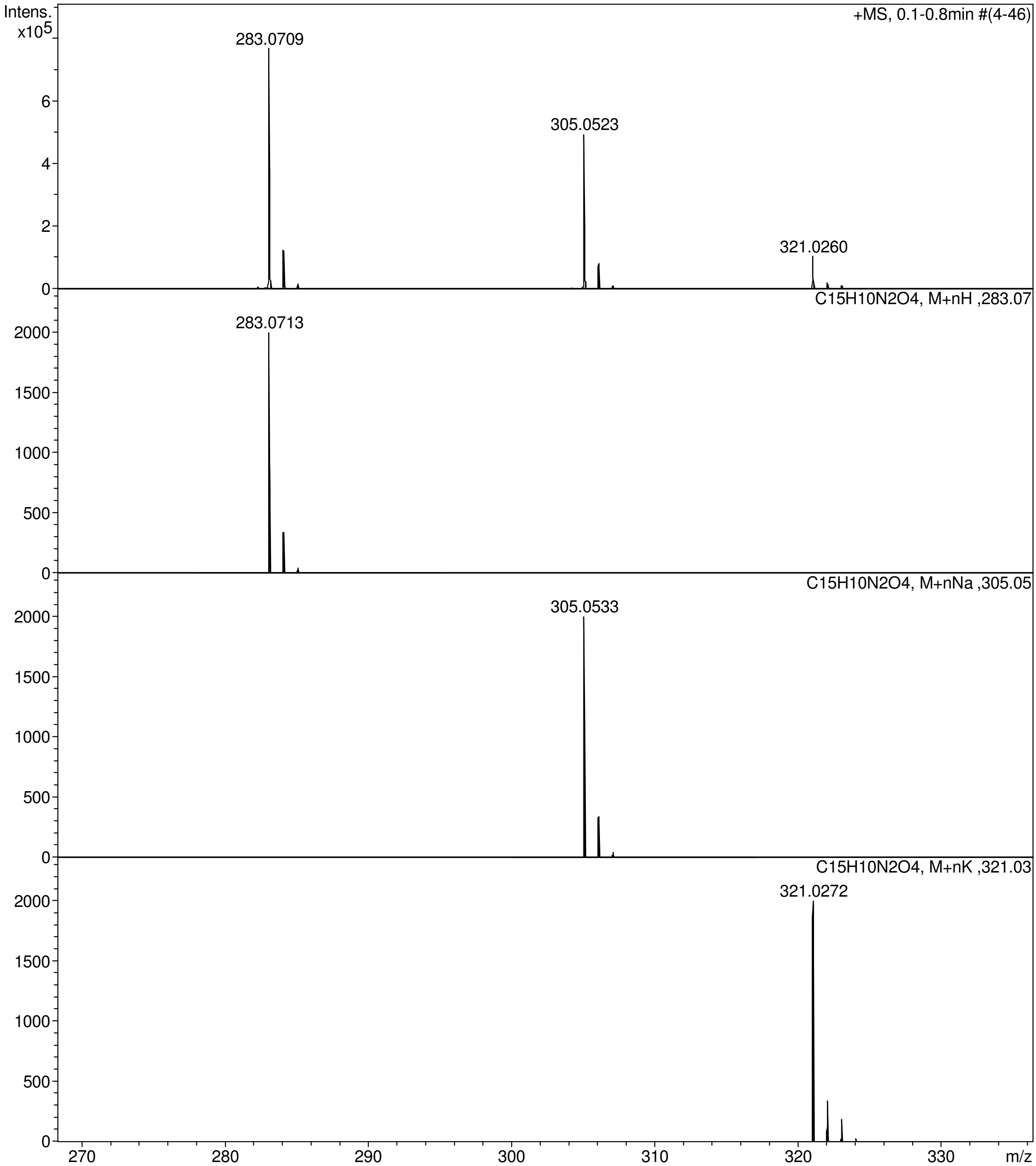

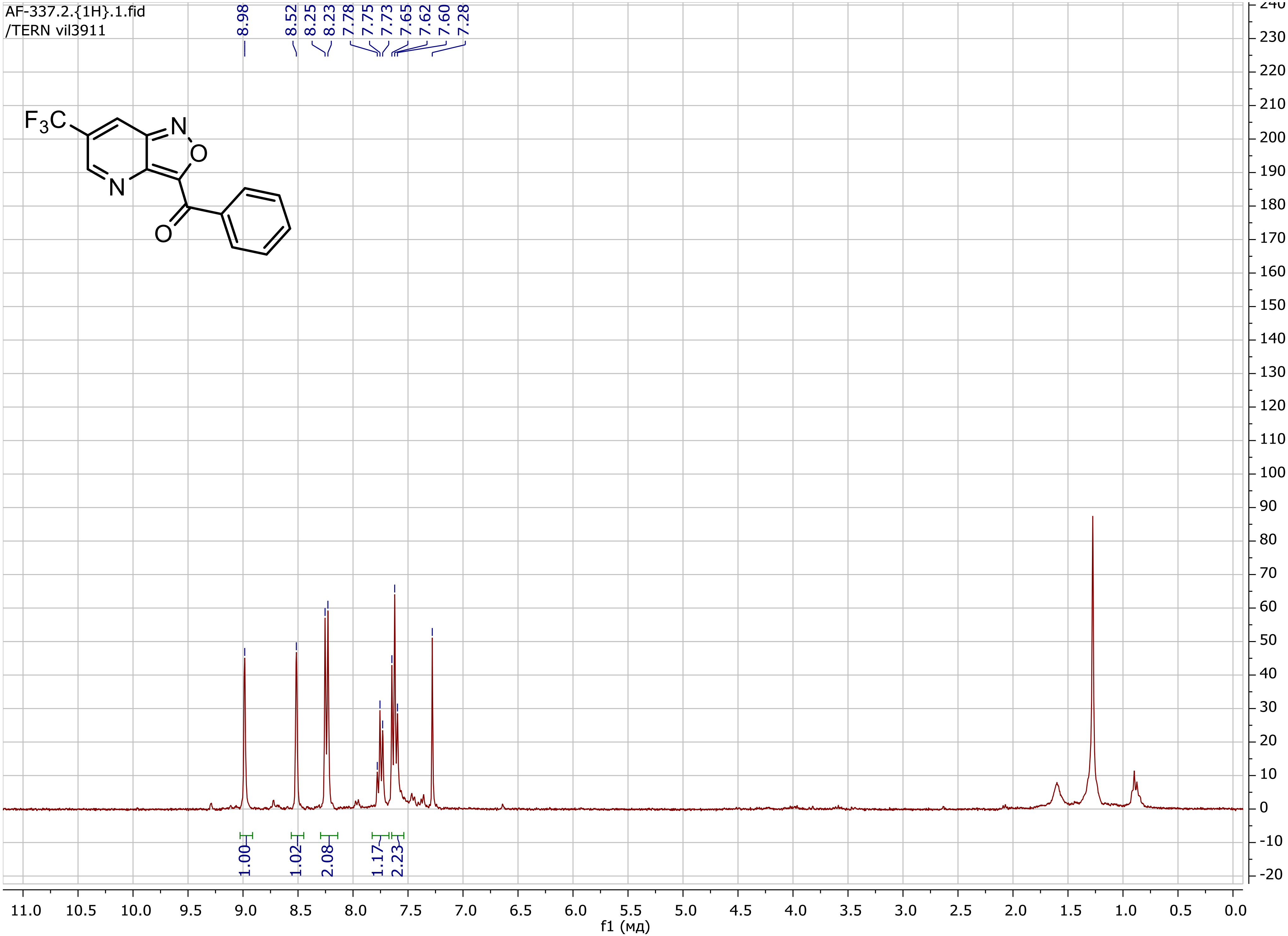
AF-337.2.{1H}.1.fid
/TERN vil3911
8.98
8.52
8.25
8.23
7.78
7.75
7.73
7.65
7.62
7.60
7.28
F3C
N
O
N
O
1.00
1.02
2.08
1.17
2.23
f1 (мд)

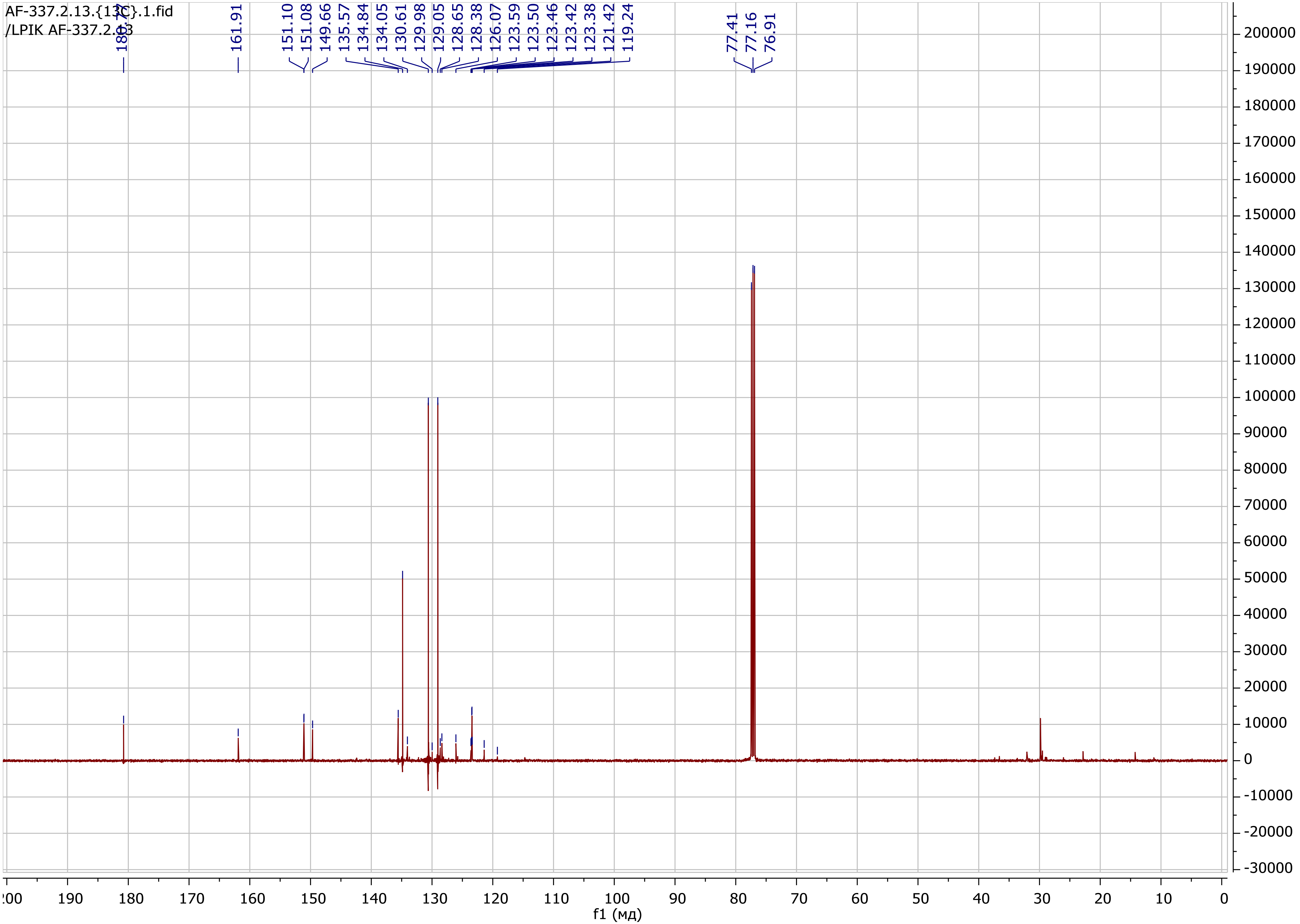
AF-337.2.13.{13C}.1.fid
/LPIK AF-337.2.13
180.77
161.91
151.10
151.08
149.66
135.57
134.84
134.05
130.61
129.98
129.05
128.65
128.38
126.07
123.59
123.50
123.46
123.42
123.38
121.42
119.24
77.41
77.16
76.91
f1 (мд)

Display Report

Analysis Info

Analysis Name	D:\Data\Kolotyrkina\2019\Bastrakov\1127009.d	Acquisition Date	27.11.2019 15:40:50
Method	tune_50-1600.m	Operator	BDAL@DE
Sample Name	/LPIK AF-337.2	Instrument / Ser#	micrOTOF 10248
Comment	C14H7F3N2O2 mH 293.0532 calibrant added		

Acquisition Parameter

Source Type	ESI	Ion Polarity	Positive	Set Nebulizer	1.0 Bar
Focus	Not active			Set Dry Heater	200 °C
Scan Begin	50 m/z	Set Capillary	4500 V	Set Dry Gas	4.0 l/min
Scan End	1600 m/z	Set End Plate Offset	-500 V	Set Divert Valve	Waste

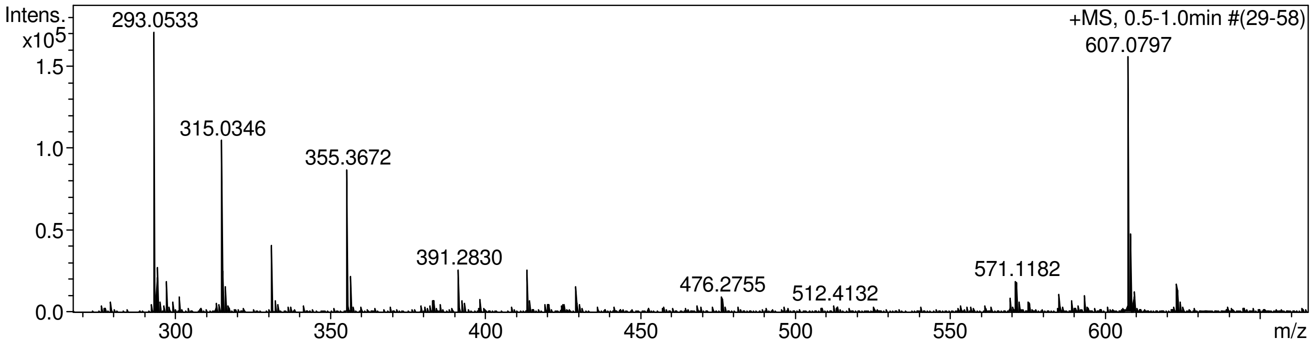

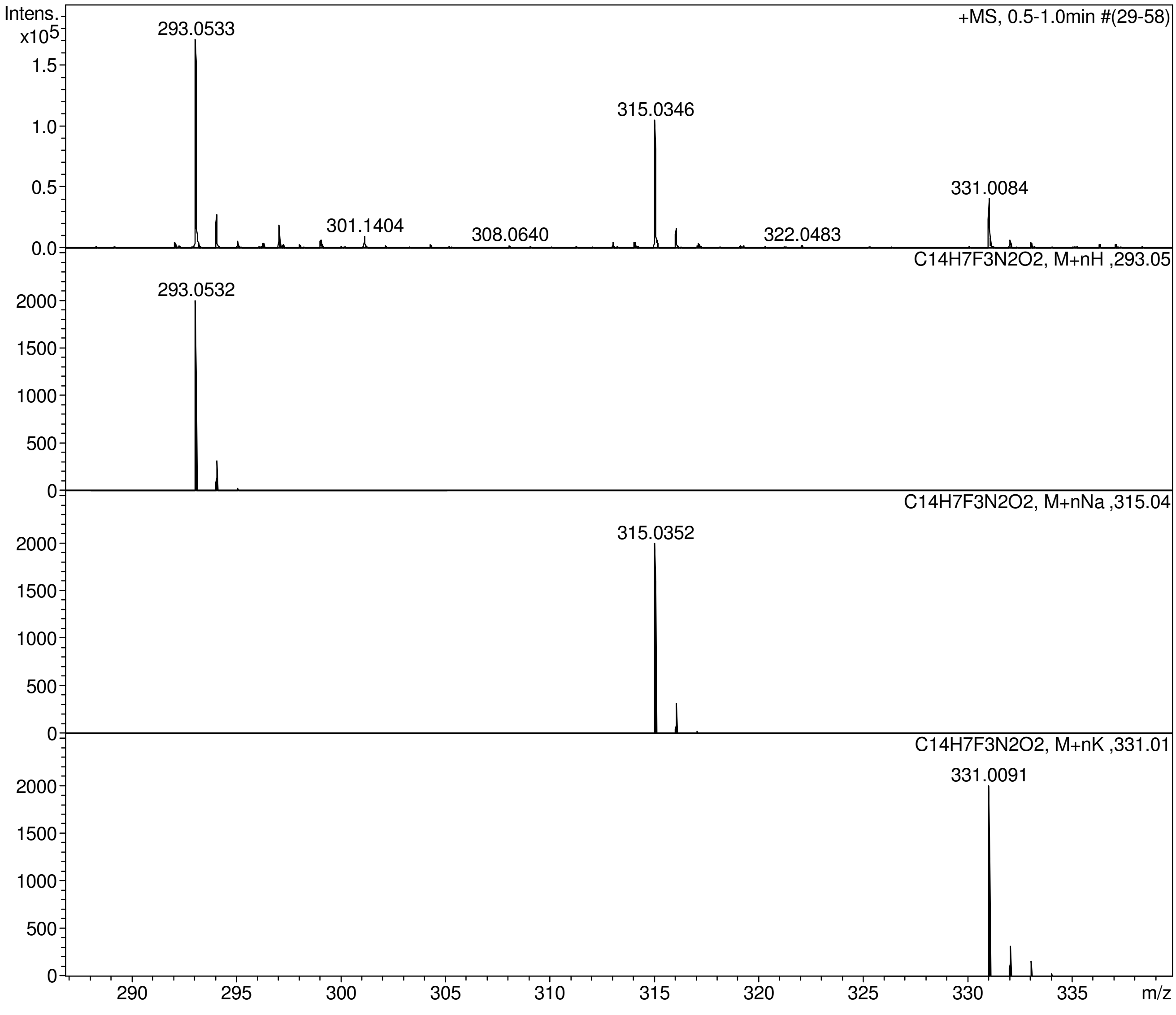

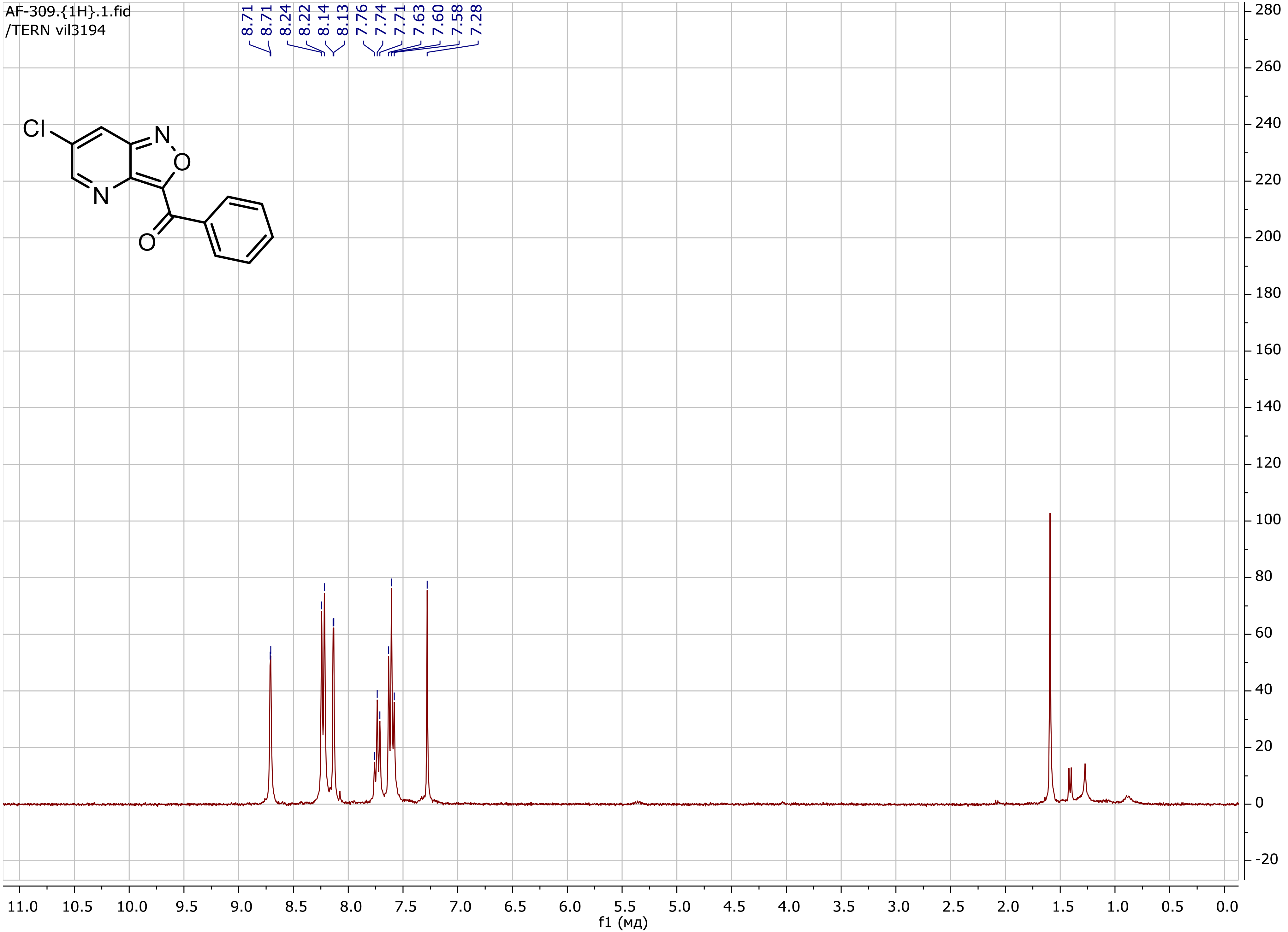
AF-309.{1H}.1.fid
/TERN vil3194
8.71
8.71
8.24
8.22
8.14
8.13
7.76
7.74
7.71
7.63
7.60
7.58
7.28
Cl
N
O
N
O
f1 (мд)

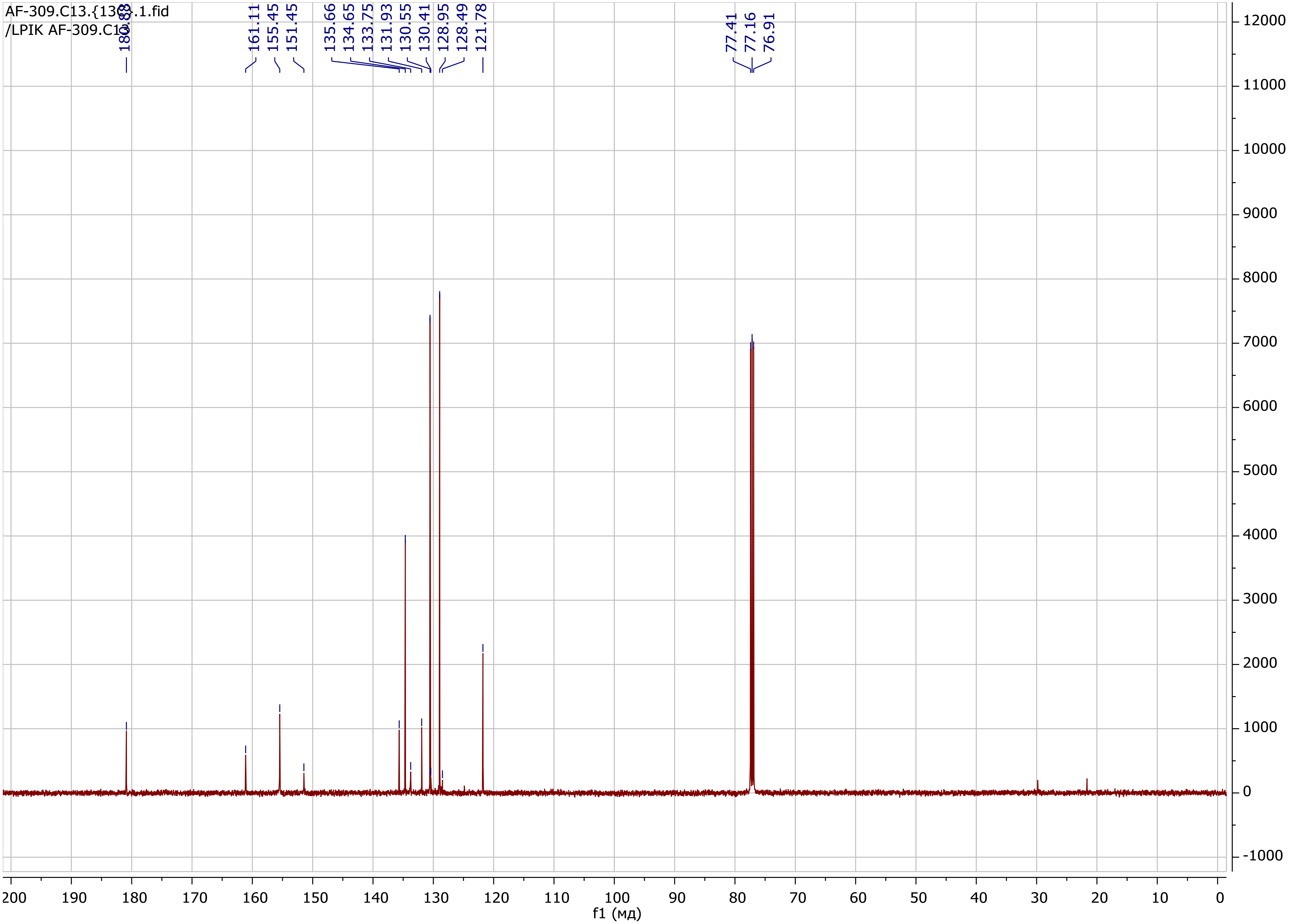

AF-309.C13.{13C}.1.fid
/LPIK AF-309.C13
180.88
161.11
155.45
151.45
135.66
134.65
133.75
131.93
130.55
130.41
128.95
128.49
121.78
77.41
77.16
76.91
12000
11000
10000
9000
8000
7000
6000
5000
4000
3000
2000
1000
0
-1000
200
190
180
170
160
150
140
130
120
110
100
90
80
70
60
50
40
30
20
10
0
f1 (мд)

Display Report

Analysis Info

Analysis Name	D:\Data\Kolotyrkina\2019\Bastrakov\0925006.d	Acquisition Date	25.09.2019 12:43:56
Method	tune_50-1600.m	Operator	BDAL@DE
Sample Name	/LPIK AF-309	Instrument / Ser#	micrOTOF 10248
Comment	C13H7ClN2O2 mH 259.0268 calibrant added		

Acquisition Parameter

Source Type	ESI	Ion Polarity	Positive	Set Nebulizer	1.0 Bar
Focus	Not active			Set Dry Heater	200 °C
Scan Begin	50 m/z	Set Capillary	4500 V	Set Dry Gas	4.0 l/min
Scan End	1600 m/z	Set End Plate Offset	-500 V	Set Divert Valve	Waste

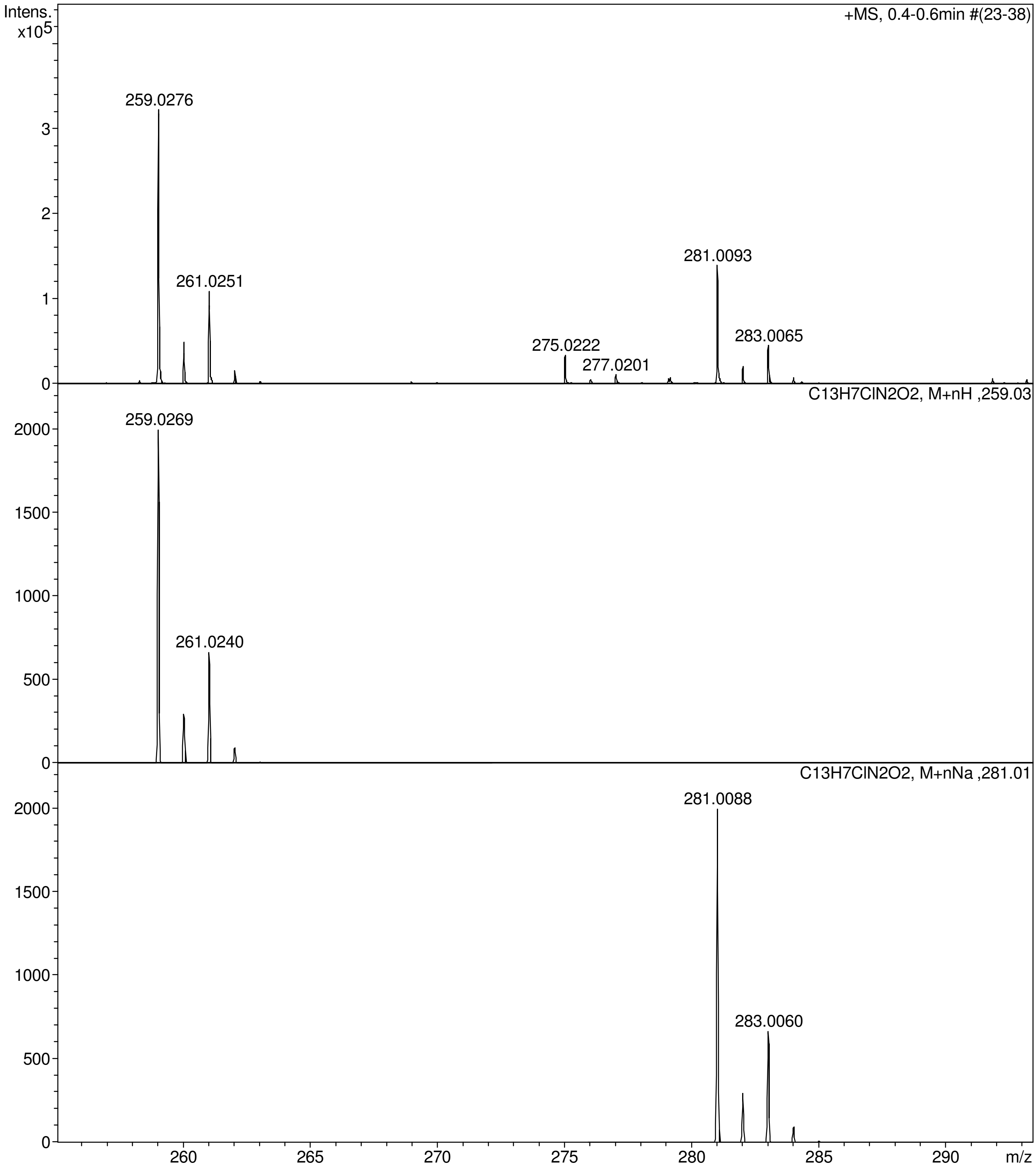

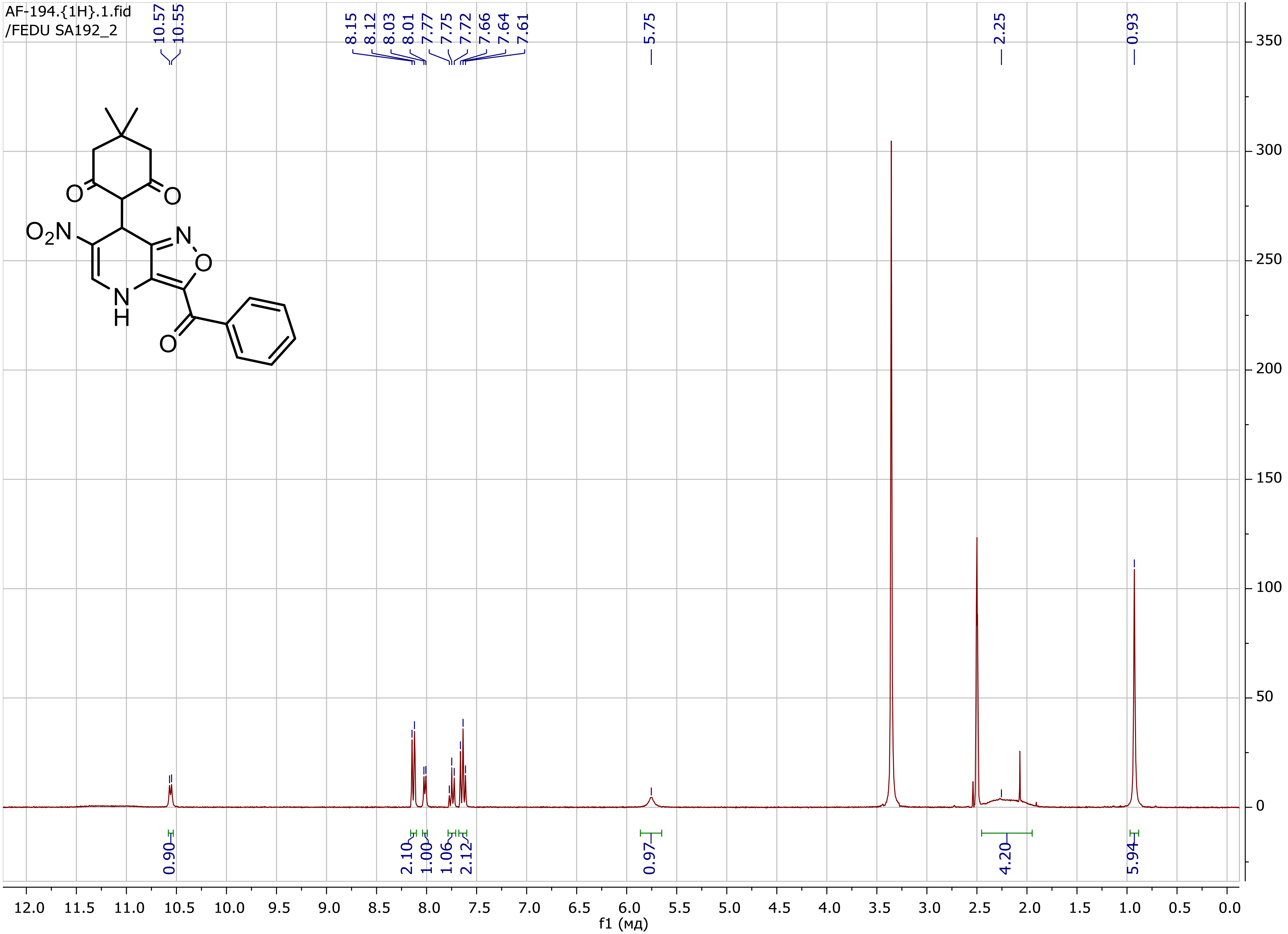
AF-194.{1H}.1.fid
/FEDU SA192_2
10.57
10.55
8.15
8.12
8.03
8.01
7.77
7.75
7.72
7.66
7.64
7.61
5.75
2.25
0.93
O
O
O₂N
N
O
N
H
O
0.90
2.10
1.00
1.06
2.12
0.97
4.20
5.94
350
300
250
200
150
100
50
0
12.0 11.5 11.0 10.5 10.0 9.5 9.0 8.5 8.0 7.5 7.0 6.5 6.0 5.5 5.0 4.5 4.0 3.5 3.0 2.5 2.0 1.5 1.0 0.5 0.0
f1 (мд)

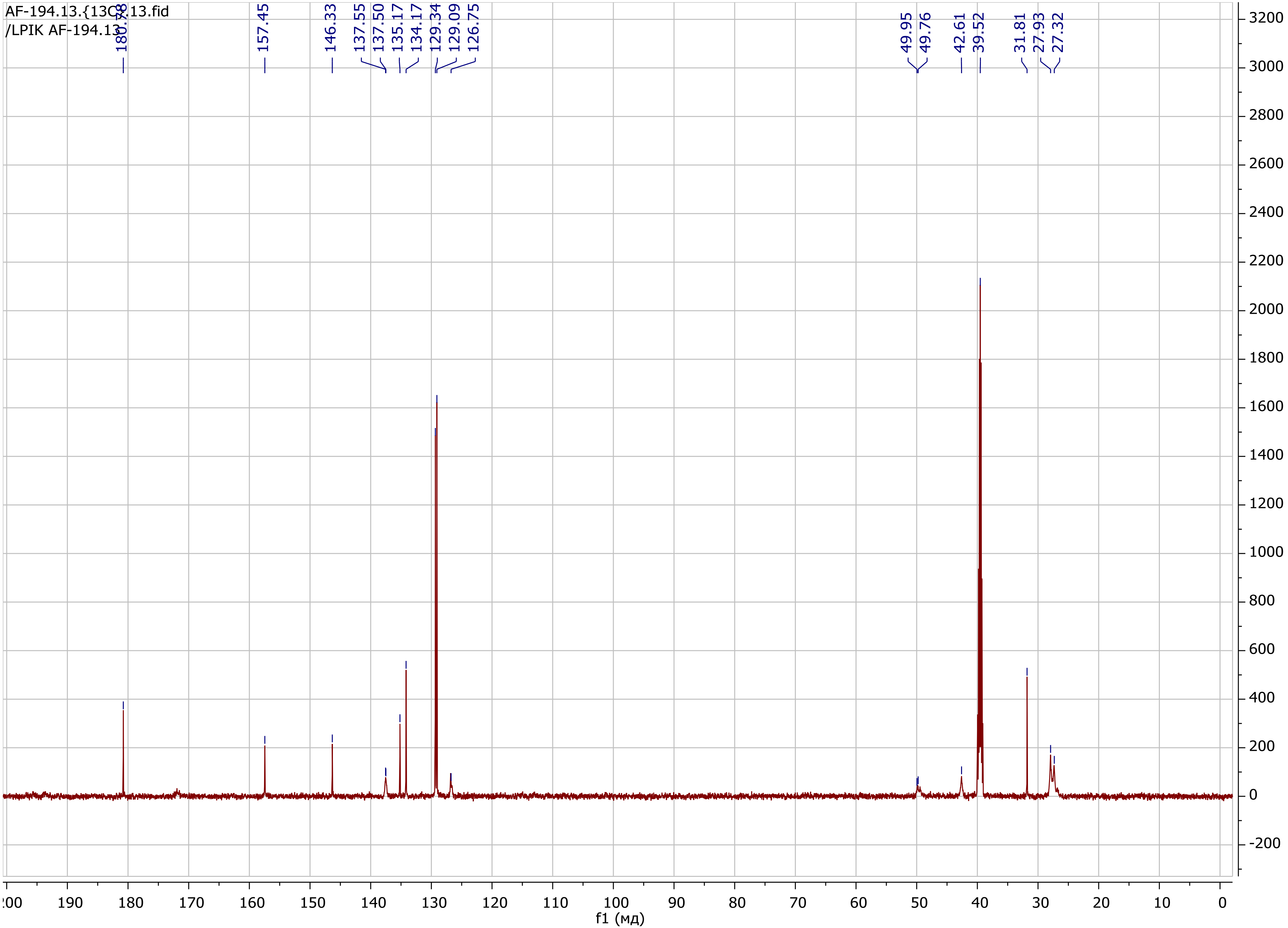
AF-194.13.{13C}.13.fid
/LPIK AF-194.13
180.78
157.45
146.33
137.55
137.50
135.17
134.17
129.34
129.09
126.75
49.95
49.76
42.61
39.52
31.81
27.93
27.32
f1 (мд)

Display Report

Analysis Info

Analysis Name	D:\Data\Kolotyrkina\2018\Bastrakov\1113014.d	Acquisition Date	13.11.2018 11:04:52
Method	tune_50-1600.m	Operator	BDAL@DE
Sample Name	/LPIK AF-194	Instrument / Ser#	micrOTOF 10248
Comment	C21H19N3O6 mH 410.1346 calibrant added		

Acquisition Parameter

Source Type	ESI	Ion Polarity	Positive	Set Nebulizer	1.0 Bar
Focus	Not active			Set Dry Heater	200 °C
Scan Begin	50 m/z	Set Capillary	4500 V	Set Dry Gas	4.0 l/min
Scan End	1600 m/z	Set End Plate Offset	-500 V	Set Divert Valve	Waste

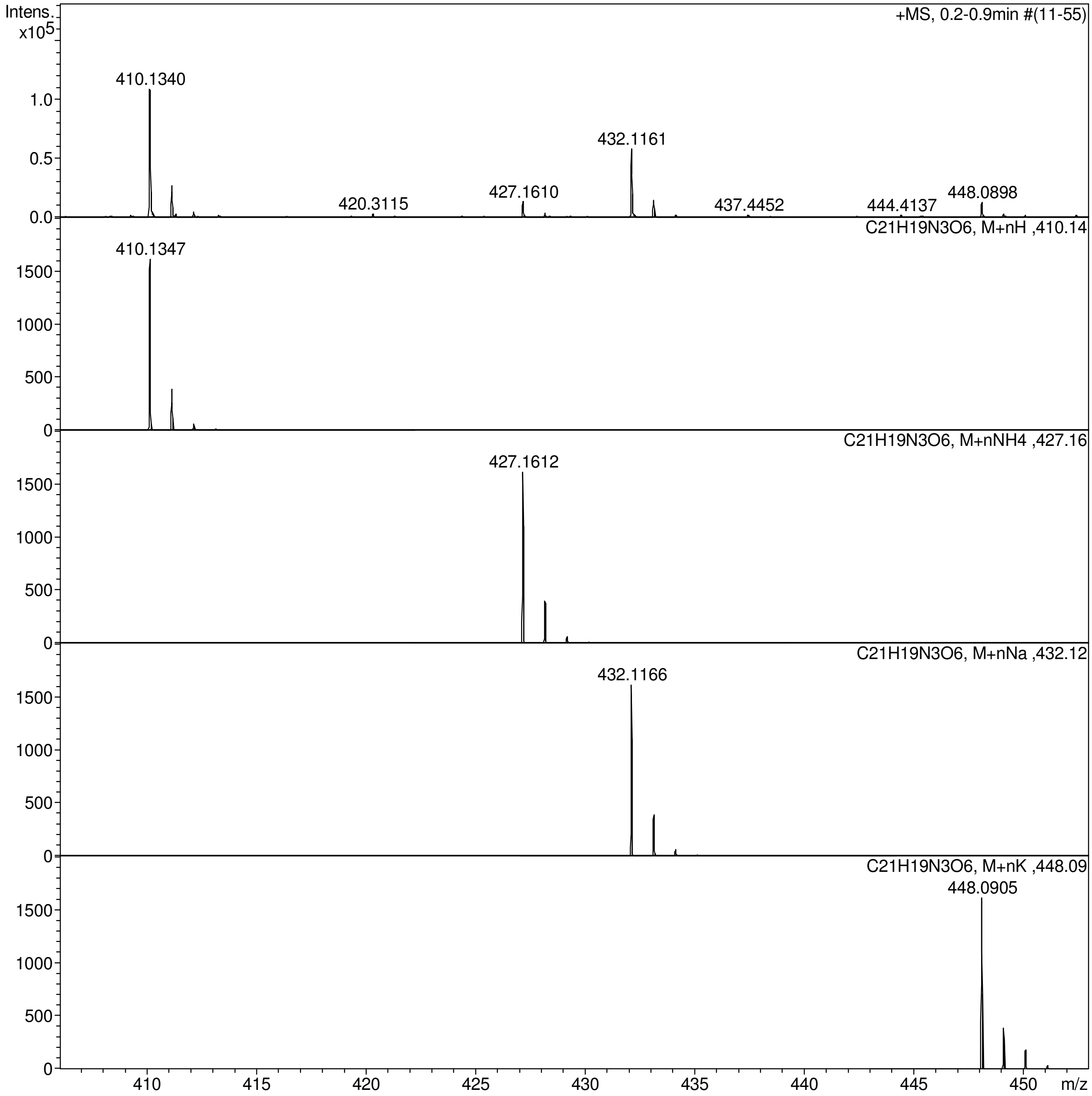

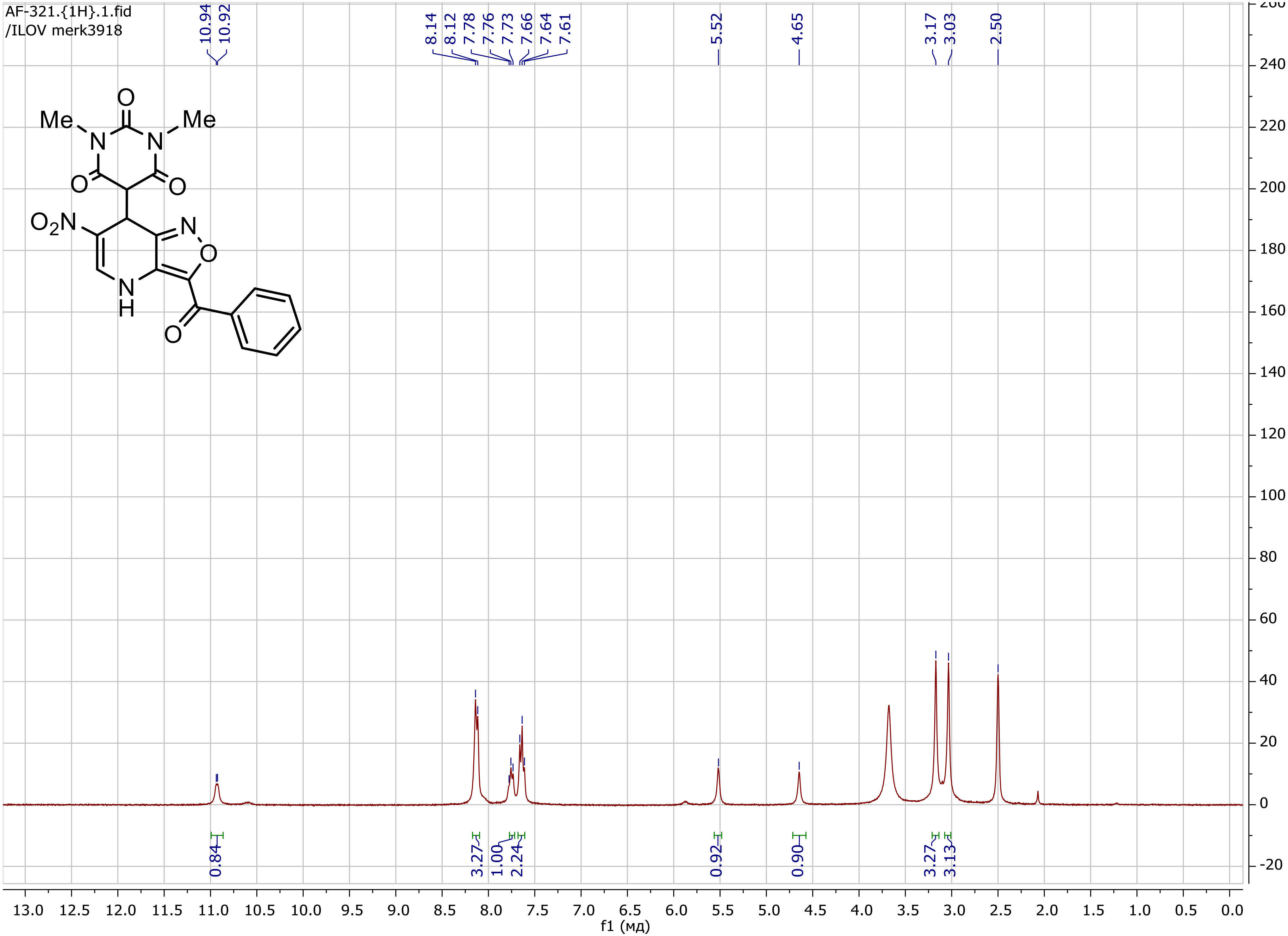

AF-321.{1H}.1.fid
/ILOV merk3918
10.94
10.92
8.14
8.12
7.78
7.76
7.73
7.66
7.64
7.61
5.52
4.65
3.17
3.03
2.50
0.84
3.27
1.00
2.24
0.92
0.90
3.27
3.13
f1 (мд)

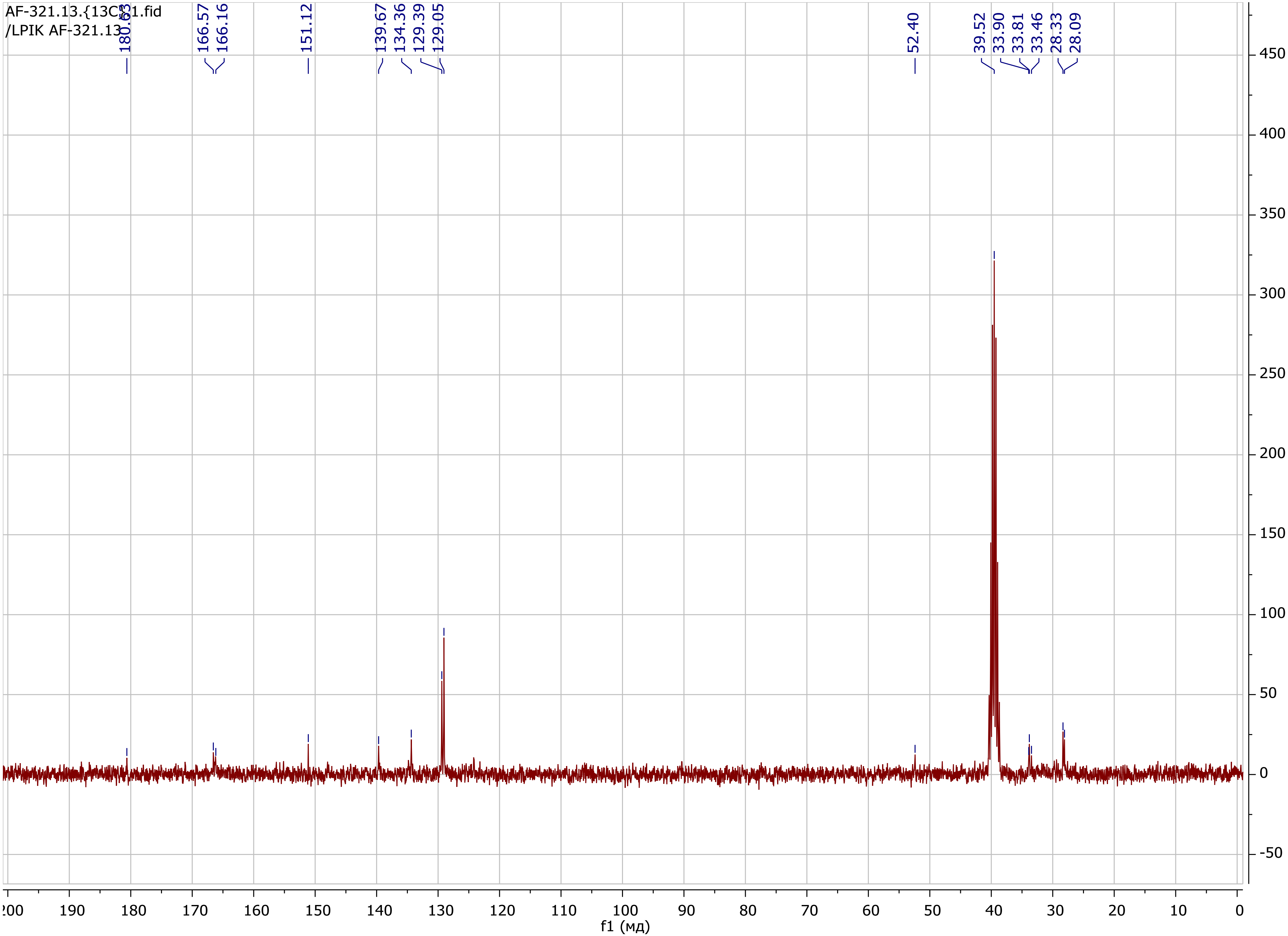
AF-321.13.{13C}1.fid
/LPIK AF-321.13
180.63
166.57
166.16
151.12
139.67
134.36
129.39
129.05
52.40
39.52
33.90
33.81
33.46
28.33
28.09
f1 (мд)

Display Report

Analysis Info

Analysis Name	D:\Data\Kolotyrkina\2019\Bastrakov\0925009.d	Acquisition Date	25.09.2019 13:01:51
Method	tune_50-1600.m	Operator	BDAL@DE
Sample Name	/LPIK AF-321	Instrument / Ser#	micrOTOF 10248
Comment	C19H15N5O7 mH 426.1044 calibrant added		

Acquisition Parameter

Source Type	ESI	Ion Polarity	Positive	Set Nebulizer	1.0 Bar
Focus	Not active			Set Dry Heater	200 °C
Scan Begin	50 m/z	Set Capillary	4500 V	Set Dry Gas	4.0 l/min
Scan End	1600 m/z	Set End Plate Offset	-500 V	Set Divert Valve	Waste

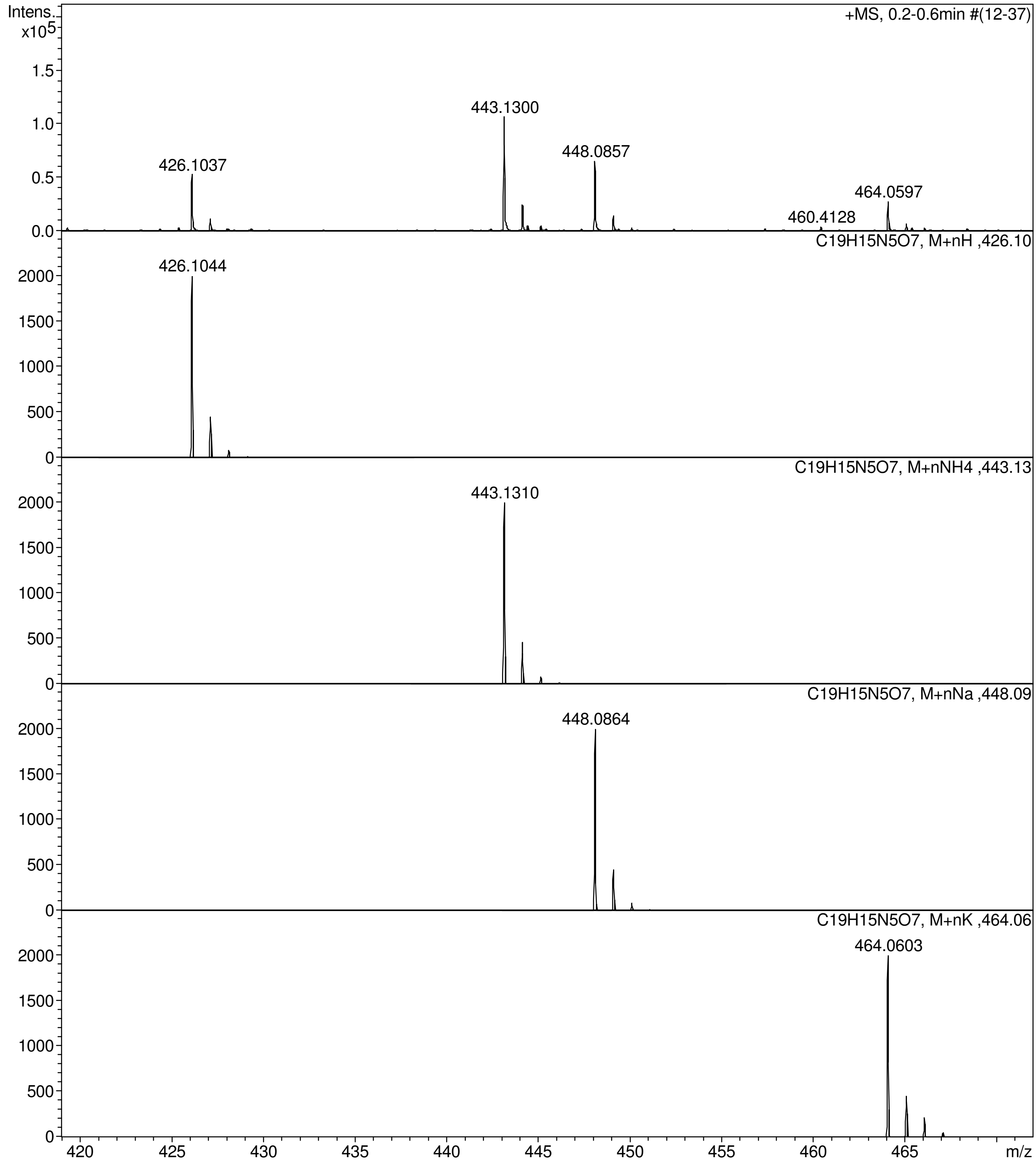

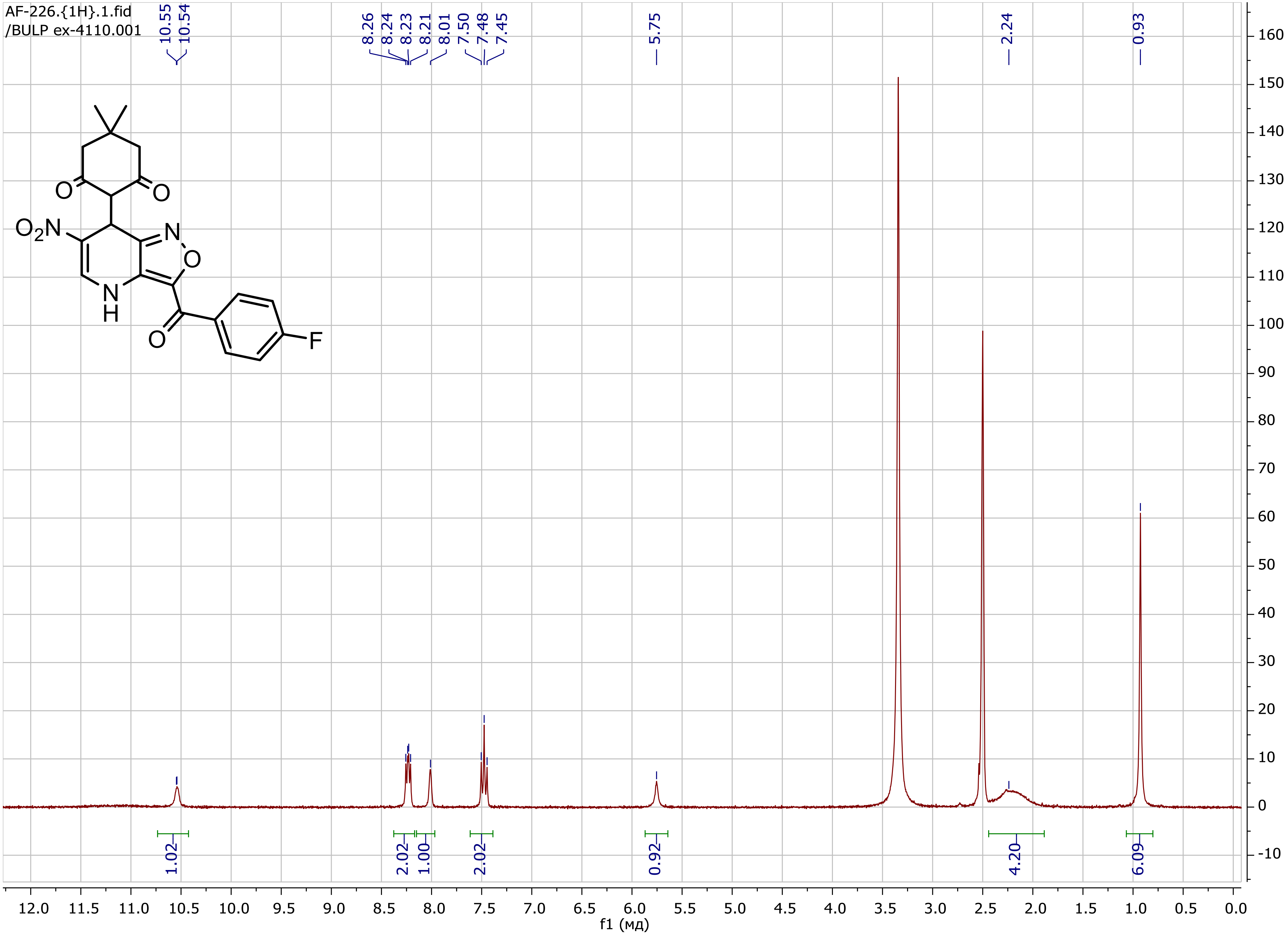
AF-226.{1H}.1.fid
/BULP ex-4110.001
10.55
10.54
8.26
8.24
8.23
8.21
8.01
7.50
7.48
7.45
5.75
2.24
0.93
O
O
O₂N
N
O
N
H
O
F
1.02
2.02
1.00
2.02
0.92
4.20
6.09
f1 (мд)

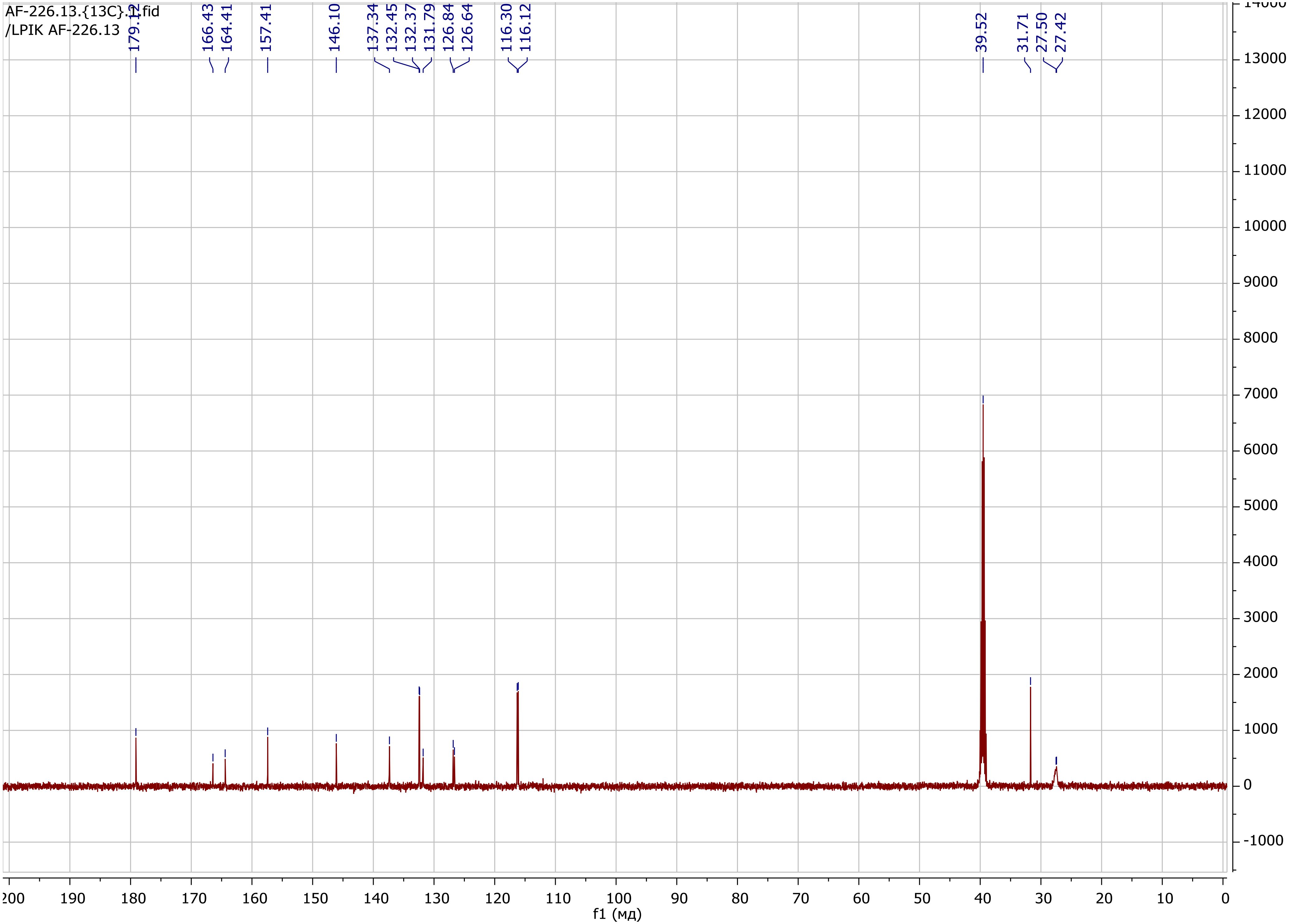
AF-226.13.{13C}.1.fid
/LPIK AF-226.13
179.11
166.43
164.41
157.41
146.10
137.34
132.45
132.37
131.79
126.84
126.64
116.30
116.12
39.52
31.71
27.50
27.42
f1 (мд)

Display Report

Analysis Info

Analysis Name	D:\Data\Kolotyrkina\2019\Bastrakov\0326040.d	Acquisition Date	26.03.2019 19:03:42
Method	tune_50-1600.m	Operator	BDAL@DE
Sample Name	/LPIK AF-226	Instrument / Ser#	micrOTOF 10248
Comment	C21H18FN3O6 mH 428.1252 calibrant added CH3CN		

Acquisition Parameter

Source Type	ESI	Ion Polarity	Positive	Set Nebulizer	1.0 Bar
Focus	Not active			Set Dry Heater	200 °C
Scan Begin	50 m/z	Set Capillary	4500 V	Set Dry Gas	4.0 l/min
Scan End	1600 m/z	Set End Plate Offset	-500 V	Set Divert Valve	Waste

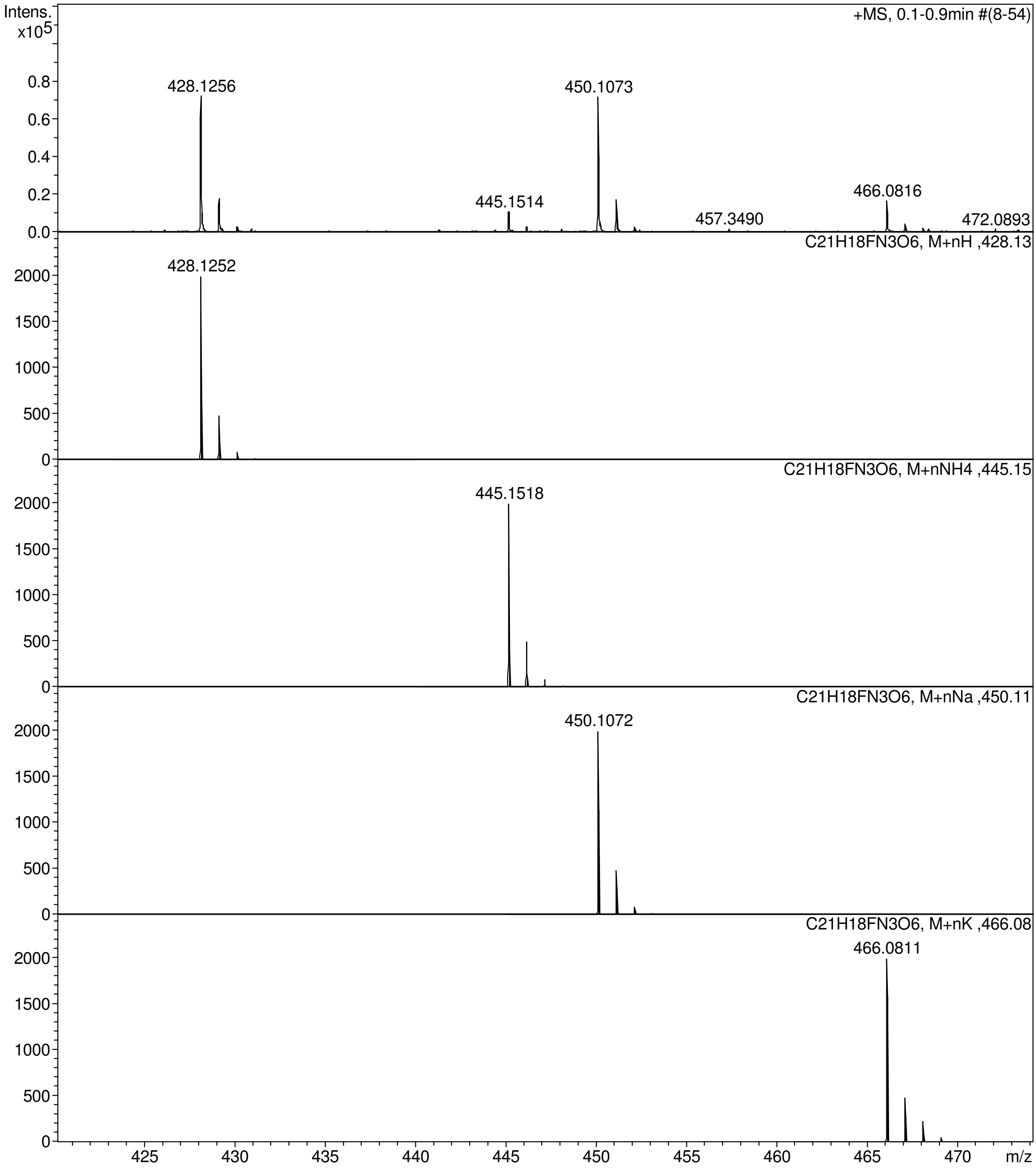

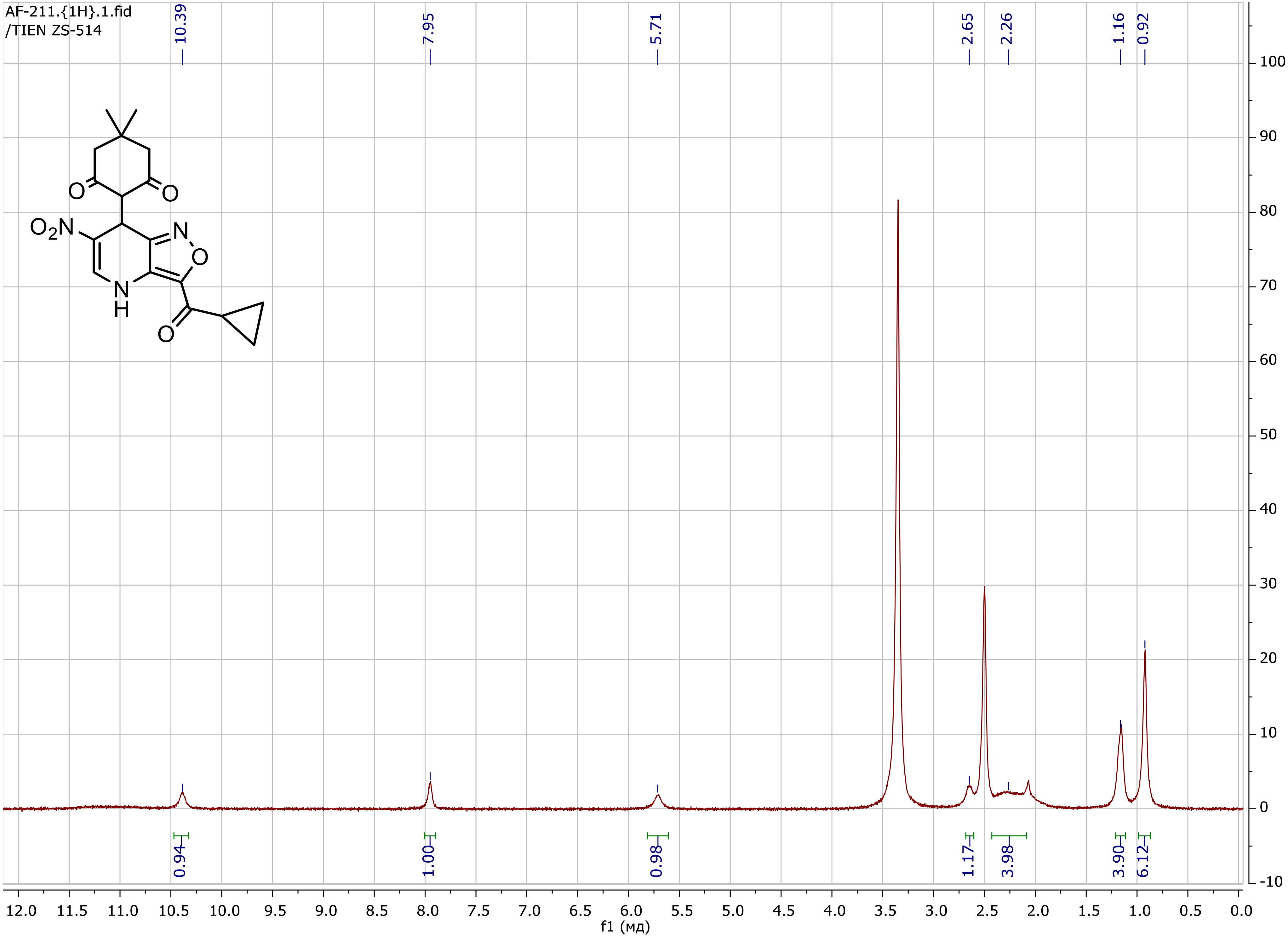

AF-211.{1H}.1.fid
/TIEN ZS-514
10.39
7.95
5.71
2.65
2.26
1.16
0.92
O_2N
N
O
N
H
0.94
1.00
0.98
1.17
3.98
3.90
6.12
f1 (мд)

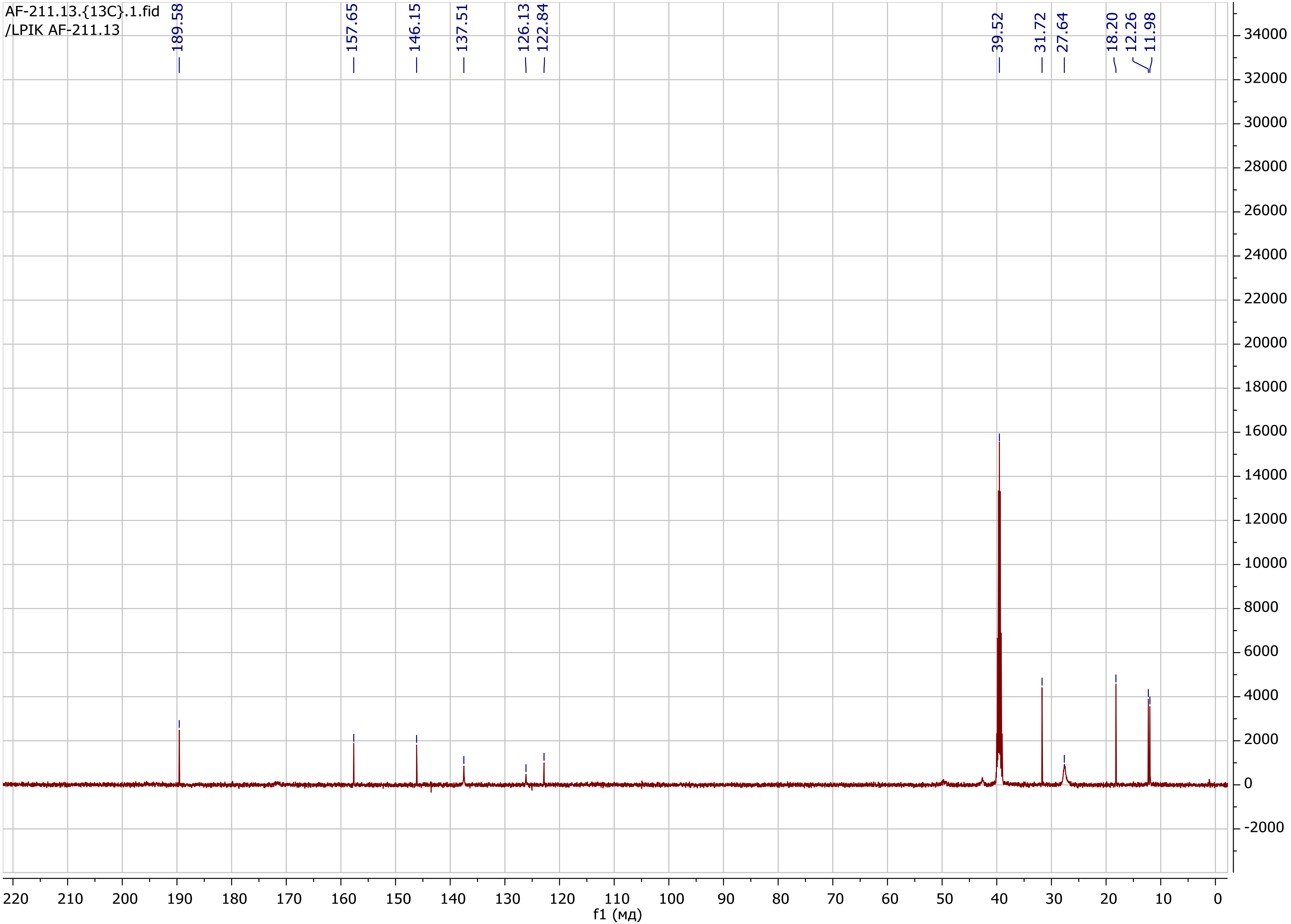

AF-211.13.{13C}.1.fid
/LPIK AF-211.13
189.58
157.65
146.15
137.51
126.13
122.84
39.52
31.72
27.64
18.20
12.26
11.98
f1 (мд)

Display Report

Analysis Info

Analysis Name	D:\Data\Kolotyrkina\2018\Bastrakov\1113015.d	Acquisition Date	13.11.2018 11:09:49
Method	tune_50-1600.m	Operator	BDAL@DE
Sample Name	/LPIK AF-211	Instrument / Ser#	micrOTOF 10248
Comment	C18H19N3O6 mH 374.1346 calibrant added		

Acquisition Parameter

Source Type	ESI	Ion Polarity	Positive	Set Nebulizer	1.0 Bar
Focus	Not active			Set Dry Heater	200 °C
Scan Begin	50 m/z	Set Capillary	4500 V	Set Dry Gas	4.0 l/min
Scan End	1600 m/z	Set End Plate Offset	-500 V	Set Divert Valve	Waste

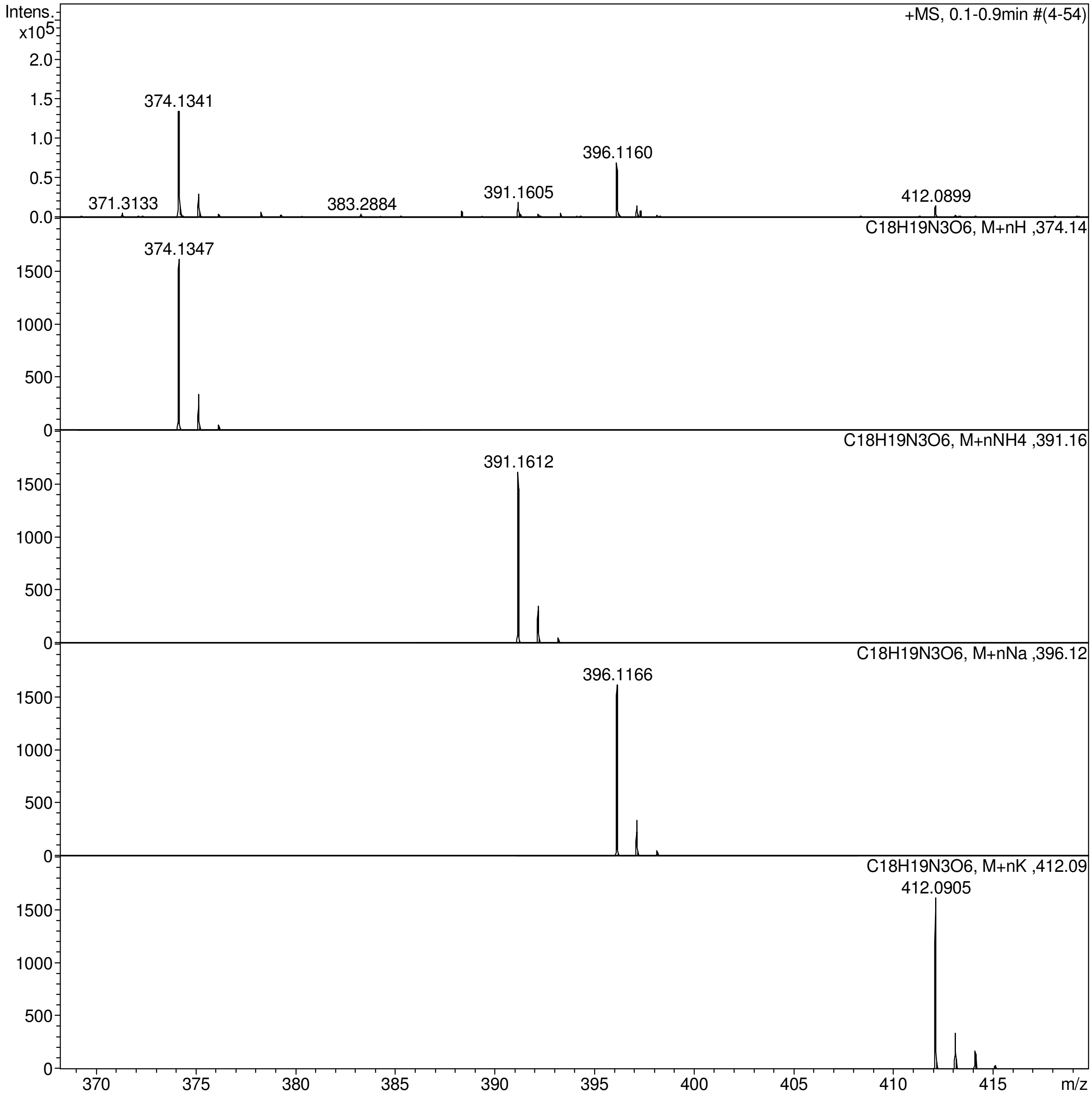

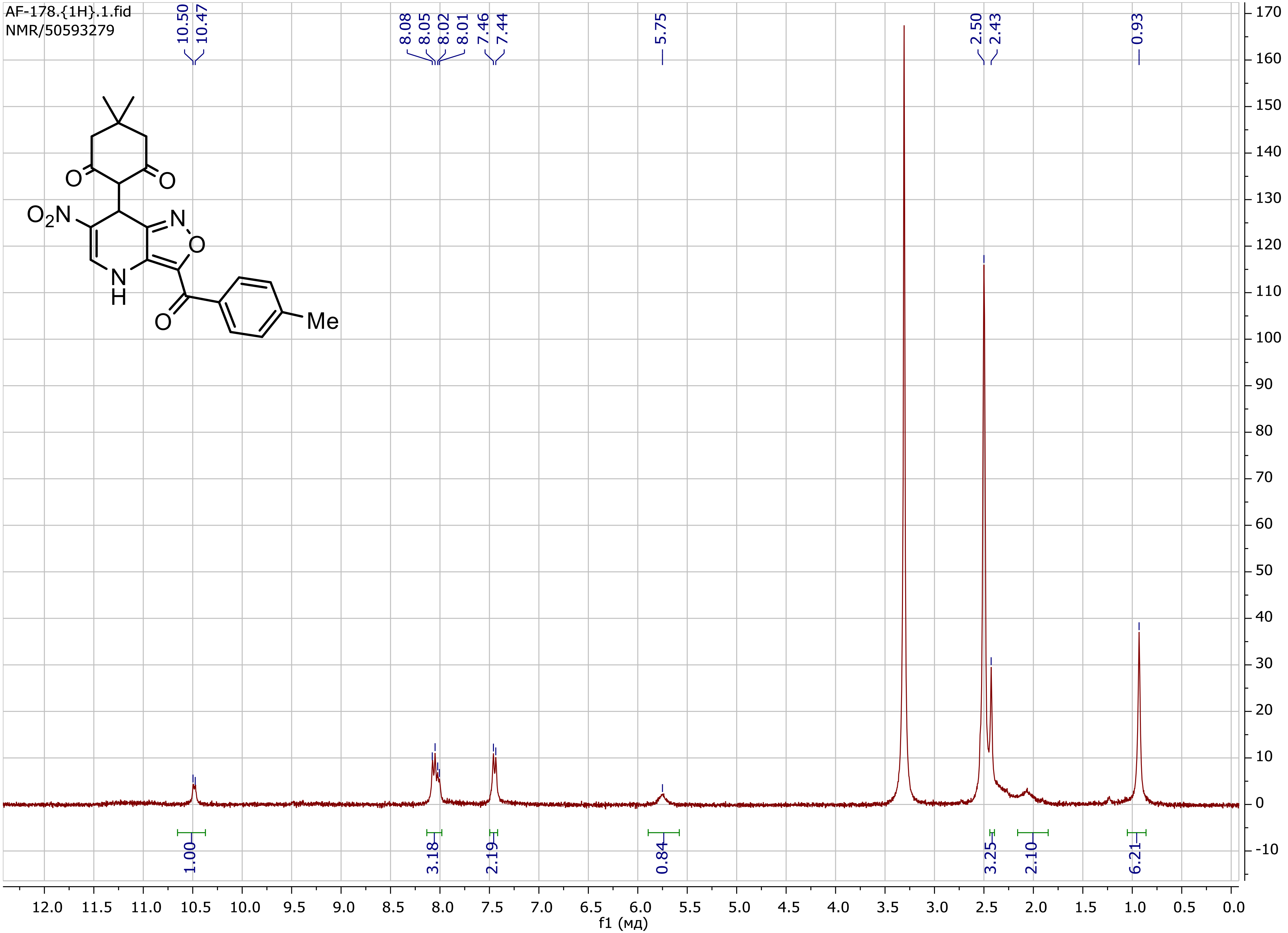

AF-178.{1H}.1.fid
NMR/50593279
10.50
10.47
8.08
8.05
8.02
8.01
7.46
7.44
5.75
2.50
2.43
0.93
O
O
O₂N
N
O
N
H
O
Me
1.00
3.18
2.19
0.84
3.25
2.10
6.21
12.0 11.5 11.0 10.5 10.0 9.5 9.0 8.5 8.0 7.5 7.0 6.5 6.0 5.5 5.0 4.5 4.0 3.5 3.0 2.5 2.0 1.5 1.0 0.5 0.0
f1 (мд)
170 160 150 140 130 120 110 100 90 80 70 60 50 40 30 20 10 0 -10

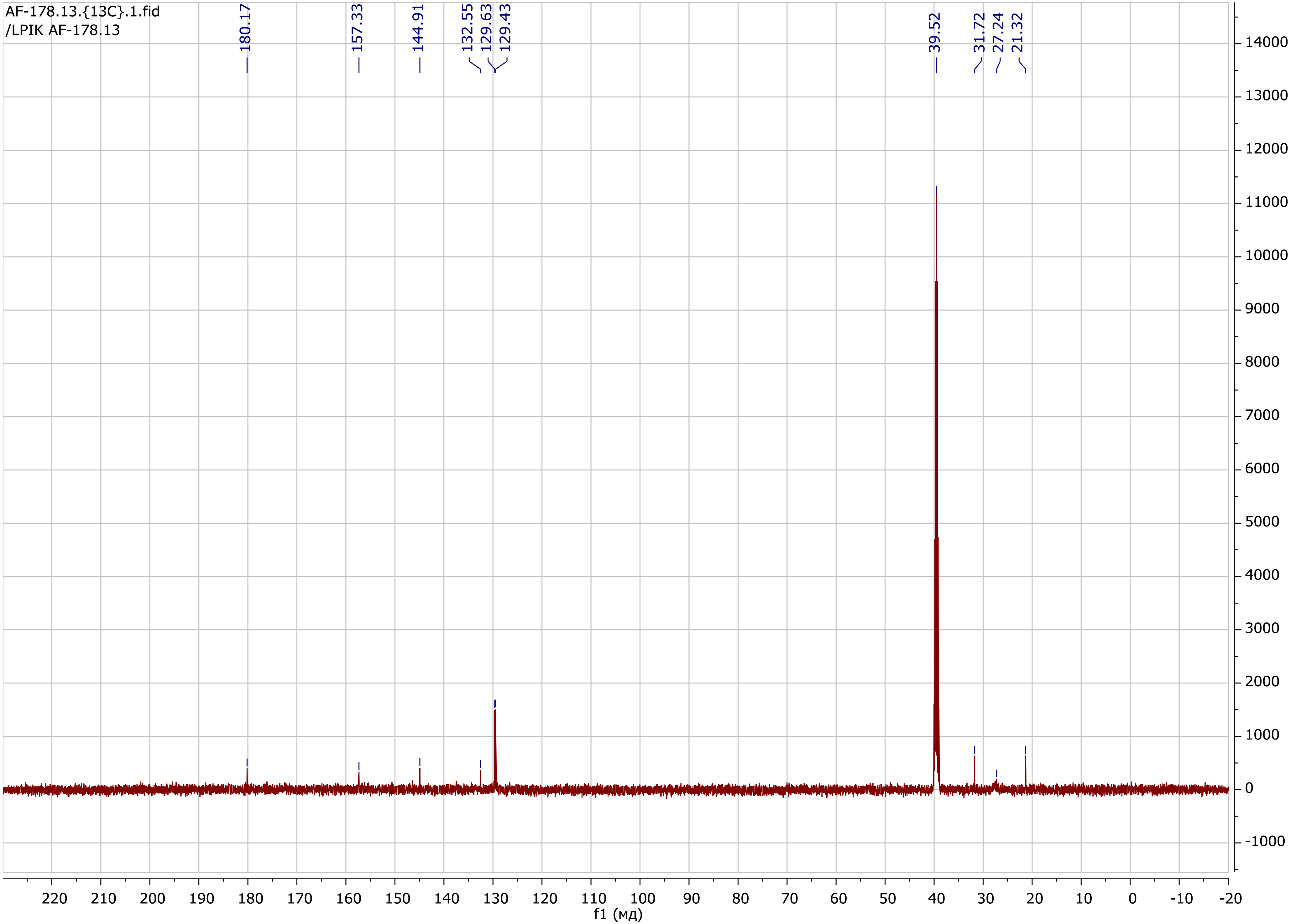
AF-178.13.{13C}.1.fid
/LPIK AF-178.13
180.17
157.33
144.91
132.55
129.63
129.43
39.52
31.72
27.24
21.32
f1 (мд)

Display Report

Analysis Info

Analysis Name	D:\Data\Kolotyrkina\2018\Bastrakov\0703003.d	Acquisition Date	03.07.2018 10:43:38
Method	tune_50-1600.m	Operator	BDAL@DE
Sample Name	/LPIK AF-178	Instrument / Ser#	micrOTOF 10248
Comment	C22H21N3O6 mH 424.1503 calibrant added		

Acquisition Parameter

Source Type	ESI	Ion Polarity	Positive	Set Nebulizer	1.0 Bar
Focus	Not active			Set Dry Heater	200 °C
Scan Begin	50 m/z	Set Capillary	4500 V	Set Dry Gas	4.0 l/min
Scan End	1600 m/z	Set End Plate Offset	-500 V	Set Divert Valve	Waste

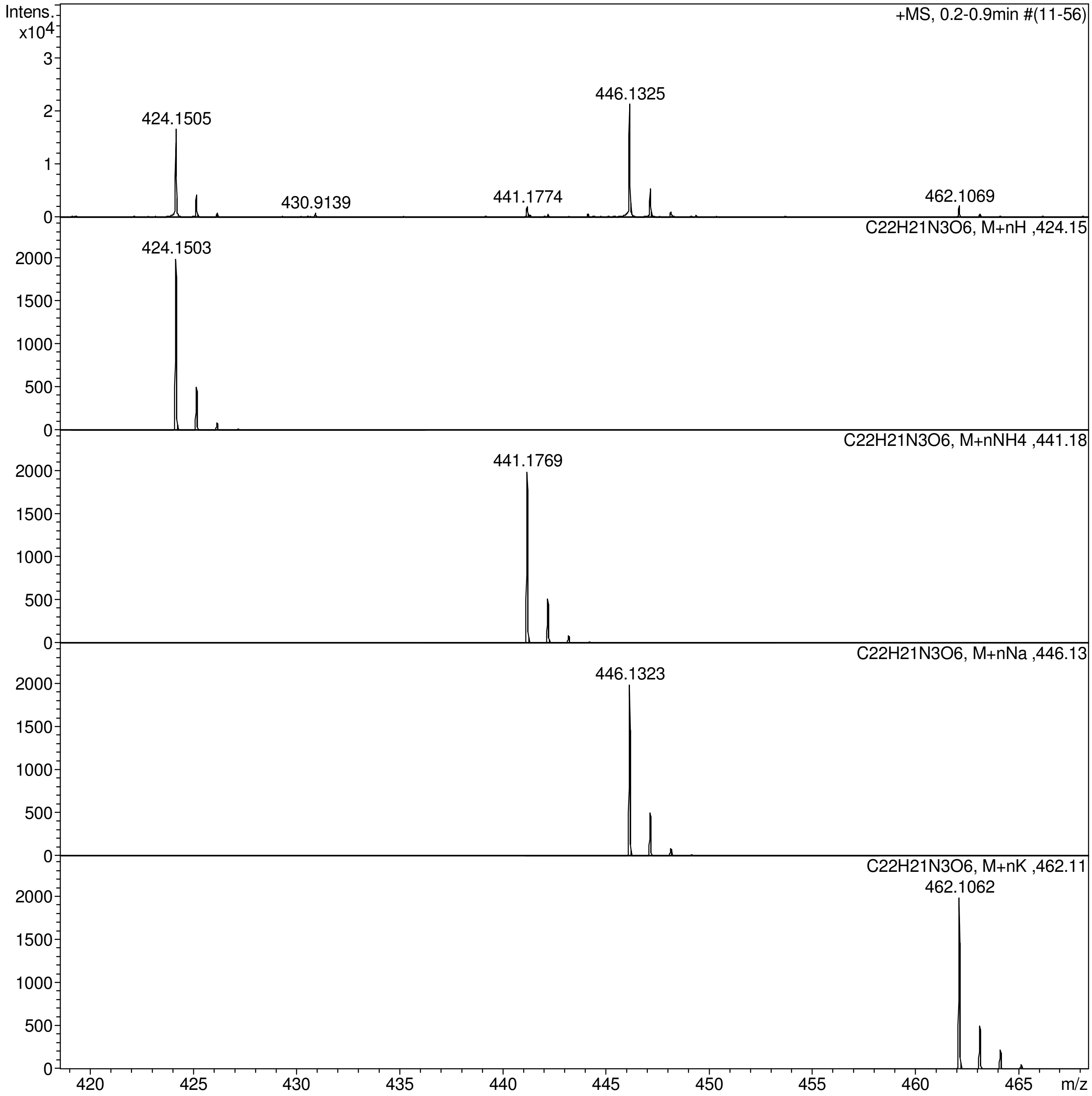

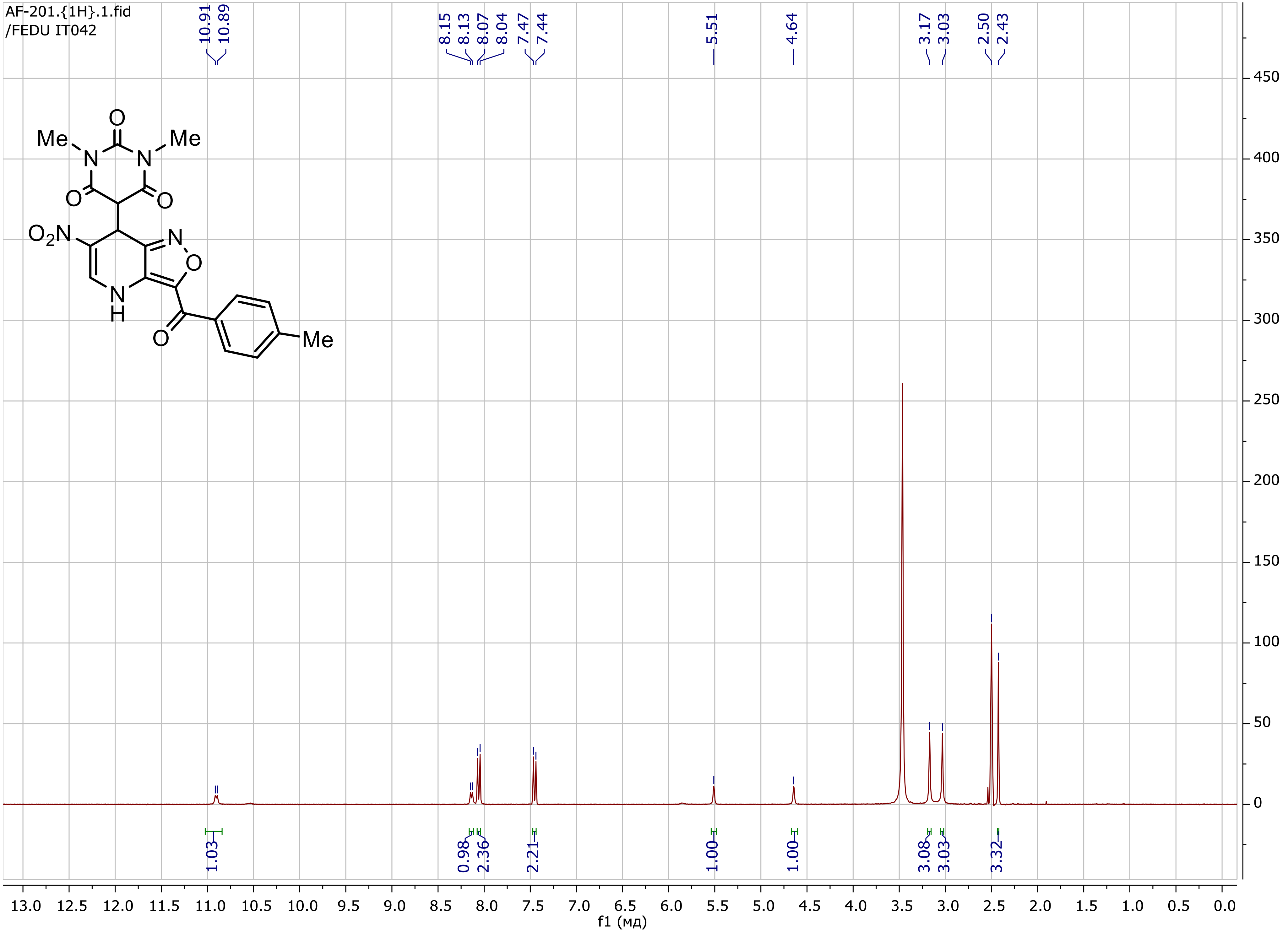

AF-201.{1H}.1.fid
/FEDU IT042
10.91
10.89
8.15
8.13
8.07
8.04
7.47
7.44
5.51
4.64
3.17
3.03
2.50
2.43
1.03
0.98
2.36
2.21
1.00
1.00
3.08
3.03
3.32
Me
Me
O
O
O
O_2N
N
N
N
O
N
H
O
Me
f1 (мд)

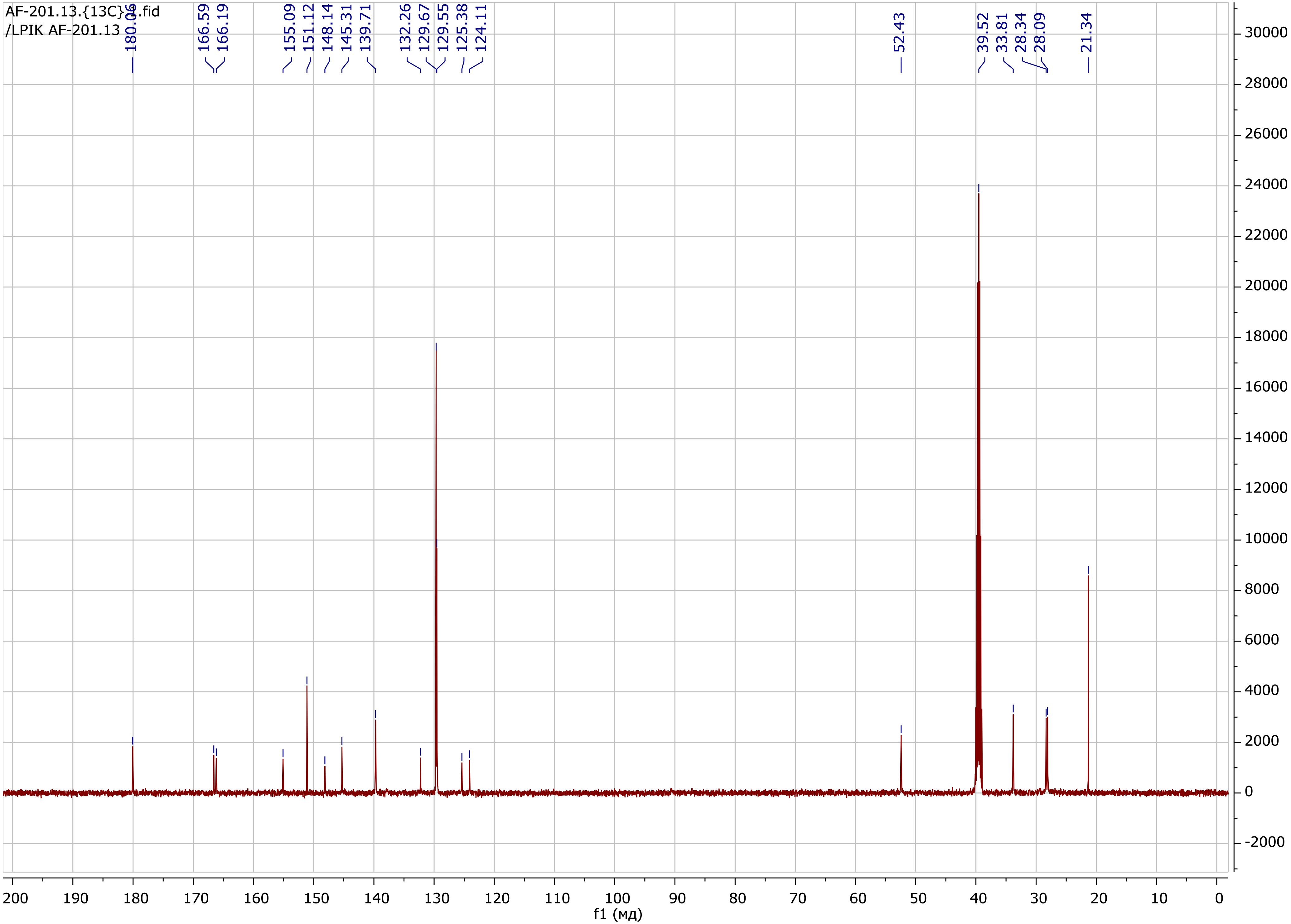
AF-201.13.{13C}1.fid
/LPIK AF-201.13
180.06
166.59
166.19
155.09
151.12
148.14
145.31
139.71
132.26
129.67
129.55
125.38
124.11
52.43
39.52
33.81
28.34
28.09
21.34
f1 (мд)

Display Report

Analysis Info

Analysis Name	D:\Data\Kolotyrkina\2018\Bastrakov\1113025.d	Acquisition Date	13.11.2018 12:52:08
Method	tune_50-1600.m	Operator	BDAL@DE
Sample Name	/LPIK AF-201	Instrument / Ser#	micrOTOF 10248
Comment	C20H17N5O7 mH 440.1201 calibrant added		

Acquisition Parameter

Source Type	ESI	Ion Polarity	Positive	Set Nebulizer	1.0 Bar
Focus	Not active			Set Dry Heater	200 °C
Scan Begin	50 m/z	Set Capillary	4500 V	Set Dry Gas	4.0 l/min
Scan End	1600 m/z	Set End Plate Offset	-500 V	Set Divert Valve	Waste

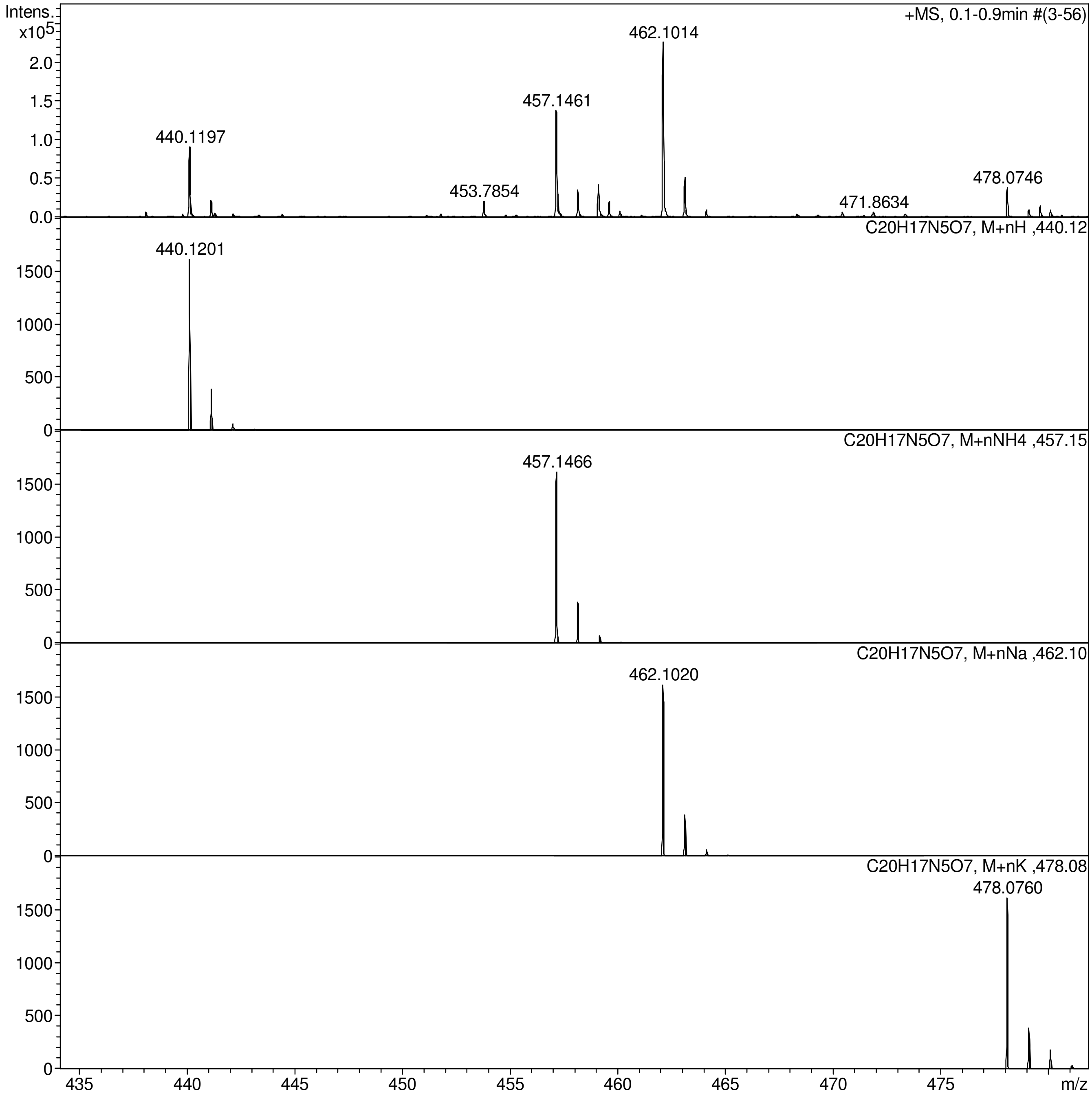

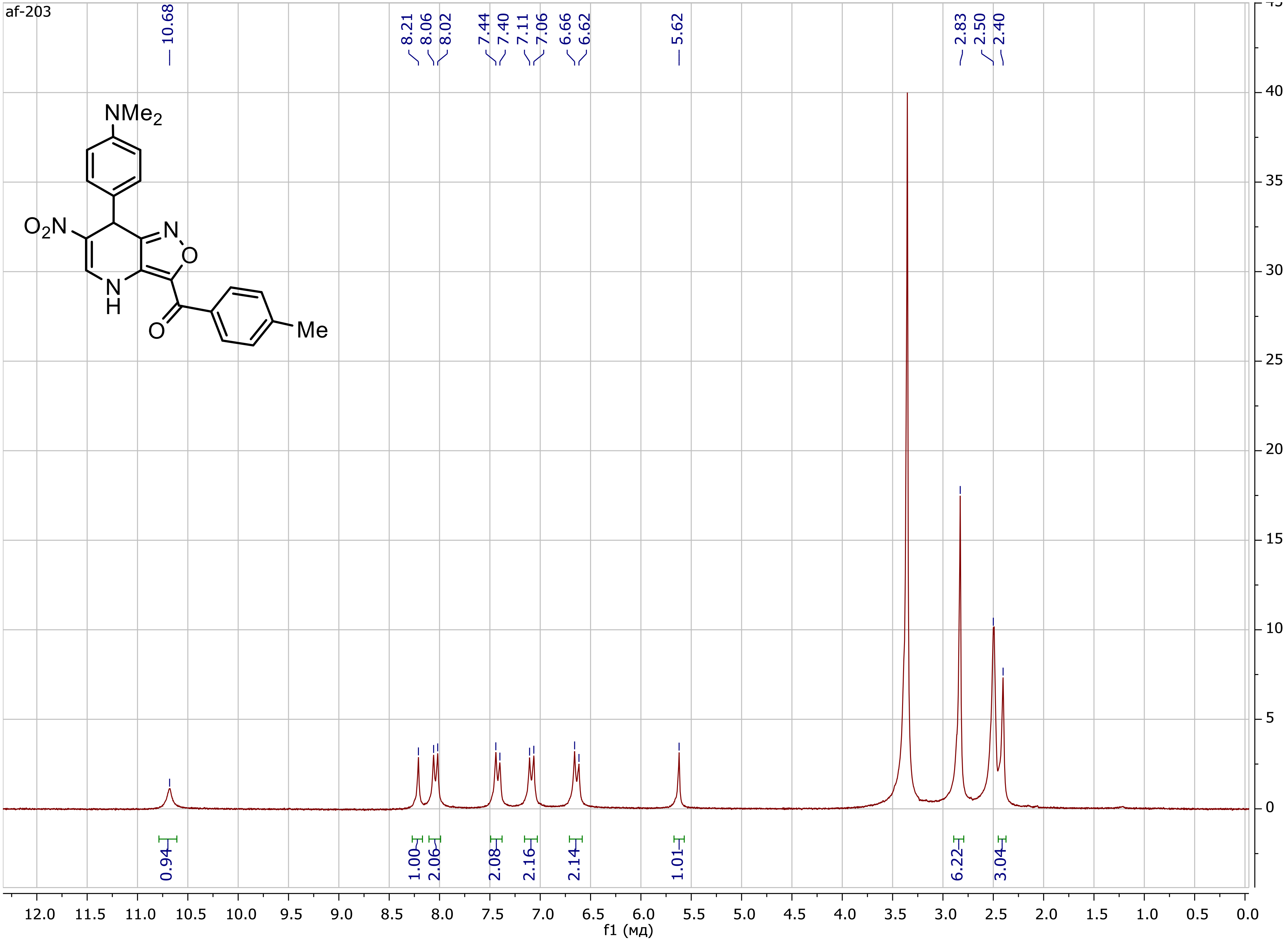
af-203
10.68
8.21
8.06
8.02
7.44
7.40
7.11
7.06
6.66
6.62
5.62
2.83
2.50
2.40
NMe2
O2N
N
O
N
H
O
Me
0.94
1.00
2.06
2.08
2.16
2.14
1.01
6.22
3.04
f1 (мд)

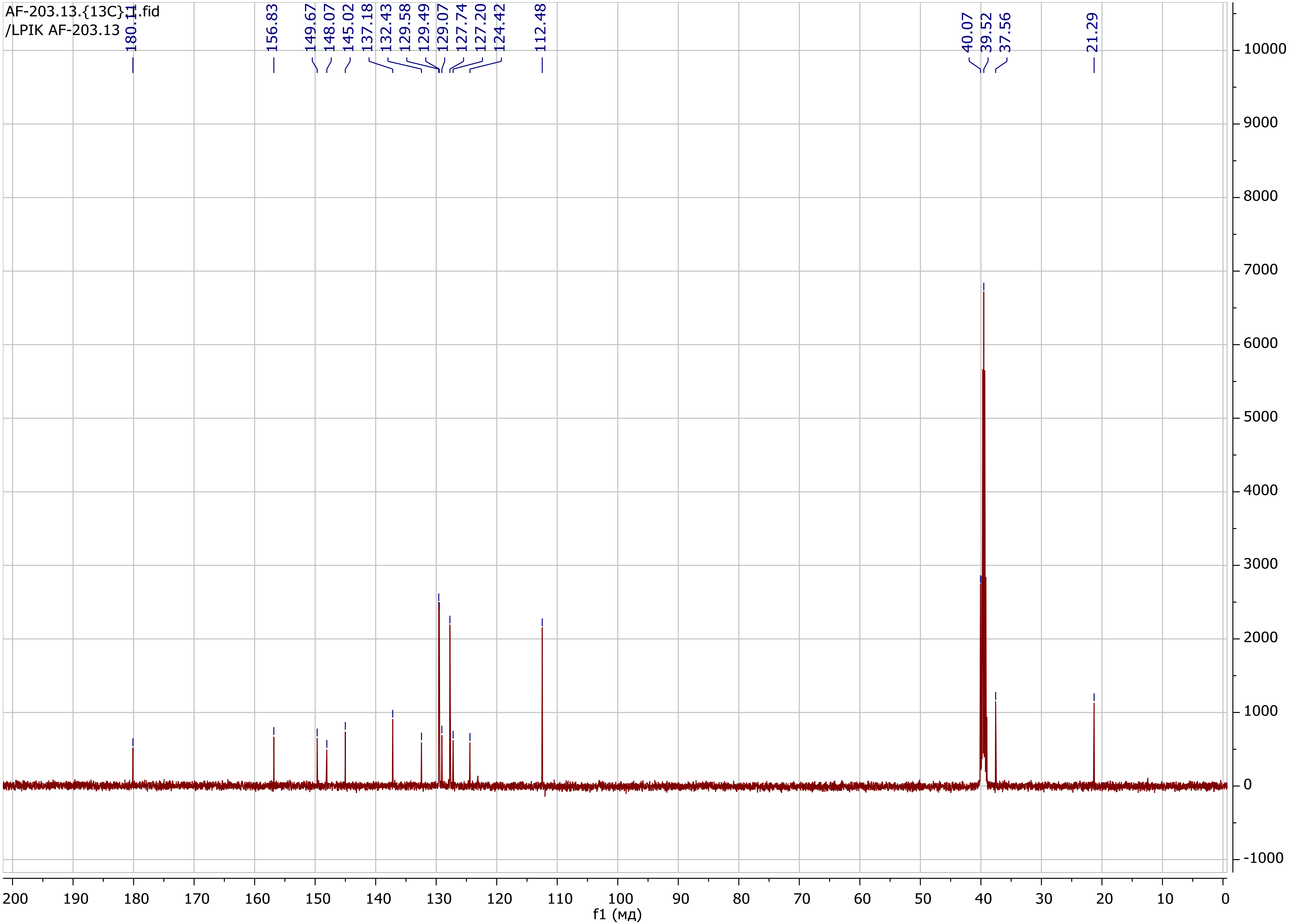
AF-203.13.{13C}.1.fid
/LPIK AF-203.13
180.11
156.83
149.67
148.07
145.02
137.18
132.43
129.58
129.49
129.07
127.74
127.20
124.42
112.48
40.07
39.52
37.56
21.29
f1 (мд)

Display Report

Analysis Info

Analysis Name	D:\Data\Kolotyrkina\2019\Bastrakov\0326047.d	Acquisition Date	26.03.2019 20:00:38
Method	tune_50-1600.m	Operator	BDAL@DE
Sample Name	/LPIK AF-203	Instrument / Ser#	micrOTOF 10248
Comment	C22H20N4O4 mH 405.1557 calibrant added CH3CN		

Acquisition Parameter

Source Type	ESI	Ion Polarity	Positive	Set Nebulizer	1.0 Bar
Focus	Not active			Set Dry Heater	200 °C
Scan Begin	50 m/z	Set Capillary	4500 V	Set Dry Gas	4.0 l/min
Scan End	1600 m/z	Set End Plate Offset	-500 V	Set Divert Valve	Waste

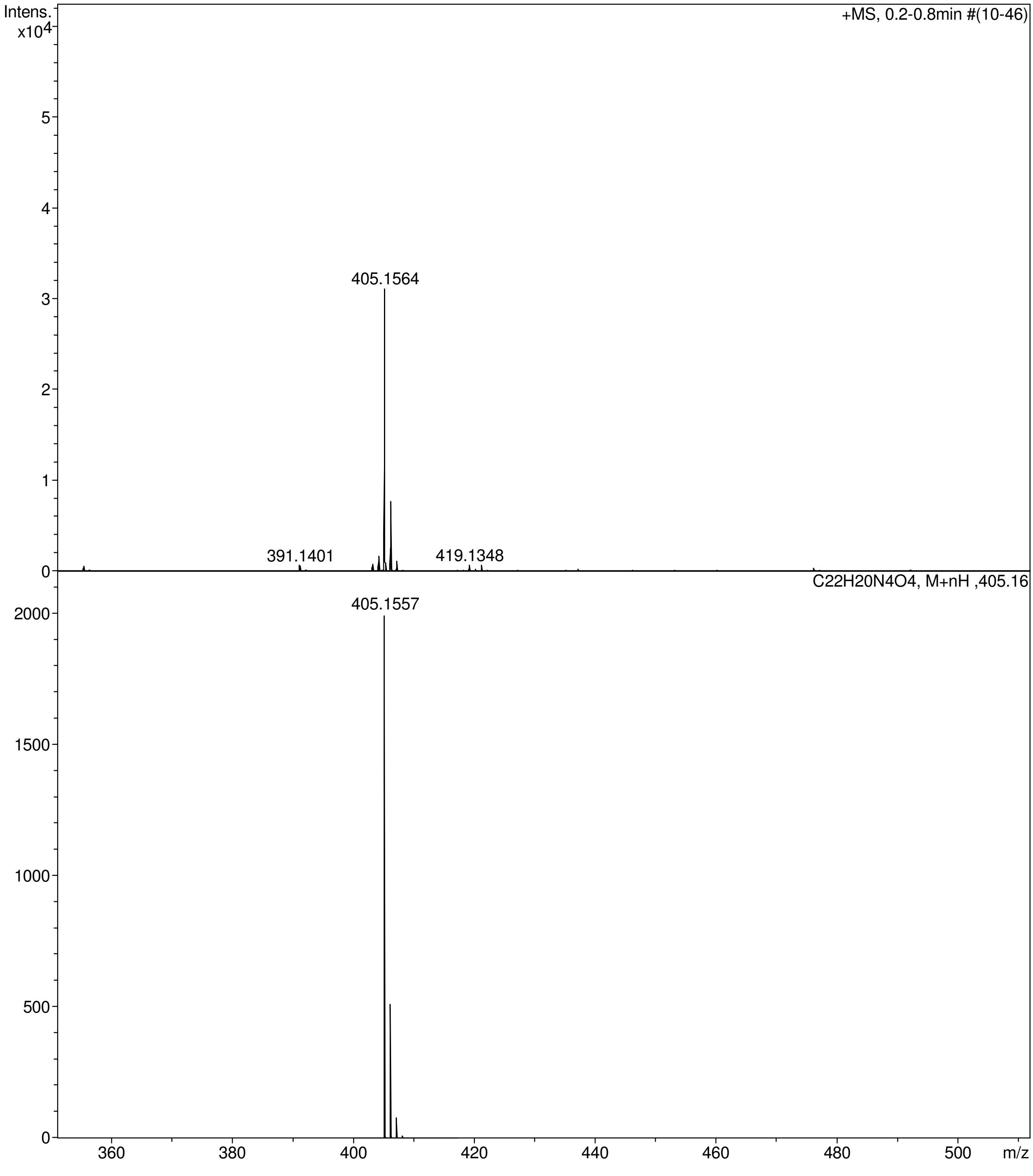

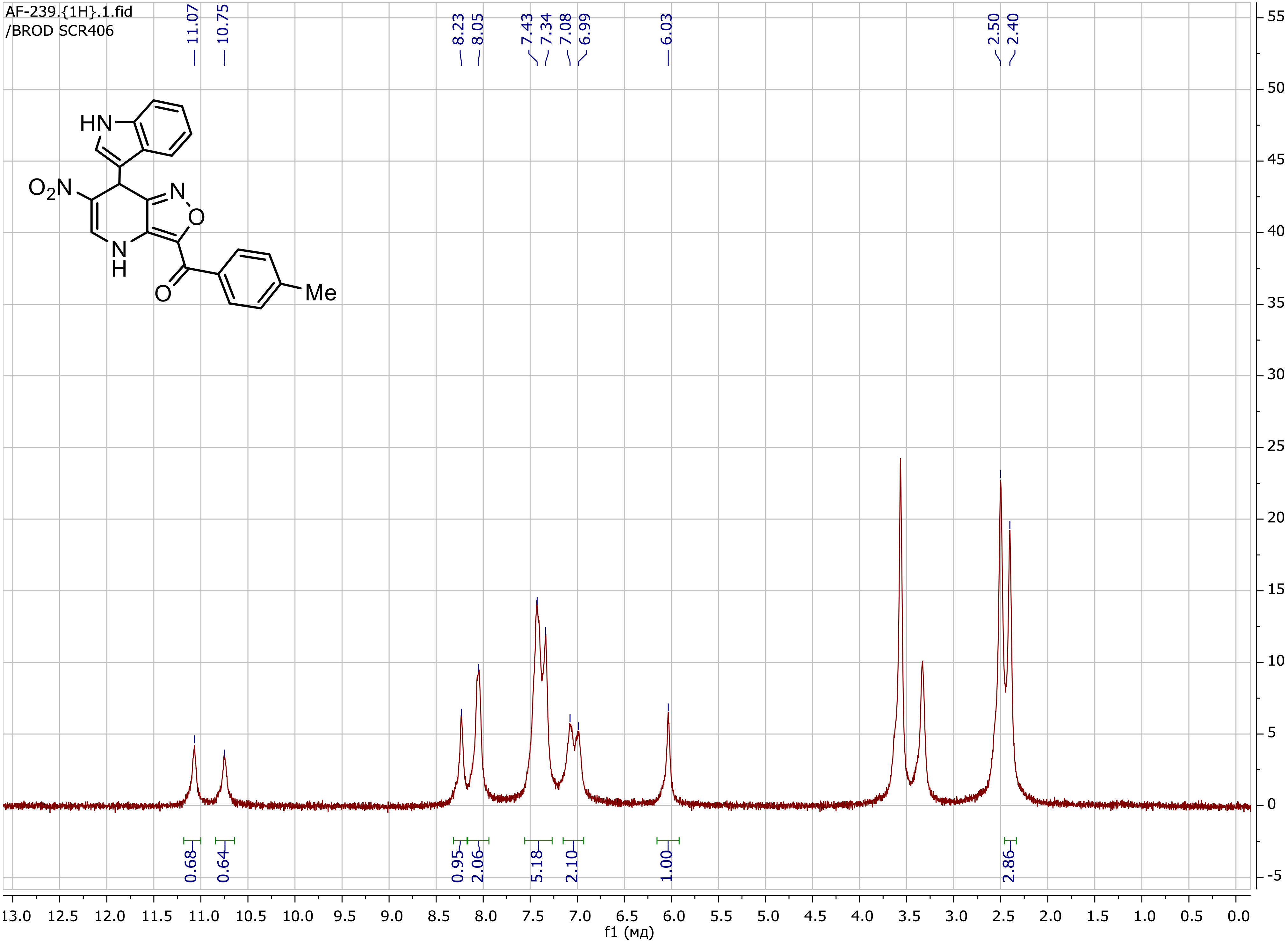
AF-239.{1H}.1.fid
/BROD SCR406
11.07
10.75
8.23
8.05
7.43
7.34
7.08
6.99
6.03
2.50
2.40
HN
O_2N
N
O
N
H
O
Me
0.68
0.64
0.95
2.06
5.18
2.10
1.00
2.86
f1 (мд)

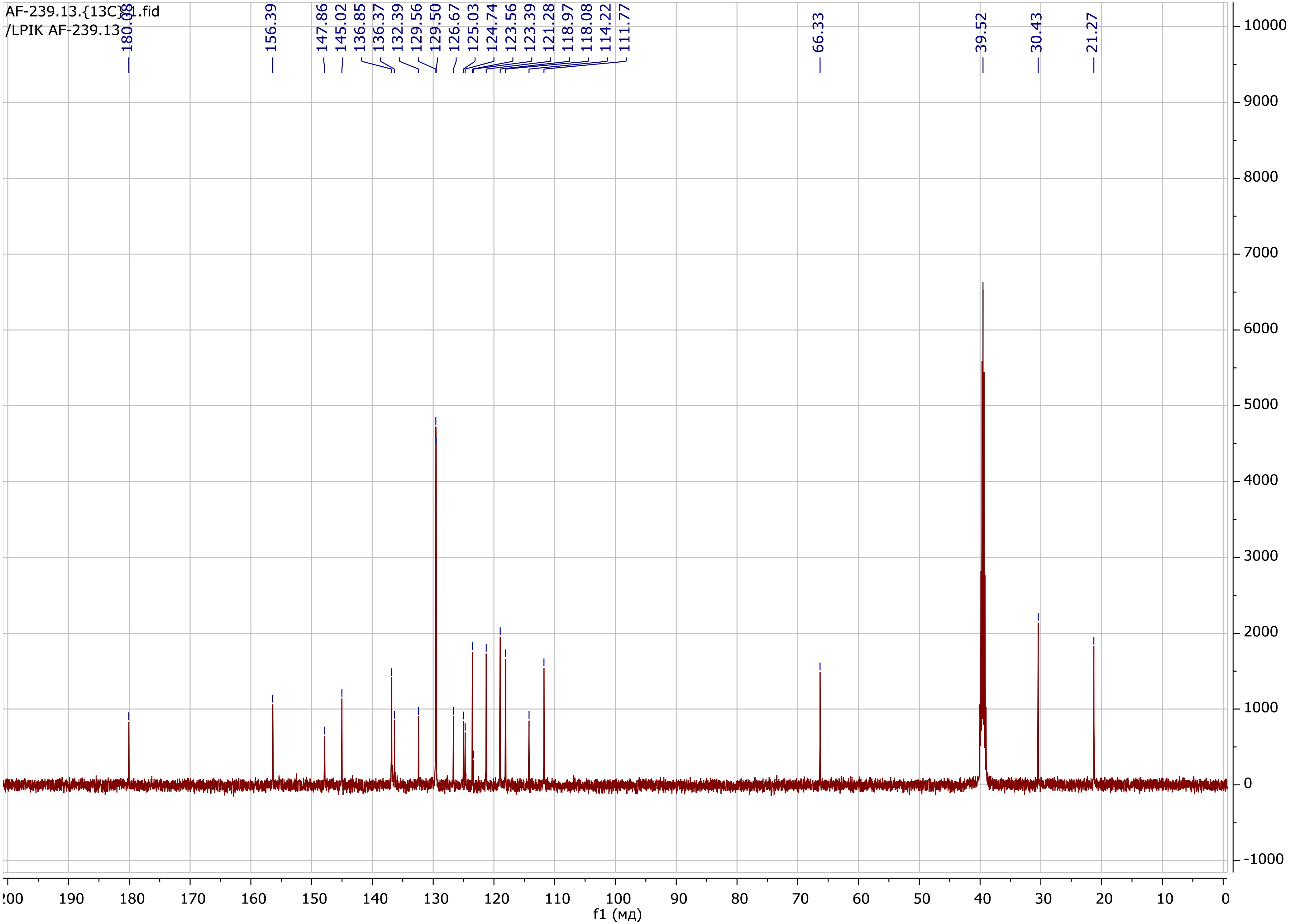
AF-239.13.{13C}01.fid
/LPIK AF-239.13
180.08
156.39
147.86
145.02
136.85
136.37
132.39
129.56
129.50
126.67
125.03
124.74
123.56
123.39
121.28
118.97
118.08
114.22
111.77
66.33
39.52
30.43
21.27
f1 (мд)

Display Report

Analysis Info

Analysis Name	D:\Data\Kolotyrkina\2019\Bastrakov\0326049.d	Acquisition Date	26.03.2019 20:09:53
Method	tune_50-1600.m	Operator	BDAL@DE
Sample Name	/LPIK AF-239	Instrument / Ser#	micrOTOF 10248
Comment	C22H16N4O4 mH 401.1243 calibrant added CH3CN		

Acquisition Parameter

Source Type	ESI	Ion Polarity	Positive	Set Nebulizer	1.0 Bar
Focus	Not active			Set Dry Heater	200 °C
Scan Begin	50 m/z	Set Capillary	4500 V	Set Dry Gas	4.0 l/min
Scan End	1600 m/z	Set End Plate Offset	-500 V	Set Divert Valve	Waste

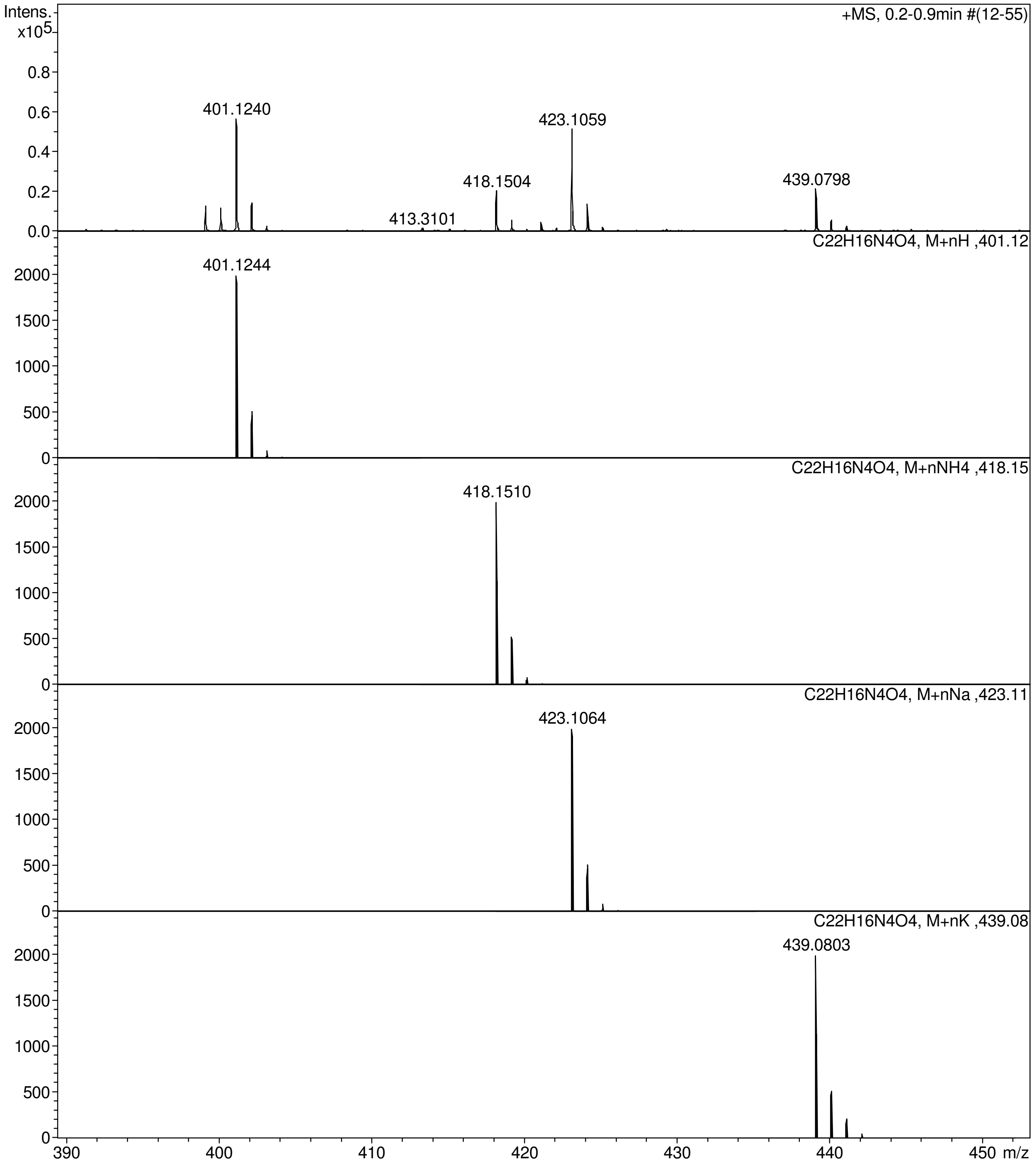

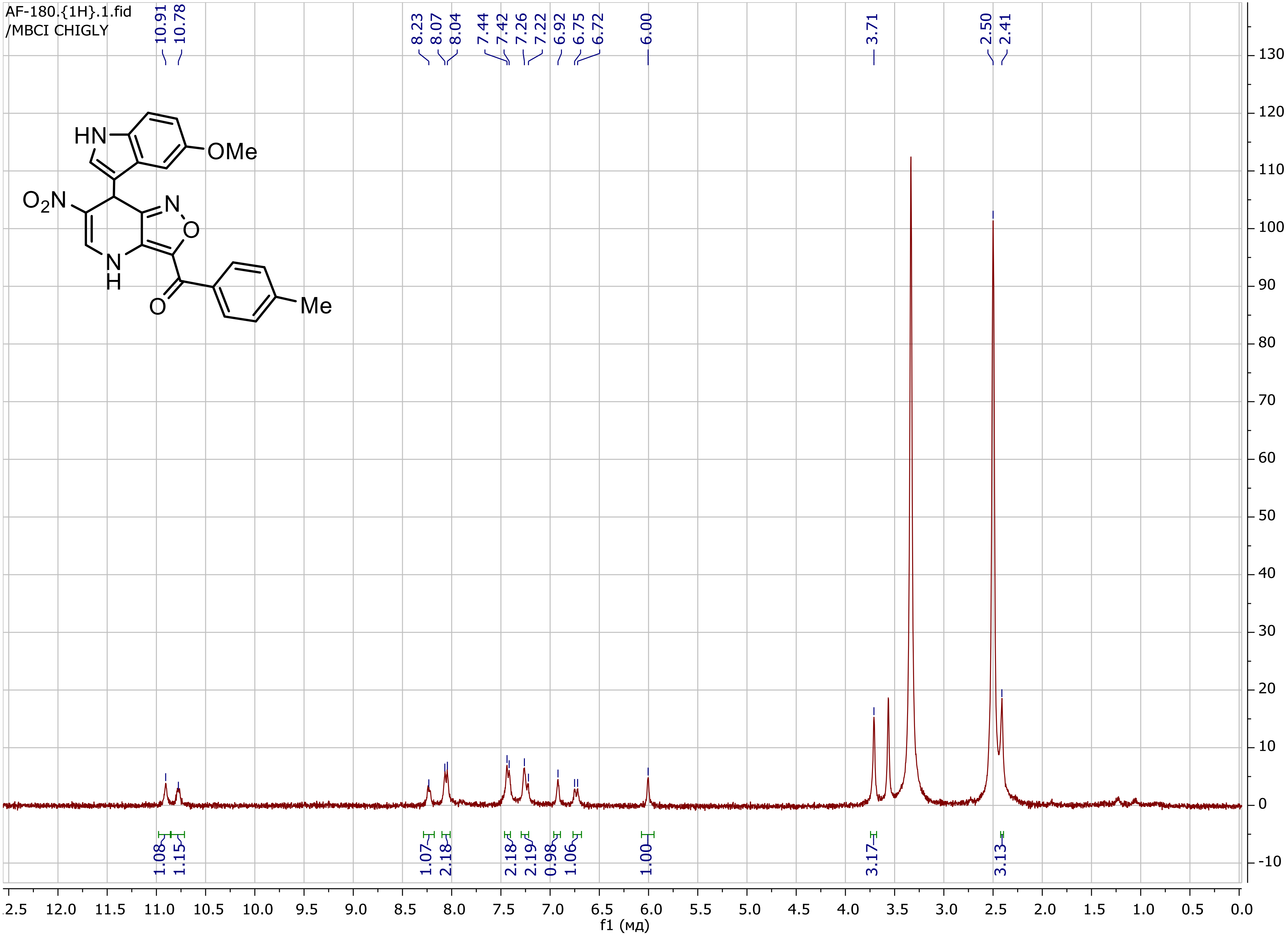

AF-180.{1H}.1.fid
/MBCI CHIGLY
10.91
10.78
8.23
8.07
8.04
7.44
7.42
7.26
7.22
6.92
6.75
6.72
6.00
3.71
2.50
2.41
HN
OMe
O_2N
N
O
N
H
O
Me
1.08
1.15
1.07
2.18
2.18
2.19
0.98
1.06
1.00
3.17
3.13
f1 (мд)

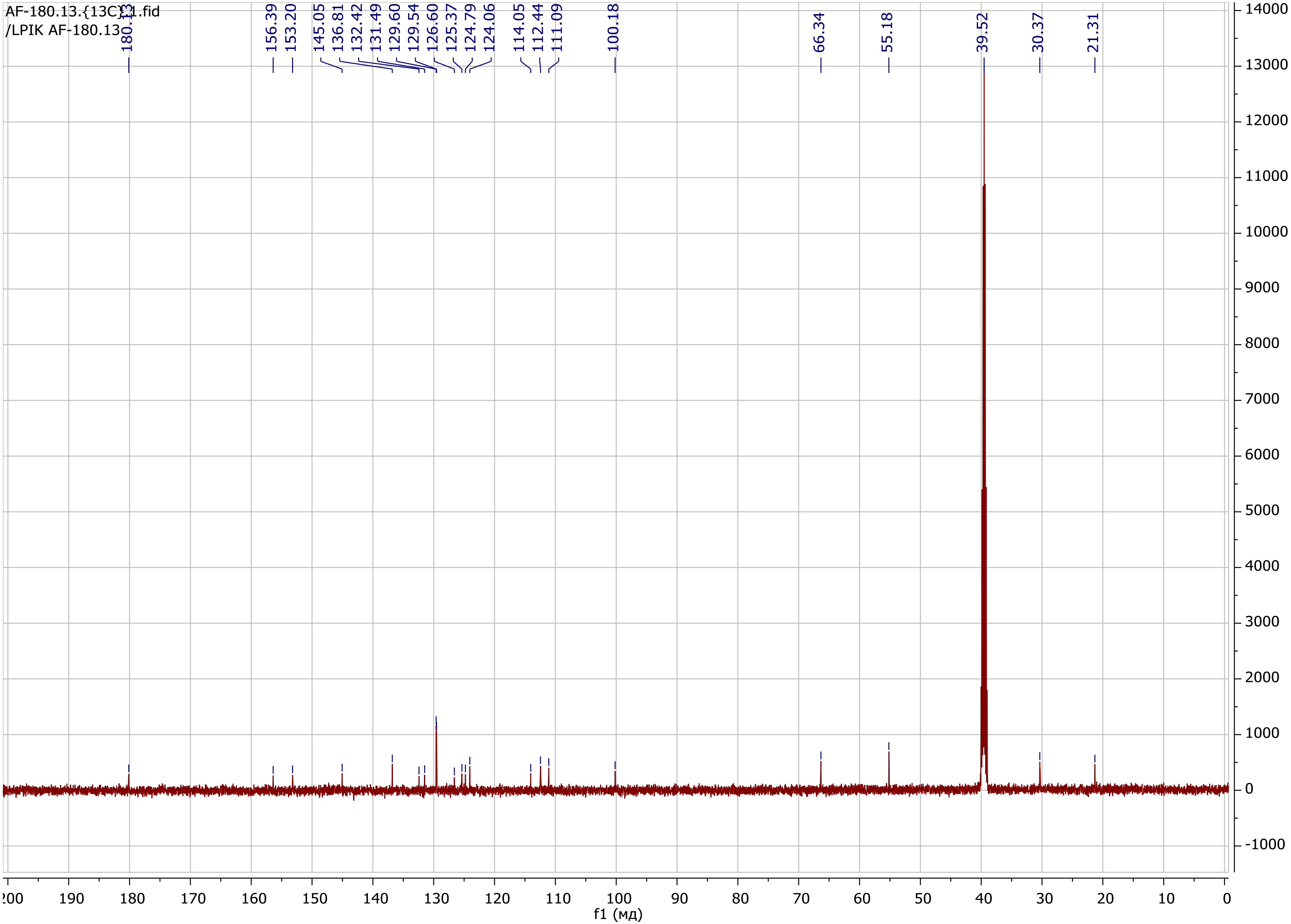
AF-180.13.{13C}11.fid
/LPIK AF-180.13
180.13
156.39
153.20
145.05
136.81
132.42
131.49
129.60
129.54
126.60
125.37
124.79
124.06
114.05
112.44
111.09
100.18
66.34
55.18
39.52
30.37
21.31
f1 (мд)

Display Report

Analysis Info

Analysis Name	D:\Data\Kolotyrkina\2018\Bastrakov\0703005.d	Acquisition Date	03.07.2018 10:59:14
Method	tune_50-1600.m	Operator	BDAL@DE
Sample Name	/LPIK AF-180	Instrument / Ser#	micrOTOF 10248
Comment	C23H18N4O5 mH 431.1349 calibrant added		

Acquisition Parameter

Source Type	ESI	Ion Polarity	Positive	Set Nebulizer	1.0 Bar
Focus	Not active			Set Dry Heater	200 °C
Scan Begin	50 m/z	Set Capillary	4500 V	Set Dry Gas	4.0 l/min
Scan End	1600 m/z	Set End Plate Offset	-500 V	Set Divert Valve	Waste

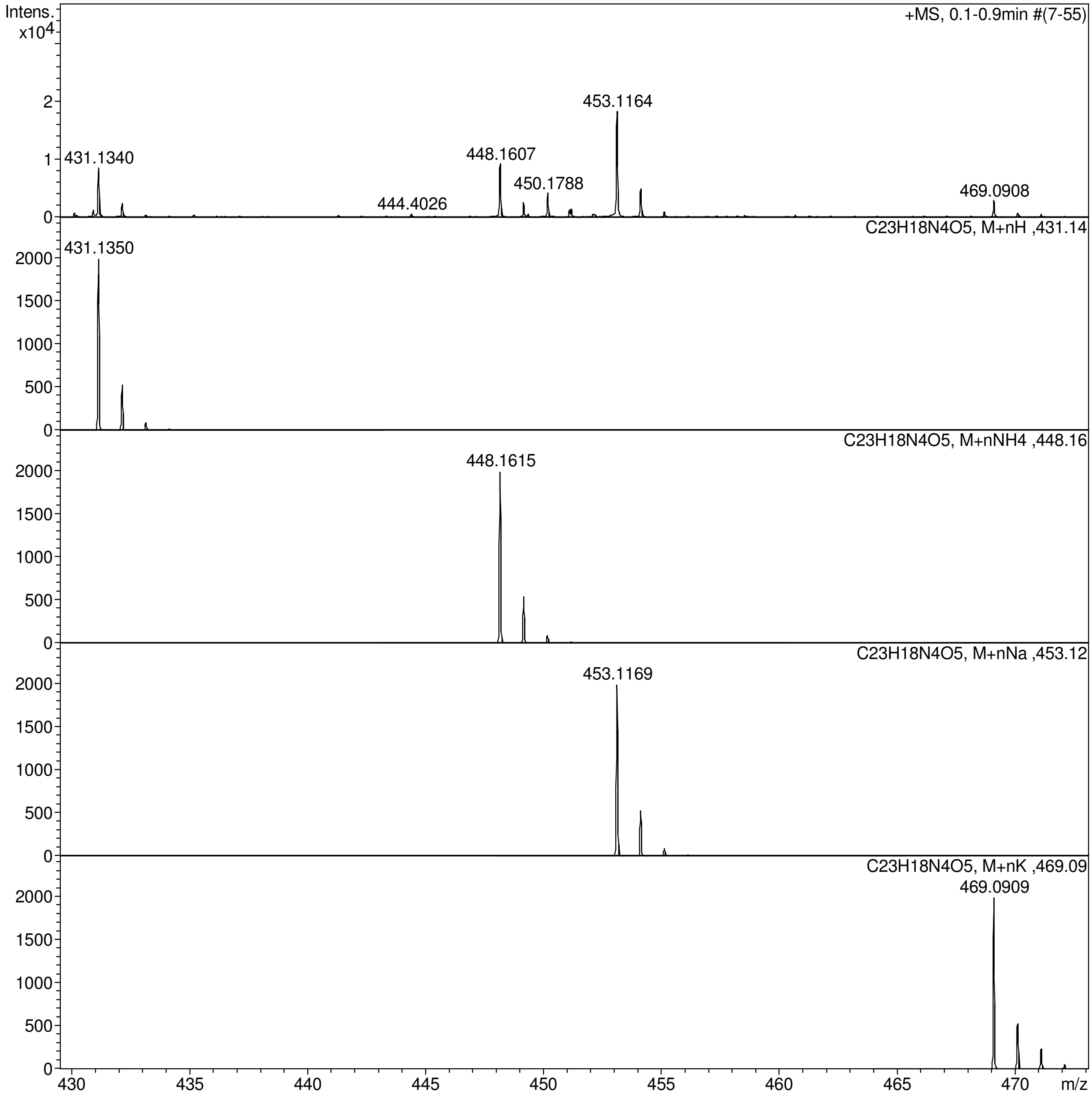

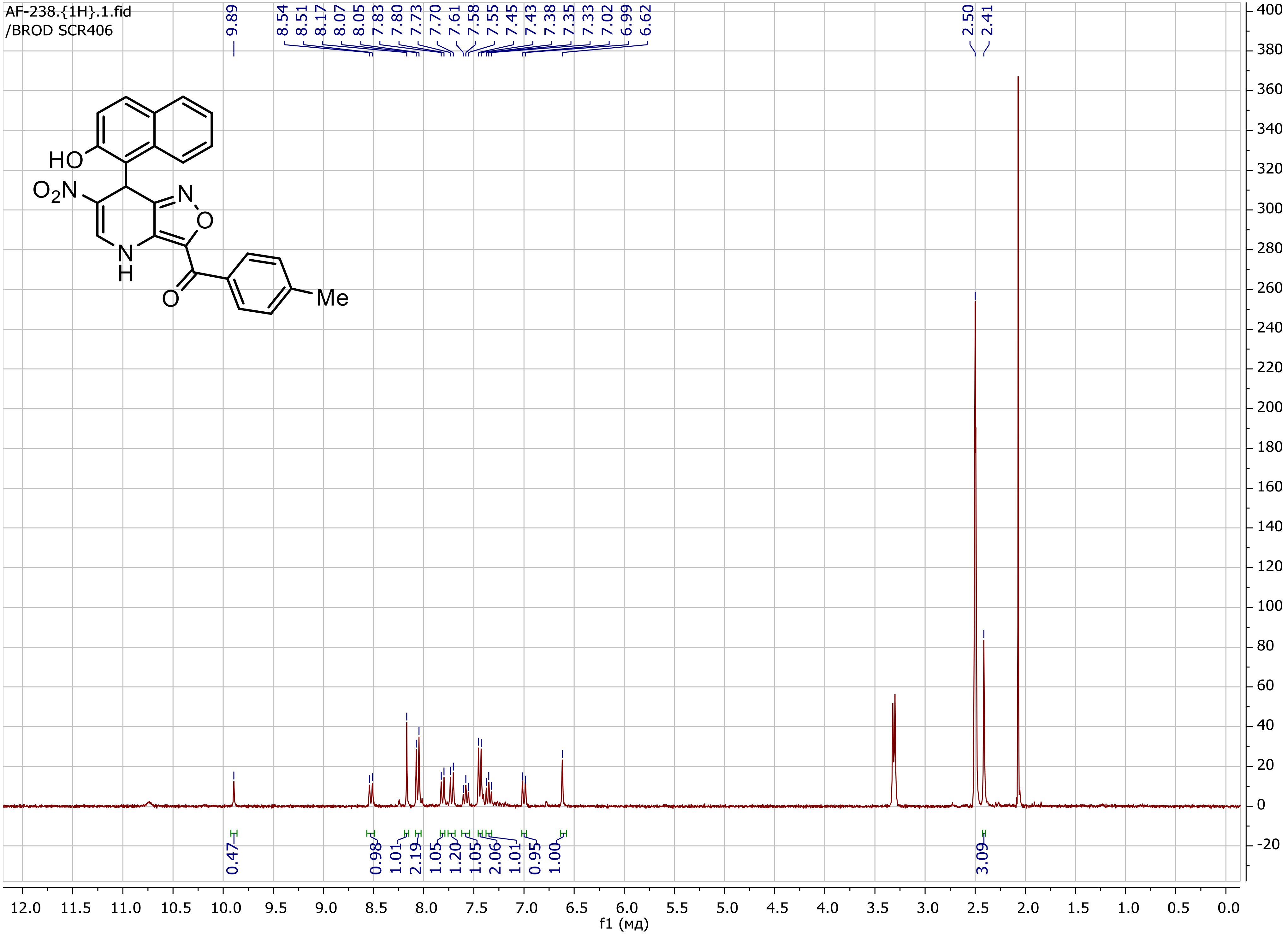

AF-238.{1H}.1.fid
/BROD SCR406
9.89
8.54 8.51 8.17 8.07 8.05 7.83 7.80 7.73 7.70 7.61 7.58 7.55 7.45 7.43 7.38 7.35 7.33 7.02 6.99 6.62
2.50 2.41
HO
O2N
N
O
N
H
O
Me
0.47 0.98 1.01 2.19 1.05 1.20 1.05 2.06 1.01 0.95 1.00
3.09
f1 (мд)

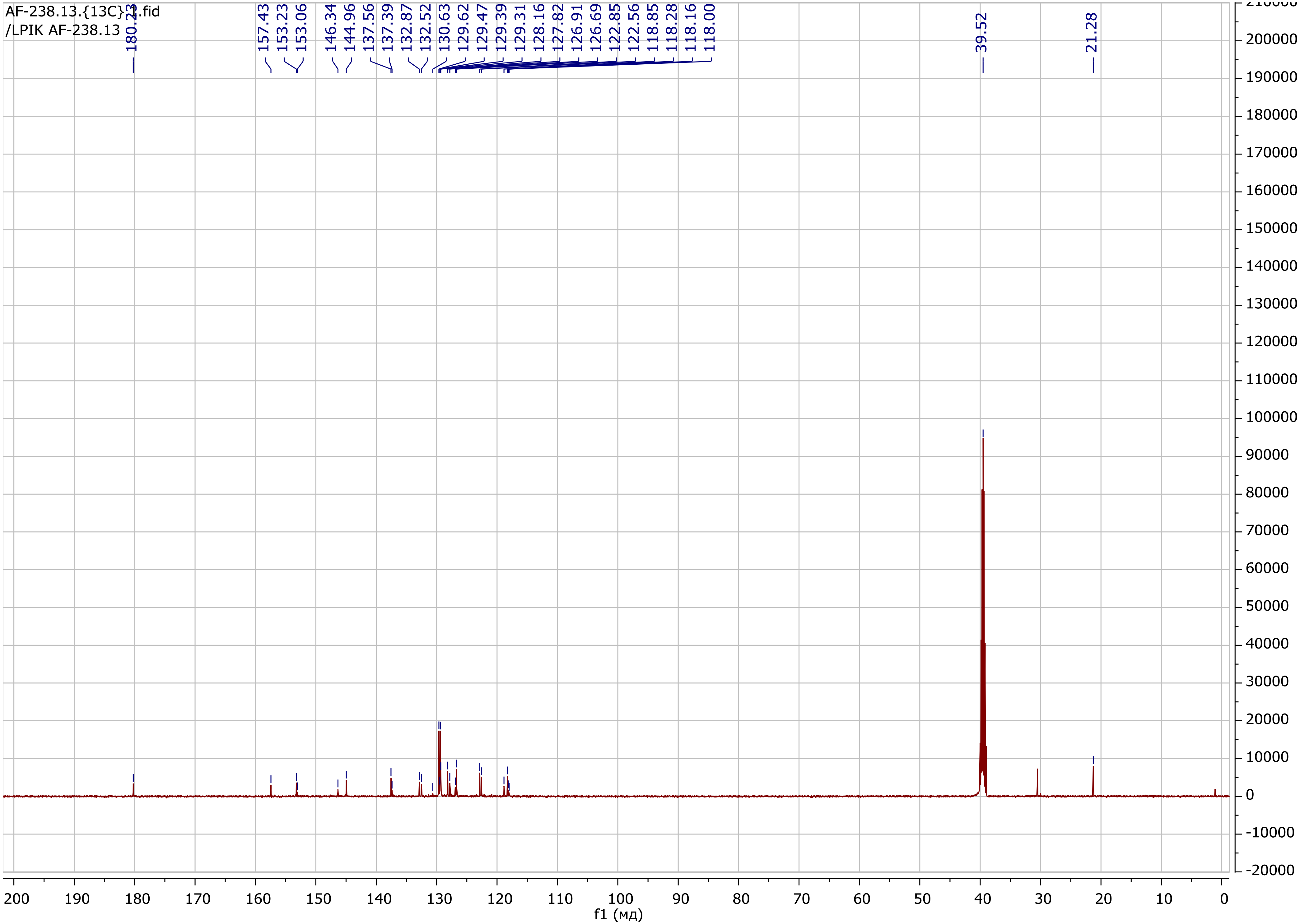

AF-238.13.{13C}1.fid
/LPIK AF-238.13
180.23
157.43
153.23
153.06
146.34
144.96
137.56
137.39
132.87
132.52
130.63
129.62
129.47
129.39
129.31
128.16
127.82
126.91
126.69
122.85
122.56
118.85
118.28
118.16
118.00
39.52
21.28
f1 (мд)

Display Report

Analysis Info

Analysis Name	D:\Data\Kolotyrkina\2019\Bastrakov\0326048.d	Acquisition Date	26.03.2019 20:04:56
Method	tune_50-1600.m	Operator	BDAL@DE
Sample Name	/LPIK AF-238	Instrument / Ser#	micrOTOF 10248
Comment	C24H17N3O5 mH 428.1240 calibrant added CH3CN		

Acquisition Parameter

Source Type	ESI	Ion Polarity	Positive	Set Nebulizer	1.0 Bar
Focus	Not active			Set Dry Heater	200 °C
Scan Begin	50 m/z	Set Capillary	4500 V	Set Dry Gas	4.0 l/min
Scan End	1600 m/z	Set End Plate Offset	-500 V	Set Divert Valve	Waste

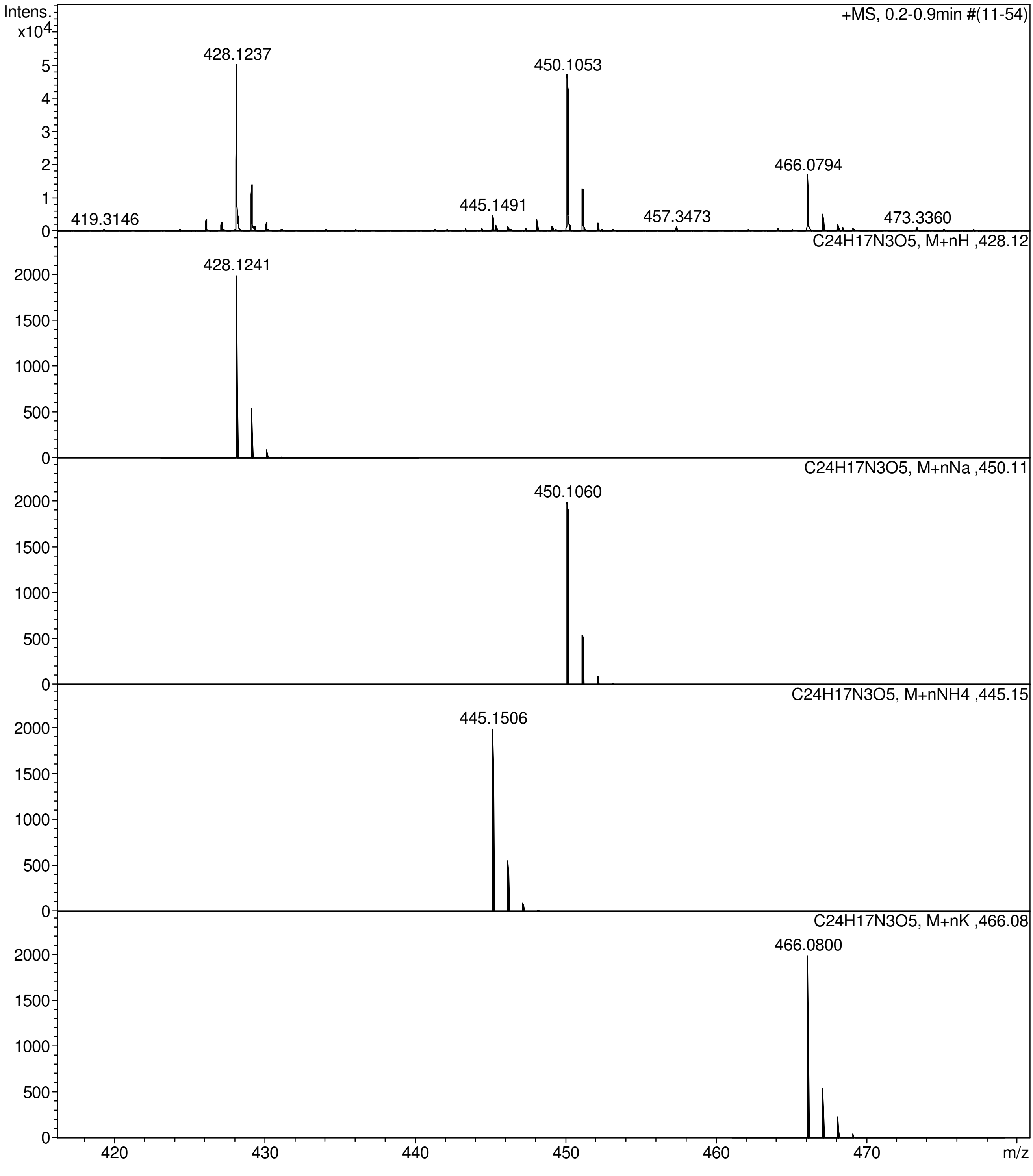

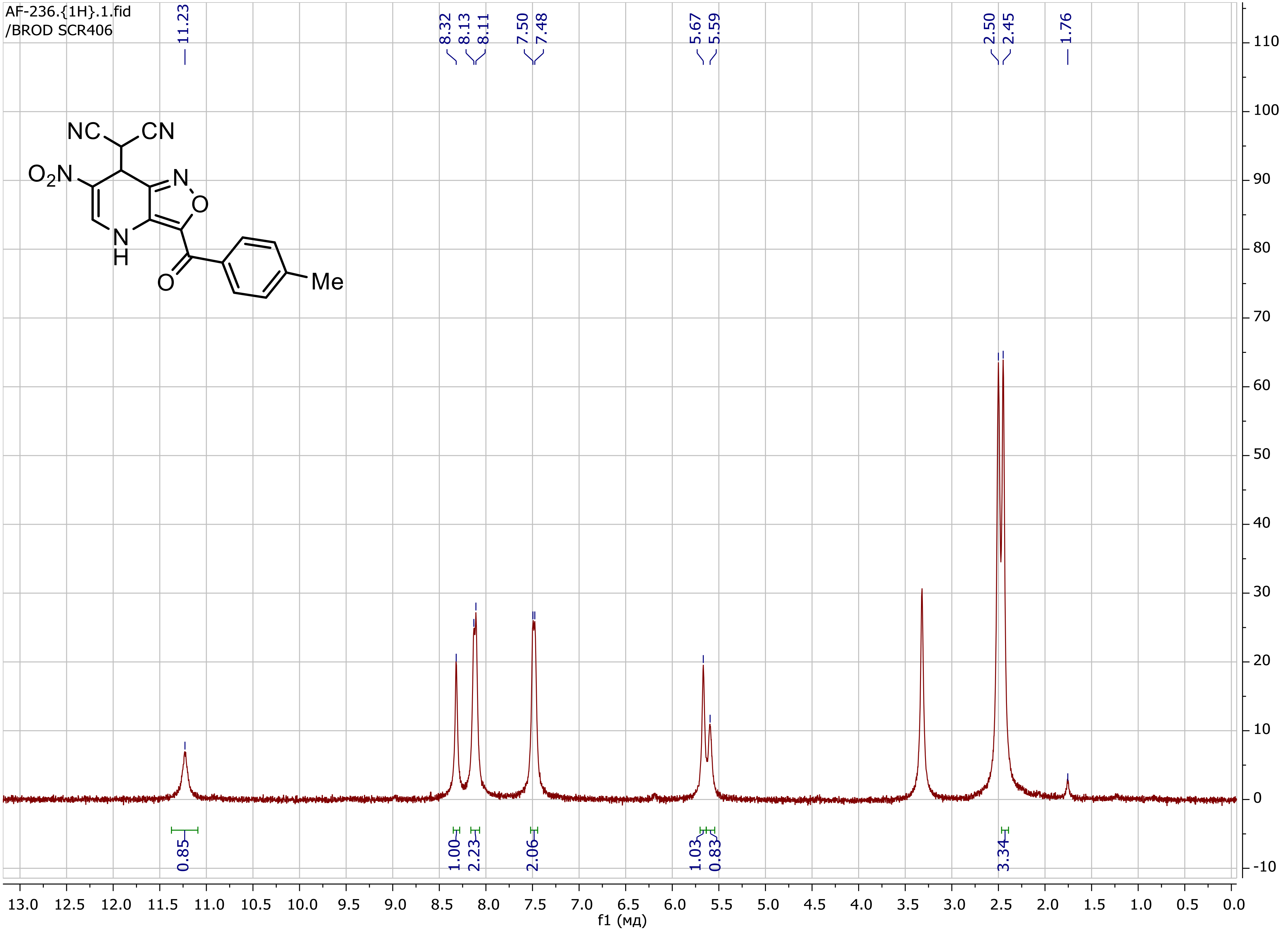
AF-236.{1H}.1.fid
/BROD SCR406
11.23
8.32
8.13
8.11
7.50
7.48
5.67
5.59
2.50
2.45
1.76
NC
CN
O2N
N
O
N
H
O
Me
0.85
1.00
2.23
2.06
1.03
0.83
3.34
f1 (мд)

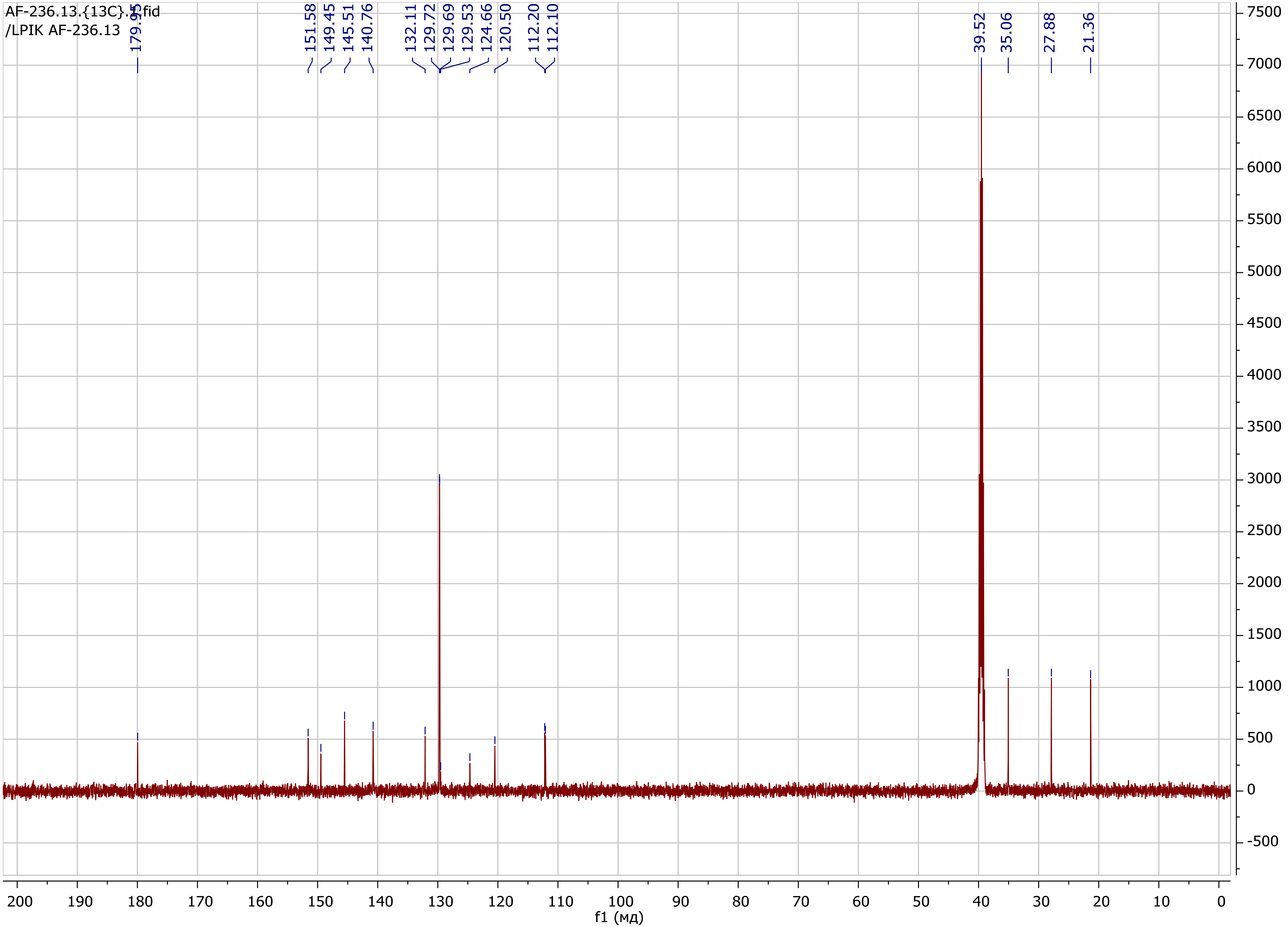
AF-236.13.{13C}.1.fid
/LPIK AF-236.13
179.95
151.58
149.45
145.51
140.76
132.11
129.72
129.69
129.53
124.66
120.50
112.20
112.10
39.52
35.06
27.88
21.36
f1 (мд)

Display Report

Analysis Info

Analysis Name	D:\Data\Kolotyrkina\2019\Bastrakov\0326045.d	Acquisition Date	26.03.2019 19:47:49
Method	tune_50-1600.m	Operator	BDAL@DE
Sample Name	/LPIK AF-236	Instrument / Ser#	micrOTOF 10248
Comment	C17H11N5O4 mH 350.0883 calibrant added CH3CN		

Acquisition Parameter

Source Type	ESI	Ion Polarity	Positive	Set Nebulizer	1.0 Bar
Focus	Not active			Set Dry Heater	200 °C
Scan Begin	50 m/z	Set Capillary	4500 V	Set Dry Gas	4.0 l/min
Scan End	1600 m/z	Set End Plate Offset	-500 V	Set Divert Valve	Waste

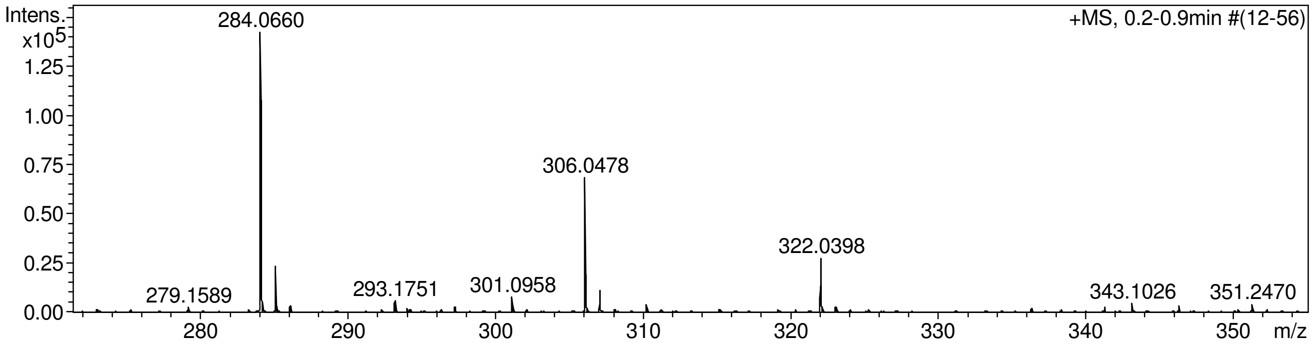

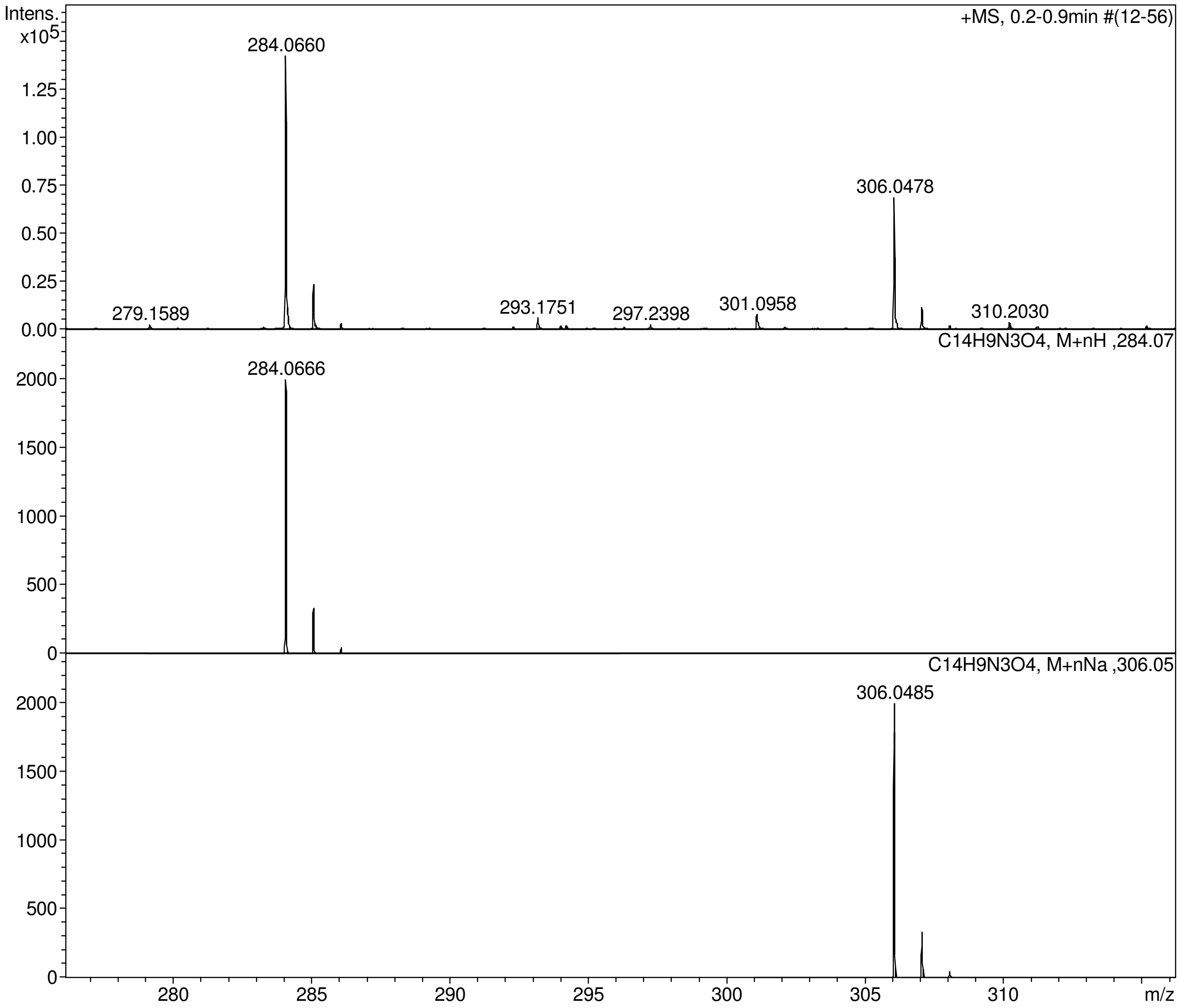

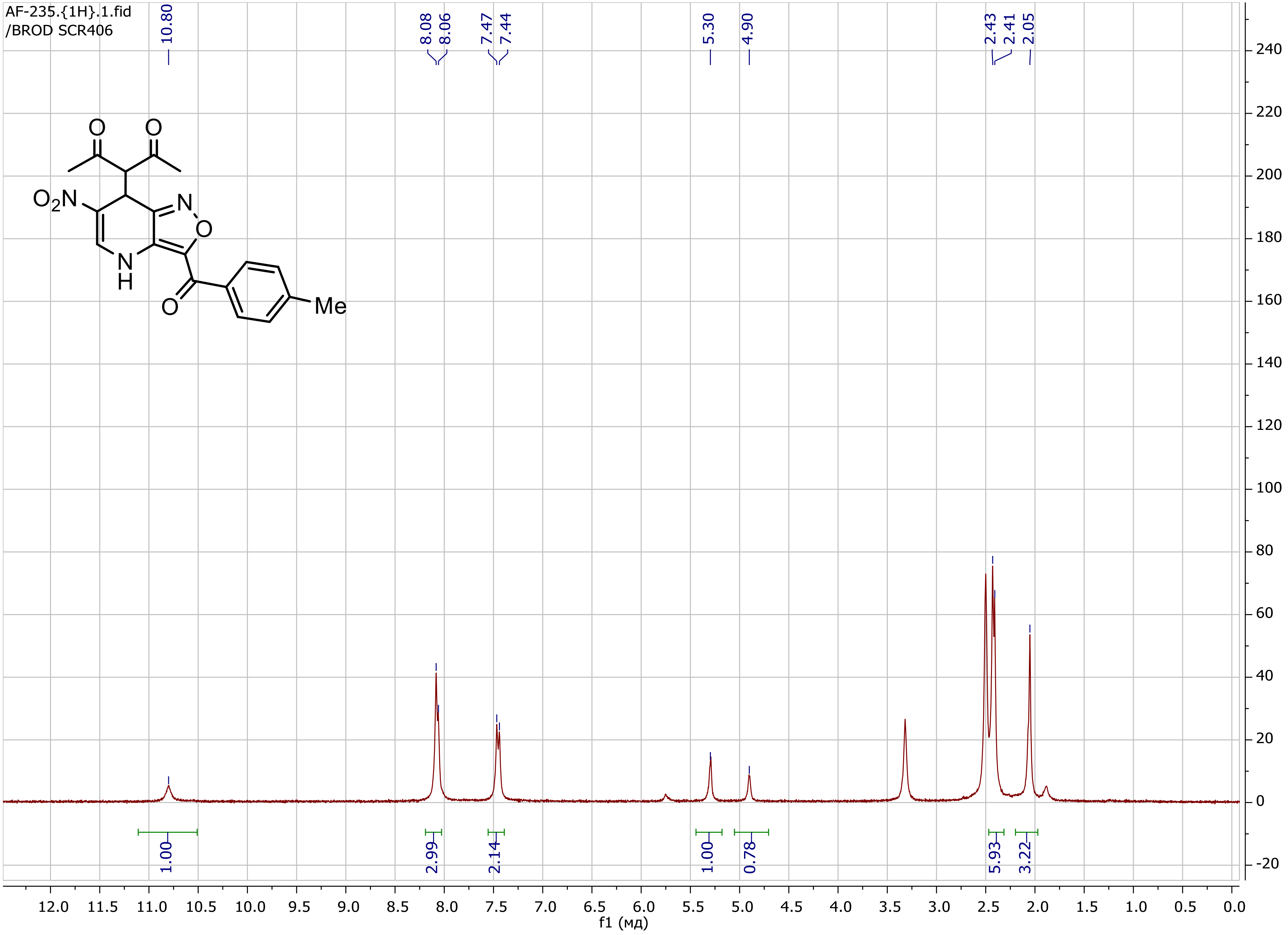
AF-235.{1H}.1.fid
/BROD SCR406
10.80
8.08
8.06
7.47
7.44
5.30
4.90
2.43
2.41
2.05
O
O
O_2N
N
O
N
H
O
Me
1.00
2.99
2.14
1.00
0.78
5.93
3.22
f1 (мд)

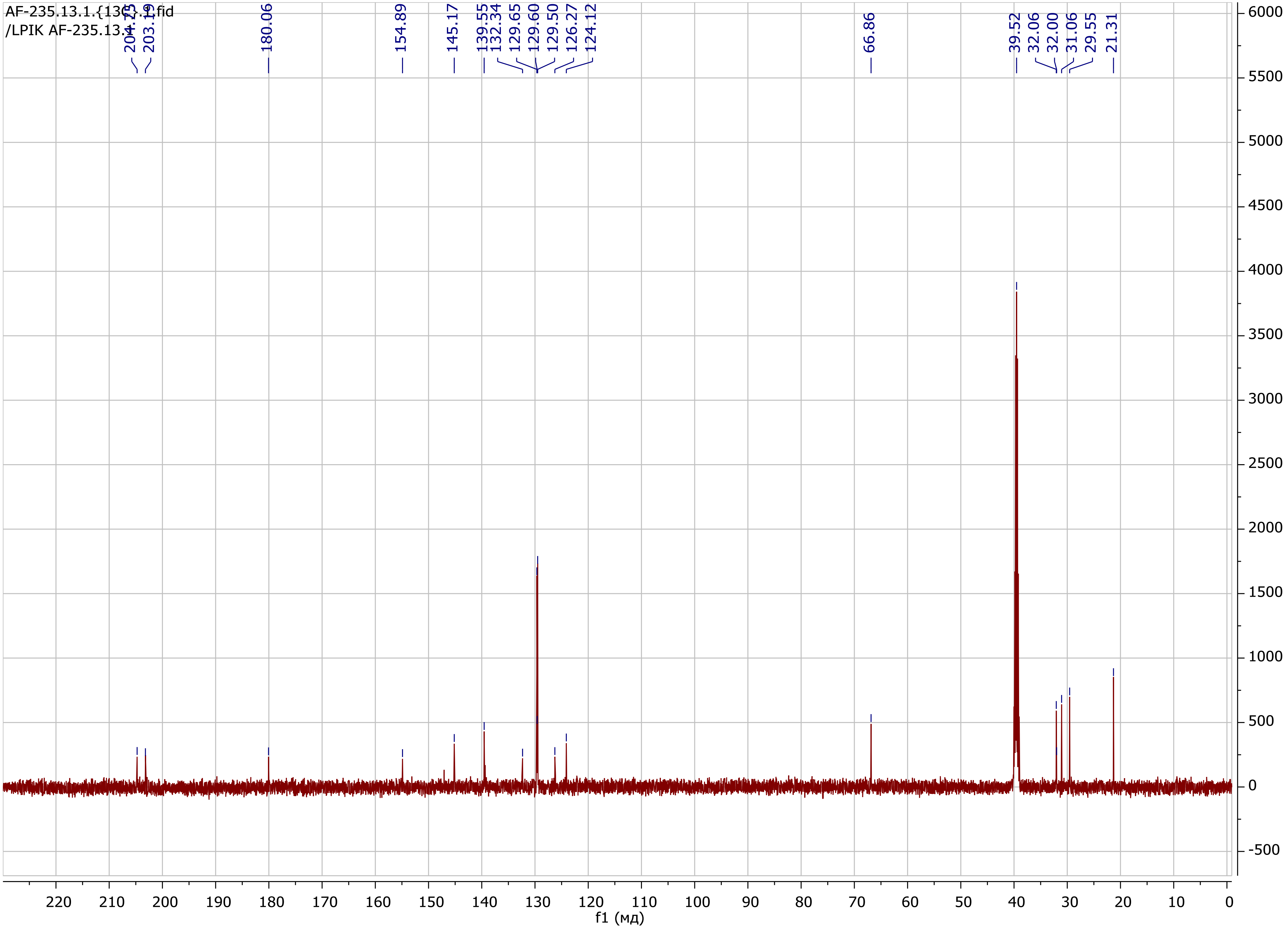
AF-235.13.1.{13C}.1.fid
/LPIK AF-235.13.1
204.75
203.19
180.06
154.89
145.17
139.55
132.34
129.65
129.60
129.50
126.27
124.12
66.86
39.52
32.06
32.00
31.06
29.55
21.31
6000
5500
5000
4500
4000
3500
3000
2500
2000
1500
1000
500
0
-500
220
210
200
190
180
170
160
150
140
130
120
110
100
90
80
70
60
50
40
30
20
10
0
f1 (мд)

Display Report

Analysis Info

Analysis Name	D:\Data\Kolotyrkina\2019\Bastrakov\0326043.d
Method	tune_50-1600.m
Sample Name	/LPIK AF-235
Comment	C19H17N3O6 mH 384.1190 calibrant added CH3CN
Acquisition Date	26.03.2019 19:37:54
Operator	BDAL@DE
Instrument / Ser#	micrOTOF 10248

Acquisition Parameter

Source Type	ESI	Ion Polarity	Positive	Set Nebulizer	1.0 Bar
Focus	Not active			Set Dry Heater	200 °C
Scan Begin	50 m/z	Set Capillary	4500 V	Set Dry Gas	4.0 l/min
Scan End	1600 m/z	Set End Plate Offset	-500 V	Set Divert Valve	Waste

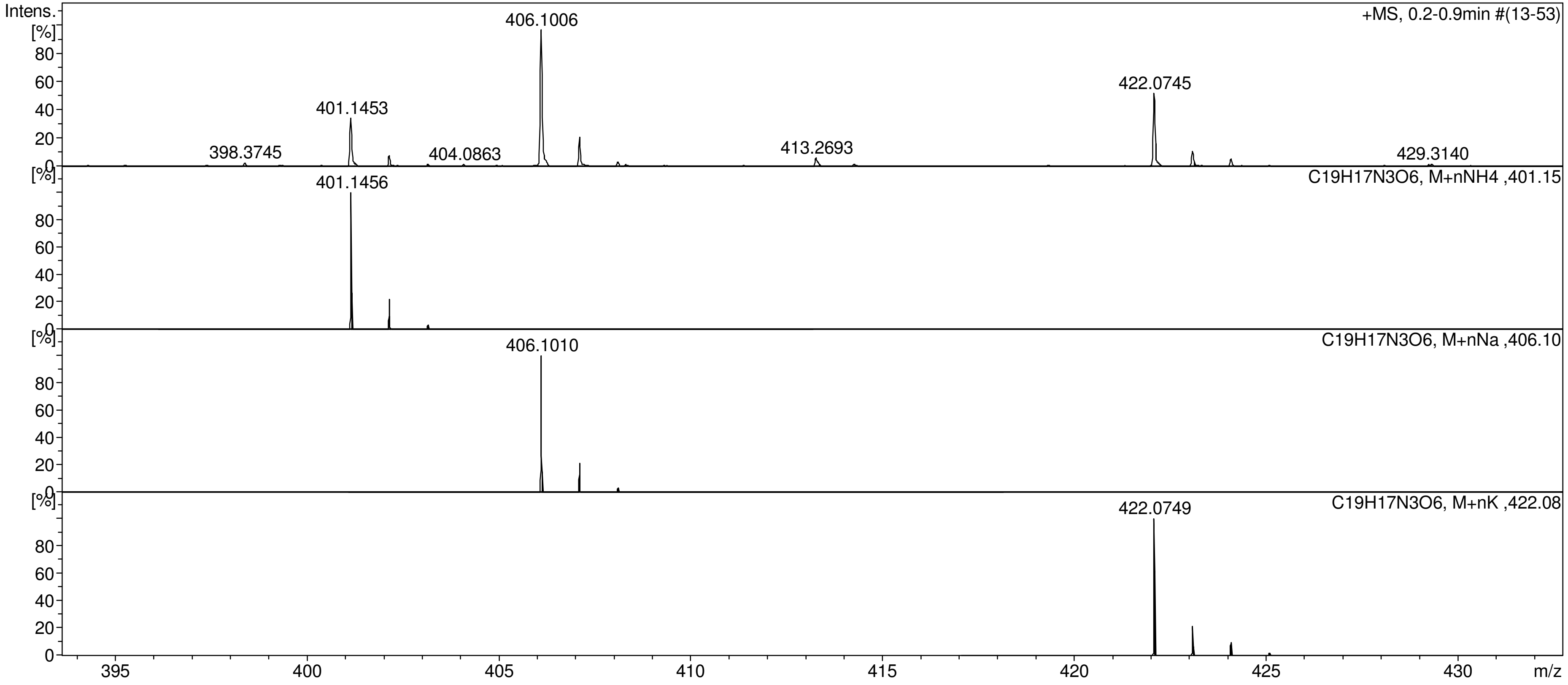

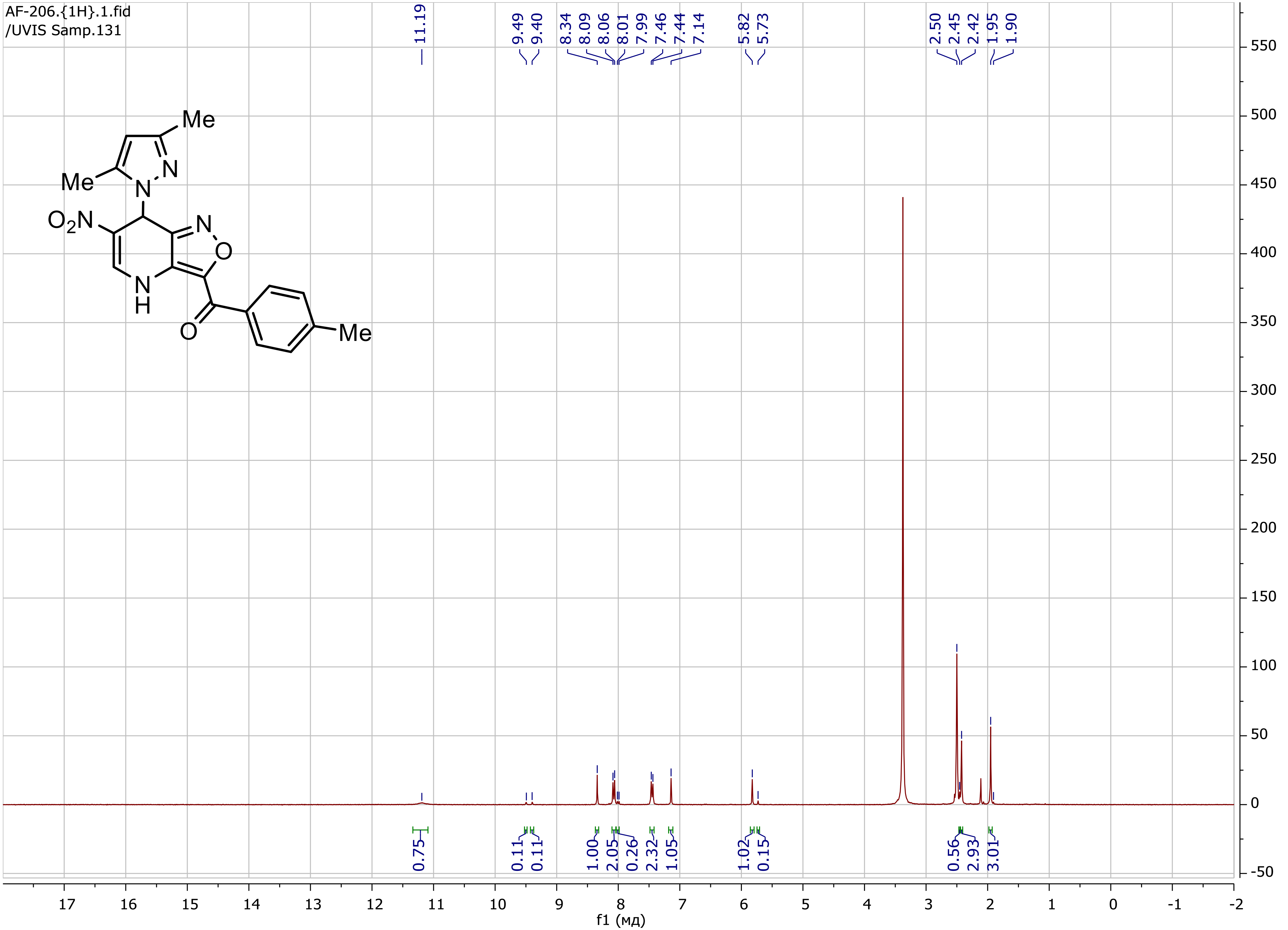

AF-206.{1H}.1.fid
/UVIS Samp.131
11.19
9.49
9.40
8.34
8.09
8.06
8.01
7.99
7.46
7.44
7.14
5.82
5.73
2.50
2.45
2.42
1.95
1.90
Me
Me
N
N
O2N
N
O
N
H
O
Me
0.75
0.11
0.11
1.00
2.05
0.26
2.32
1.05
1.02
0.15
0.56
2.93
3.01
f1 (мд)

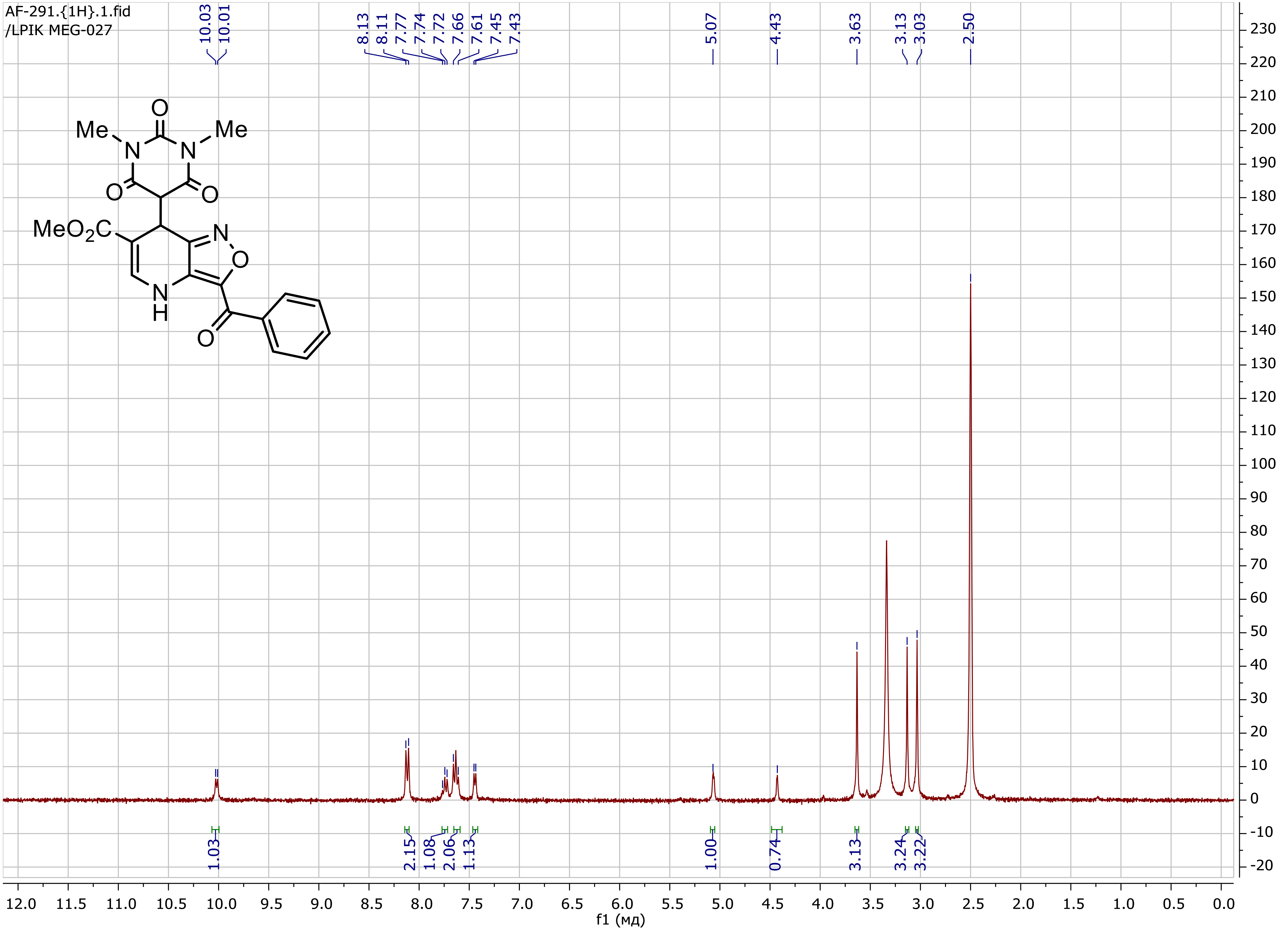
AF-291.{1H}.1.fid
/LPIK MEG-027
10.03
10.01
8.13
8.11
7.77
7.74
7.72
7.66
7.61
7.45
7.43
5.07
4.43
3.63
3.13
3.03
2.50
Me
N
O
MeO2C
N
O
H
1.03
2.15
1.08
2.06
1.13
1.00
0.74
3.13
3.24
3.22
f1 (мд)

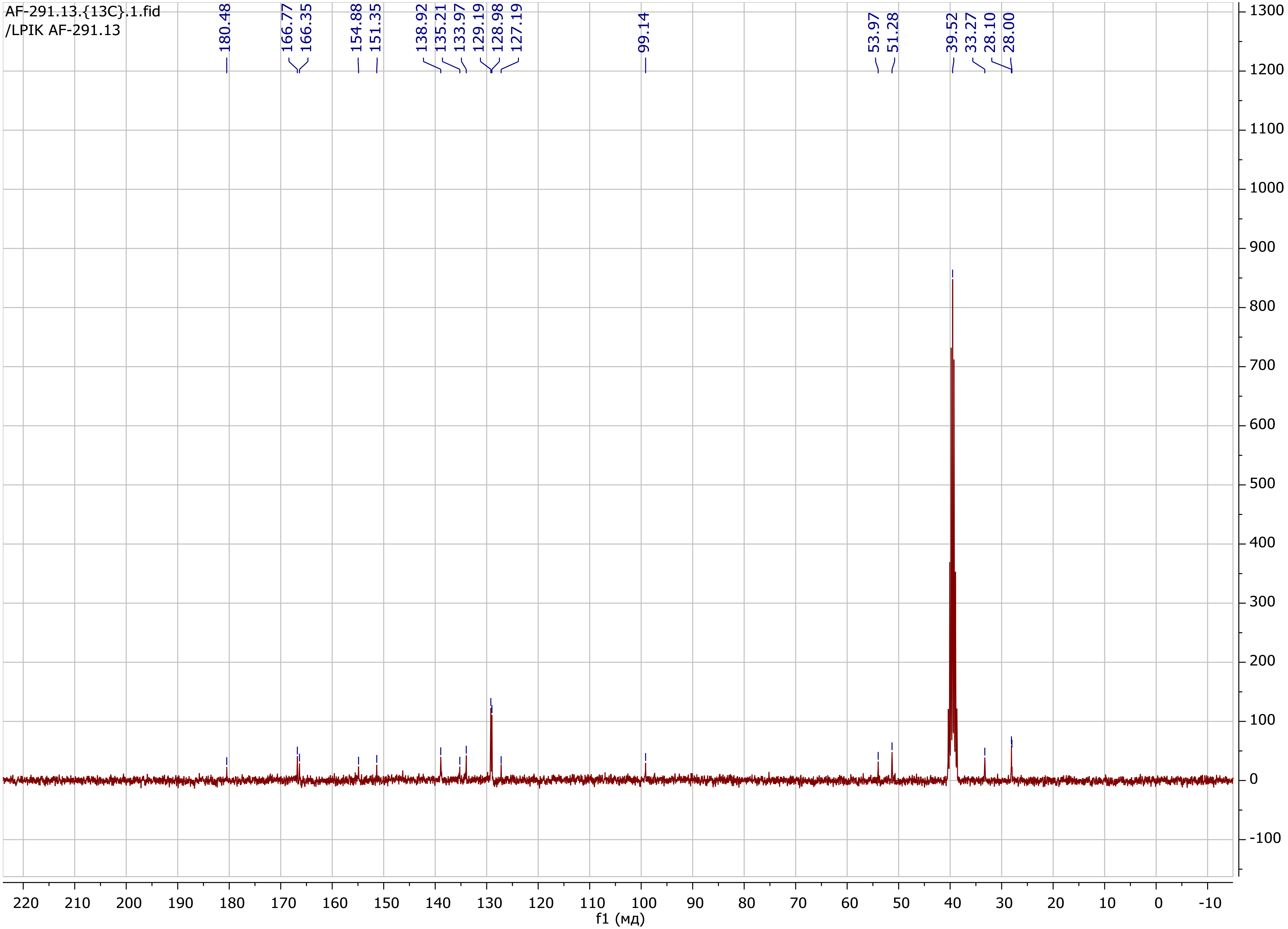
AF-291.13.{13C}.1.fid
/LPIK AF-291.13
180.48
166.77
166.35
154.88
151.35
138.92
135.21
133.97
129.19
128.98
127.19
99.14
53.97
51.28
39.52
33.27
28.10
28.00
f1 (мд)

Display Report

Analysis Info

Analysis Name	D:\Data\Kolotyrkina\2019\Bastrakov\0925008.d	Acquisition Date	25.09.2019 12:53:52
Method	tune_50-1600.m	Operator	BDAL@DE
Sample Name	/LPIK AF-291	Instrument / Ser#	micrOTOF 10248
Comment	C21H18N4O7 mH 439.1248 calibrant added		

Acquisition Parameter

Source Type	ESI	Ion Polarity	Positive	Set Nebulizer	1.0 Bar
Focus	Not active			Set Dry Heater	200 °C
Scan Begin	50 m/z	Set Capillary	4500 V	Set Dry Gas	4.0 l/min
Scan End	1600 m/z	Set End Plate Offset	-500 V	Set Divert Valve	Waste

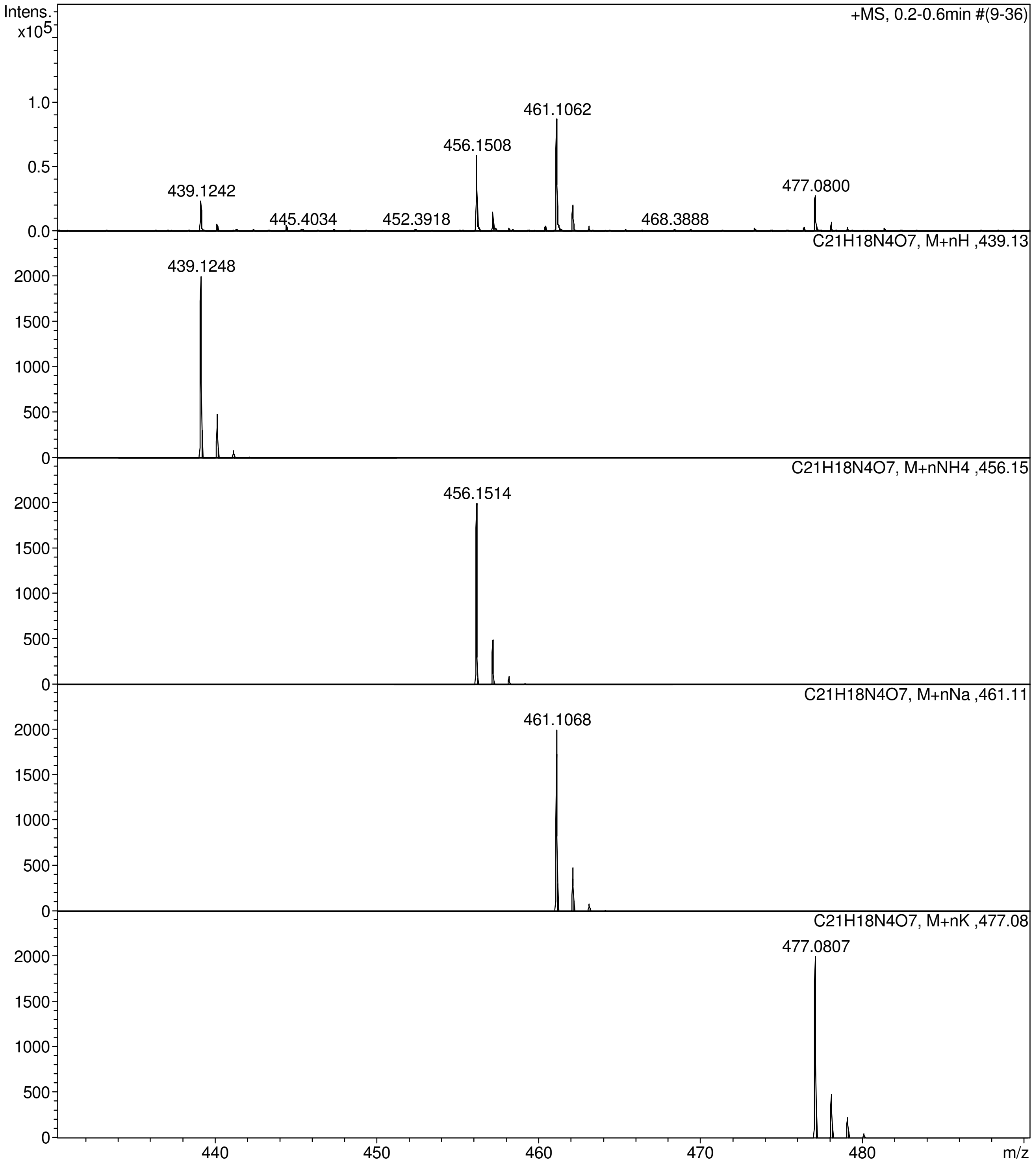

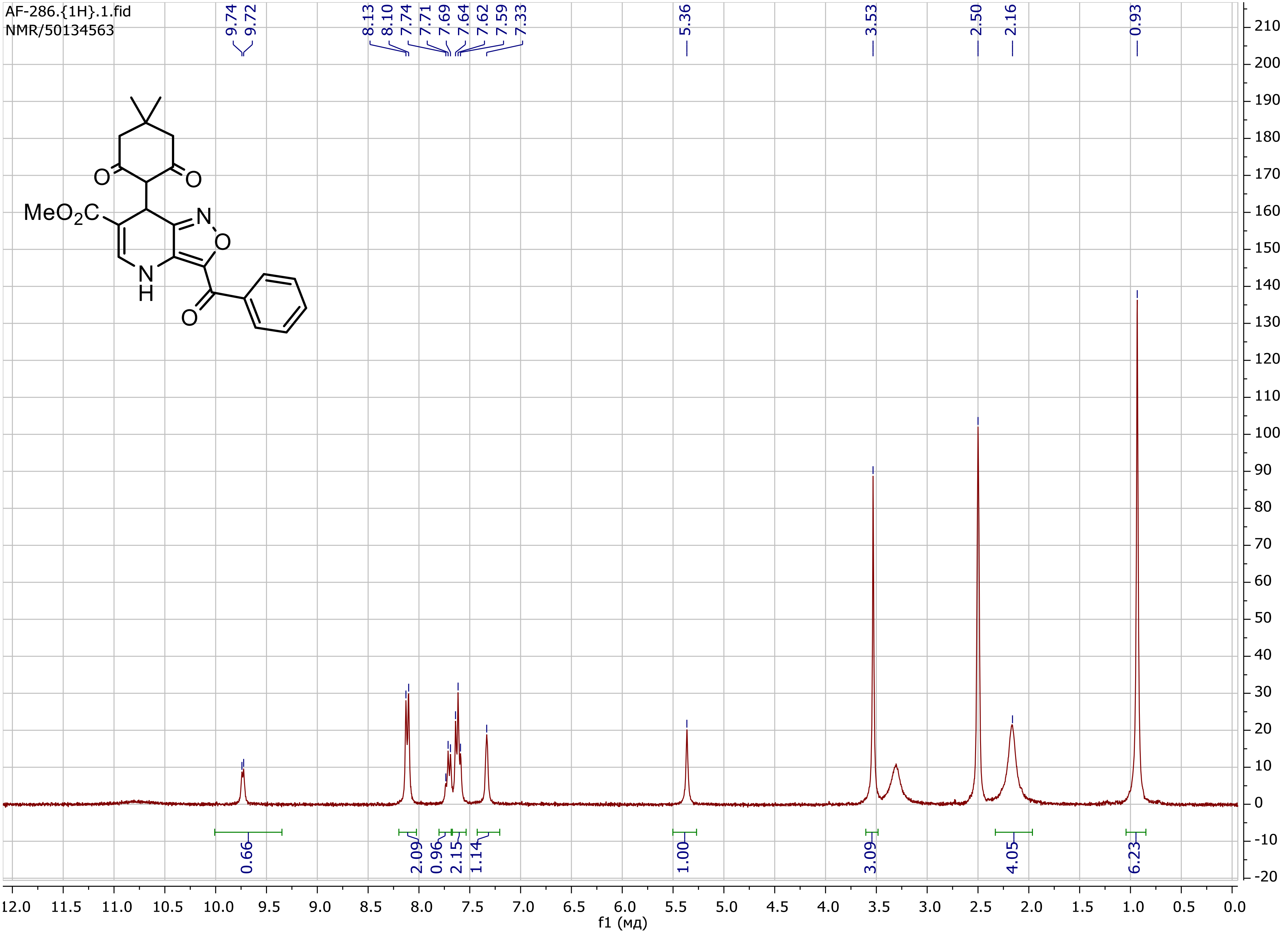

AF-286.{1H}.1.fid
NMR/50134563
9.74
9.72
8.13
8.10
7.74
7.71
7.69
7.64
7.62
7.59
7.33
5.36
3.53
2.50
2.16
0.93
MeO2C
N
O
N
H
O
O
O
0.66
2.09
0.96
2.15
1.14
1.00
3.09
4.05
6.23
f1 (мд)

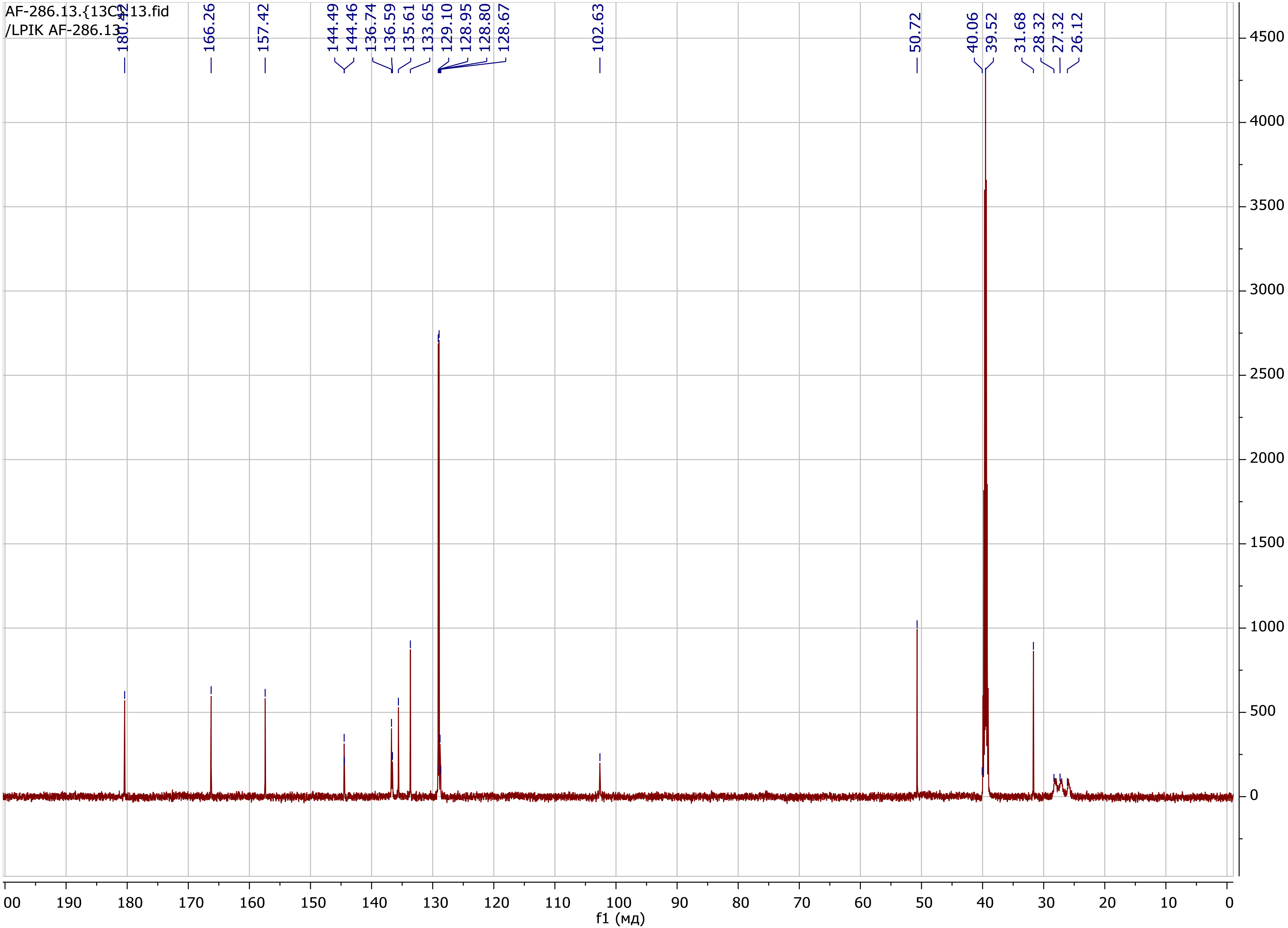
AF-286.13.{13C}13.fid
/LPIK AF-286.13
180.42
166.26
157.42
144.49
144.46
136.74
136.59
135.61
133.65
129.10
128.95
128.80
128.67
102.63
50.72
40.06
39.52
31.68
28.32
27.32
26.12
f1 (мд)

Display Report

Analysis Info

Acquisition Date 23.04.2019 12:54:40

Analysis Name D:\Data\Kolotyrkina\2019\Bastrakov\0423026.d
Method tune_50-1600.m
Sample Name /LPIK AF-286
Comment C23H22N2O6 mH 423.1550 calibrant added

Operator BDAL@DE
Instrument / Ser# micrOTOF 10248

Acquisition Parameter

Source Type	ESI	Ion Polarity	Positive	Set Nebulizer	1.0 Bar
Focus	Not active			Set Dry Heater	200 °C
Scan Begin	50 m/z	Set Capillary	4500 V	Set Dry Gas	4.0 l/min
Scan End	1600 m/z	Set End Plate Offset	-500 V	Set Divert Valve	Waste

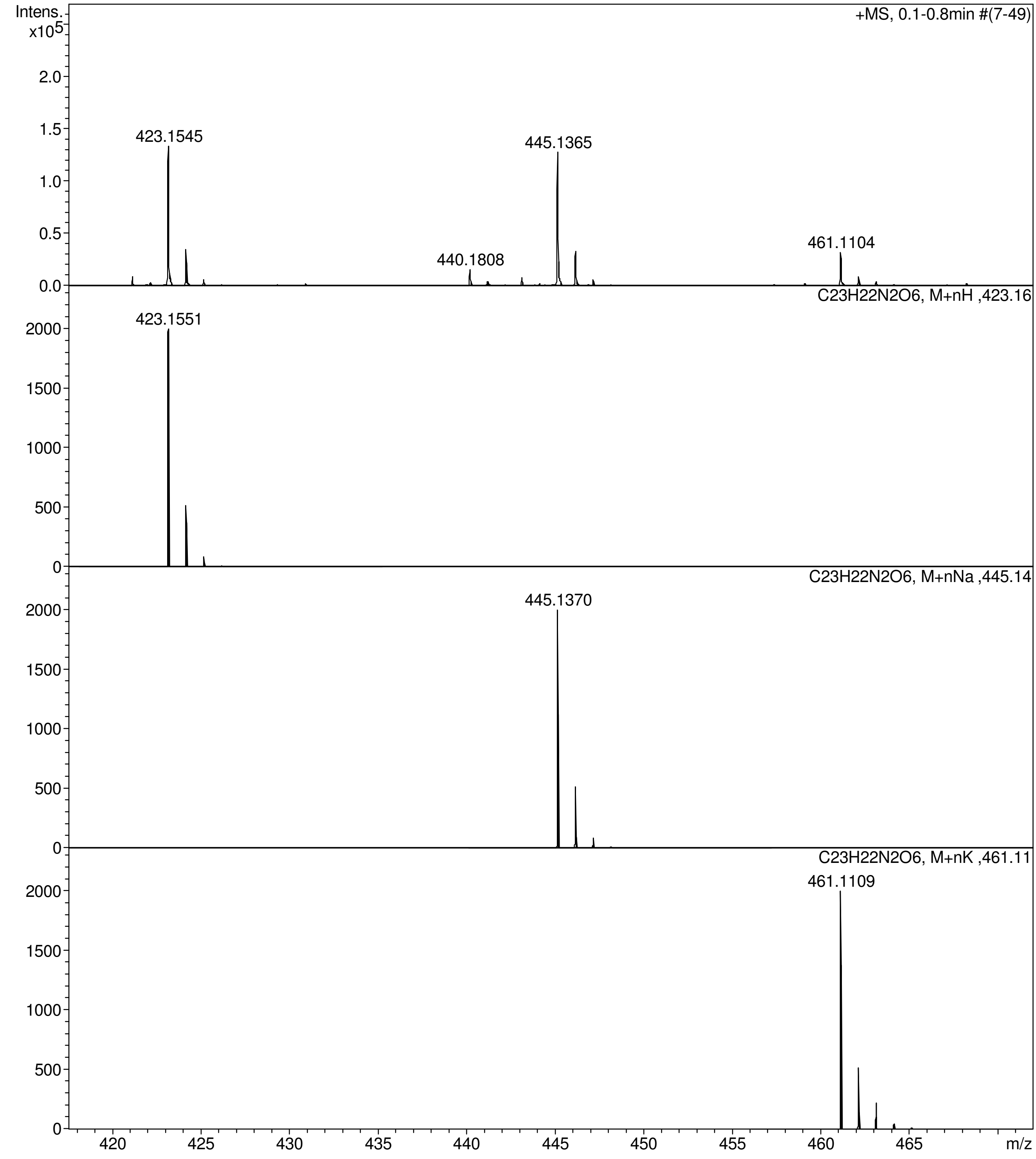

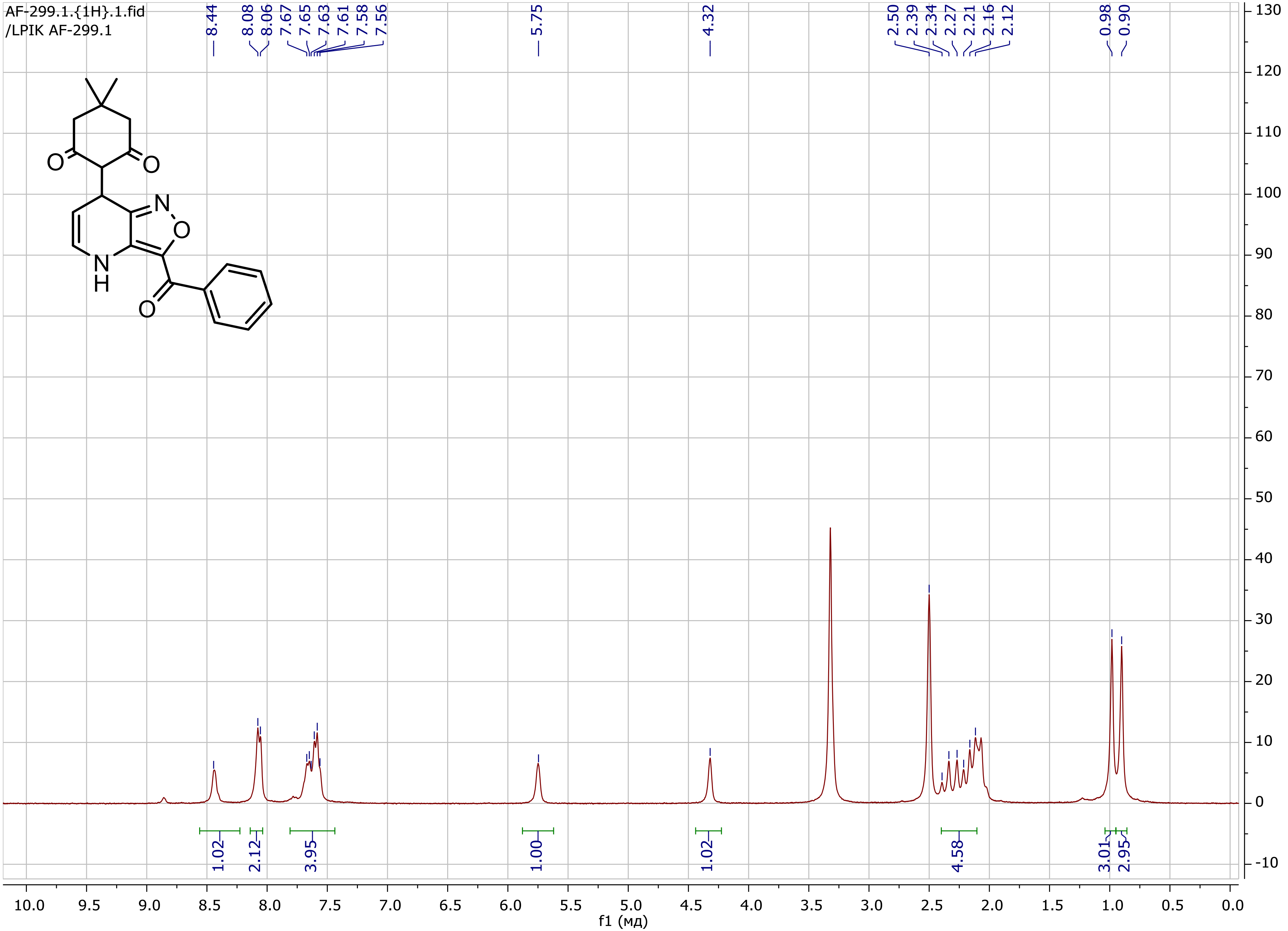
AF-299.1.{1H}.1.fid
/LPIK AF-299.1
8.44
8.08
8.06
7.67
7.65
7.63
7.61
7.58
7.56
5.75
4.32
2.50
2.39
2.34
2.27
2.21
2.16
2.12
0.98
0.90
1.02
2.12
3.95
1.00
1.02
4.58
3.01
2.95
f1 (мд)

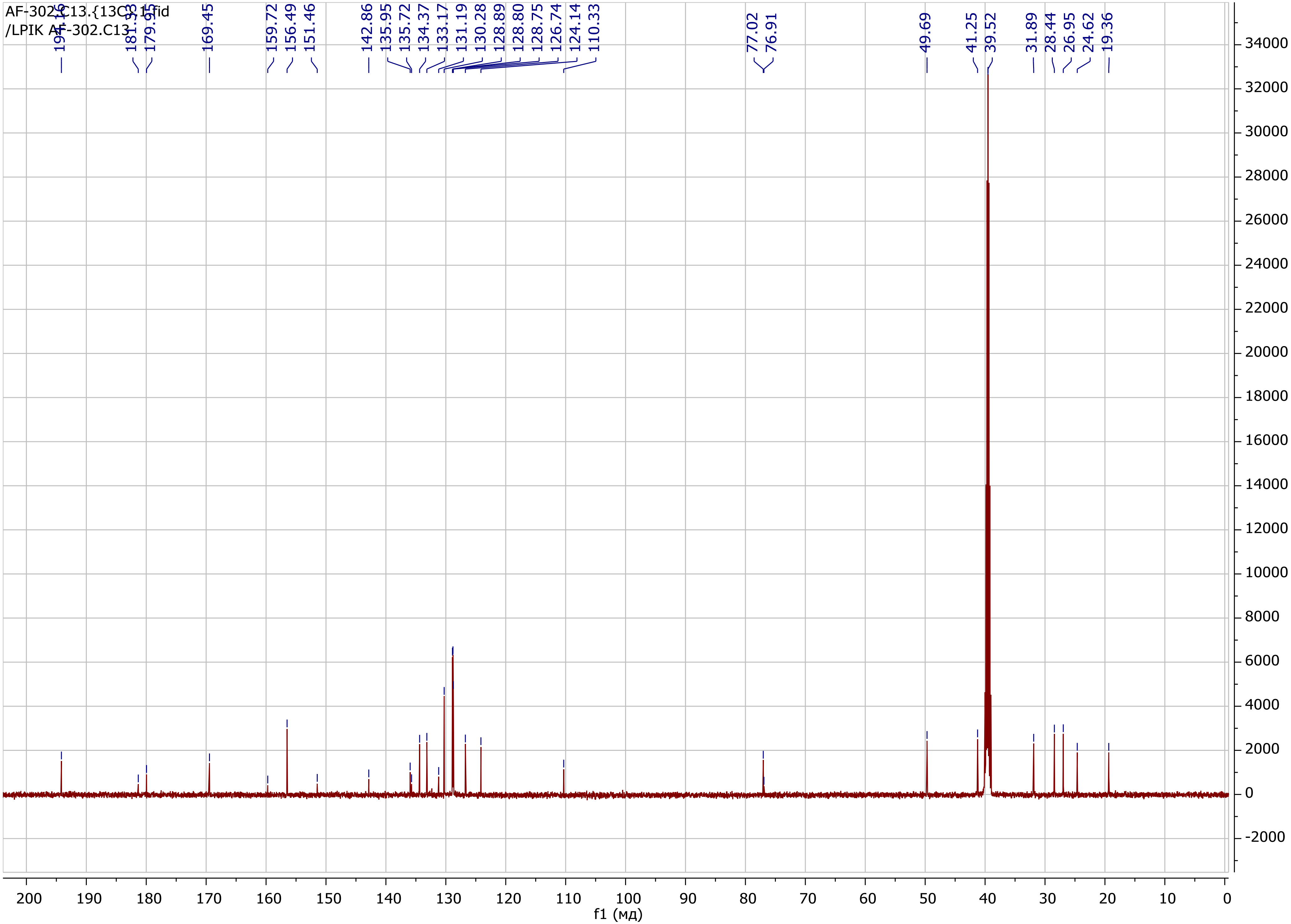
AF-302.C13.{13C}.1.fid
/LPIK AF-302.C13
194.16
181.33
179.95
169.45
159.72
156.49
151.46
142.86
135.95
135.72
134.37
133.17
131.19
130.28
128.89
128.80
128.75
126.74
124.14
110.33
77.02
76.91
49.69
41.25
39.52
31.89
28.44
26.95
24.62
19.36
f1 (мд)

Display Report

Analysis Info

Analysis Name	D:\Data\Kolotyrkina\2019\Bastrakov\0925007.d	Acquisition Date	25.09.2019 12:48:55
Method	tune_50-1600.m	Operator	BDAL@DE
Sample Name	/LPIK AF-302	Instrument / Ser#	micrOTOF 10248
Comment	C21H20N2O4 mH 365.1495 calibrant added		

Acquisition Parameter

Source Type	ESI	Ion Polarity	Positive	Set Nebulizer	1.0 Bar
Focus	Not active			Set Dry Heater	200 °C
Scan Begin	50 m/z	Set Capillary	4500 V	Set Dry Gas	4.0 l/min
Scan End	1600 m/z	Set End Plate Offset	-500 V	Set Divert Valve	Waste

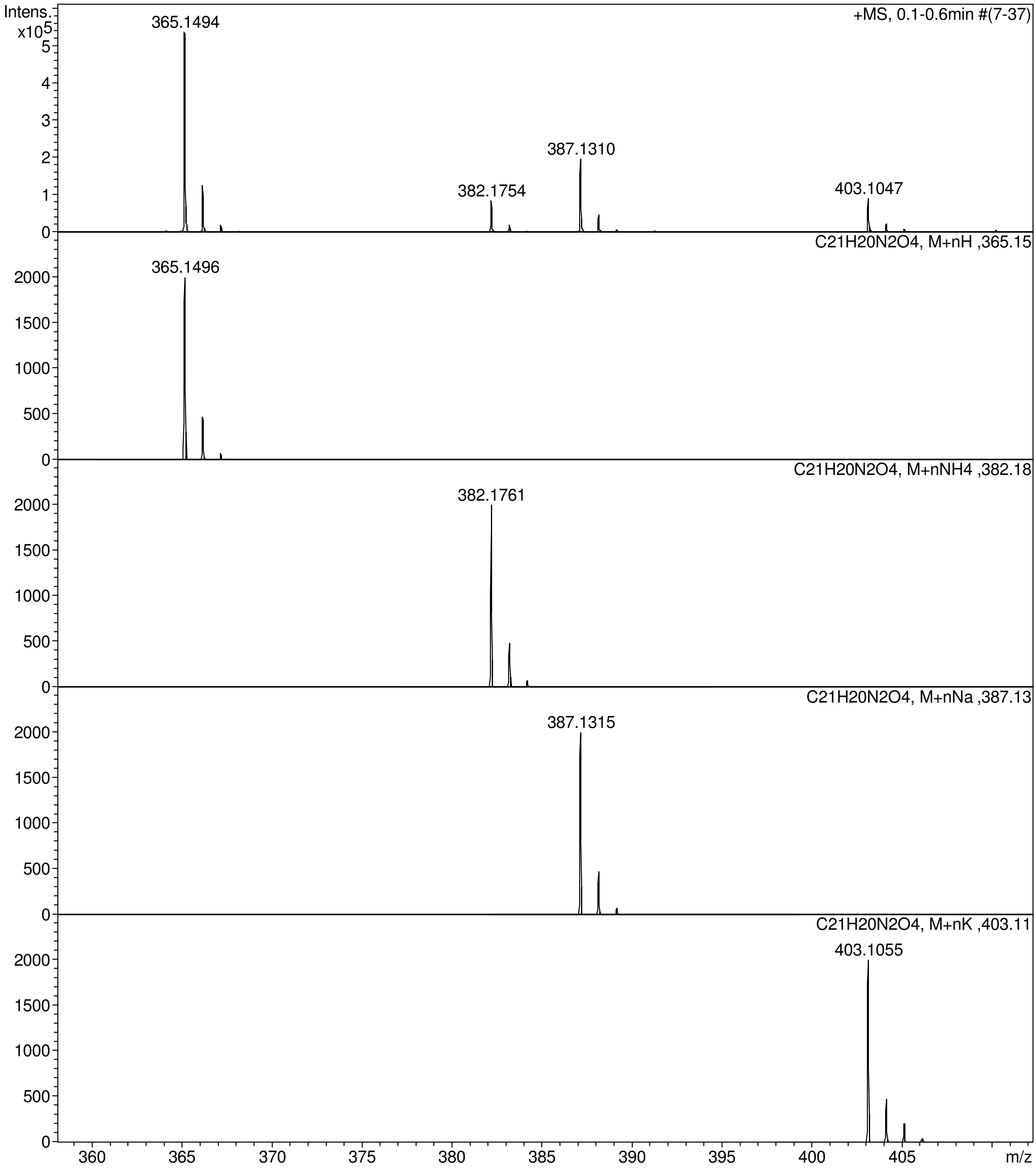

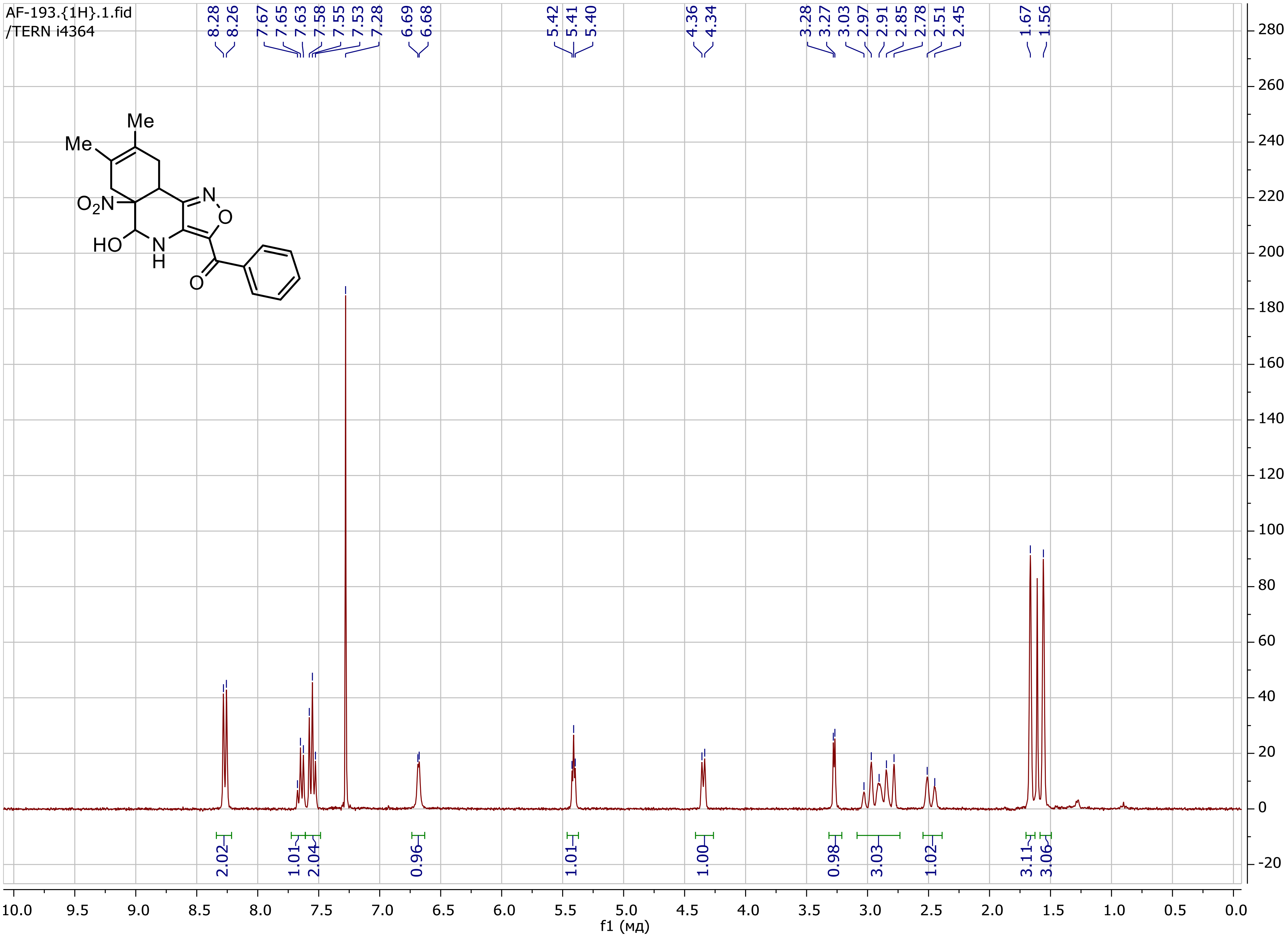

AF-193.{1H}.1.fid
/TERN i4364
8.28
8.26
7.67
7.65
7.63
7.58
7.55
7.53
7.28
6.69
6.68
5.42
5.41
5.40
4.36
4.34
3.28
3.27
3.03
2.97
2.91
2.85
2.78
2.51
2.45
1.67
1.56
Me
Me
O2N
N
O
HO
N
H
O
2.02
1.01
2.04
0.96
1.01
1.00
0.98
3.03
1.02
3.11
3.06
f1 (мд)

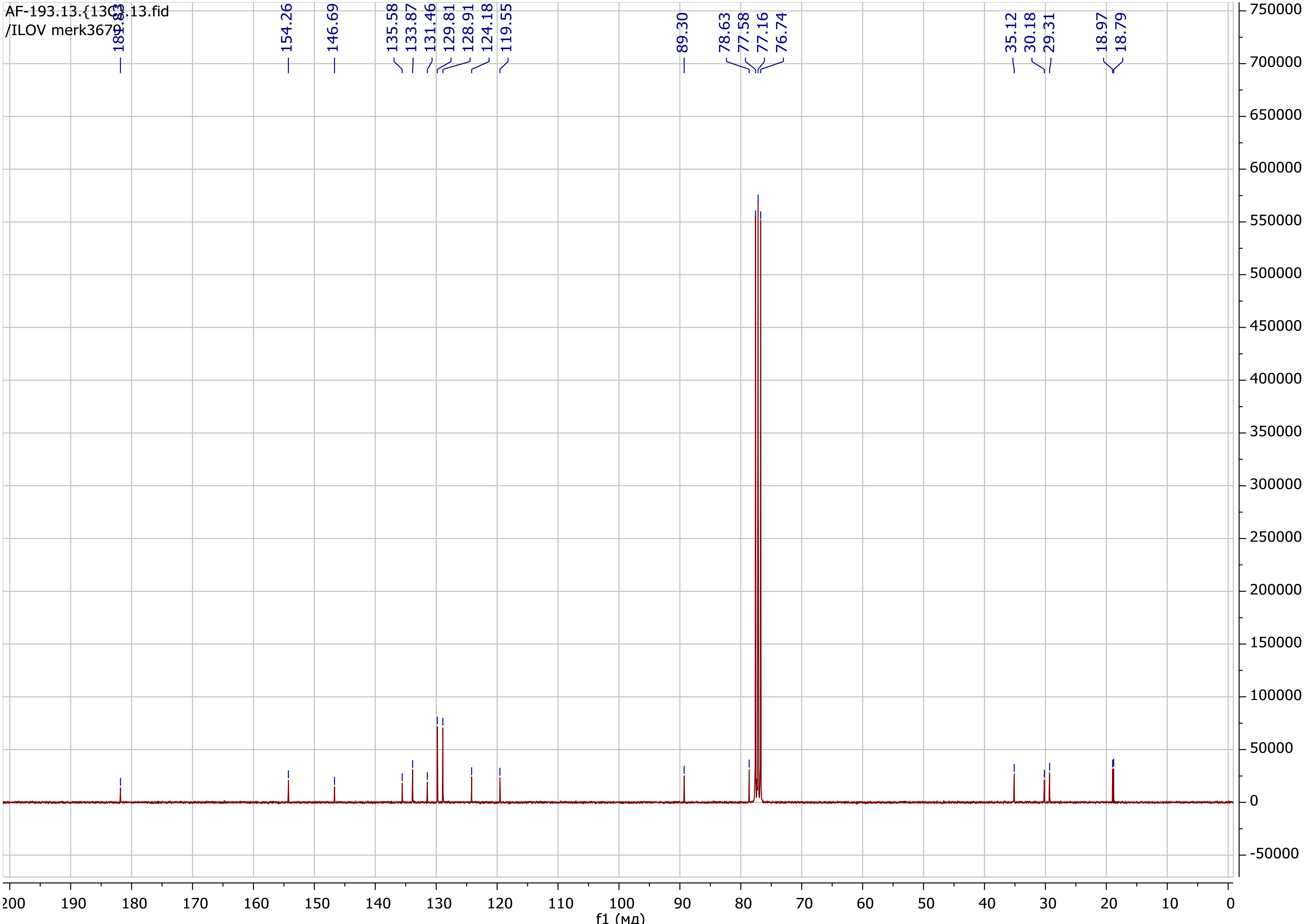
AF-193.13.{13C}.13.fid
/ILOV merk3679
154.26
146.69
135.58
133.87
131.46
129.81
128.91
124.18
119.55
89.30
78.63
77.58
77.16
76.74
35.12
30.18
29.31
18.97
18.79
f1 (мд)

Display Report

Analysis Info

Analysis Name	D:\Data\Kolotyrkina\2018\Bastrakov\1113020.d	Acquisition Date	13.11.2018 12:05:43
Method	tune_50-1600.m	Operator	BDAL@DE
Sample Name	/LPIK AF-193	Instrument / Ser#	micrOTOF 10248
Comment	C19H19N3O5 mH 370.1397 calibrant added		

Acquisition Parameter

Source Type	ESI	Ion Polarity	Positive	Set Nebulizer	1.0 Bar
Focus	Not active			Set Dry Heater	200 °C
Scan Begin	50 m/z	Set Capillary	4500 V	Set Dry Gas	4.0 l/min
Scan End	1600 m/z	Set End Plate Offset	-500 V	Set Divert Valve	Waste

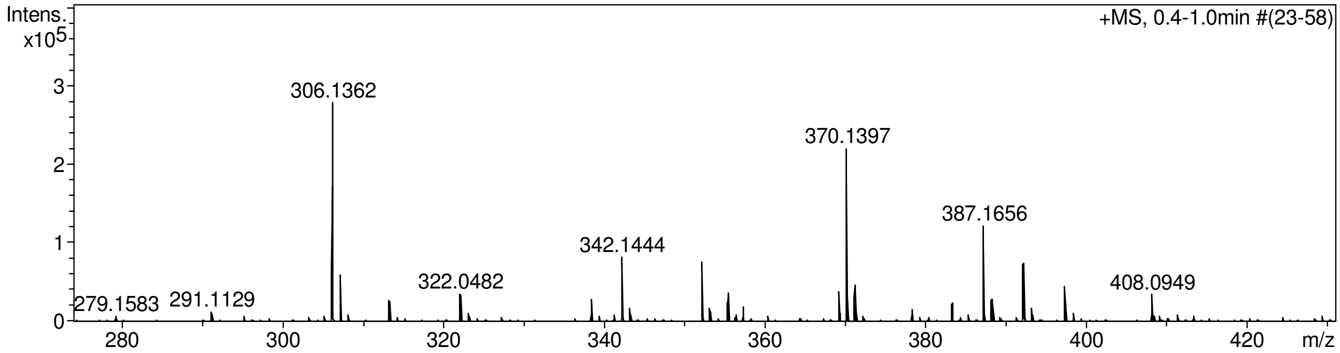

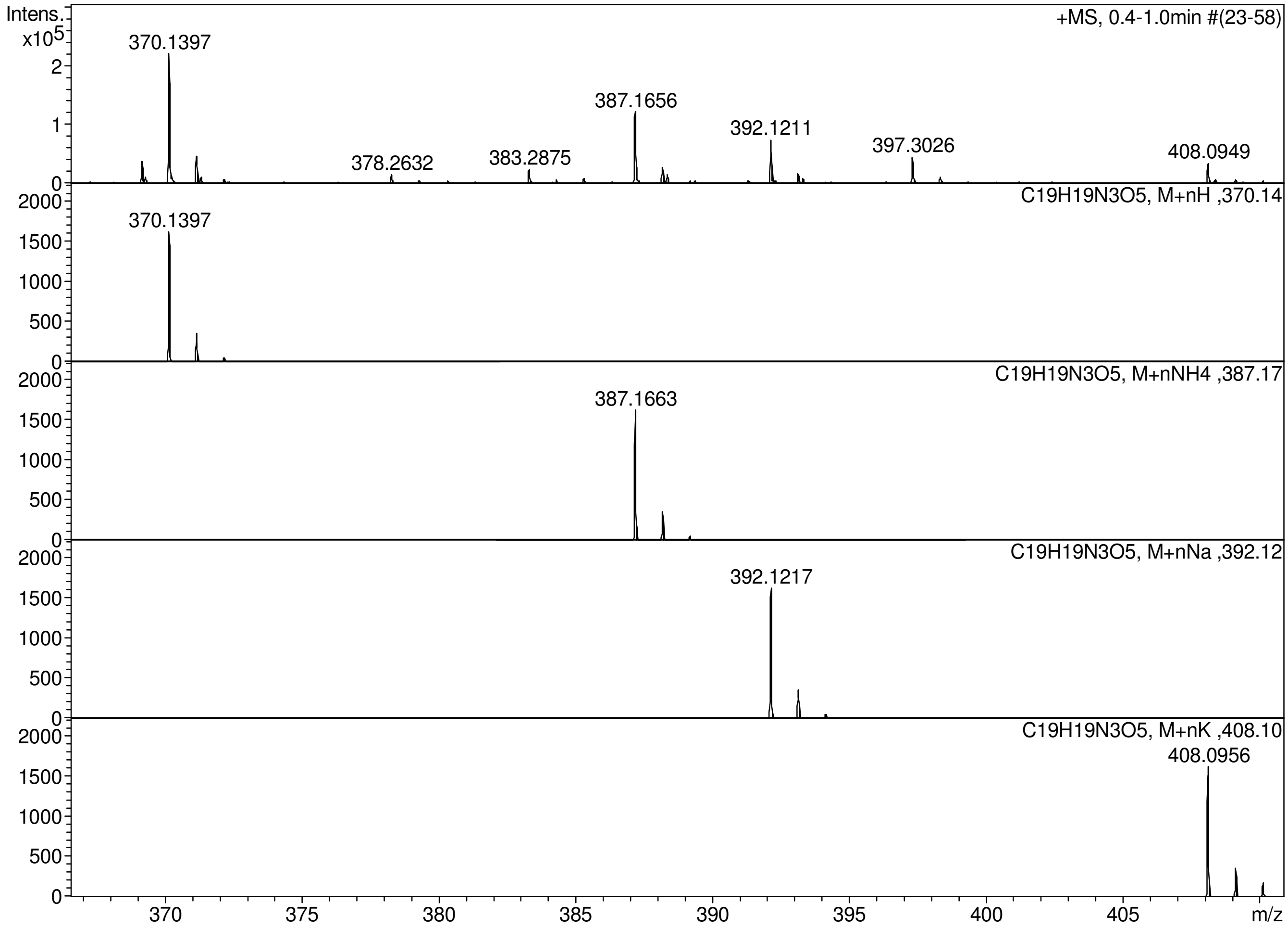

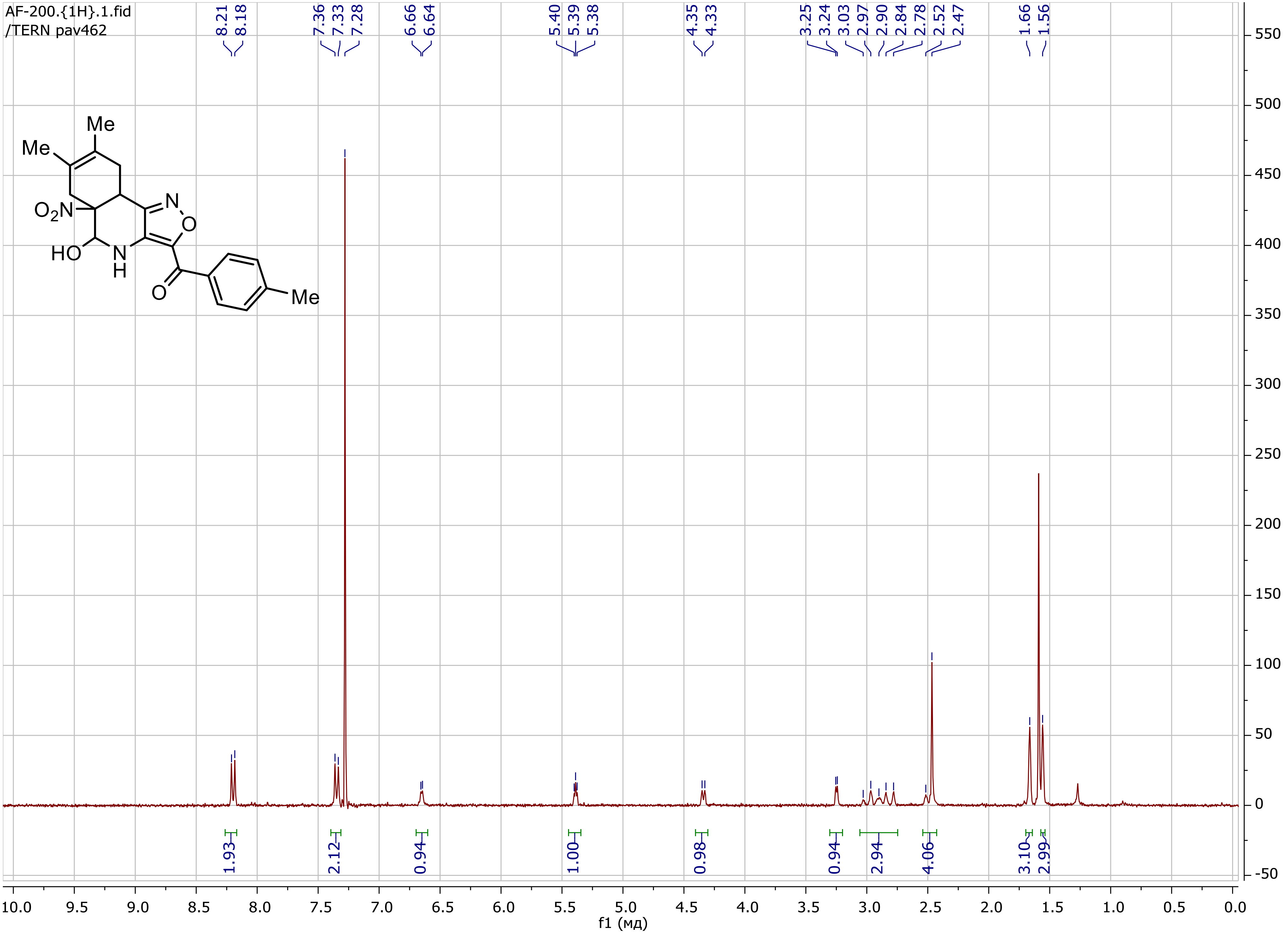
AF-200.{1H}.1.fid
/TERN pav462
8.21
8.18
7.36
7.33
7.28
6.66
6.64
5.40
5.39
5.38
4.35
4.33
3.25
3.24
3.03
2.97
2.90
2.84
2.78
2.52
2.47
1.66
1.56
Me
Me
O_2N
N
O
HO
N
H
O
Me
1.93
2.12
0.94
1.00
0.98
0.94
2.94
4.06
3.10
2.99
f1 (мд)

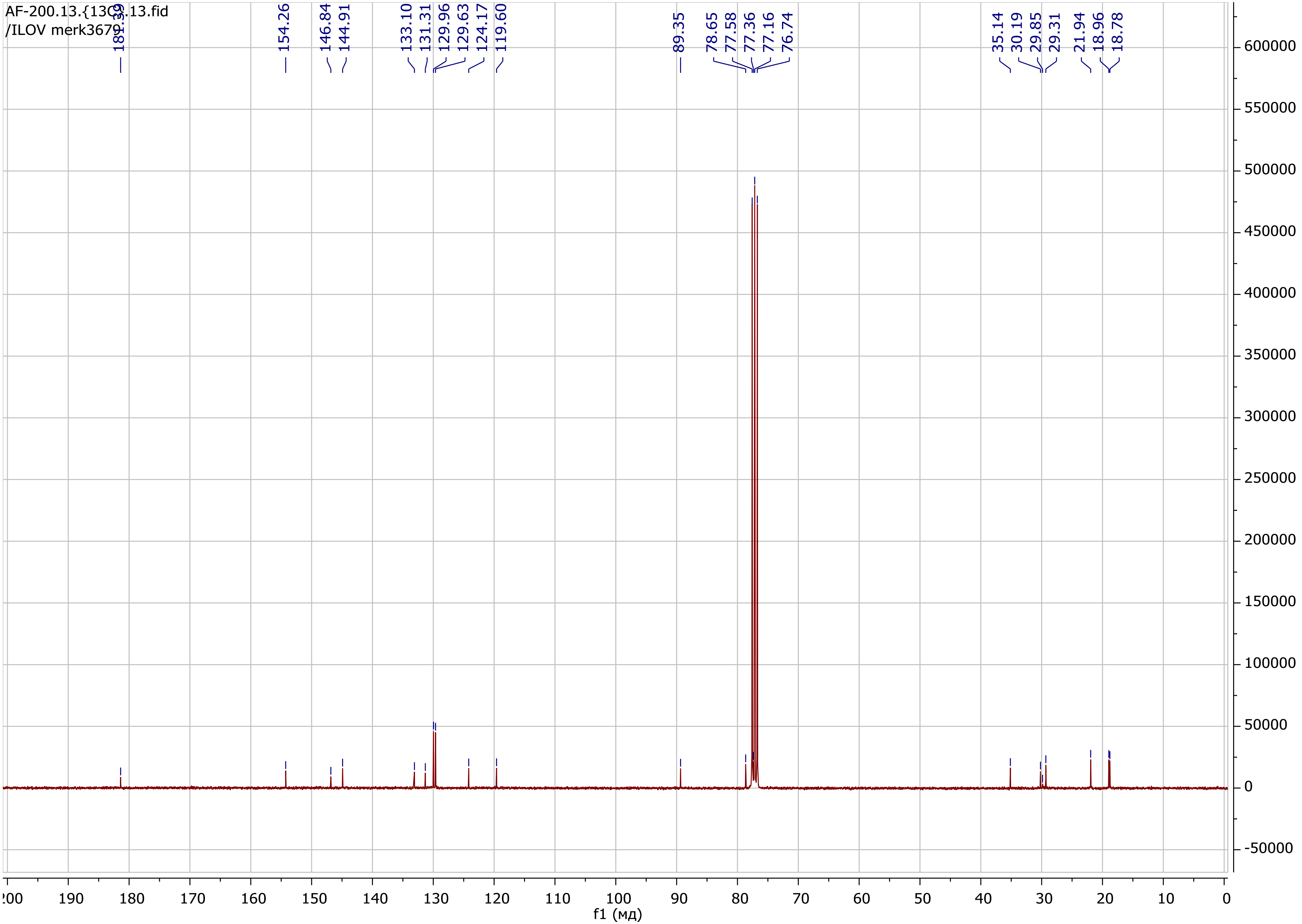
AF-200.13.{13C}.13.fid
/ILOV merk3679
181.39
154.26
146.84
144.91
133.10
131.31
129.96
129.63
124.17
119.60
89.35
78.65
77.58
77.36
77.16
76.74
35.14
30.19
29.85
29.31
21.94
18.96
18.78
600000
550000
500000
450000
400000
350000
300000
250000
200000
150000
100000
50000
0
-50000
200
190
180
170
160
150
140
130
120
110
100
90
80
70
60
50
40
30
20
10
0
f1 (мд)

Display Report

Analysis Info

Analysis Name	D:\Data\Kolotyrkina\2018\Bastrakov\1113019.d	Acquisition Date	13.11.2018 11:57:14
Method	tune_50-1600.m	Operator	BDAL@DE
Sample Name	/LPIK AF-200	Instrument / Ser#	micrOTOF 10248
Comment	C20H21N3O5 mH 384.1553 calibrant added		

Acquisition Parameter

Source Type	ESI	Ion Polarity	Positive	Set Nebulizer	1.0 Bar
Focus	Not active			Set Dry Heater	200 °C
Scan Begin	50 m/z	Set Capillary	4500 V	Set Dry Gas	4.0 l/min
Scan End	1600 m/z	Set End Plate Offset	-500 V	Set Divert Valve	Waste

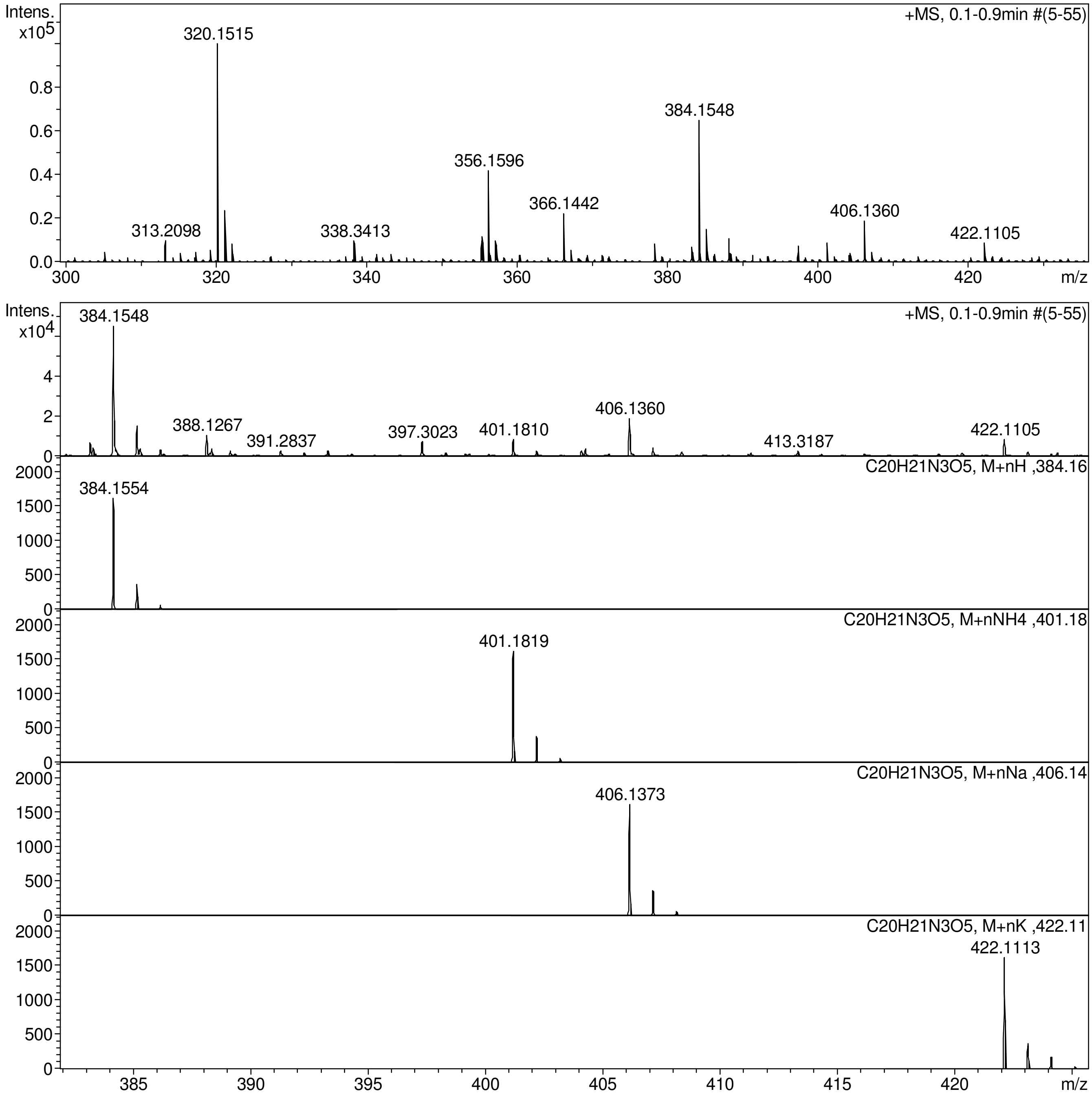

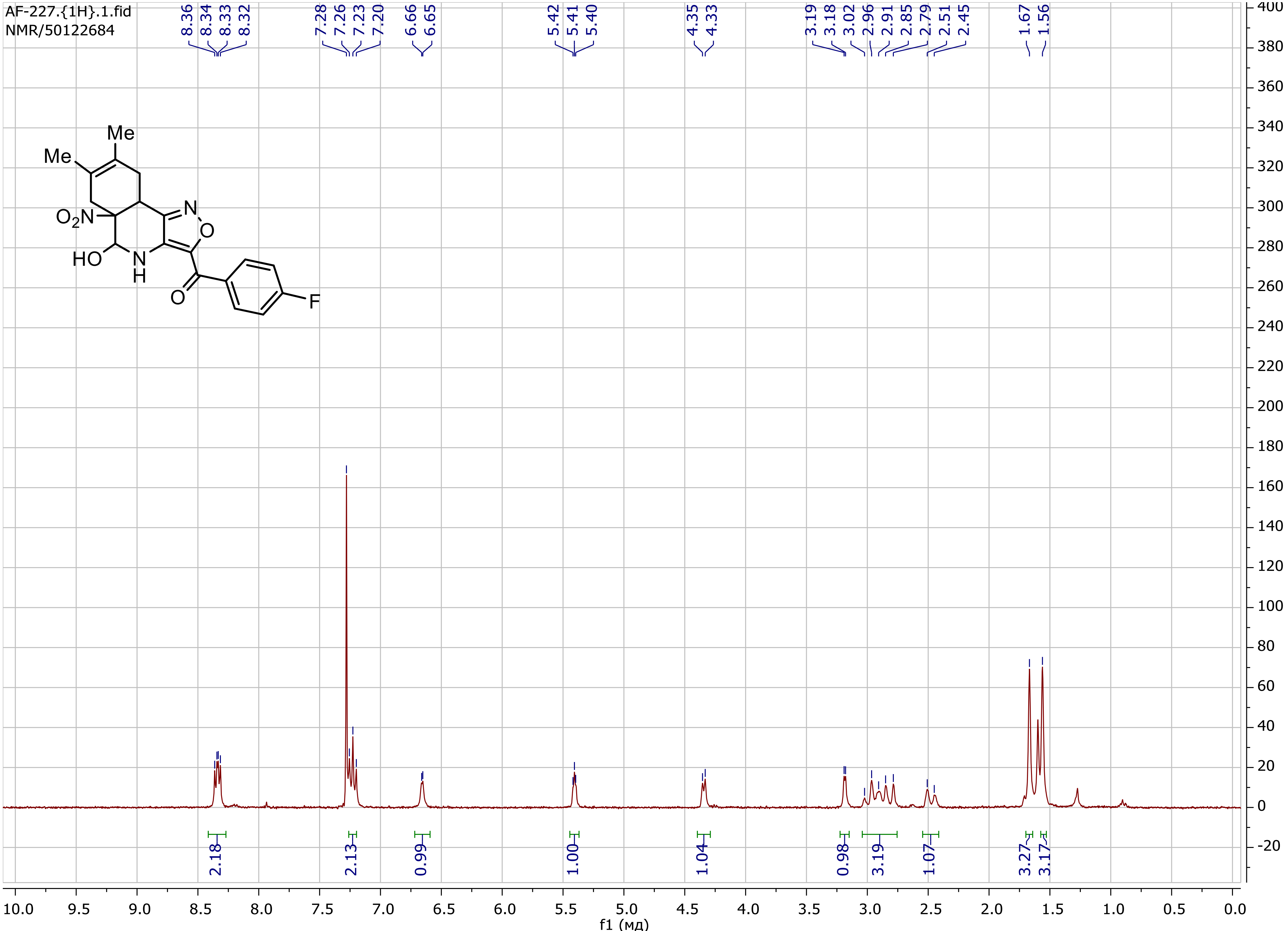

AF-227.{1H}.1.fid
NMR/50122684
8.36 8.34 8.33 8.32
7.28 7.26 7.23 7.20
6.66 6.65
5.42 5.41 5.40
4.35 4.33
3.19 3.18 3.02 2.96 2.91 2.85 2.79 2.51 2.45
1.67 1.56
Me
Me
O_2N
N
O
HO
N
H
O
F
2.18
2.13
0.99
1.00
1.04
0.98
3.19
1.07
3.27
3.17
f1 (мд)

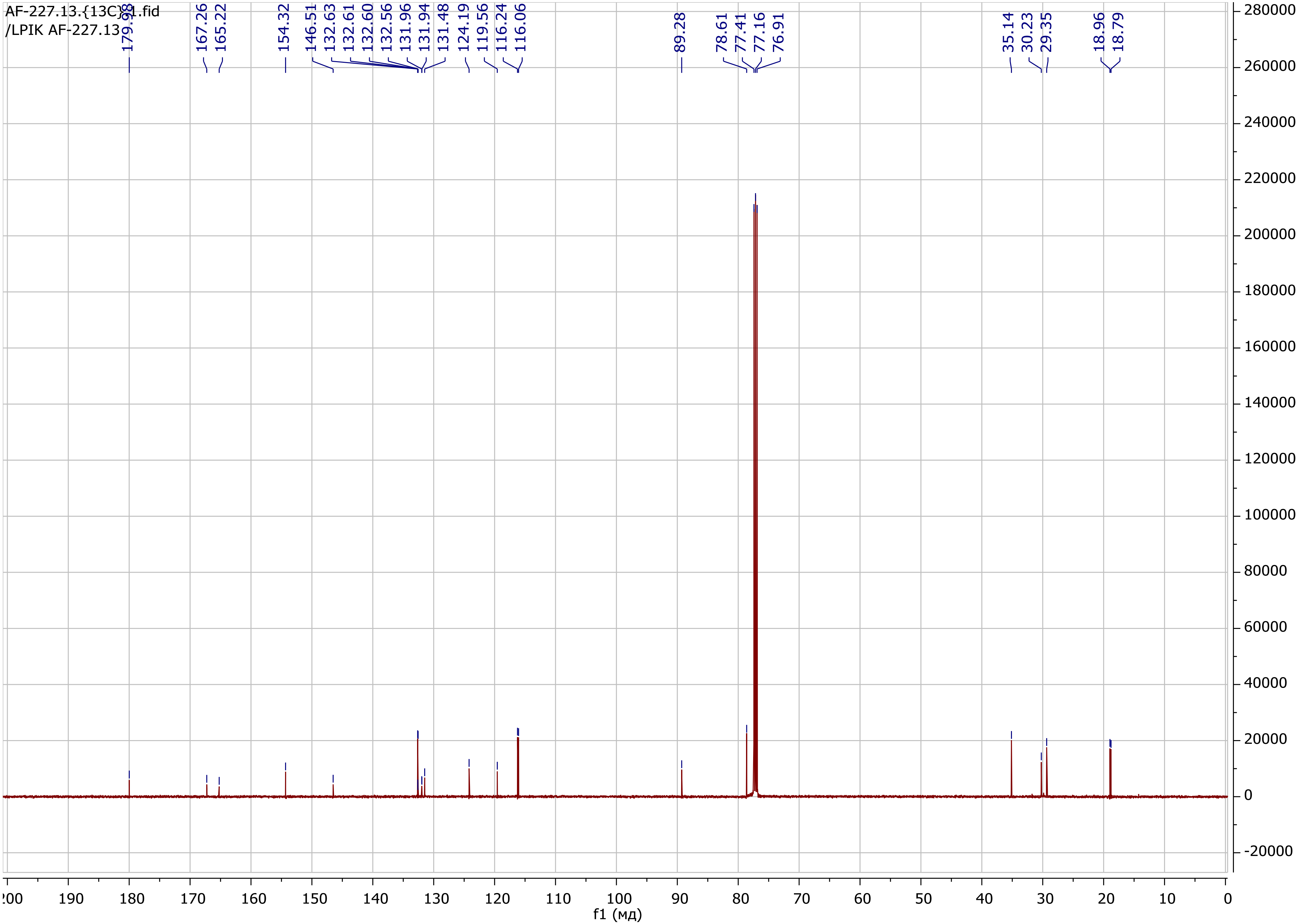

AF-227.13.{13C}1.fid
/LPIK AF-227.13
179.98
167.26
165.22
154.32
146.51
132.63
132.61
132.60
132.56
131.96
131.94
131.48
124.19
119.56
116.24
116.06
89.28
78.61
77.41
77.16
76.91
35.14
30.23
29.35
18.96
18.79
f1 (мд)

Display Report

Analysis Info

Analysis Name	D:\Data\Kolotyrkina\2019\Bastrakov\0326042.d	Acquisition Date	26.03.2019 19:28:30
Method	tune_50-1600.m	Operator	BDAL@DE
Sample Name	/LPIK AF-227	Instrument / Ser#	micrOTOF 10248
Comment	C19H18FN3O5 mH 388.1303 calibrant added CH3CN		

Acquisition Parameter

Source Type	ESI	Ion Polarity	Positive	Set Nebulizer	1.0 Bar
Focus	Not active			Set Dry Heater	200 °C
Scan Begin	50 m/z	Set Capillary	4500 V	Set Dry Gas	4.0 l/min
Scan End	1600 m/z	Set End Plate Offset	-500 V	Set Divert Valve	Waste

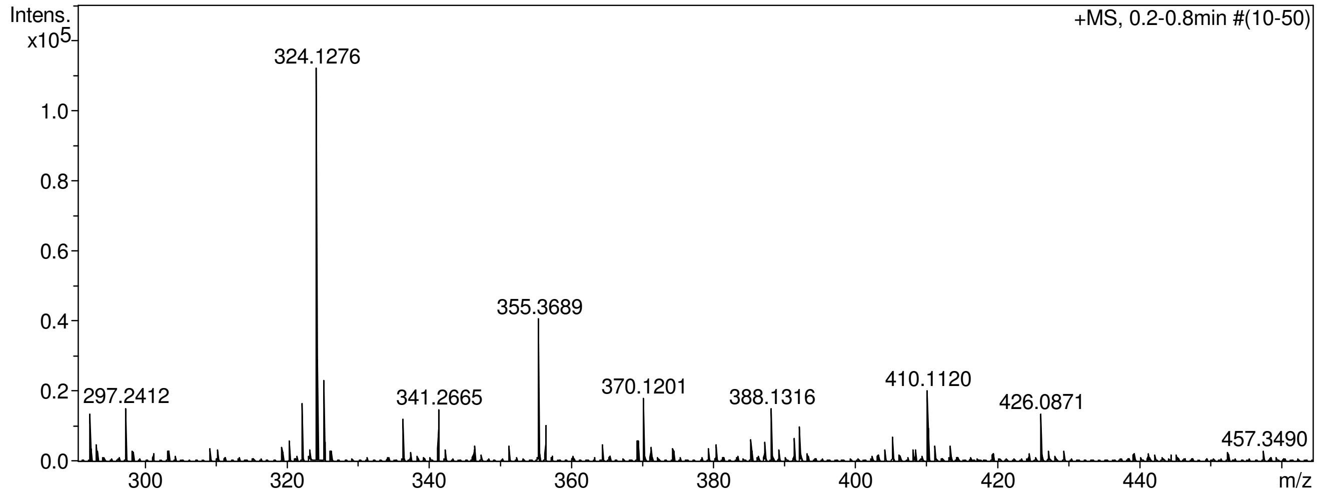

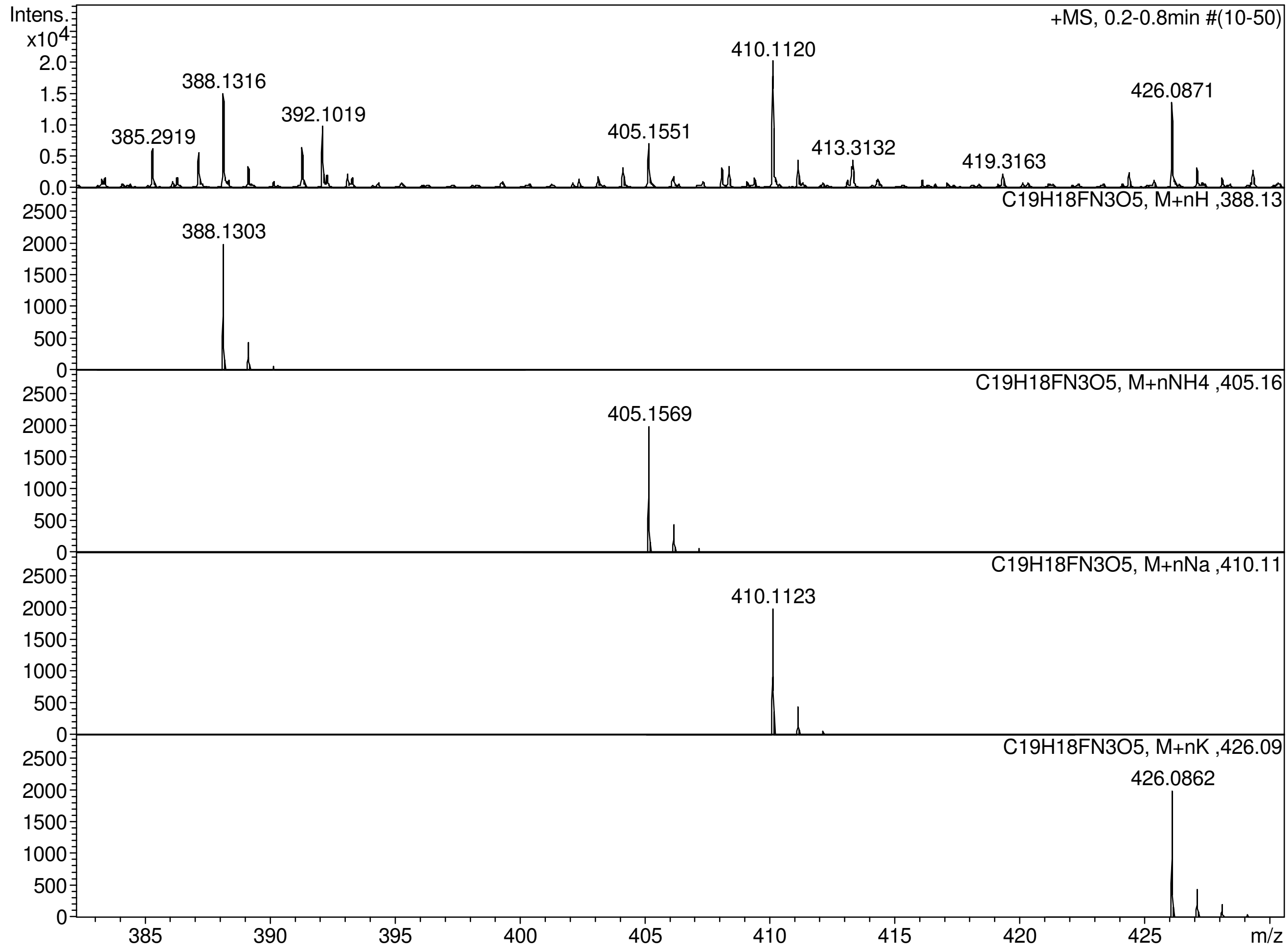

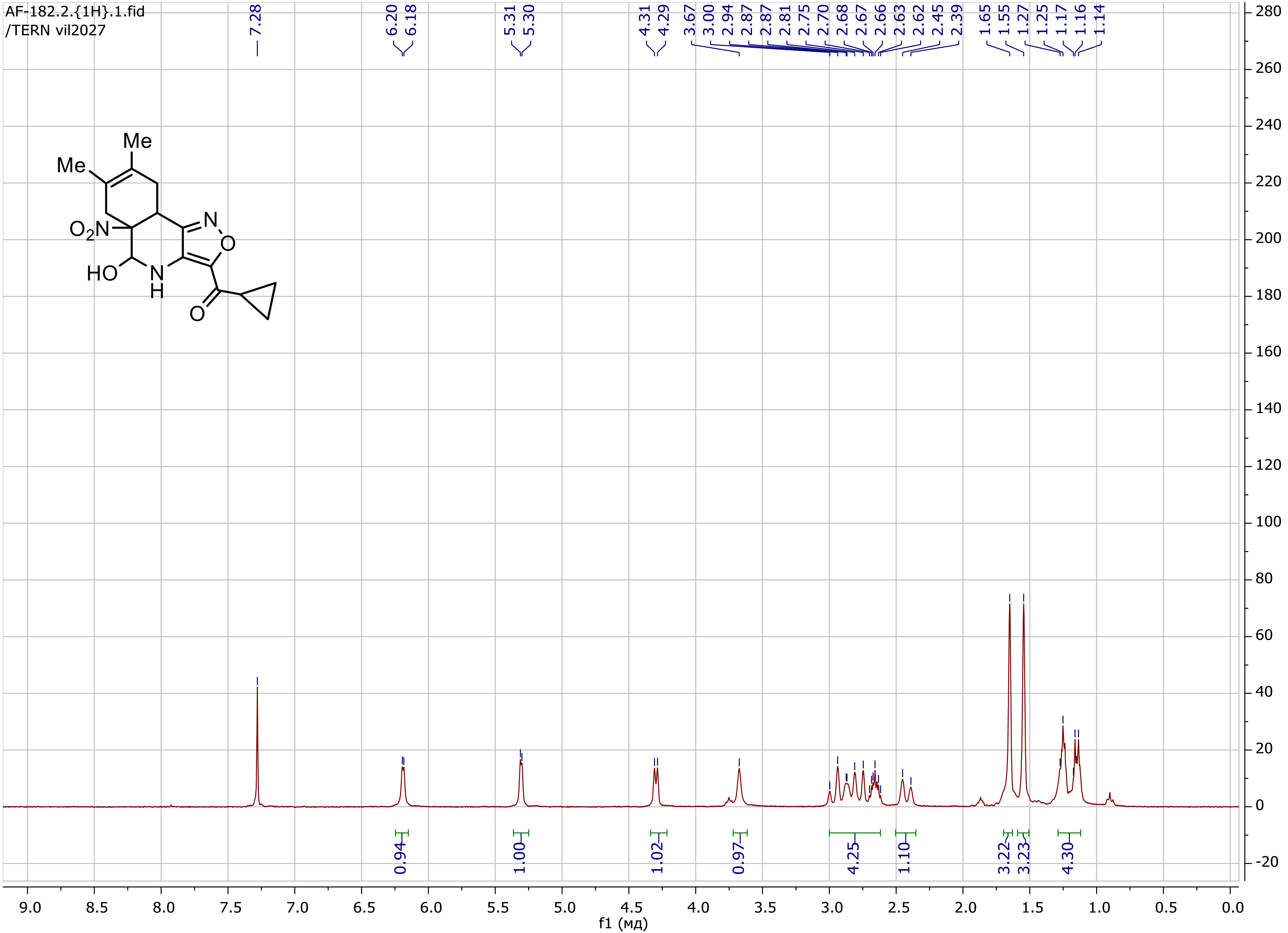
AF-182.2.{1H}.1.fid
/TERN vil2027
7.28
6.20
6.18
5.31
5.30
4.31
4.29
3.67
3.00
2.94
2.87
2.87
2.81
2.75
2.70
2.68
2.67
2.66
2.63
2.62
2.45
2.39
1.65
1.55
1.27
1.25
1.17
1.16
1.14
Me
Me
O2N
N
O
HO
N
H
O
0.94
1.00
1.02
0.97
4.25
1.10
3.22
3.23
4.30
9.0
8.5
8.0
7.5
7.0
6.5
6.0
5.5
5.0
4.5
4.0
3.5
3.0
2.5
2.0
1.5
1.0
0.5
0.0
f1 (мд)
280
260
240
220
200
180
160
140
120
100
80
60
40
20
0
-20

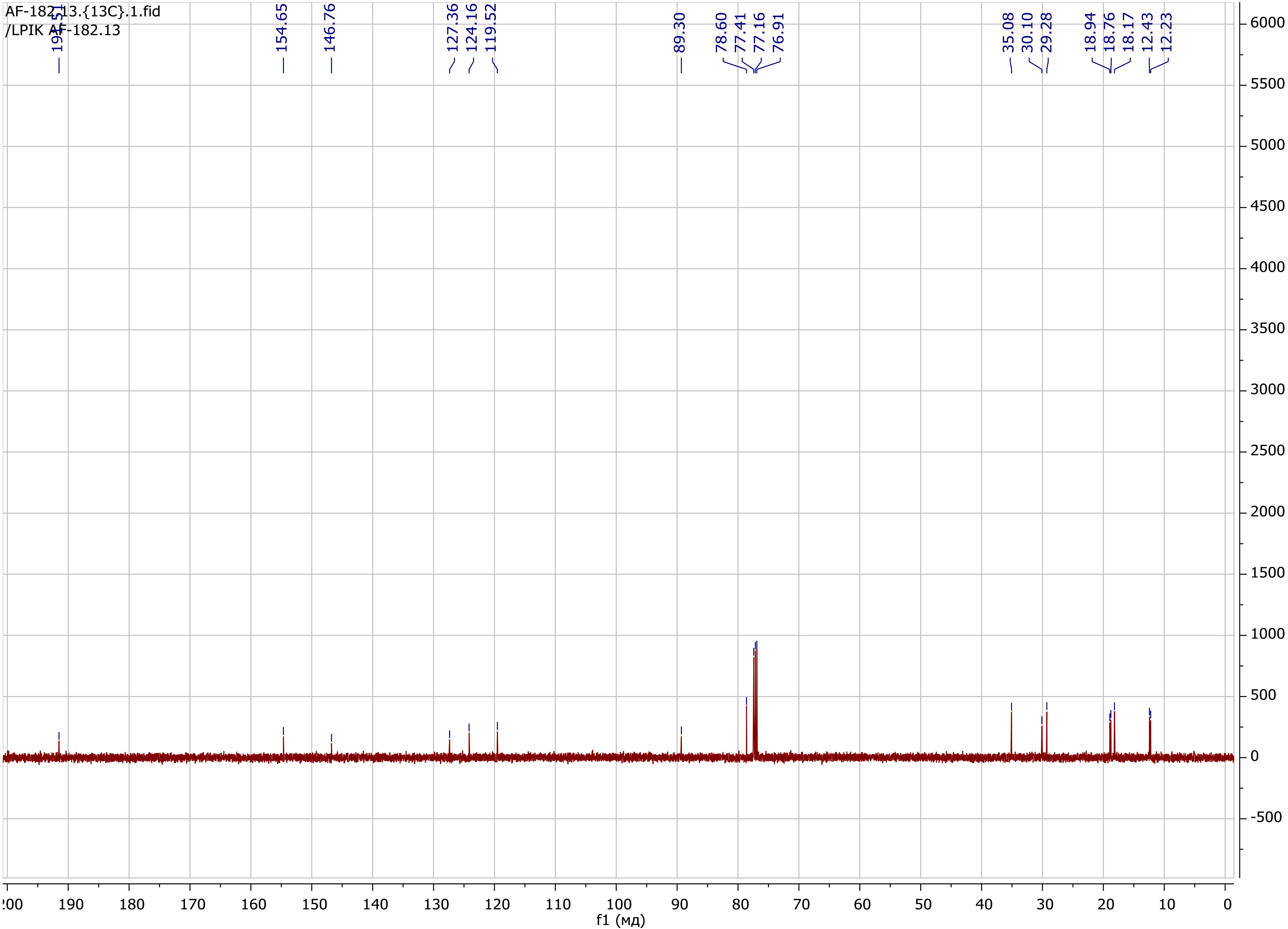
AF-182.13.{13C}.1.fid
/LPIK AF-182.13
191.51
154.65
146.76
127.36
124.16
119.52
89.30
78.60
77.41
77.16
76.91
35.08
30.10
29.28
18.94
18.76
18.17
12.43
12.23
f1 (мд)

Display Report

Analysis Info

Analysis Name	D:\Data\Kolotyrkina\2018\Bastrakov\0703006.d	Acquisition Date	03.07.2018 11:09:21
Method	tune_50-1600.m	Operator	BDAL@DE
Sample Name	/LPIK AF-182	Instrument / Ser#	micrOTOF 10248
Comment	C16H19N3O5 mH 334.1397 calibrant added		

Acquisition Parameter

Source Type	ESI	Ion Polarity	Positive	Set Nebulizer	1.0 Bar
Focus	Not active			Set Dry Heater	200 °C
Scan Begin	50 m/z	Set Capillary	4500 V	Set Dry Gas	4.0 l/min
Scan End	1600 m/z	Set End Plate Offset	-500 V	Set Divert Valve	Waste

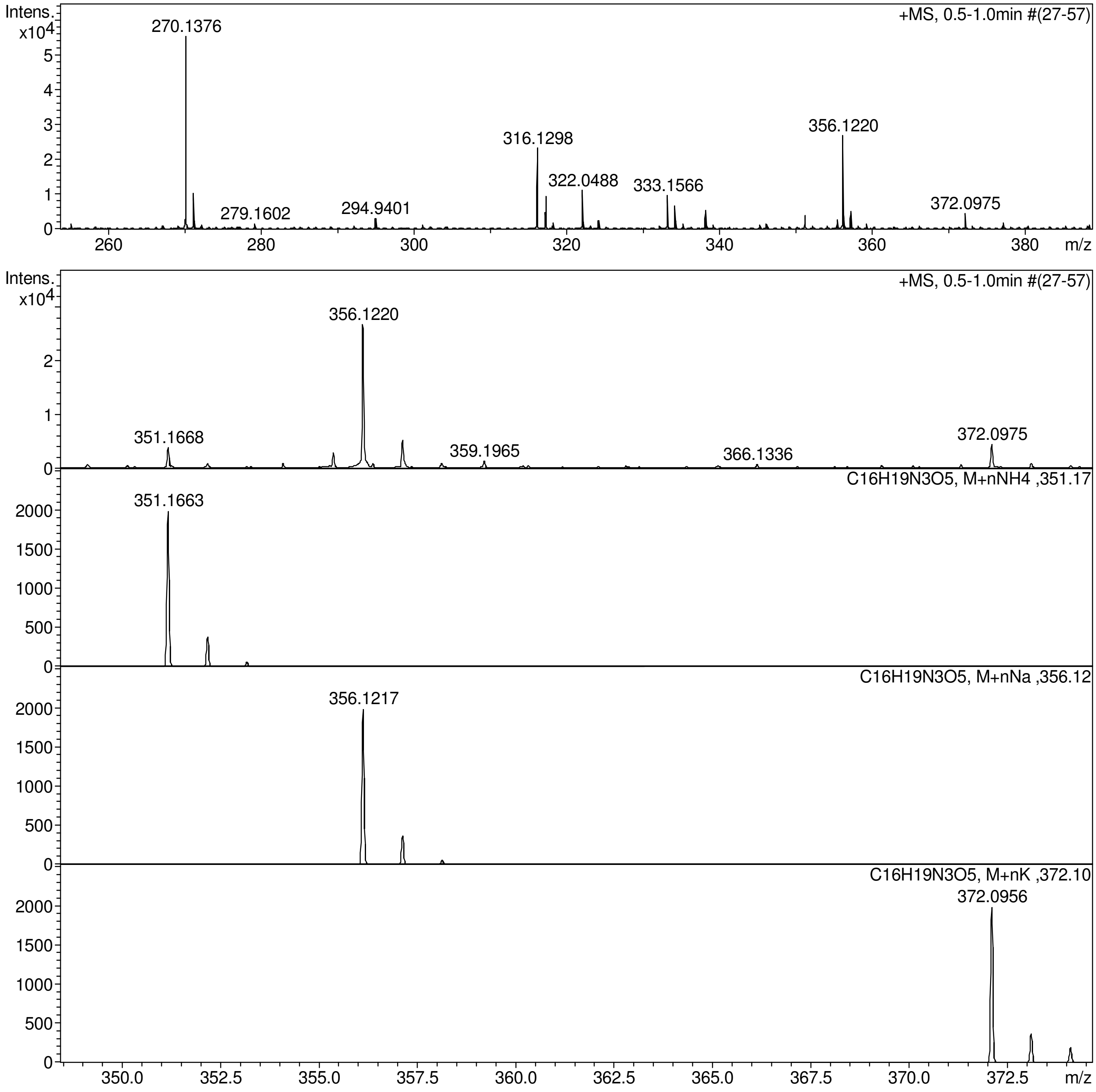

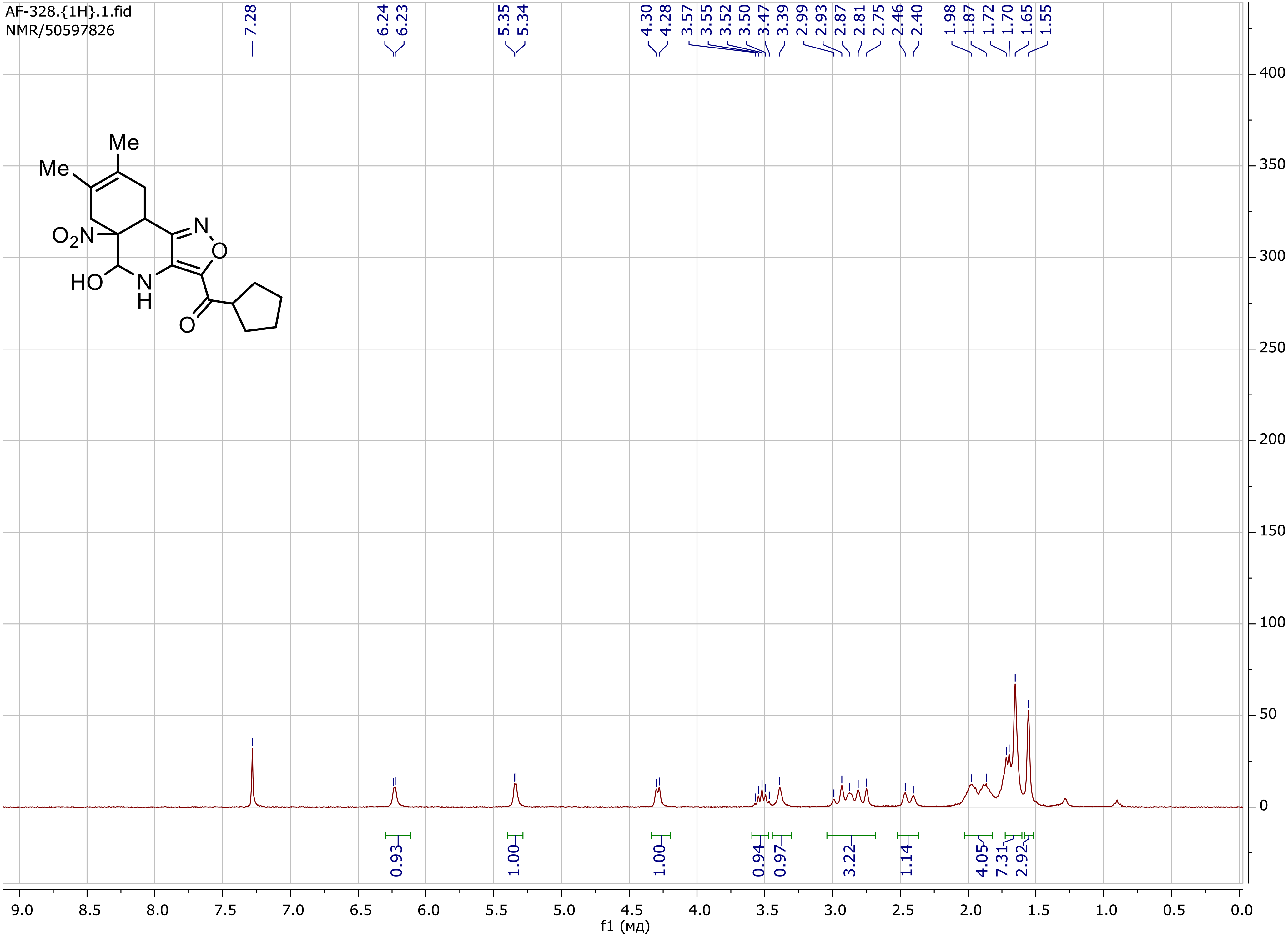
AF-328.{1H}.1.fid
NMR/50597826
7.28
6.24
6.23
5.35
5.34
4.30
4.28
3.57
3.55
3.52
3.50
3.47
3.39
2.99
2.93
2.87
2.81
2.75
2.46
2.40
1.98
1.87
1.72
1.70
1.65
1.55
Me
Me
O_2N
N
O
HO
N
H
O
0.93
1.00
1.00
0.94
0.97
3.22
1.14
4.05
7.31
2.92
f1 (мд)

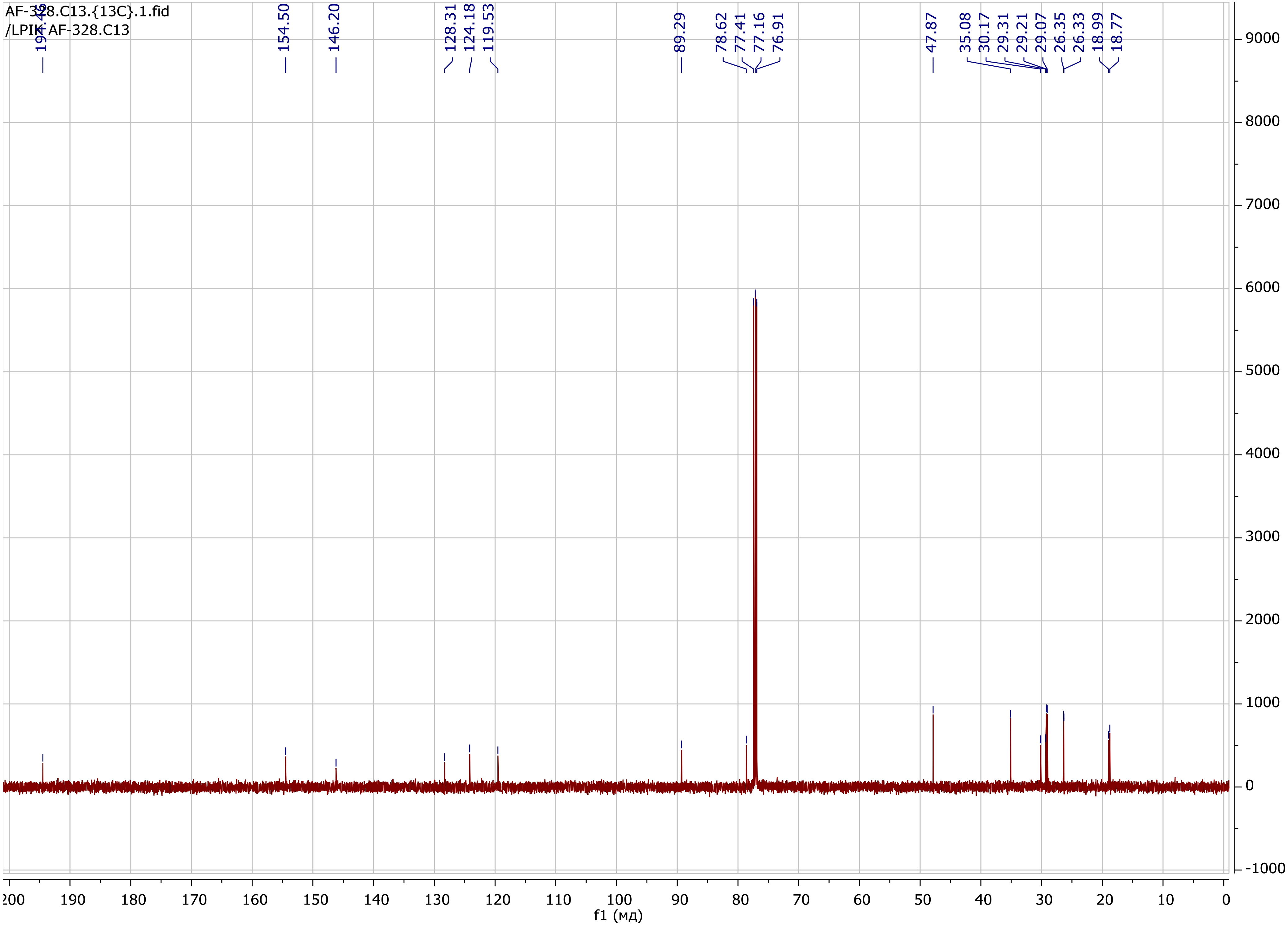
AF-328.C13.{13C}.1.fid
/LPIK AF-328.C13
194.46
154.50
146.20
128.31
124.18
119.53
89.29
78.62
77.41
77.16
76.91
47.87
35.08
30.17
29.31
29.21
29.07
26.35
26.33
18.99
18.77
9000
8000
7000
6000
5000
4000
3000
2000
1000
0
-1000
200
190
180
170
160
150
140
130
120
110
100
90
80
70
60
50
40
30
20
10
0
f1 (мд)

Display Report

Analysis Info

Analysis Name	D:\Data\Kolotyrkina\2020\Bastrakov\0121022.d	Acquisition Date	21.01.2020 16:47:06
Method	tune_50-1600_neg.m	Operator	BDAL@DE
Sample Name	/LPIK AF-328	Instrument / Ser#	micrOTOF 10248
Comment	C18H23N3O5 mH 362.1710 calibrant added CH3CN		

Acquisition Parameter

Source Type	ESI	Ion Polarity	Negative	Set Nebulizer	1.0 Bar
Focus	Not active			Set Dry Heater	200 °C
Scan Begin	50 m/z	Set Capillary	3200 V	Set Dry Gas	4.0 l/min
Scan End	1650 m/z	Set End Plate Offset	-500 V	Set Divert Valve	Waste

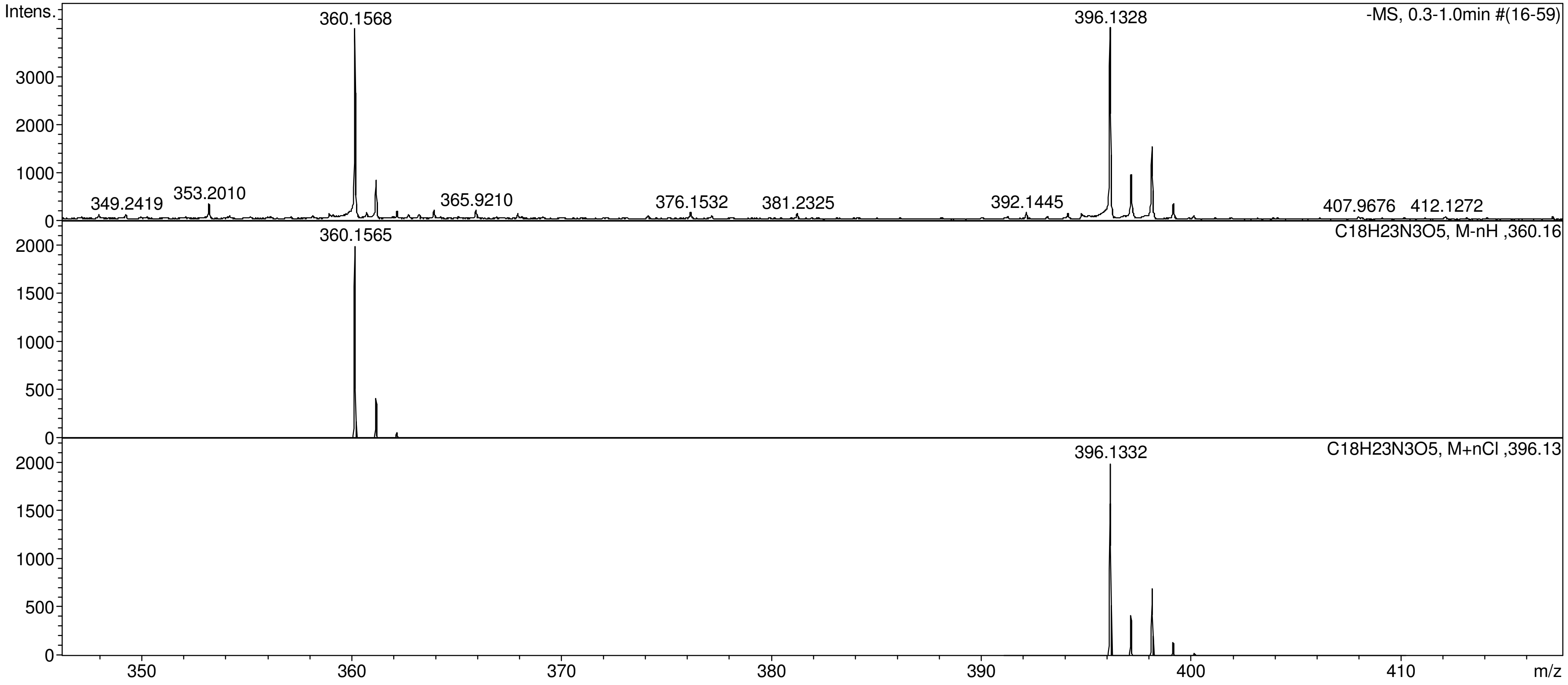

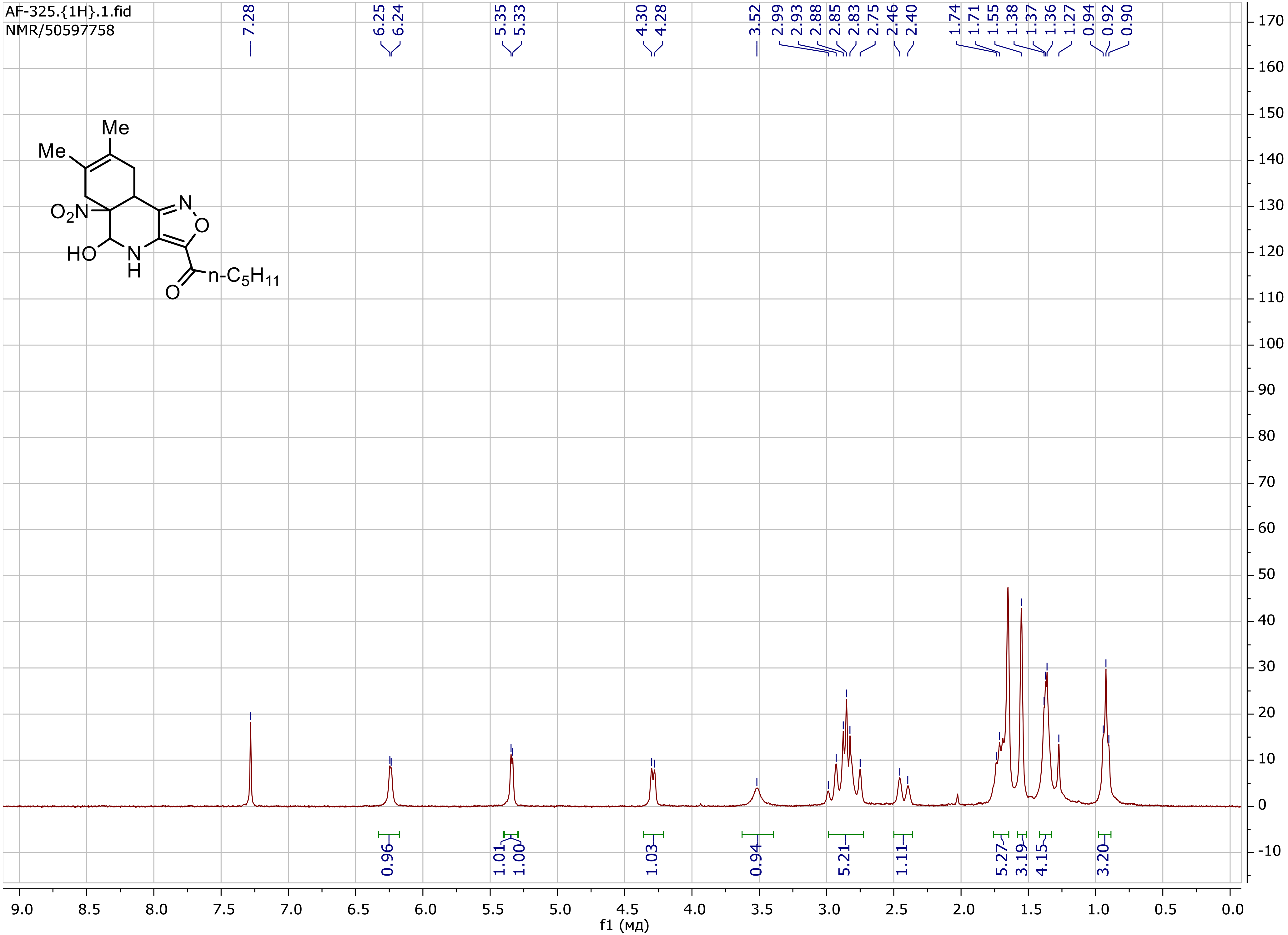
AF-325.{1H}.1.fid
NMR/50597758
7.28
6.25
6.24
5.35
5.33
4.30
4.28
3.52
2.99
2.93
2.88
2.85
2.83
2.75
2.46
2.40
1.74
1.71
1.55
1.38
1.37
1.36
1.27
0.94
0.92
0.90
Me
Me
O2N
N
O
HO
N
H
O
n-C5H11
0.96
1.01
1.00
1.03
0.94
5.21
1.11
5.27
3.19
4.15
3.20
f1 (мд)

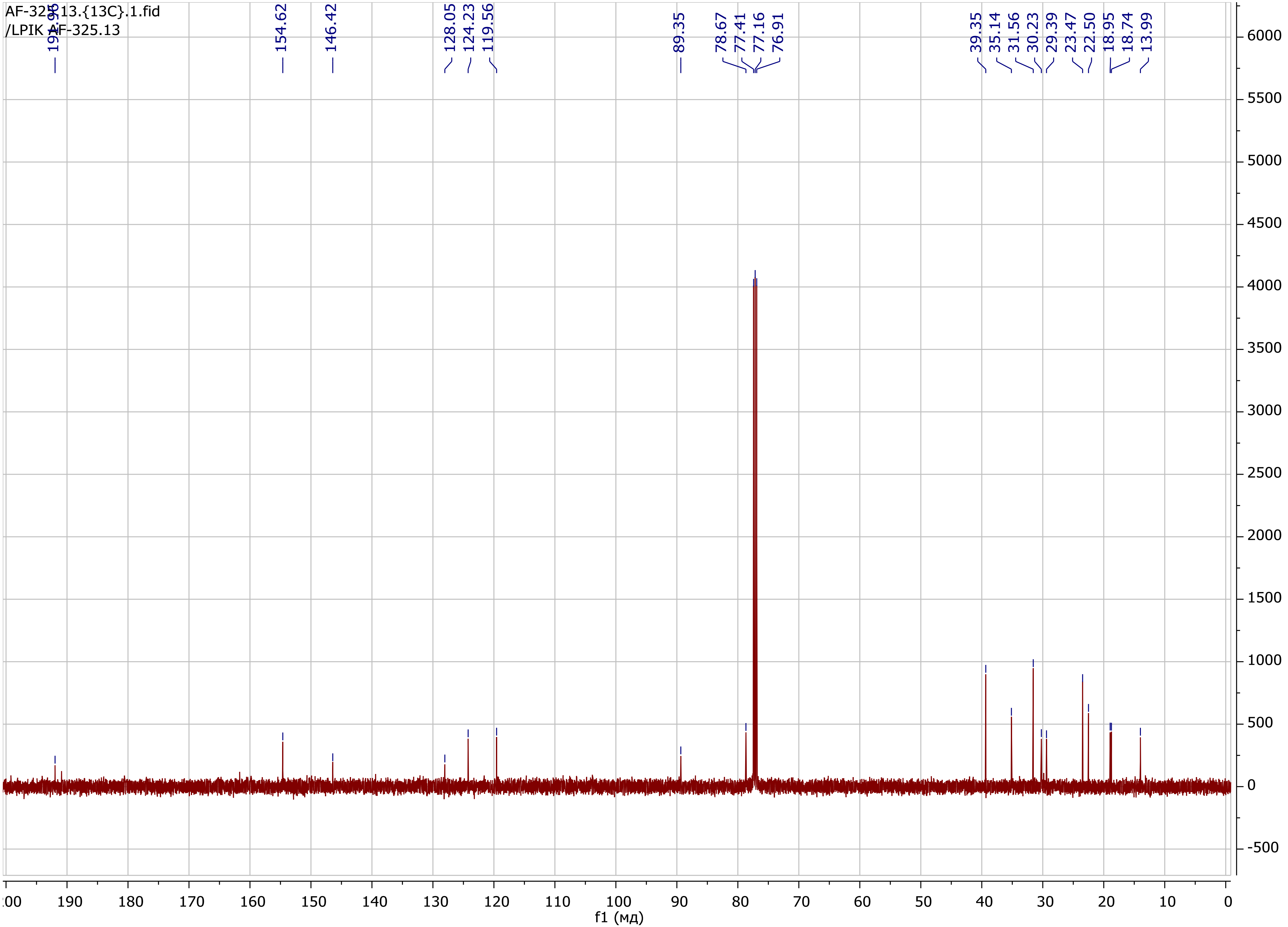

AF-325.13.{13C}.1.fid
/LPIK AF-325.13
191.96
154.62
146.42
128.05
124.23
119.56
89.35
78.67
77.41
77.16
76.91
39.35
35.14
31.56
30.23
29.39
23.47
22.50
18.95
18.74
13.99
f1 (мд)

Display Report

Analysis Info

Analysis Name	D:\Data\Kolotyrkina\2019\Bastrakov\1009018.d	Acquisition Date	09.10.2019 16:54:25
Method	tune_50-1600.m	Operator	BDAL@DE
Sample Name	/LPIK AF-325	Instrument / Ser#	micrOTOF 10248
Comment	C18H25N3O5 mH 364.1866 calibrant added		

Acquisition Parameter

Source Type	ESI	Ion Polarity	Positive	Set Nebulizer	1.0 Bar
Focus	Not active			Set Dry Heater	200 °C
Scan Begin	50 m/z	Set Capillary	4500 V	Set Dry Gas	4.0 l/min
Scan End	1600 m/z	Set End Plate Offset	-500 V	Set Divert Valve	Waste

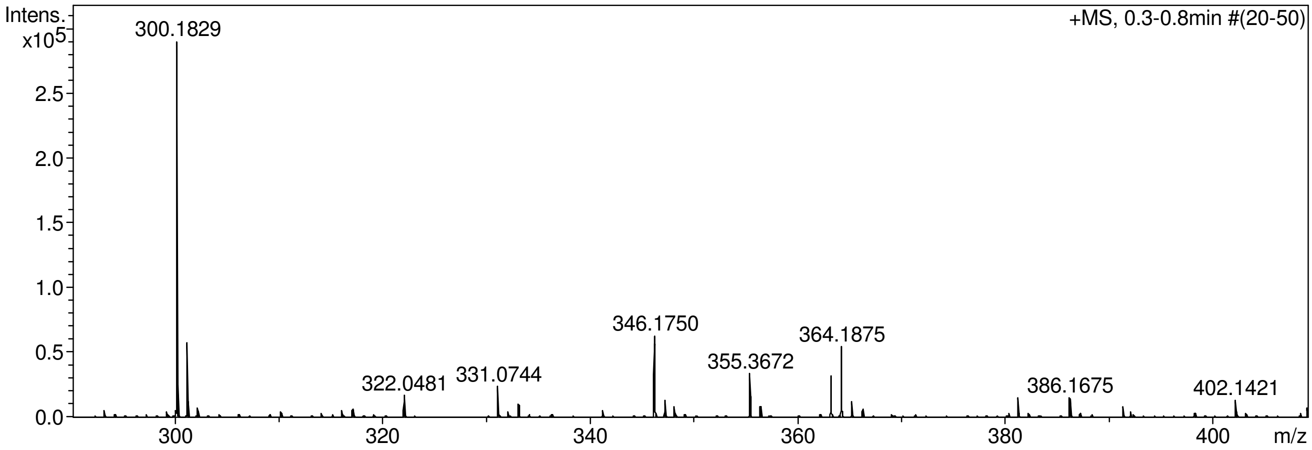

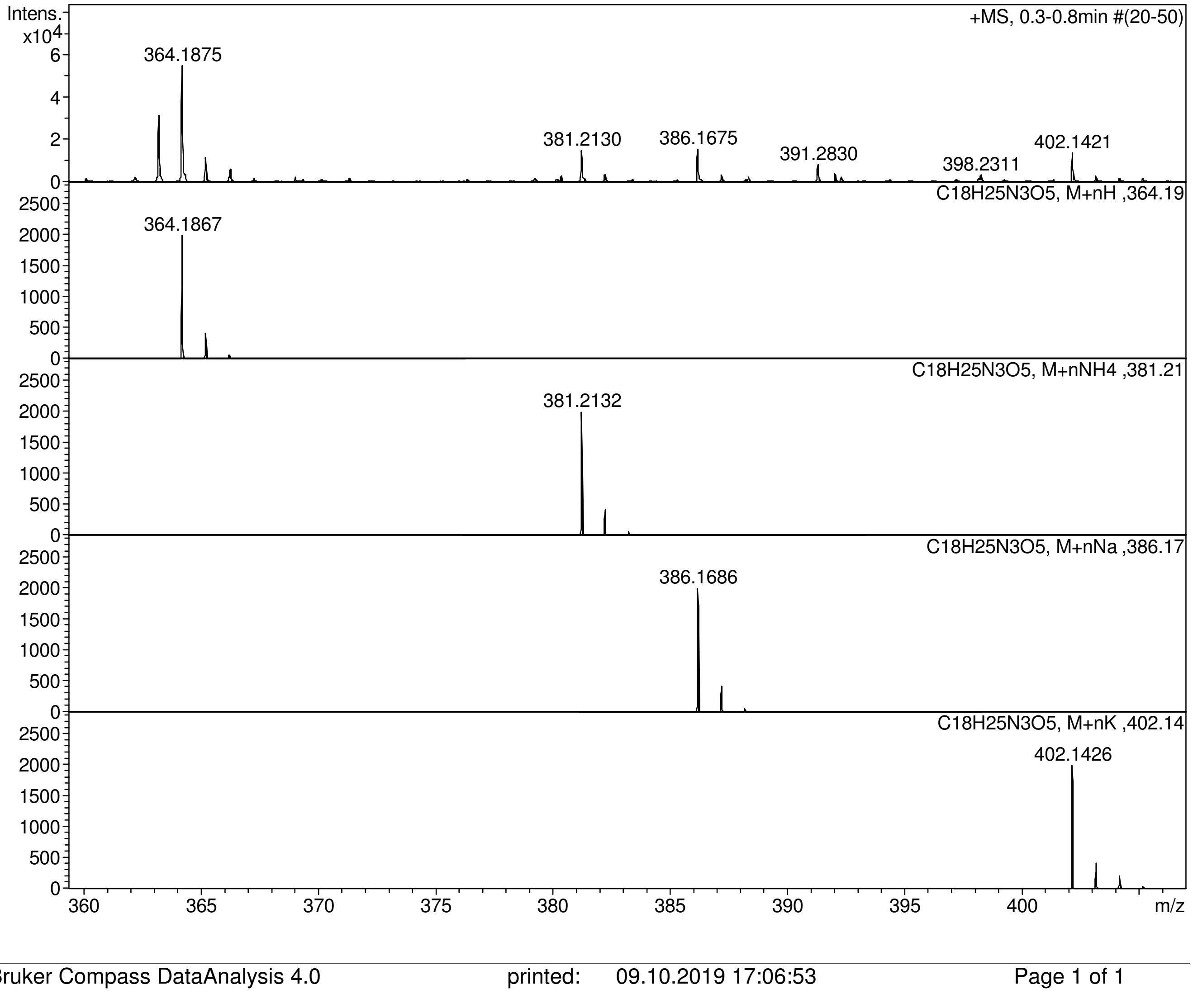

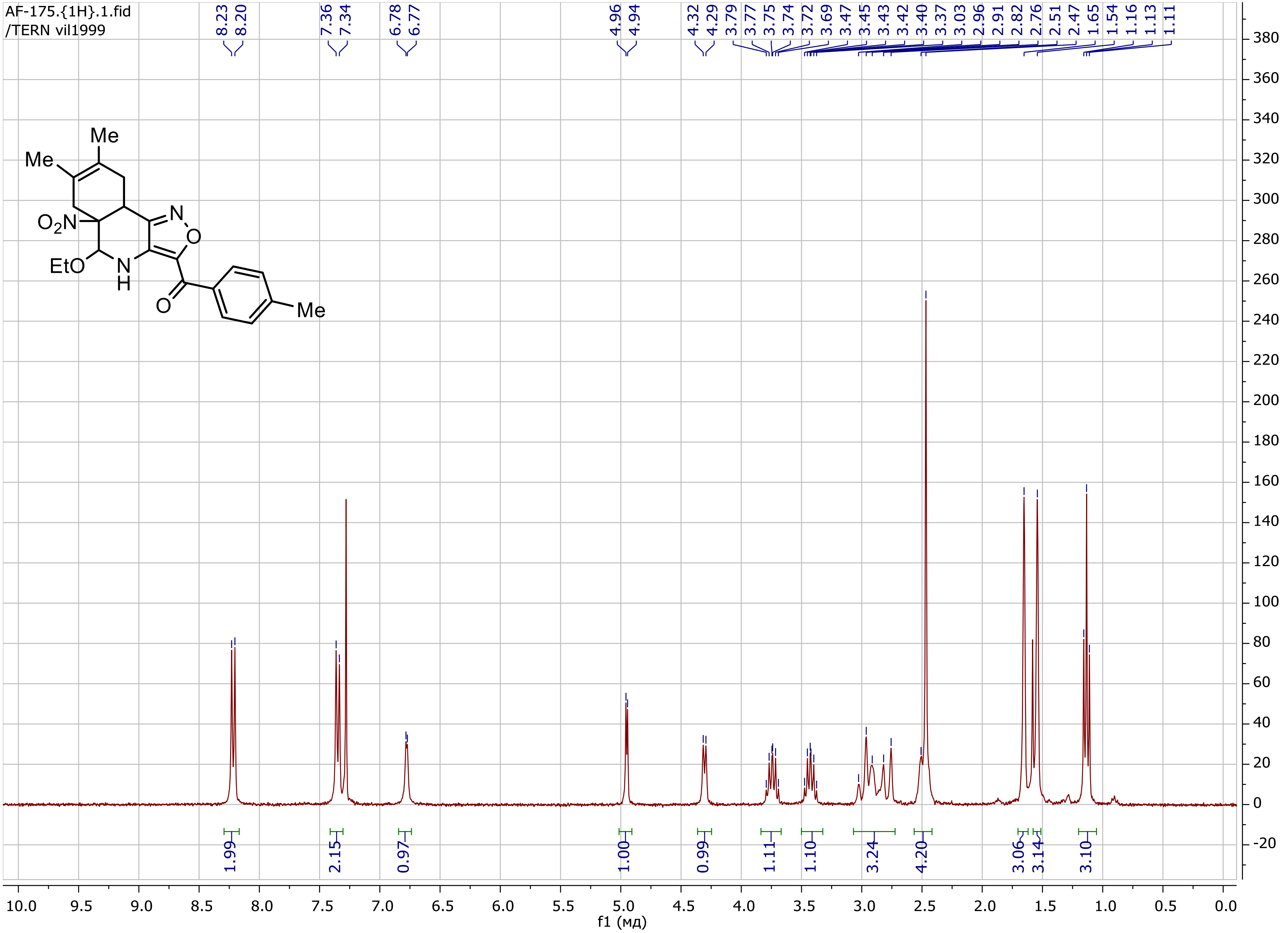
AF-175.{1H}.1.fid
/TERN vil1999
8.23
8.20
7.36
7.34
6.78
6.77
4.96
4.94
4.32
4.29
3.79
3.77
3.75
3.74
3.72
3.69
3.47
3.45
3.43
3.42
3.40
3.37
3.03
2.96
2.91
2.82
2.76
2.51
2.47
1.65
1.54
1.16
1.13
1.11
Me
Me
O_2N
N
O
EtO
N
H
O
Me
1.99
2.15
0.97
1.00
0.99
1.11
1.10
3.24
4.20
3.06
3.14
3.10
f1 (мд)

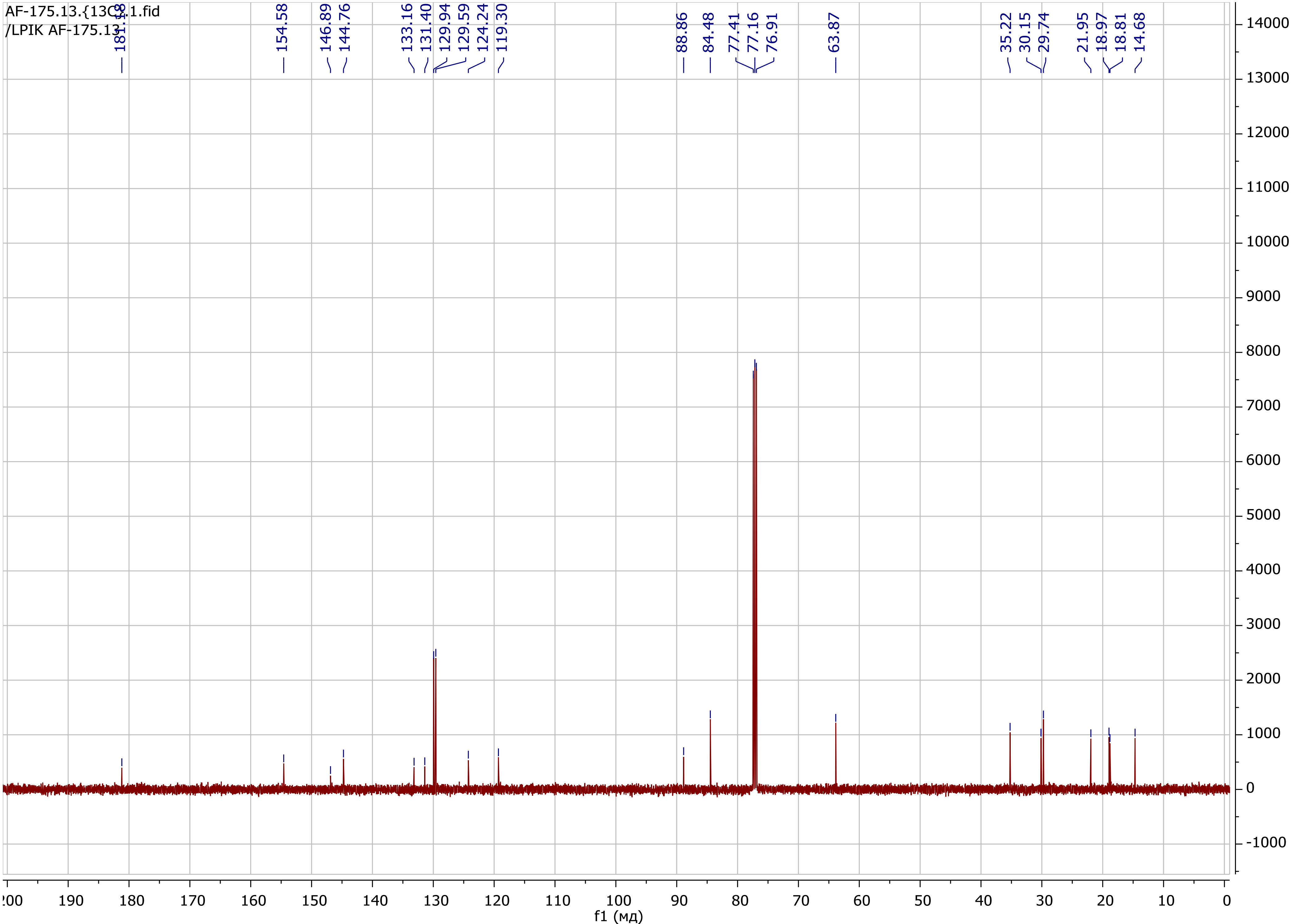
AF-175.13.{13C}.1.fid
/LPIK AF-175.13
181.18
154.58
146.89
144.76
133.16
131.40
129.94
129.59
124.24
119.30
88.86
84.48
77.41
77.16
76.91
63.87
35.22
30.15
29.74
21.95
18.97
18.81
14.68
f1 (мд)

Display Report

Analysis Info

Analysis Name	D:\Data\Kolotyrkina\2018\Bastrakov\0703002.d	Acquisition Date	03.07.2018 10:33:01
Method	tune_50-1600.m	Operator	BDAL@DE
Sample Name	/LPIK AF-175	Instrument / Ser#	micrOTOF 10248
Comment	C22H24N3O5 mH 412.1866 calibrant added		

Acquisition Parameter

Source Type	ESI	Ion Polarity	Positive	Set Nebulizer	1.0 Bar
Focus	Not active			Set Dry Heater	200 °C
Scan Begin	50 m/z	Set Capillary	4500 V	Set Dry Gas	4.0 l/min
Scan End	1600 m/z	Set End Plate Offset	-500 V	Set Divert Valve	Waste

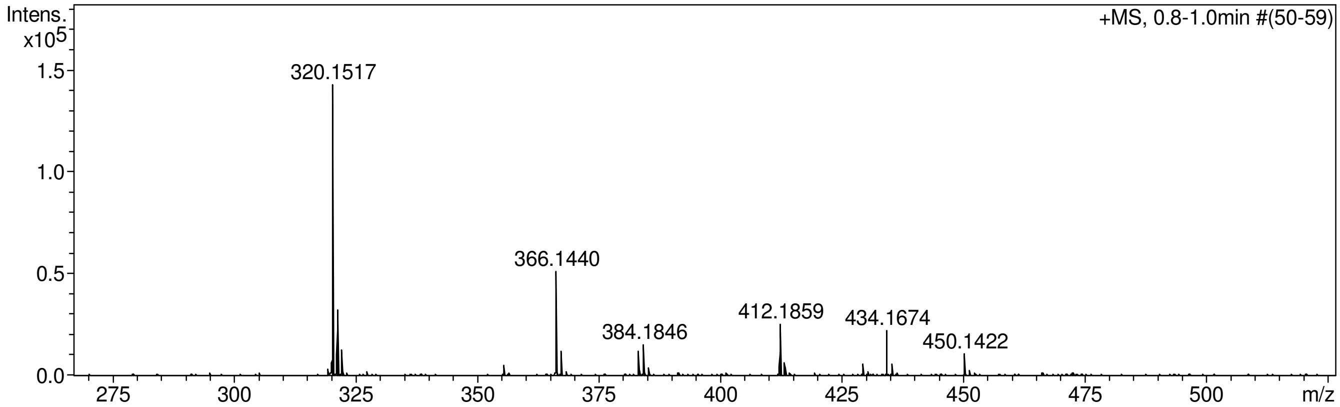

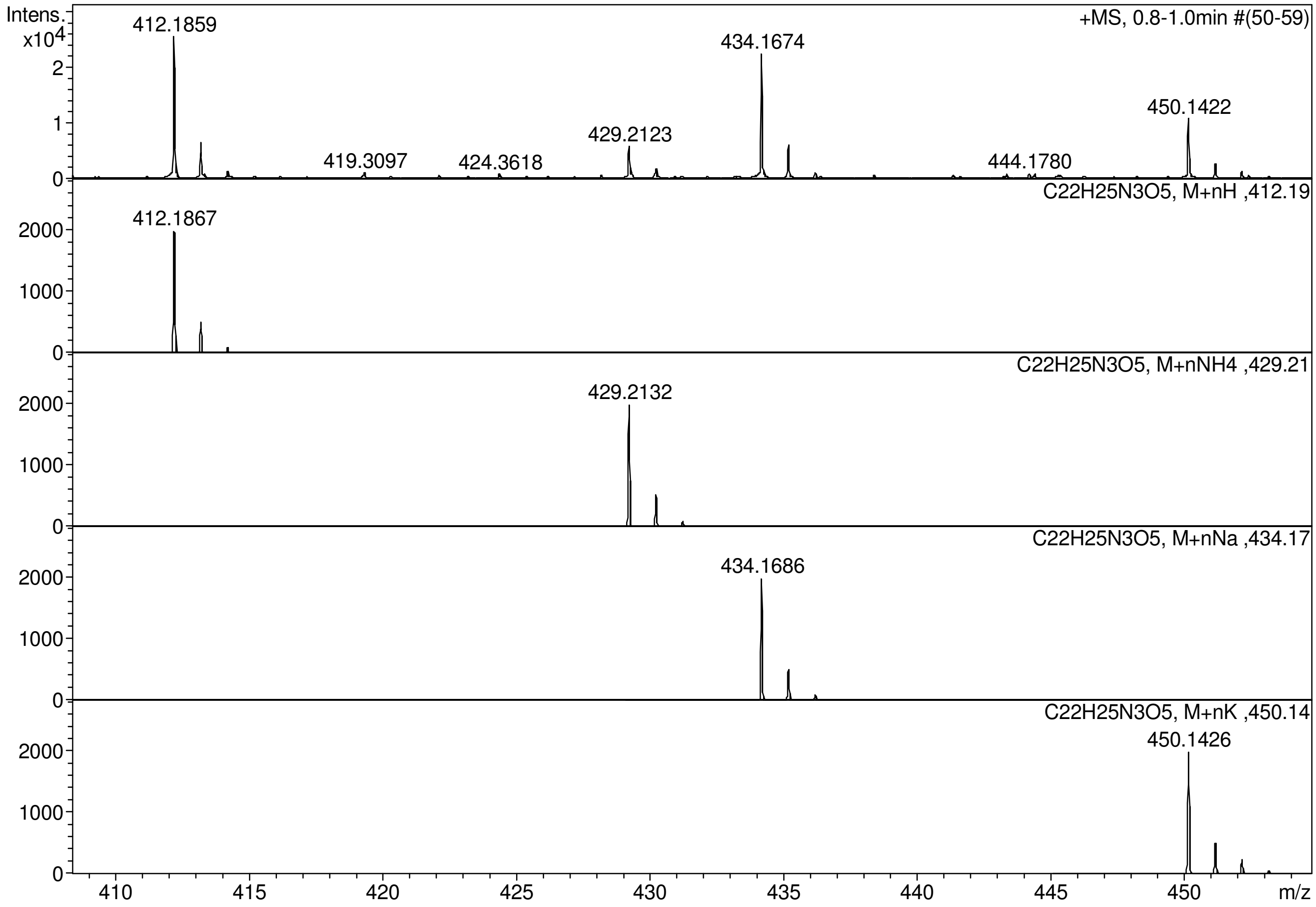

checkCIF/PLATON report

Structure factors have been supplied for datablock(s) dnp-cc-ph

THIS REPORT IS FOR GUIDANCE ONLY. IF USED AS PART OF A REVIEW PROCEDURE FOR PUBLICATION, IT SHOULD NOT REPLACE THE EXPERTISE OF AN EXPERIENCED CRYSTALLOGRAPHIC REFEREE.

No syntax errors found. CIF dictionary Interpreting this report

Datablock: dnp-cc-ph

```
Bond precision:  C-C = 0.0019 A          Wavelength=0.71073

Cell:           a=7.3210(4)    b=7.1462(4)        c=22.6111(13)
                alpha=90       beta=91.163(1)     gamma=90
Temperature:    120 K

                Calculated                 Reported
Volume          1182.71(11)                1182.71(11)
Space group     P 21/c                     P 21/c
Hall group      -P 2ybc                    -P 2ybc
Moiety formula  C13 H7 N3 O4               C13 H7 N3 O4
Sum formula     C13 H7 N3 O4               C13 H7 N3 O4
Mr              269.22                     269.22
Dx,g cm-3       1.512                      1.512
Z               4                          4
Mu (mm-1)       0.116                      0.116
F000            552.0                      552.0
F000'           552.30
h,k,lmax        10,10,31                   10,10,31
Nref            3448                       3447
Tmin,Tmax       0.968,0.979                0.388,0.433
Tmin'           0.968

Correction method= # Reported T Limits: Tmin=0.388 Tmax=0.433
AbsCorr = MULTI-SCAN

Data completeness= 1.000          Theta(max)= 29.995

R(reflections)= 0.0396( 2321)     wR2(reflections)= 0.0889( 3447)

S = 1.006                  Npar= 181
```

The following ALERTS were generated. Each ALERT has the format
test-name_ALERT_alert-type_alert-level.
Click on the hyperlinks for more details of the test.

Alert level C

```
PLAT905_ALERT_3_C Negative K value in the Analysis of Variance ...     -0.568 Report
```

Alert level G

```
PLAT230_ALERT_2_G Hirshfeld Test Diff for    C8       --C9       .        6.0 s.u.
PLAT371_ALERT_2_G Long   C(sp2)-C(sp1) Bond  C2       - C7       .       1.43 Ang.
PLAT371_ALERT_2_G Long   C(sp2)-C(sp1) Bond  C8       - C9       .       1.44 Ang.
PLAT910_ALERT_3_G Missing # of FCF Reflection(s) Below Theta(Min).          1 Note
PLAT960_ALERT_3_G Number of Intensities with I  <   - 2*sig(I) ...          6 Check
PLAT978_ALERT_2_G Number C-C Bonds with Positive Residual Density.         14 Info
PLAT992_ALERT_5_G Repd & Actual _reflns_number_gt Values Differ by          1 Check
```

0 **ALERT level A** = Most likely a serious problem - resolve or explain
0 **ALERT level B** = A potentially serious problem, consider carefully
1 **ALERT level C** = Check. Ensure it is not caused by an omission or oversight
7 **ALERT level G** = General information/check it is not something unexpected

0 ALERT type 1 CIF construction/syntax error, inconsistent or missing data
4 ALERT type 2 Indicator that the structure model may be wrong or deficient
3 ALERT type 3 Indicator that the structure quality may be low
0 ALERT type 4 Improvement, methodology, query or suggestion
1 ALERT type 5 Informative message, check

It is advisable to attempt to resolve as many as possible of the alerts in all categories. Often the minor alerts point to easily fixed oversights, errors and omissions in your CIF or refinement strategy, so attention to these fine details can be worthwhile. In order to resolve some of the more serious problems it may be necessary to carry out additional measurements or structure refinements. However, the purpose of your study may justify the reported deviations and the more serious of these should normally be commented upon in the discussion or experimental section of a paper or in the "special_details" fields of the CIF. checkCIF was carefully designed to identify outliers and unusual parameters, but every test has its limitations and alerts that are not important in a particular case may appear. Conversely, the absence of alerts does not guarantee there are no aspects of the results needing attention. It is up to the individual to critically assess their own results and, if necessary, seek expert advice.

Publication of your CIF in IUCr journals

A basic structural check has been run on your CIF. These basic checks will be run on all CIFs submitted for publication in IUCr journals (*Acta Crystallographica*, *Journal of Applied Crystallography*, *Journal of Synchrotron Radiation*); however, if you intend to submit to *Acta Crystallographica Section C* or *E* or *IUCrData*, you should make sure that full publication checks are run on the final version of your CIF prior to submission.

Publication of your CIF in other journals

Please refer to the *Notes for Authors* of the relevant journal for any special instructions relating to CIF submission.

PLATON version of 22/12/2019; check.def file version of 13/12/2019

Datablock dnp-cc-ph - ellipsoid plot

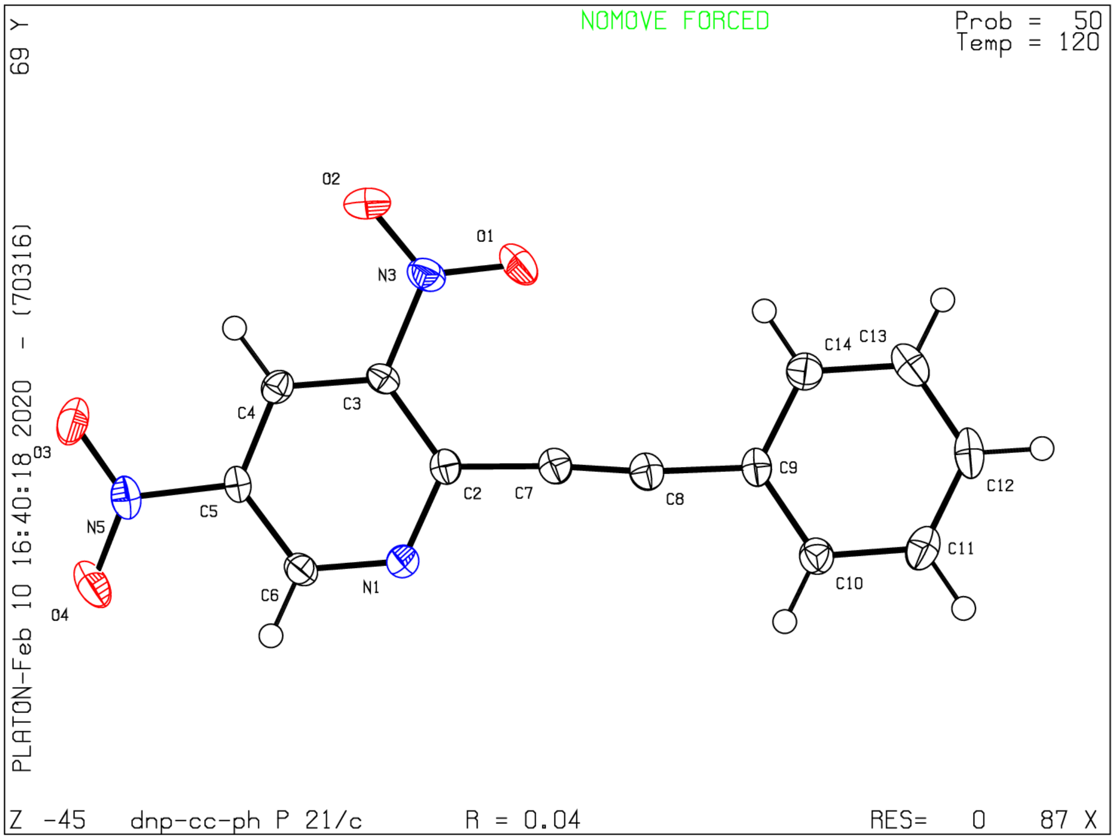

checkCIF/PLATON report

Structure factors have been supplied for datablock(s) dnp-cc-phf

THIS REPORT IS FOR GUIDANCE ONLY. IF USED AS PART OF A REVIEW PROCEDURE FOR PUBLICATION, IT SHOULD NOT REPLACE THE EXPERTISE OF AN EXPERIENCED CRYSTALLOGRAPHIC REFEREE.

No syntax errors found. CIF dictionary Interpreting this report

Datablock: dnp-cc-phf

```
Bond precision:  C-C = 0.0019 A          Wavelength=0.71073

Cell:            a=7.2818(5)    b=7.1802(4)        c=22.6490(14)
                 alpha=90       beta=90.592(1)     gamma=90
Temperature:     120 K

                Calculated                 Reported
Volume          1184.14(13)                1184.13(13)
Space group     P 21/c                     P 21/c
Hall group      -P 2ybc                    -P 2ybc
Moiety formula  C13 H6 F N3 O4             C13 H6 F N3 O4
Sum formula     C13 H6 F N3 O4             C13 H6 F N3 O4
Mr              287.21                     287.21
Dx,g cm-3       1.611                      1.611
Z               4                          4
Mu (mm-1)       0.133                      0.133
F000            584.0                      584.0
F000'           584.36
h,k,lmax        10,10,31                   10,10,31
Nref            3454                       3454
Tmin,Tmax       0.947,0.961                0.810,0.862
Tmin'           0.946

Correction method= # Reported T Limits: Tmin=0.810 Tmax=0.862
AbsCorr = MULTI-SCAN

Data completeness= 1.000          Theta(max)= 29.997

R(reflections)= 0.0438( 2424)     wR2(reflections)= 0.0992( 3454)

S = 1.033                  Npar= 190
```

The following ALERTS were generated. Each ALERT has the format
test-name_ALERT_alert-type_alert-level.
Click on the hyperlinks for more details of the test.

Alert level C

```
PLAT906_ALERT_3_C Large K Value in the Analysis of Variance ......      2.595 Check
```

Alert level G

```
PLAT371_ALERT_2_G Long   C(sp2)-C(sp1) Bond  C2       - C7       .       1.43 Ang.
PLAT371_ALERT_2_G Long   C(sp2)-C(sp1) Bond  C8       - C9       .       1.43 Ang.
PLAT960_ALERT_3_G Number of Intensities with I  <   - 2*sig(I) ...          5 Check
PLAT978_ALERT_2_G Number C-C Bonds with Positive Residual Density.         12 Info
```

```
   0 ALERT level A = Most likely a serious problem - resolve or explain
   0 ALERT level B = A potentially serious problem, consider carefully
   1 ALERT level C = Check. Ensure it is not caused by an omission or oversight
   4 ALERT level G = General information/check it is not something unexpected

   0 ALERT type 1 CIF construction/syntax error, inconsistent or missing data
   3 ALERT type 2 Indicator that the structure model may be wrong or deficient
   2 ALERT type 3 Indicator that the structure quality may be low
   0 ALERT type 4 Improvement, methodology, query or suggestion
   0 ALERT type 5 Informative message, check
```

It is advisable to attempt to resolve as many as possible of the alerts in all categories. Often the minor alerts point to easily fixed oversights, errors and omissions in your CIF or refinement strategy, so attention to these fine details can be worthwhile. In order to resolve some of the more serious problems it may be necessary to carry out additional measurements or structure refinements. However, the purpose of your study may justify the reported deviations and the more serious of these should normally be commented upon in the discussion or experimental section of a paper or in the "special_details" fields of the CIF. checkCIF was carefully designed to identify outliers and unusual parameters, but every test has its limitations and alerts that are not important in a particular case may appear. Conversely, the absence of alerts does not guarantee there are no aspects of the results needing attention. It is up to the individual to critically assess their own results and, if necessary, seek expert advice.

Publication of your CIF in IUCr journals

A basic structural check has been run on your CIF. These basic checks will be run on all CIFs submitted for publication in IUCr journals (*Acta Crystallographica*, *Journal of Applied Crystallography*, *Journal of Synchrotron Radiation*); however, if you intend to submit to *Acta Crystallographica Section C* or *E* or *IUCrData*, you should make sure that full publication checks are run on the final version of your CIF prior to submission.

Publication of your CIF in other journals

Please refer to the *Notes for Authors* of the relevant journal for any special instructions relating to CIF submission.

PLATON version of 22/12/2019; check.def file version of 13/12/2019

Datablock dnp-cc-phf - ellipsoid plot

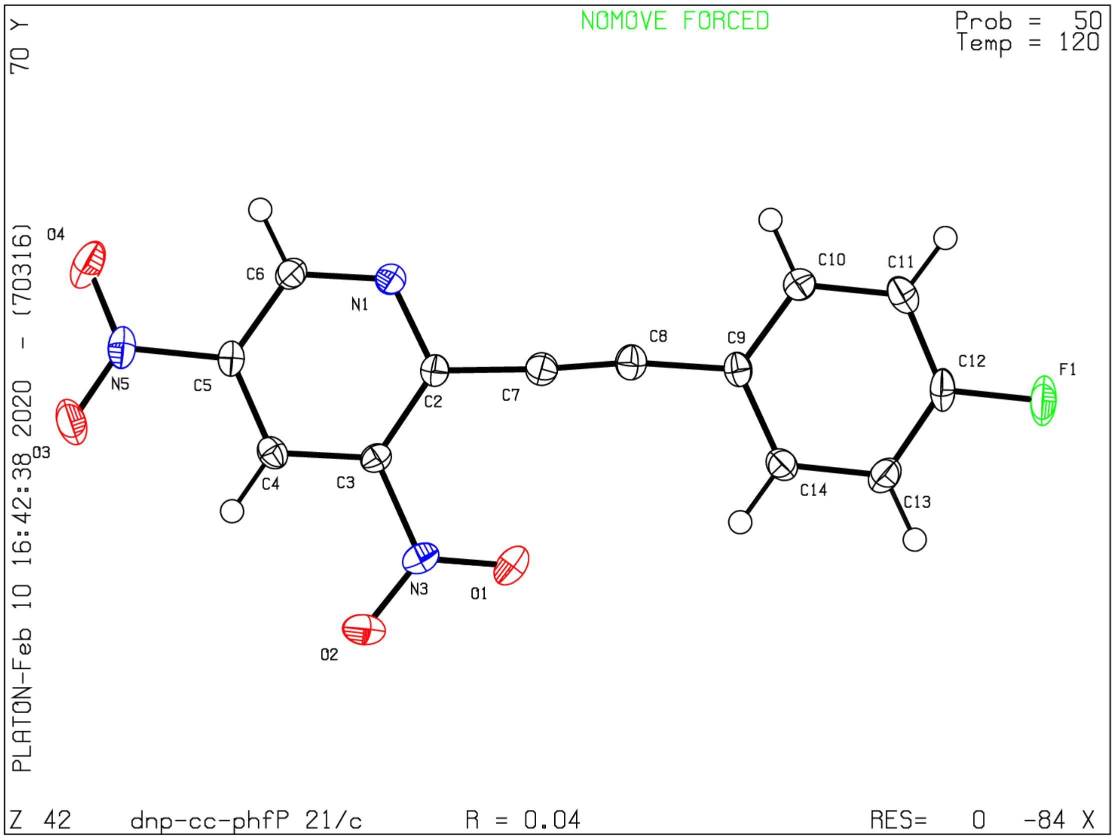

checkCIF/PLATON report

Structure factors have been supplied for datablock(s) ka-14

THIS REPORT IS FOR GUIDANCE ONLY. IF USED AS PART OF A REVIEW PROCEDURE FOR PUBLICATION, IT SHOULD NOT REPLACE THE EXPERTISE OF AN EXPERIENCED CRYSTALLOGRAPHIC REFEREE.

No syntax errors found.   CIF dictionary   Interpreting this report

Datablock: ka-14

```
Bond precision:  C-C = 0.0023 A          Wavelength=0.71073

Cell:             a=6.6772(4)      b=7.2749(4)      c=25.0344(14)
                  alpha=90         beta=90          gamma=90
Temperature:      120 K

                Calculated                 Reported
Volume          1216.07(12)                1216.07(12)
Space group     P 21 21 21                 P 21 21 21
Hall group      P 2ac 2ab                  P 2ac 2ab
Moiety formula  C14 H9 N3 O4               C14 H9 N3 O4
Sum formula     C14 H9 N3 O4               C14 H9 N3 O4
Mr              283.24                     283.24
Dx,g cm-3       1.547                      1.547
Z               4                          4
Mu (mm-1)       0.117                      0.117
F000            584.0                      584.0
F000'           584.31
h,k,lmax        9,10,35                    9,10,35
Nref            3546[ 2069]                3548
Tmin,Tmax       0.970,0.979                0.832,0.862
Tmin'           0.969

Correction method= # Reported T Limits: Tmin=0.832 Tmax=0.862
AbsCorr = MULTI-SCAN

Data completeness= 1.71/1.00      Theta(max)= 29.996

R(reflections)= 0.0357( 3328)     wR2(reflections)= 0.0920( 3548)

S = 1.028                   Npar= 191
```

The following ALERTS were generated. Each ALERT has the format
test-name_ALERT_alert-type_alert-level.
Click on the hyperlinks for more details of the test.

Alert level G

```
PLAT910_ALERT_3_G Missing # of FCF Reflection(s) Below Theta(Min).          1 Note
PLAT933_ALERT_2_G Number of OMIT Records in Embedded .res File ...          1 Note
PLAT978_ALERT_2_G Number C-C Bonds with Positive Residual Density.         14 Info
```

```
   0 ALERT level A = Most likely a serious problem - resolve or explain
   0 ALERT level B = A potentially serious problem, consider carefully
   0 ALERT level C = Check. Ensure it is not caused by an omission or oversight
   3 ALERT level G = General information/check it is not something unexpected

   0 ALERT type 1 CIF construction/syntax error, inconsistent or missing data
   2 ALERT type 2 Indicator that the structure model may be wrong or deficient
   1 ALERT type 3 Indicator that the structure quality may be low
   0 ALERT type 4 Improvement, methodology, query or suggestion
   0 ALERT type 5 Informative message, check
```

It is advisable to attempt to resolve as many as possible of the alerts in all categories. Often the minor alerts point to easily fixed oversights, errors and omissions in your CIF or refinement strategy, so attention to these fine details can be worthwhile. In order to resolve some of the more serious problems it may be necessary to carry out additional measurements or structure refinements. However, the purpose of your study may justify the reported deviations and the more serious of these should normally be commented upon in the discussion or experimental section of a paper or in the "special_details" fields of the CIF. checkCIF was carefully designed to identify outliers and unusual parameters, but every test has its limitations and alerts that are not important in a particular case may appear. Conversely, the absence of alerts does not guarantee there are no aspects of the results needing attention. It is up to the individual to critically assess their own results and, if necessary, seek expert advice.

Publication of your CIF in IUCr journals

A basic structural check has been run on your CIF. These basic checks will be run on all CIFs submitted for publication in IUCr journals (*Acta Crystallographica*, *Journal of Applied Crystallography*, *Journal of Synchrotron Radiation*); however, if you intend to submit to *Acta Crystallographica Section C* or *E* or *IUCrData*, you should make sure that full publication checks are run on the final version of your CIF prior to submission.

Publication of your CIF in other journals

Please refer to the *Notes for Authors* of the relevant journal for any special instructions relating to CIF submission.

PLATON version of 22/12/2019; check.def file version of 13/12/2019

Datablock ka-14 - ellipsoid plot

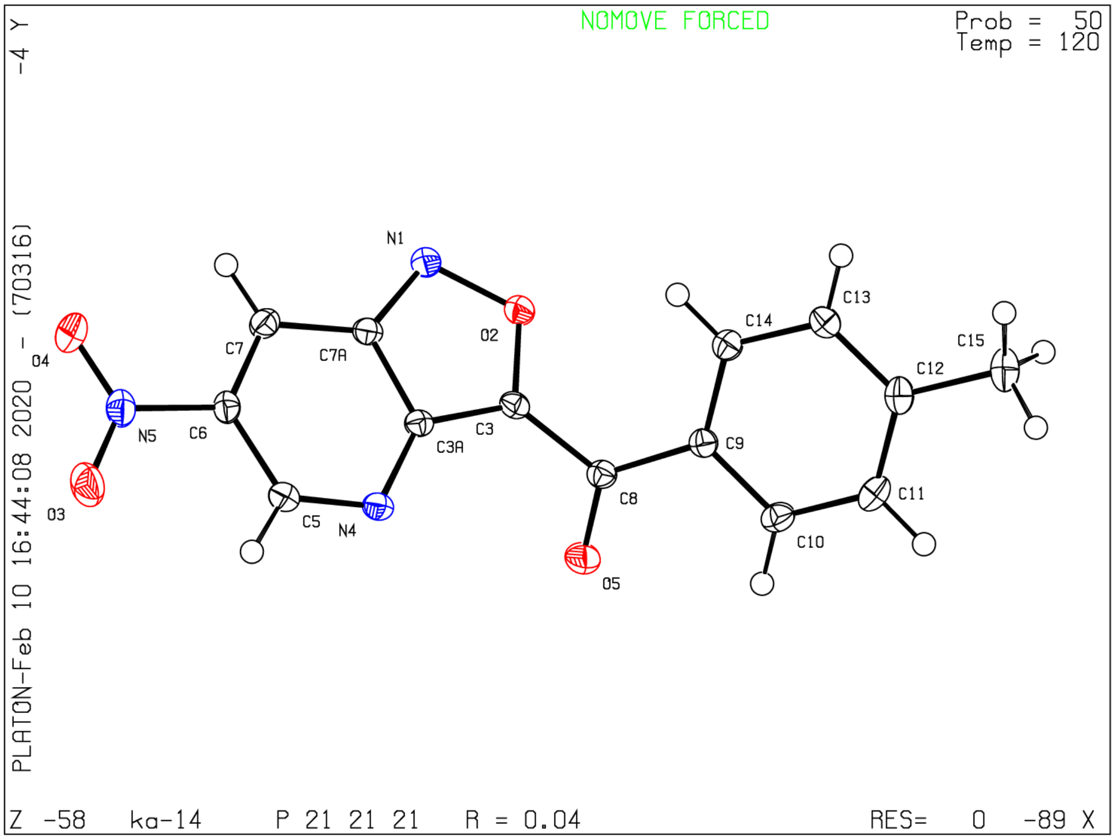

Table 3. Crystallographic data for **2a, 2c** and **3b**.

	**2a**	**2c**	**3b**
CCDC	1983532	1983530	1983531
Formula	$C_{13}H_7N_3O_4$	$C_{13}H_6FN_3O_4$	$C_{14}H_9N_3O_4$
Formula weight	269.22	287.21	283.24
T, K	120	120	120
Crystal system	monoclinic	monoclinic	orthorhombic
Space group	$P2_1/c$	$P2_1/c$	$P2_12_12_1$
Z / Z'	4 / 1	4 / 1	4 / 1
a, Å	7.3210(4)	7.2818(5)	6.6772(4)
b, Å	7.1462(4)	7.1802(4)	7.2749(4)
c, Å	22.6111(13)	22.6490(14)	25.0344(14)
β, °	91.1630(10)	90.5920(10)	
V, Å^3	1182.71(11)	1184.13(13)	1216.07(12)
d_{calc}, g cm^{-3}	1.512	1.611	1.547
μ, cm^{-1}	1.16	1.33	1.17
$2\theta_{max}$, °	60	60	60
Reflns. collected / independent	19354 / 3447	14535 / 3454	16184 / 3548
Observed reflections [I>2σ(I)]	2321	2424	3328
R_1	0.0396	0.0438	0.0357
wR_2	0.0889	0.0992	0.0920
GOF	1.006	1.033	1.028
Residual density, e Å^{-3} (d_{max}/d_{min})	0.303/-0.234	0.395/-0.238	0.396/-0.238
